# Regulatory mechanism of ABCB1 transcriptional repression by HDAC5 in rat hepatocytes under hypoxic environment

**DOI:** 10.3389/fphys.2025.1520246

**Published:** 2025-04-08

**Authors:** Ziqin Wei, Hongfang Mu, Fangfang Qiu, Minghui Zhao, Xiaojing Zhang, Wenbin Li, Hai Jia, Rong Wang

**Affiliations:** ^1^ School of Pharmacy, Lanzhou University, Lanzhou, China; ^2^ PLA Key Laboratory of the Plateau of the Environmental Damage Control, The 940th Hospital of Logistics Support Force of PLA, Lanzhou, China; ^3^ Gansu Provincial People's Hospital, Lanzhou, China

**Keywords:** hypoxia, ABCB1, HDAC inhibitor, HDAC5, Sp1, H3K9ac

## Abstract

**Objective:**

Previous research has demonstrated that the hypoxic environment at high altitudes significantly alters the pharmacokinetics of many drugs, reducing their efficacy and increasing adverse effects. A key factor in this altered drug metabolism is the inhibition of ATP-binding cassette subfamily B member 1 (ABCB1), an efflux transporter protein, in the liver tissues of plateau rats. Rat ABCB1, encoded by the *ABCB1A* and *ABCB1B* genes, has two isoforms functionally analogous to human ABCB1. Histone acetylation, an epigenetic mechanism, may regulate ABCB1 transcription in hypoxic conditions by modifying chromatin structure and interacting with signaling pathways. However, its role in ABCB1 transcriptional regulation under hypoxia remains unclear. Based on this, the present study employed the BRL cell line to establish a hypoxia model, aiming to investigate the histone acetylation-mediated regulatory mechanisms of ABCB1 expression under hypoxic conditions, with the ultimate goal of providing novel theoretical foundations for rational drug use in high-altitude regions.

**Methods:**

Establishment of BRL hypoxia model: BRL cell viability was detected by CCK-8 assay, and HIF-1α expression was measured by RT-qPCR and Western blot. After treating the BRL hypoxia model with HDAC inhibitors, ABCB1 and HDAC5 expression were detected by RT-qPCR, Western blot, and immunofluorescence. Rhodamine 123 accumulation assay was performed to examine the effect of HDAC inhibitors on ABCB1 functional activity. HDAC5 was targeted by siRNA technology to detect ABCB1 and H3K9ac expression. CUT&Tag assay was used to measure H3K9ac levels at the ABCB1 promoter region. After SAHA treatment of the BRL hypoxia model, SP1 expression was detected by RT-qPCR and Western blot. Combined treatment with SAHA and siRNA targeting SP1 was performed to detect ABCB1 expression. Co-immunoprecipitation and fluorescence colocalization assays were conducted to examine interactions among SP1, HDAC5, and ABCB1.

**Results:**

After hypoxic culture for different durations, cell viability decreased while HIF-1α expression increased, indicating the successful establishment of the BRL hypoxia model. In the BRL hypoxia model, ABCB1 and SP1 expression decreased while HDAC5 expression increased. After SAHA treatment, ABCB1 and SP1 expression were upregulated while HDAC5 was downregulated. Rhodamine 123 accumulation assay showed that SAHA could enhance ABCB1 functional activity by inducing its expression. After HDAC5 was knocked down using siRNA, ABCB1 and H3K9ac expression increased, and ABCB1 functional activity was enhanced. CUT&Tag assay demonstrated that H3K9ac levels at the *ABCB1B* promoter region decreased in the BRL hypoxia model, while HDAC5 inhibition increased H3K9ac levels at this region. After SP1 was knocked down using siRNA, the inductive effect of SAHA on ABCB1 was blocked. Co-immunoprecipitation and fluorescence colocalization showed interactions among SP1, HDAC5, and ABCB1.

**Conclusion:**

In BRL cells, HDAC5 may be recruited by SP1 to form a complex, reducing free HDAC5, increasing H3K9ac at the *ABCB1B* promoter, and activating ABCB1 transcription. In the BRL hypoxia model, disruption of the SP1-HDAC5 complex increased free HDAC5, lowered H3K9ac at the *ABCB1B* promoter, and suppressed ABCB1 transcription. These results suggest that HDAC inhibitors enhance ABCB1 expression in hypoxic environments, indicating that combining HDAC inhibitors with therapeutic agents could mitigate reduced drug efficacy and adverse effects caused by ABCB1 suppression.

## 1 Introduction

Previous studies have shown that the pharmacokinetics of drugs such as norfloxacin, furosemide, sildenafil, and levofloxacin are significantly altered after acute or chronic exposure of rats to high-altitude environments. This consequently affects the efficacy of the drugs and leads to adverse effects (Luo et al., 2017; Luo et al., 2018; Zhang and Wang, 2022; Li et al., 2016). Hypoxia, the most prominent characteristic of high-altitude environments, becomes a crucial influence in this process. ATP-binding cassette transporters are a well-researched superfamily of transporters with a wide range of substrates and are important for the physiological and pharmacological effects of drugs *in vivo*. ABCB1, as one of its representative transporters, is widely distributed in the body, indicating that it plays a key role in drug metabolism (Li et al., 2015). It has been reported that the alteration of ABCB1 expression in vital organs, such as the liver, kidney, small intestine, and brain, significantly affects the absorption, distribution, metabolism, and excretion processes of drugs in low-pressure oxygen chambers in plateau hypoxia or simulated hypoxia in rats (Luo et al., 2016; Ding et al., 2019). ABCB1 expression is tightly regulated by multiple transcriptional and translational mechanisms. Its promoter and its vicinity contain multiple transcription factor recognition sites, such as GC-rich region, hypoxia-inducible factor 1 (HIF-1) response element, activating protein 1 (AP-1) binding site, CCAAT box, *etc.* Currently, the most studied transcription factors include HIF-1, nuclear transcription factor-κB (NF-κB), pregnane X receptor (PXR), nuclear transcription factor Y (NF-Y), constitutive androstane receptor (CAR), AP-1, p53, SP1, etc (Chen et al., 2016; Song et al., 2023; Chen et al., 2014; Guo et al., 2018; Phatak et al., 2021; Xu et al., 2012; Wang et al., 2023; You et al., 2020). Transcription factors more closely related to hypoxia include HIF-1, NF-κB, PXR, CAR, *etc.* (Liang et al., 2013; Duan et al., 2022; Min et al., 2019). Overall, ABCB1 is synergistically regulated by multiple signaling pathways.

Recently, histone acetylation has received attention as a potential epigenetic mechanism regulating ABCB1 transcription (Yang et al., 2019; You et al., 2020; Zhao et al., 2023). Histone acetylation enhances the ability of histones to bind to negatively charged DNA by neutralizing the positive charge of lysine, resulting in a sparse local chromatin structure that promotes gene transcription (Grunstein, 1997; Tessarz and Kouzarides, 2014). Whereas HDAC restores the positive charge of lysine and represses gene transcription (Sun et al., 2022). The HDAC family is divided into four major classes. Class I is HDAC1, HDAC2, HDAC3, and HDAC8; class IIa is HDAC4, HDAC5, HDAC7, and HDAC9; class IIb is HDAC6 and HDAC10; class III is the Sirtuins family; and class IV is HDAC11. HDACs have emerged as a key target for cancer therapy (Zhang et al., 2021). Its inhibitors, including valproic acid, SAHA, and romidepsin (ROMI), have been approved by the Federal Drug Administration for a variety of clinical indications (Wawruszak et al., 2021; Chen et al., 2017; Bachy et al., 2022).

Tumors are one of the active areas of epigenetic modification research and their microenvironment is characterized by hypoxia (Zaib et al., 2022; Yoo and Jones, 2006; Ramaiah et al., 2021). Several studies have indicated that overexpression of ABCB1 in tumor cells is correlated with increased levels of H3ac, H3K4me2, H3K4me3, H3K9ac, and H4ac in the promoter region of the gene (Chen et al., 2016). Similarly, the downregulation of ABCB1 expression in cancer cells is associated with a reduction in H3/H4ac, and changes in ABCB1 expression and/or activity can be observed with the use of HDAC inhibitors (You et al., 2020; Ni et al., 2015). TSA reversed the decrease in ABCB1 expression in canine lymphoma cell lines and SW620 cells by increasing H3ac levels in the ABCB1 promoter region (Tomiyasu et al., 2014). TSA induces the expression of ABCB1 in small-cell lung cancer cell lines by increasing the levels of H3/H4ac (El-Khoury et al., 2007). The HDAC inhibitor sodium butyrate increases ABCB1 expression in lung and colorectal cancer cells by elevating H3K9ac levels through the inhibition of HDAC4 (Shi et al., 2020). SAHA and TSA may increase the expression of ABCB1 in lung and colorectal cancer cells by downregulating HDAC3 or HDAC4 (Wang et al., 2019). Collectively, the above studies point to an epigenetic mechanism that regulates ABCB1 expression in cancer cells through histone acetylation. Therefore, we hypothesized that ABCB1 expression in a hypoxic environment is also regulated by epigenetic modifications. Currently, there are few studies on the role of histone acetylation in the regulation of ABCB1 in a hypoxic environment. HDAC5, as a class IIa HDAC, is a key transcriptional regulator enzyme that shuttles between the nucleus and cytoplasm with significant effects on protein expression. A previous study found that HDAC5 expression was significantly elevated in the liver tissue of plateau rats (Zhao et al., 2023). In summary, this study aimed to investigate the mechanism of epigenetic regulation of ABCB1 by HDAC5 through HDAC inhibitors in a BRL cell hypoxia model. We hypothesized that under a hypoxic environment, HDAC inhibitors activate ABCB1 transcription and enhance its function by increasing the level of H3K9ac in the ABCB1 promoter region by inhibiting the expression of HDAC5 in the hypoxia model.

## 2 Materials and methods

### 2.1 Cell culture

BRL (Buffalo Rat Liver) cells and HEK-293T (Servicebio, Wuhan, China) cells were grown in a dulbecco’s modified eagle medium [(+) 4.5 g/L D-glucose, (+) L-Glutamine, (+) Phenol Red, (+) 0.11 g/L sodium pyruvate; (−) HEPES] (BasalMedia, Shanghai, China) supplemented with 10% characterized fetal bovine serum (Animal Blood Ware, Shanghai, China) and 1% penicillin-streptomycin (Beijing Solarbio Science and Technology Co., Ltd., Beijing, China). Cells were maintained at 37°C in a humidified incubator with 5% CO_2_.

### 2.2 Establishment of hypoxia model in BRL cells

BRL cells were seeded in 96-well plates at a density of 4,000 cells/well and allowed to adhere overnight. The cells were then cultured in modular culture chambers (Brincubator, California, United States) under 1% O_2_ for 12, 24, 36, or 48 h, with a normoxic control group maintained under standard conditions. Cell viability was assessed using a CCK-8 assay kit (Jiancheng Bioengineering Institute, Nanjing, China).

### 2.3 Cell counting Kit-8 (CCK-8) assay

BRL cells were seeded in 96-well plates and allowed to adhere overnight. HDAC inhibitors, including SAHA, TSA, ROMI, Entinostat (MS-275), MC-1568 (Abmole, Houston, Texas, United States), and Bufexamac (Aladdin, Shanghai, China), were dissolved in DMSO and diluted to various concentrations using the complete medium. The cells were then treated with these inhibitors and subjected to hypoxia for 24 h, with a final DMSO concentration of 0.1%. Subsequently, 10 μL of CCK-8 solution and 90 μL of complete medium were added to each well. The plates were incubated for 40 min, and absorbance was measured at 450 nm using a SpectraMax® i3 microplate reader (Molecular Devices, San Jose, CA, USA). The assay relies on the principle that mitochondrial dehydrogenase in viable cells reduces the tetrazolium salt in CCK-8 into a water-soluble, orange-yellow formazan product, a reaction not performed by dead cells. The amount of formazan produced is directly proportional to the number of living cells, allowing absorbance measurements to reflect cell viability or proliferation.

### 2.4 Real-time quantitative polymerase chain reaction analysis (RT-qPCR)

Total cellular RNA was extracted by the Trizol method, and RNA purity and concentration were assessed by a NanoPhotometer® NP80 Touch Ultra-micro Spectrophotometer (Implen, Munich, Germany). The isolated RNA was subsequently reverse-transcribed to cDNA using PrimeScript RT Master Mix (Takara, Tokyo, Japan). We analyzed *ABCB1B*, HDAC5, SP1, and HIF-1α gene expression by RT-qPCR using the ViiA™7 real-time PCR instrument (Thermo Scientific, MA, USA) and SYBR Premix EX Taq (Takara, Tokyo, Japan). Ct values were converted to delta Ct values by comparing them to reference gene β-actin. Primers (Sangon Biotech, Shanghai, China) used in this study are listed in Table 1.

**TABLE 1:** The primers sequence of *ABCB1B*, HIF-1α, HDAC5, SP1 and β-actin.

Gene	Forward sequence	Reverse sequence
*ABCB1B*	TGAGGTCGTGATGGAGTTT	ATGCCAACAGCAGGTTTC
HIF-1α	GAACGTCGAAAAGAAAAGTCTC	CCTTATCAAGATGCGAACTCACA
HDAC5	CCGTGATGACTTTCCCCTCC	TGTGCCTGTGATCTCGACTG
SP1	CCAGACCATTAACCTCAGTGCA	ATGTATTCCATCACCGCCAG
β-actin	CACCCGCGAGTACAACCTTC	CCCATACCCACCATCACACC

### 2.5 Western blot Analysis

Cells were harvested at the specified time points, and total protein was extracted using RIPA lysis buffer supplemented with PMSF (Servicebio, Wuhan, China). Protein concentrations were determined with a BCA kit (Beijing Solarbio Science and Technology Co., Ltd., Beijing, China). Proteins were separated by SDS-PAGE, transferred to PVDF membranes (Millipore, Darmstadt, Germany), and blocked with 5% skim milk powder. Membranes were incubated overnight at 4°C with primary antibodies diluted in TBST (Servicebio, Wuhan, China): ABCB1 (1:1000; Proteintech, IL, USA), HDAC5 (1:1000; Abmart, Shanghai, China), H3K9ac (1:1000; Abcam, Cambridge, MA, USA, ab32129), HIF-1α (1:1000; Proteintech, IL, USA), SP1 (1:1000; Proteintech, IL, USA), and β-actin (1:3000; Proteintech, IL, USA). After washing with PBST, membranes were incubated with species-specific horseradish peroxidase-conjugated secondary antibodies (1:3000). Protein bands were visualized using the Bio-Rad ChemiDoc MP Chemiluminescent Gel Imaging System (Bio-Rad, CA, USA), and densitometric analysis was performed with ImageJ software, using β-actin as the internal control.

### 2.6 Rhodamine 123 accumulation assay

BRL cells were inoculated in 96-well plates and allowed to attach to the wall overnight. After adding SAHA, TSA, and Bufexamac for 24 h of anoxic incubation, the cells were incubated with or without P-gp inhibitor verapamil (Shanghai Yuanye Biotechnology Co., Ltd., Shanghai, China) for 1 h before adding rhodamine 123 (Shanghai Yuan Ye Biotechnology Co., Ltd., Shanghai, China) (5 μM, excitation light 485 nm, emission light 535 nm) was incubated at 37°C in a 5% CO_2_ incubator for 2 h. The reaction was terminated by rinsing the cells twice with pre-cooled HBSS (Servicebio, Wuhan, China), and then the cells were lysed by adding 100 µL of lysis buffer (1% Triton X-100) (Biotronik, Shanghai, China) for fluorescence assay using a SpectraMax® i3 enzyme-labeled instrument (Molecular Devices, San Jose, CA, USA).

### 2.7 Small interfering RNA transfection

The BRL cells were transfected according to the instructions of Lipofectamine™ 2000 (Thermo Scientific, MA, USA) for 4 h. After 4 h, the serum-containing culture medium was changed for anoxic culture, and gene expression was determined by Real-time PCR and Western blot Analysis after hypoxia for 24 h. The siRNA was designed and synthesized by Shanghai Sangon Biotech. The sequence is shown in Table 2.

**TABLE 2:** The sequence of siRNA.

siRNA	Sense (5′–3′)	Antisense (5′–3′)
siHDAC5-1	GAAACAAGGAGAAGAGCAATT	UUGCUCUUCUCCUUGUUUCTTT
siHDAC5-2	GCAACAGAGCACGCUCAUATT	UAUGAGCGUGCUCUGUUGCTT
siHDAC5-3	GGCAGAAGCUGGACAGUAATT	UUACUGUCCAGCUUCUGCCTT
siSP1-1	CGGCAAAGUAUAUGGCAAATT	UUUGCCAUAUACUUUGCCGTT
siSP1-2	CGGAUGAGCUUCAGAGACATT	UGUCUCUGAAGCUCAUCCGTT
siSP1-3	GAUCAUACCAGGUGCAAAUTT	AUUUGCACCUGGUAUGAUCTT

### 2.8 Cleavage under targets and tagmentation (CUT&Tag)

CUT&Tag assay was performed using the NovoNGS CUT&Tag 4.0 High-Sensitivity Kit (for Illumina) (Novoprotein, Suzhou, China) following the manufacturer’s protocol. Briefly, 100,000 cells were washed twice gently with Wash Buffer. Ten microliters Concanavalin A coated magnetic beads were added per sample and incubated at RT for 10 min. Remove the unbound supernatant and resuspend the bead-bound cells with the Primary Antibody Buffer and a 1:50 dilution of the H3K9ac antibody (Abcam, Cambridge, MA, USA) or the IgG control antibody (Cell Signaling Technology, Danvers, MA, USA), and incubate them overnight at 4°C. The primary antibody was removed using a magnet stand (Tiangen, Beijing, China). The secondary antibody (Goat anti-rabbit IgG H&L pAb, Hangzhou Jingjie Biotechnology, Hangzhou, China) was diluted 1:200 in Antibody Buffer and cells were incubated at RT for 60 min. Cells were washed using the magnet stand 2–3 times in Antibody Buffer. A 1:80 dilution of the pA-Tn5 adapter complex was prepared in ChiTag Buffer and incubated with cells at RT for 1 h. Cells were washed 3 × for 5 min in ChiTag Buffer. Then cells were resuspended in Tagmentation Buffer and incubated at 37°C for 1 h. DNA was purified using Tagment DNA Extract Beads. To amplify libraries, 20 μL DNA was mixed with 2.5 μL of a universal i5 and a uniquely barcoded i7 primer (Novoprotein, Suzhou, China). A volume of 25 μL 2 × HiFi AmpliMix (Novoprotein, Suzhou, China) was added and mixed. The sample was placed in a Thermocycler (Labnet International, Edison, NJ, USA) with a heated lid using the following cycling conditions: 72°C for 3 min; 98°C for 30 s; 13 cycles of 98°C for 15 s, 63°C for 8 s, 72°C for 5 s; final extension at 72°C for 2 min and hold at 10°C. Library clean-up was performed with NovoNGS DNA Clean Beads. Library quality was assessed on the Agilent 5400 system (Agilent, Santa Clara, CA, USA) and quantified by qPCR (1.5 nM). The qualified libraries were pooled and sequenced on Illumina platforms (San Diego, CA, USA) with PE150 strategy according to effective library concentration and data amount required. After obtaining high-resolution data, combine it with professional data analysis platforms to complete quality control, genome alignment, annotation of enrichment peaks, Motif identification, Gene Ontology (GO) functional analysis, and Kyoto Encyclopedia of Genes and Genomes (KEGG) pathway analysis. The experimental part was done by Hangzhou Jingjie Biotechnology Co., Ltd.

### 2.9 Coimmunoprecipitation (Co-IP)

BRL and HEK-293T cells were lysed with native lysis buffer (Servicebio, Wuhan, China) buffer and the supernatant was collected by centrifugation. Primary antibodies against SP1, HDAC5, or ABCB1 were incubated with the supernatant at 4°C overnight. The antigen-antibody complexes were then incubated with protein A/G magnetic beads for 6 h at 4°C and centrifuged to discard the supernatant, washed with lysis buffer, and then the beads were boiled for 5 min by adding 1 × Loading Buffer (Beijing Solarbio Science and Technology Co., Ltd., Beijing, China) for WB validation. Bio-Rad ChemiDoc MP Chemiluminescent Gel Imaging System (Bio-Rad, CA, USA) visualized the protein blots. The concentrations of the antibodies used were as follows: ABCB1 (1:300), HDAC5 (1:100), SP1 (1:500), Flag, Myc, and HA (Proteintech, IL, USA) (1:1000).

### 2.10 Immunofluorescence assays

HEK-293T cells were transfected with recombinant plasmids pcDNA3.1(+)-SP1-Flag, pcDNA3.1(+)-HDAC5-Myc, and pcDNA3.1(+)-ABCB1-HA (Nanjing Jingpu Sail Biotechnology, co, Ltd., Nanjing, China) for 24 h according to the transfection instructions of Lipofectamine™ 2000 and the empty vector pcDNA3.1(+)- was used as the negative control. The cells were washed twice with pre-cooled PBS (Beijing Solarbio Science and Technology Co., Ltd., Beijing, China), fixed with 4% paraformaldehyde (Servicebio, Wuhan, China) for 15–20 min, and washed 3 times with 1 × PBS for 5 min each time. 0.1% Triton was added for 25 min, and washed three times with 1 × PBS for 5 min each time. After 1% BSA (Beijing Solarbio Science and Technology Co., Ltd., Beijing, China) was blocked for 1 h, the cells were incubated overnight at 4°C with the labeled antibodies Flag, Myc, HA (Proteintech, IL, United States) (1:500), 1 × PBST washed three times for 10 min each time. Then incubate with fluorescent secondary antibody (Proteintech, IL, USA) (1:500) at 37°C for 1 h and wash three times with 1 × PBST for 10 min each time. Finally, add an anti-quenching reagent containing DAPI (Abcam, Cambridge, MA) to stain for 10 min and assay under an Olympus CKX53 Inverted Fluorescence Microscope (TRITC, excitation light 550 nm, emission light 570 nm, 40 × objective; FITC, excitation light 495 nm, emission light 525 nm, 40 × objective) (Olympus, Tokyo, Japan). Fluorescence co-localisation analysis was performed using ImageJ software (JACoP, co-localisation plugin).

### 2.11 Statistical analysis

GraphPad Prism 9.5 was used for statistical analysis. Differences between groups were assessed using a two-tailed Student’s t-test or one-way analysis of variance (ANOVA) followed by Dunnett’s *post hoc* test, depending on the number of comparisons and variables. A *p*-value <0.05 was considered statistically significant. All results are expressed as mean ± SD, and each experiment was performed with at least three independent replicates.

## 3 Results

### 3.1 The establishment of the BRL hypoxia model

**FIGURE 1:**
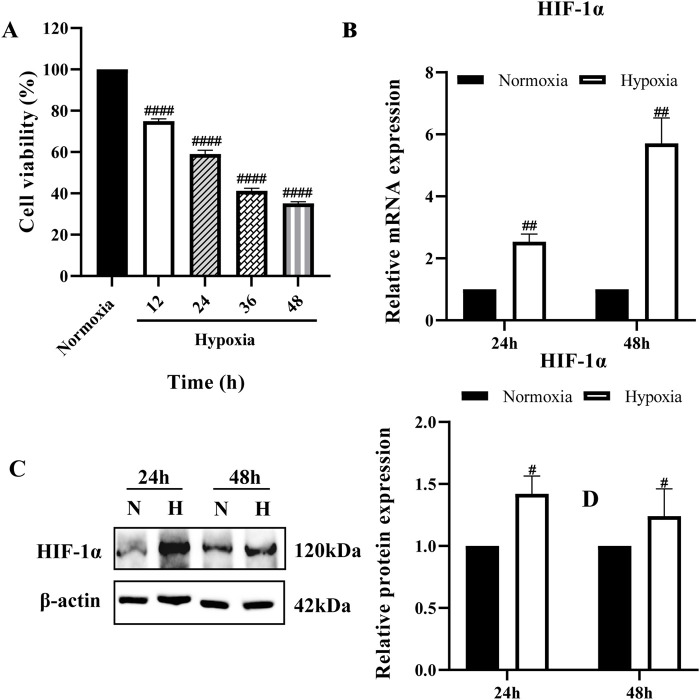
Establishment of hypoxia model **(A)** BRL cells were cultured at 1% O_2_ for different times and cell viability was determined by CCK-8; **(B,C)** HIF-1α mRNA and protein expression in BRL cells after hypoxia for 24 h and 48 h. **(A)**: one-way ANOVA, ^####^ *P* < 0.0001. **(B, C)**: two-tailed Student's t-test, ^#^ *P* < 0.05, ^##^ *P* < 0.01. ^#^: Normoxia vs Other groups. N, Normoxia; H, Hypoxia.

The cell viability of the hypoxia group was reduced compared with that of the normoxia group at all four-time points in a time-dependent manner (Figure 1A). The cell viability of the hypoxia group decreased by (25.0 ± 1.13) % at 12 h; (41 ± 1.86) % at 24 h; (58.8 ± 1.25) % at 36 h; and (64.8 ± 0.75) % at 48 h. These results indicate that 12 h of hypoxia (1% O_2_) is sufficient to induce cell damage. Based on these findings, 24 and 48 h were selected as key time points for further analysis. Next, we examined HIF-1α expression at these time points. The results showed a significant increase in HIF-1α at both the mRNA and protein levels, confirming the successful establishment of the hypoxia model (Figures 1B,C).

### 3.2 Viability of BRL cells in the hypoxia model after treatment with HDAC inhibitors

BRL cells in the hypoxia model were treated with increasing concentrations of HDAC inhibitors for 24 h, and cell viability was assessed using the CCK-8 assay (Supplementary Figure S1). Based on the results, concentrations that minimized cytotoxicity while effectively exerting drug activity were selected for further experiments. The final optimized concentrations of HDAC inhibitors were: 0.5 μM SAHA, 0.4 nM TSA, 1 μM Bufexamac, 1 nM ROMI, 0.5 μM MS-275, and 1.56 μM MC-1568.

### 3.3 Increased expression of ABCB1 transporter after HDAC inhibitor treatment

**FIGURE 2:**
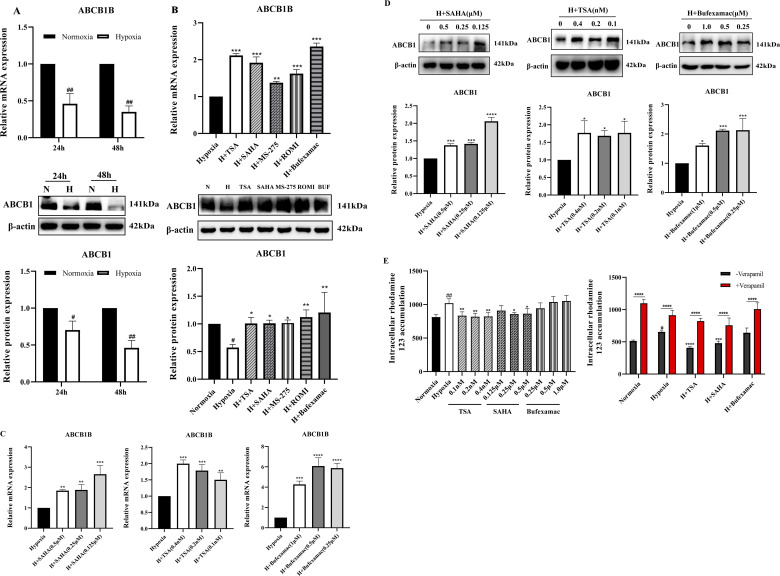
Increased expression of ABCB1 transporter after HDAC inhibitor treatment **(A)** BRL cells were hypoxia treated for 24 or 48 h and analyzed for ABCB1 mRNA and protein levels; **(B)** ABCB1 mRNA and protein levels were analyzed 24 h after treatment of the BRL hypoxia model with five HDAC inhibitors; **(C,D)** The BRL hypoxia model was treated with SAHA (0.125, 0.25, and 0.5 μM), TSA (0.1, 0.2, and 0.4 nM), and Bufexamac (0.25, 0.5, and 1 μM) for 24 h, and ABCB1 mRNA and protein levels were analyzed; **(E)** The BRL hypoxia model was treated with SAHA, TSA and Bufexamac for 24 h and then analyzed for intracellular concentrations of rhodamine 123. **(A)** two-tailed Student’s t-test, ^*#*^ *p* < 0.05, ^##^ *p* < 0.01; **(B–E)** one-way ANOVA, ^*#*^ *p* < 0.05, ^##^ *p* < 0.01, *p < 0.05, **p < 0.01, ***p < 0.001, ****p < 0.0001. ^**#**^Normoxia vs. Hypoxia; *Hypoxia vs. Other groups. N, Normoxia; H, Hypoxia; BUF, Bufexamac.

Suppression of ABCB1 transcription has been previously demonstrated in liver tissues of plateau rats (Duan et al., 2022). To confirm this result, we analyzed the expression of ABCB1 in the BRL cells in the hypoxia model. Consistent with previous studies, ABCB1 mRNA and protein expression were significantly decreased in hypoxic environments (Figure 2A). In tumors, histone deacetylation promotes the development and metastasis of various cancers by silencing a large number of chromosomal regions containing tumor suppressor genes, whereas HDAC inhibitors can affect the expression of cancer-associated genes, which in turn exerts an inhibitory effect in a wide range of malignancies (Li and Seto, 2016; Hassell, 2019; Mekala et al., 2022). Given the hypoxic nature of tumor cells, whether histone deacetylation is involved in the inhibition of ABCB1 expression in the BRL hypoxia model has not been confirmed. Therefore, we chose five different HDAC inhibitors to treat the BRL hypoxia model to analyze the expression of ABCB1 (Figure 2B). Among them, SAHA and TSA are class I and II inhibitors, MS-275 and ROMI are class I inhibitors, and Bufexamac is class IIb inhibitor. Study shows that all five HDAC inhibitors significantly upregulated the expression of both ABCB1 mRNA and protein. We selected three inhibitors with relatively stable effects: SAHA, TSA, and Bufexamac. Subsequently, treatment of the BRL hypoxia model with SAHA (0.125, 0.25, 0.5 μM), TSA (0.1, 0.2, 0.4 nM), and Bufexamac (0.25, 0.5, 1 μM), respectively, revealed that the lowest tested concentration of the HDAC inhibitor significantly increased the levels of ABCB1 mRNA and protein (Figures 2C,D). Meanwhile, corresponding to the above results, the BRL hypoxia model treated with SAHA and TSA exhibited a reduced accumulation of rhodamine 123, suggesting an enhanced exocytosis of ABCB1, which was reversed by co-incubation with verapamil, an ABCB1-specific inhibitor (Figure 2E). This suggests that the reduced retention of rhodamine 123 after 24 h exposure to SAHA and TSA is attributed to the upregulation of ABCB1 expression. Therefore, the transcriptional repression of ABCB1 in BRL cells under hypoxic conditions is closely linked to histone deacetylase activity.

### 3.4 HDAC5 inhibited ABCB1 expression by downregulating H3K9ac levels in the *ABCB1B* promoter region in the hypoxia model

**FIGURE 3:**
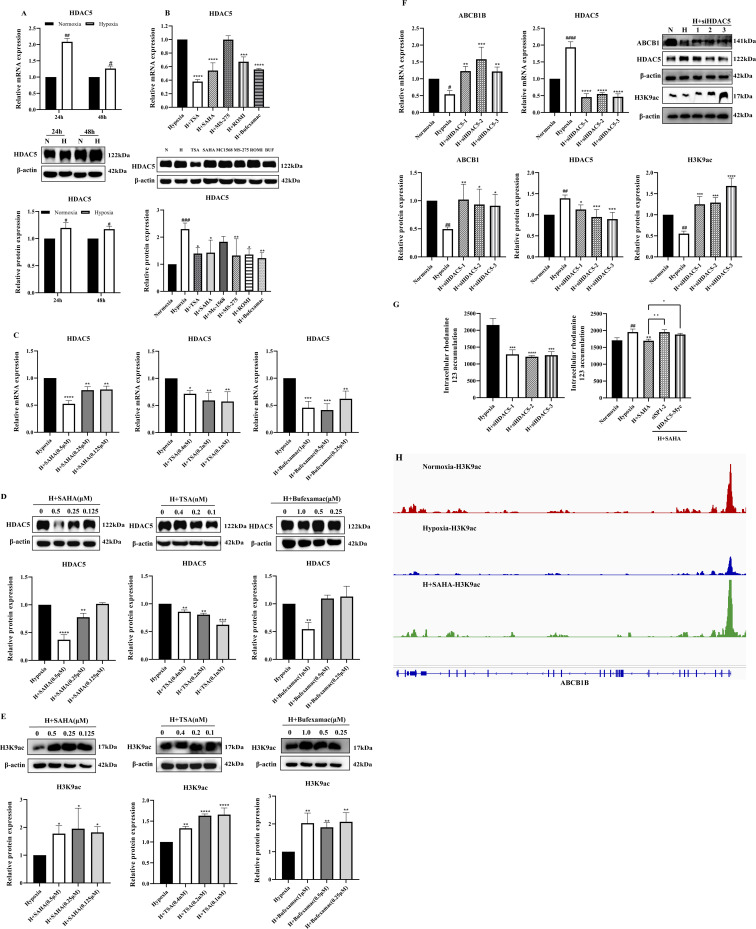
HDAC5 inhibited ABCB1 expression by downregulating H3K9ac levels in the *ABCB1B* promoter region in the hypoxia model **(A)** BRL cells were hypoxia treated for 24 or 48 h and analyzed for HDAC5 mRNA and protein levels; **(B)** HDAC5 mRNA and protein levels were analyzed 24 h after treatment of the BRL hypoxia model with HDAC inhibitors; **(C–E)** The BRL hypoxia model was treated with SAHA (0.125, 0.25, and 0.5 μM), TSA (0.1, 0.2, and 0.4 nM), and Bufexamac (0.25, 0.5, and 1 μM) for 24 h and analyzed for HDAC5 and H3K9ac expression; **(F)** BRL hypoxia model treated with siHDAC5 transfection and analyzed for HDAC5, ABCB1 and H3K9ac expression; **(G)** The BRL hypoxia model was treated with siHDAC5 transfection to analyze the intracellular concentration of rhodamine 123; the BRL hypoxia model was transfected with siSP1, pcDNA3.1-HDAC5-Myc, and then treated with SAHA for 24 h to analyze the intracellular concentration of rhodamine 123; **(H)** The BRL hypoxia model was treated with SAHA to analyze H3K9ac levels in the *ABCB1B* promoter region. **(A)** two-tailed Student’s t-test, ^*#*^ *p* < 0.05, ^##^ *p* < 0.01; **(B–G)** one-way ANOVA, ^*#*^ *p* < 0.05, ^*##*^ *p* < 0.01, ^*####*^ *p* < 0.0001, *p < 0.05, **p < 0.01, ***p < 0.001, ****p < 0.0001. ^**#**^Normoxia vs. Hypoxia; *Hypoxia vs. other groups. N, Normoxia; H, Hypoxia; BUF, Bufexamac.

A study showed that the expression of HDAC5 was significantly elevated in liver tissues of plateau rats, suggesting that alterations in its activity and function may be involved in regulating the inhibition of ABCB1 expression in the low oxygen environment of the plateau (Zhao et al., 2023). To determine the role of HDAC5 in the regulatory mechanism of ABCB1 under hypoxia, we examined the mRNA and protein expression of HDAC5 in the hypoxia model. Consistent with published results, HDAC5 expression was significantly elevated (Figure 3A). Treatment of the BRL hypoxia model with six HDAC inhibitors revealed that four compounds (SAHA, TSA, Bufexamac, and ROMI) significantly downregulated HDAC5 mRNA and protein expression. The inhibitory effect of these inhibitors on HDAC5 is closely correlated with their ability to activate ABCB1 (Figure 3B). Among them, SAHA, TSA, and Bufexamac exhibited the strongest inhibition of HDAC5, with SAHA showing a concentration-dependent effect, making it the candidate for subsequent studies (Figures 3C,D). Previous studies have indicated that H3K9ac, a histone acetylation activation marker, is closely associated with HDAC5 (Miao et al., 2024; Klymenko et al., 2020). In this study, even the lowest concentration of HDAC inhibitors significantly upregulated H3K9ac protein levels (Figure 3E). To further investigate the regulatory role of HDAC5 on ABCB1, siRNA-mediated knockdown of HDAC5 was performed in the BRL hypoxia model. After the transient knockdown of HDAC5, both ABCB1 and H3K9ac expression levels were significantly increased (Figure 3F). Additionally, fluorescent dye accumulation experiments demonstrated that HDAC5 knockdown reduced rhodamine 123 accumulation, while HDAC5 overexpression reversed the SAHA-induced reduction in rhodamine 123 accumulation (Figure 3G). These findings suggest that ABCB1 transcriptional repression in hypoxic environments may be linked to HDAC5-mediated reduction in acetylation levels. To verify this, we conducted a CUT&Tag assay to examine H3K9ac levels in the *ABCB1B* promoter region in the BRL hypoxia model. Compared to the normoxia group, H3K9ac levels were significantly lower in the hypoxia group, but SAHA treatment restored H3K9ac levels at the *ABCB1B* promoter (Figure 3H). Therefore, HDAC5 upregulation in hypoxia reduces H3K9ac levels in the *ABCB1B* promoter region, leading to ABCB1 transcriptional repression.

### 3.5 SP1 was also involved in the regulation of ABCB1 by HDAC5 in the BRL hypoxia model

**FIGURE 4:**
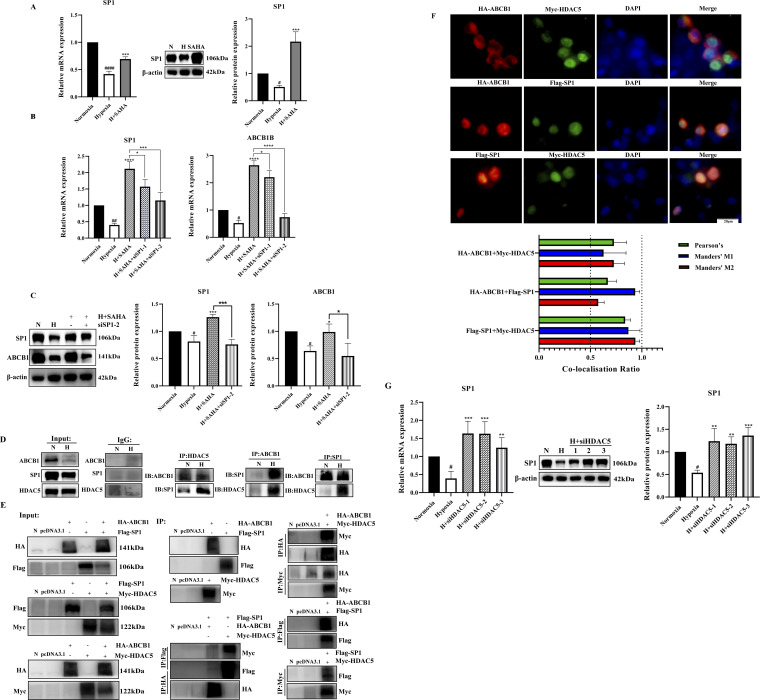
SP1 was also involved in the regulation of ABCB1 by HDAC5 in the BRL hypoxia model **(A)** BRL hypoxia model treated with SAHA for 24 h and analyzed for SP1 mRNA and protein expression; **(B,C)** The BRL hypoxia model was transfected with siSP1, then treated with SAHA for 24 h and analyzed for SP1, ABCB1 mRNA and protein expression; **(D)** Demonstration of two-by-two interactions between HDAC5, SP1, and ABCB1 in the BRL hypoxia model by Co-IP experiments. **(E)** After transfection of HEK-293T cells with the overexpression plasmid, the two-by-two interaction between HDAC5, SP1, and ABCB1 was demonstrated by the Co-IP assay. **(F)** Co-localization of HDAC5 with SP1, ABCB1, and ABCB1 with SP1 by immunofluorescence assay after transfection of HEK-293T cells with overexpression plasmids. The Pearson’s coefficient and Manders' overlap coefficients are displayed in the form of bar graphs; **(G)** The BRL hypoxia model was analyzed for SP1 expression after treatment with siHDAC5 transfection. **(A–C, G)** one-way ANOVA, ^#^ *p* < 0.05, ^##^ *p* < 0.01, ^####^ *p* < 0.0001, *p < 0.05, **p < 0.01, ***p < 0.001, ****p < 0.0001. ^**#**^Normoxia vs. Hypoxia; *Hypoxia vs. other groups. N, Normoxia; H, Hypoxia.

**FIGURE 5:**
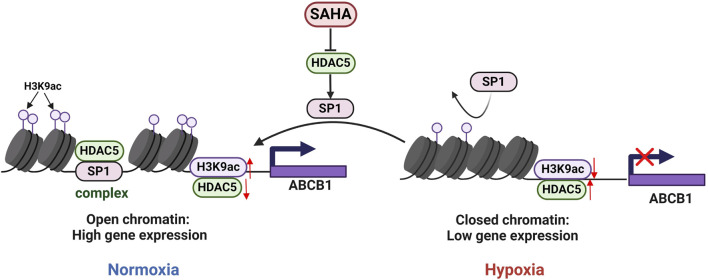
Regulatory mechanisms of ABCB1 expression inhibition by HDAC5 in the BRL hypoxia model. In BRL cells, HDAC5 may be recruited by SP1 to form a complex, reducing free HDAC5, increasing H3K9ac at the ABCB1 promoter, and activating ABCB1 transcription. In the BRL hypoxia model, disruption of the SP1-HDAC5 complex increased free HDAC5, lowered H3K9ac at the ABCB1 promoter, and suppressed ABCB1 transcription.

Published studies indicate that under normal conditions, class IIa HDAC is non-phosphorylated, resides in the nucleus, and is recruited to target genes to exert transcriptional repression via transcription factors such as SP1, MYC, and YY1. Phosphorylation disrupts these interactions, resulting in HDAC export to the cytoplasm and reduced transcriptional repression (Di Giorgio and Brancolini, 2016; Wang et al., 2021; Bi and Jiang, 2006). We investigated transcription factors regulating ABCB1 in BRL cells through literature review and database binding predictions for the ABCB1 promoter. SP1 was found to bind multiple sites in ABCB1. Our study revealed downregulated SP1 expression in the BRL hypoxia model, while SAHA treatment significantly induced SP1 expression (Figure 4A). To determine whether SP1 inhibition is critical for ABCB1 downregulation in hypoxia, we used siSP1 with SAHA to assess ABCB1 expression. Knockdown of SP1 blocked SAHA-induced ABCB1 transcriptional activation at both mRNA and protein levels (Figures 4B,C), correlating with increased intracellular rhodamine 123 accumulation, suggesting SP1 regulates ABCB1 transcription in the BRL hypoxia model (Figure 3G). Co-IP experiments confirmed interactions between ABCB1, SP1, and HDAC5 in BRL cells. Immunoprecipitation assays showed endogenous and exogenous interactions among these proteins (Figures 4D,E). Co-localization studies in HEK-293T cells further confirmed their overlapping distribution (Figure 4F). These findings suggest that changes in H3K9ac levels at the ABCB1 promoter in BRL cells may occur via SP1 recruitment of HDAC5. Since SAHA downregulates HDAC5 to induce ABCB1 expression, we examined whether SAHA regulates SP1 through HDAC5. HDAC5 knockdown significantly upregulated SP1, indicating that HDAC5 represses SP1 transcription via histone acetylation modification in the BRL hypoxia model (Figure 4G). Thus, we propose the hypothesis that hypoxia-induced HDAC5 elevation downregulates SP1, reducing its recruitment of HDAC5, increasing free HDAC5, and repressing ABCB1 transcription (Figure 5).

## 4 Discussion

Molecularly targeted therapies against drug transporter proteins like ABCB1 are increasingly effective in clinical tumor treatment (Assaraf et al., 2019). HDAC inhibitors play a vital role in oncogene-targeted therapies due to their widespread impact on gene expression (Bose et al., 2014; Bondarev et al., 2021). Notably, the hypoxic environment at high altitudes shares similarities with tumor drug resistance: both can alter the expression and function of drug transporters, including ABCB1, thereby influencing drug metabolism and ultimately therapeutic efficacy. Building on this, our study explored the regulatory role of histone acetylation on ABCB1 using HDAC inhibitors. Experimental results revealed that all five tested HDAC inhibitors significantly modulated the transcriptional levels of ABCB1, although their effects varied considerably. Specifically, both SAHA and TSA significantly upregulated ABCB1 expression at both the mRNA and protein levels. In contrast, although bufexamac induced ABCB1 expression, it failed to enhance its functional activity, as evidenced by the lack of change in rhodamine 123 accumulation in the assay. TSA demonstrated superior efficacy compared to SAHA, but SAHA’s clinical approval, longer half-life, lower toxicity, and greater stability led us to select SAHA for further research(Weichert, 2009). These findings suggest that the expression of ABCB1 is regulated by histone acetylation, and different HDAC inhibitors may produce differential regulatory effects through specific targets or signaling pathways.

Histone acetylation is dynamically regulated by HATs and HDACs (de Ruijter et al., 2003). This study found that the expression of HDAC5 in the hypoxic model was significantly increased, which was negatively correlated with the level of histone activation marker H3K9ac. Using the CUT&Tag technique, we further confirmed that HDAC5 upregulation in the BRL hypoxia model decreased H3K9ac levels in the ABCB1 promoter, inhibiting its expression. SP1, a member of the SP1/Krüppel-like family of transcription factors, binds core promoter elements like GC-BOX and dynamically recruits HAT and HDAC to modulate histone acetylation, thereby regulating gene transcription (Lee et al., 2005; Li et al., 2019). In our experiments, SAHA treatment and HDAC5 knockdown in the hypoxia model significantly induced SP1 expression, while SP1 knockdown reduced SAHA’s induction of ABCB1. Immunoprecipitation and immunofluorescence co-localization confirmed SP1, HDAC5, and ABCB1 interactions. We propose the hypothesis SP1 recruits HDAC5 to the ABCB1 promoter, reducing free HDAC5, increasing H3K9ac levels, and activating ABCB1 transcription. Hypoxia disrupts the SP1-HDAC5 complex, increasing free HDAC5, reducing H3K9ac levels, and inhibiting ABCB1 transcription.

In summary, this study suggests that hypoxia may dynamically regulate ABCB1 expression through the SP1-HDAC5 axis and demonstrates that HDAC inhibitors can enhance ABCB1 expression and functional activity by suppressing HDAC. This finding provides a potential therapeutic target to address pharmacokinetic abnormalities in high-altitude hypoxic environments and expands potential clinical applications of HDAC inhibitors. However, several critical questions remain unresolved and require further investigation: (1) Whether the SP1-HDAC5 complex can dynamically regulate ABCB1 expression; (2) The molecular mechanism by which HDAC5 downregulates SP1 expression under hypoxic environments. Furthermore, studies have demonstrated a close interplay between histone acetylation and DNA methylation. These two epigenetic modifications collaboratively regulate chromatin stability to determine gene transcription levels (Li et al., 2021). Therefore, we hypothesize that histone acetylation and DNA methylation may synergistically regulate ABCB1 expression. Future research should integrate multi-omics approaches, including epigenomics and transcriptomics, to systematically elucidate the mechanisms of epigenetic regulation under hypoxic environments, thereby providing a theoretical foundation for rational drug use in high-altitude regions.

## Data Availability

The original contributions presented in the study are included in the article/Supplementary Material, further inquiries can be directed to the corresponding author.
