# Supplementary material for: Regulatory mechanism of ABCB1 transcriptional repression by HDAC5 in rat hepatocytes under hypoxic environment
Source: Front Physiol. 2025 Apr 8;16:1520246. doi: 10.3389/fphys.2025.1520246 (PMC12011715; doi:10.3389/fphys.2025.1520246)
Supplement: Supplementary file 1 [file DataSheet13.pdf]

Normoxia vs Hypoxia -P-gp

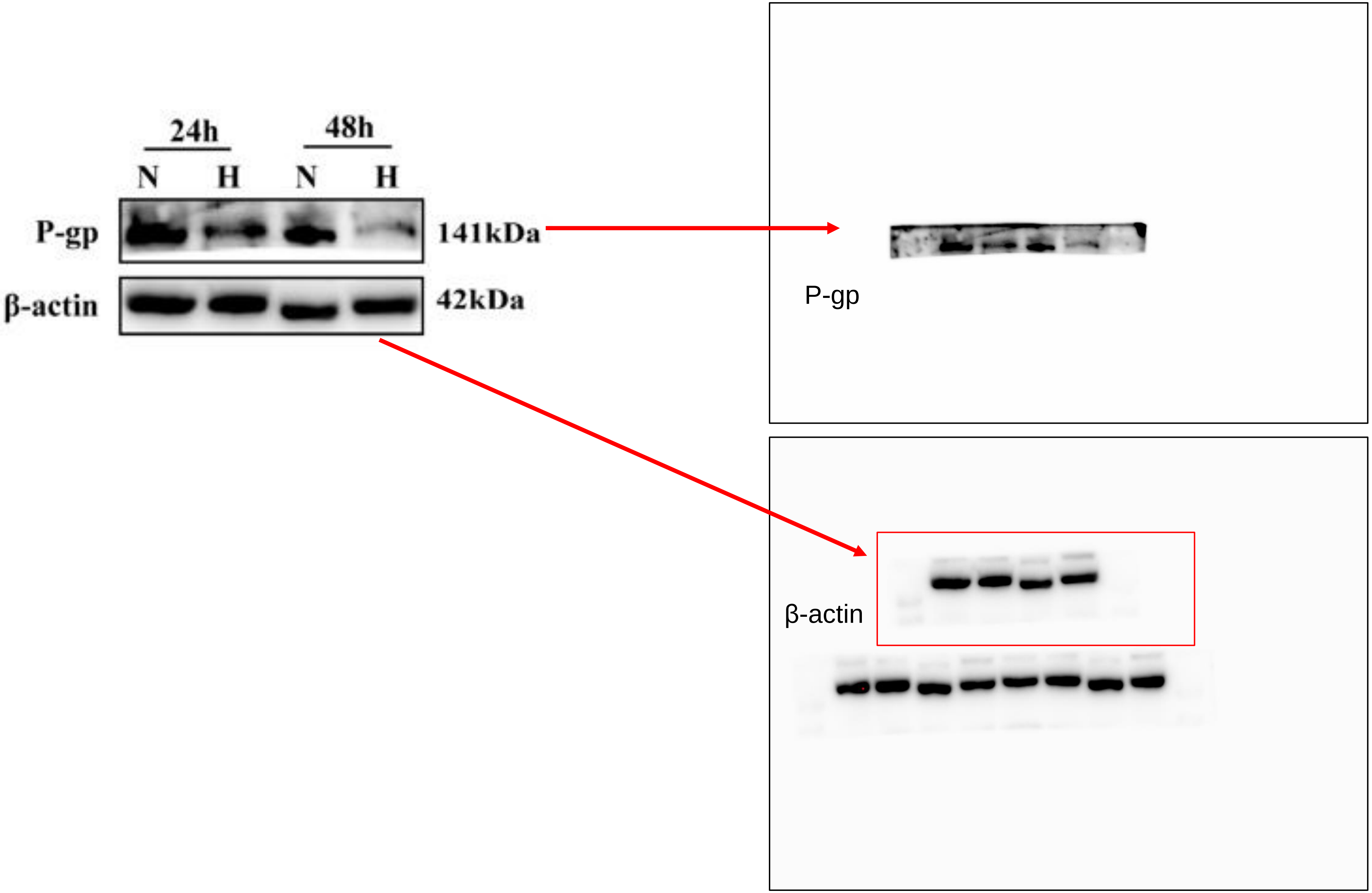

Normoxia vs Hypoxia –HDAC5

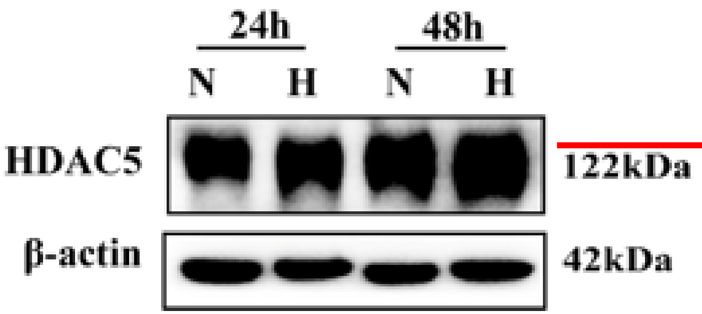

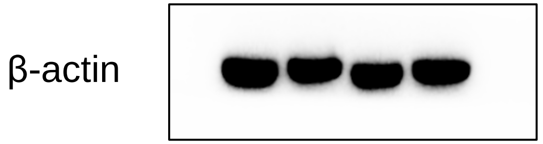

I'm very sorry, but beta-actin was distorted during cropping.

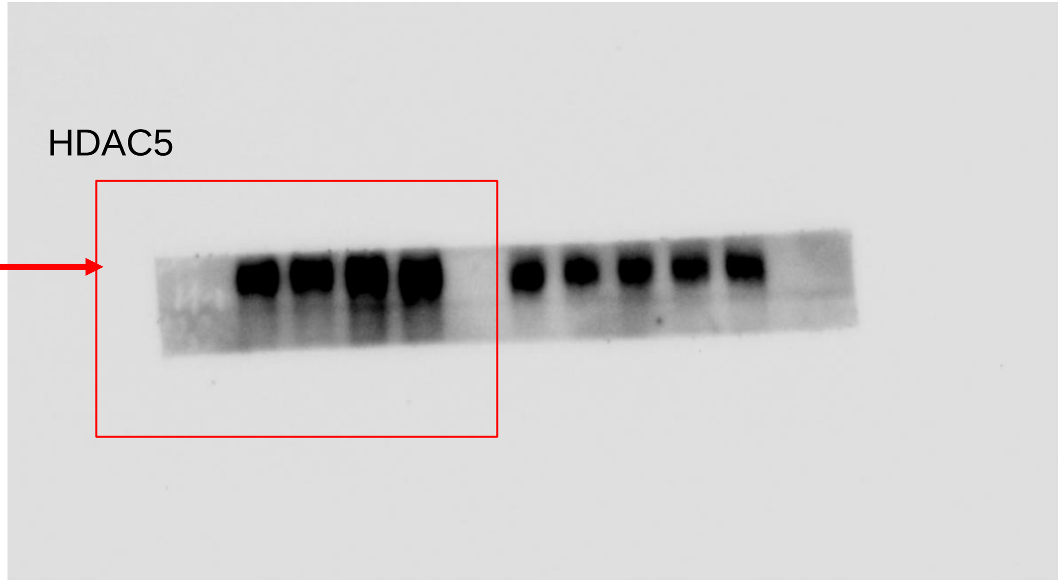

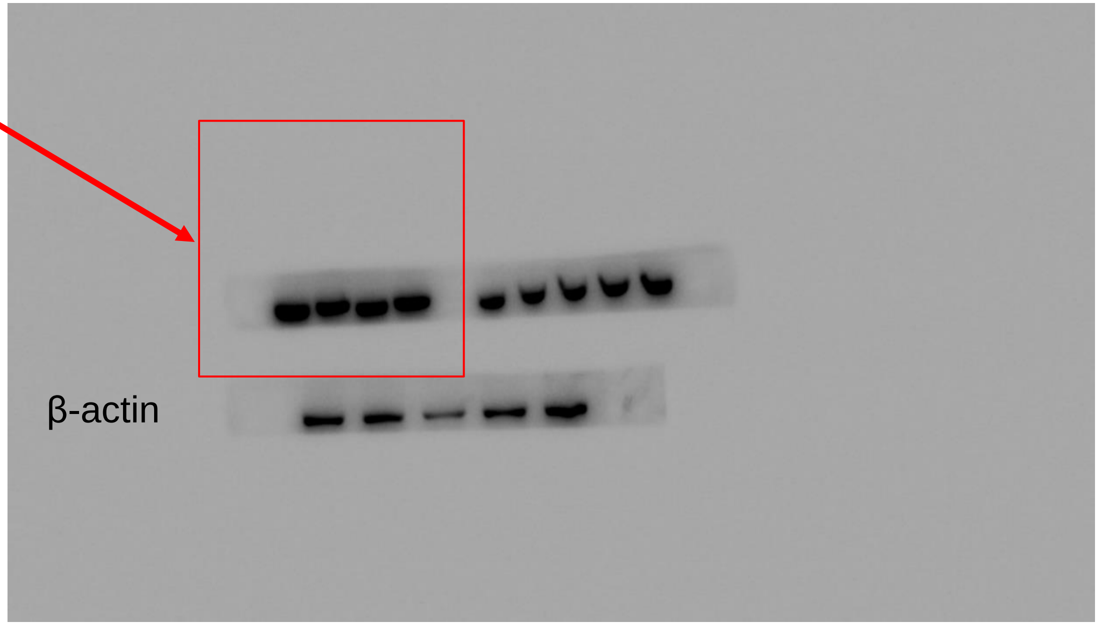

Normoxia vs Hypoxia –HIF-1

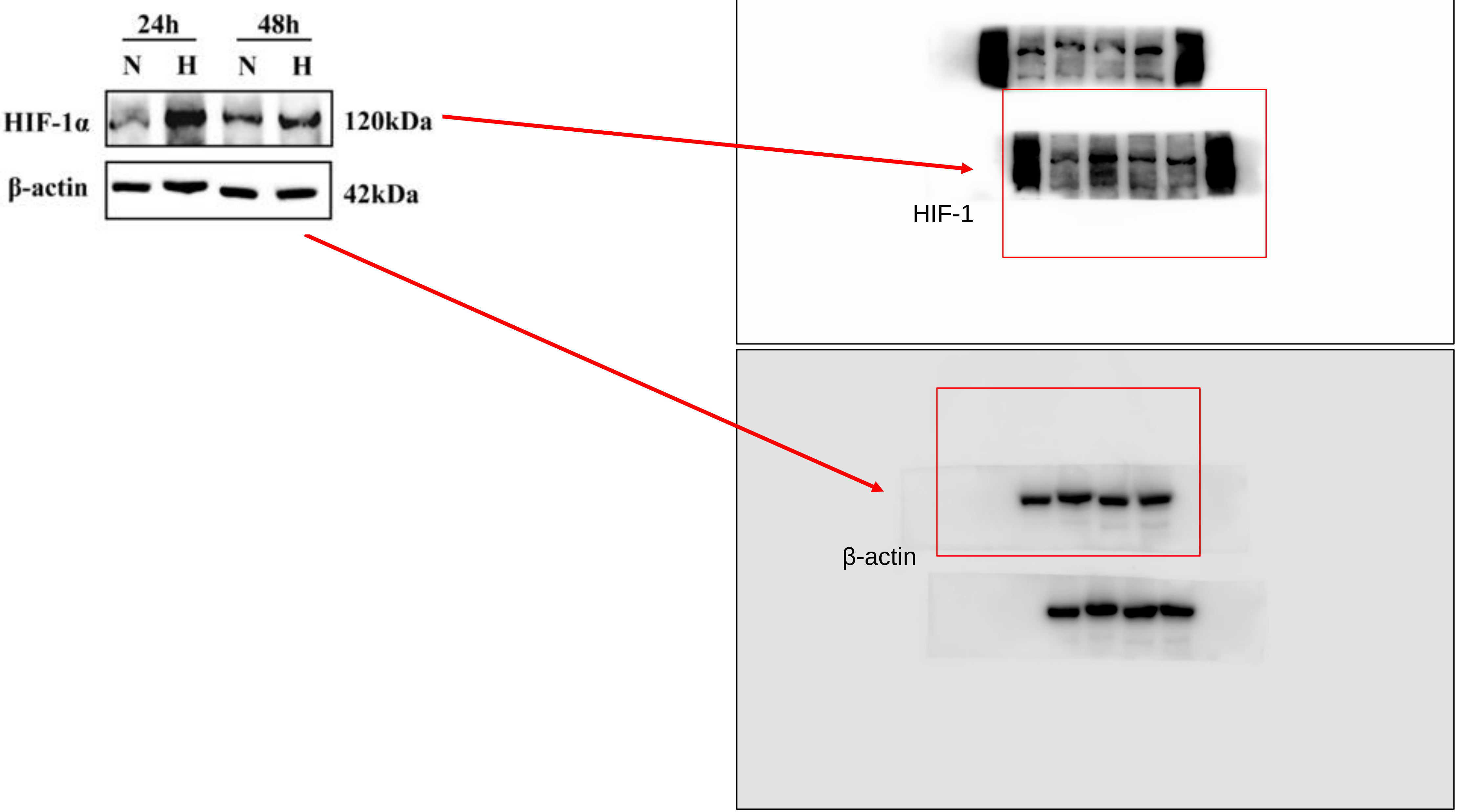

siHDAC5-P-gp,HDAC5

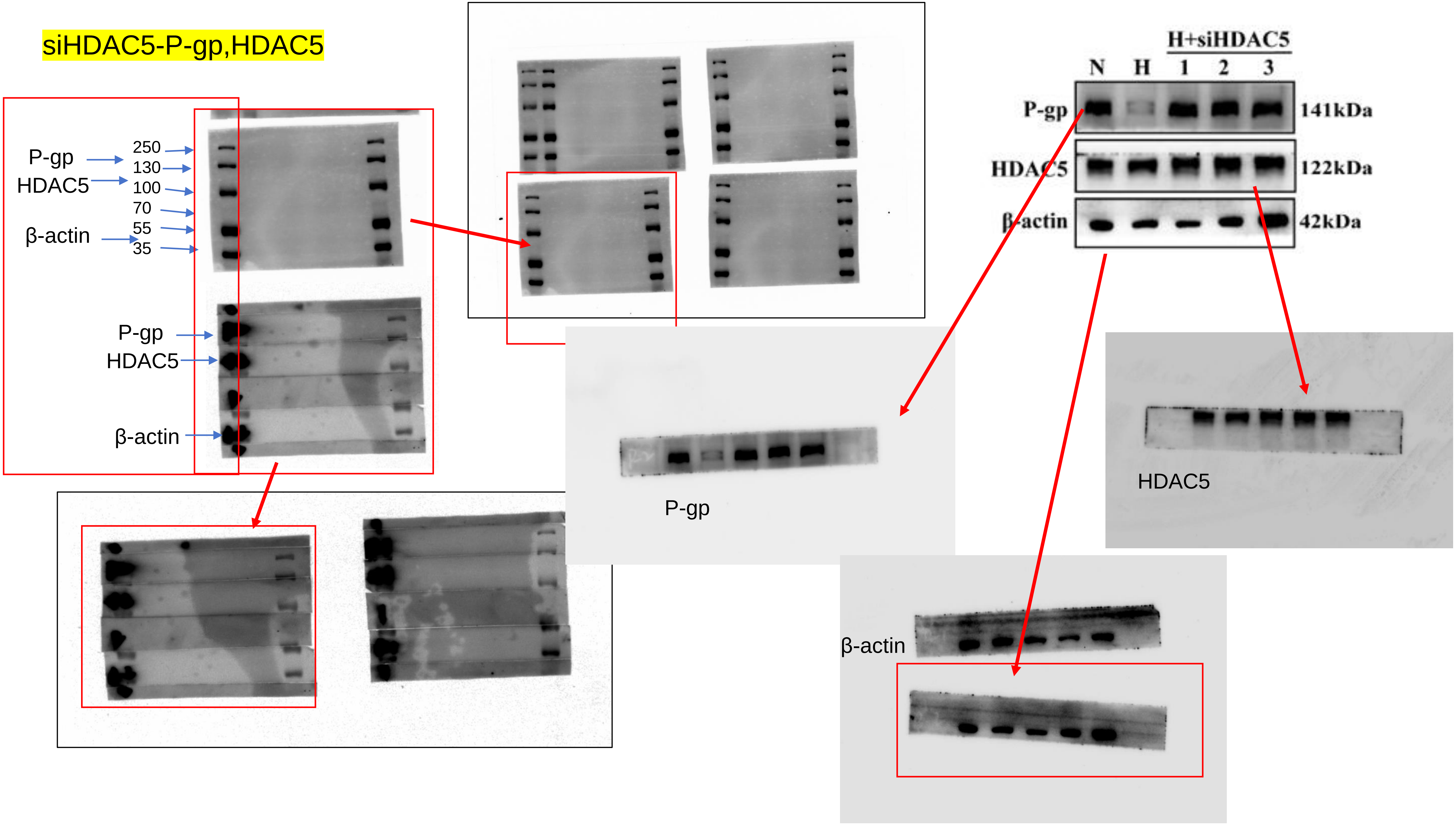

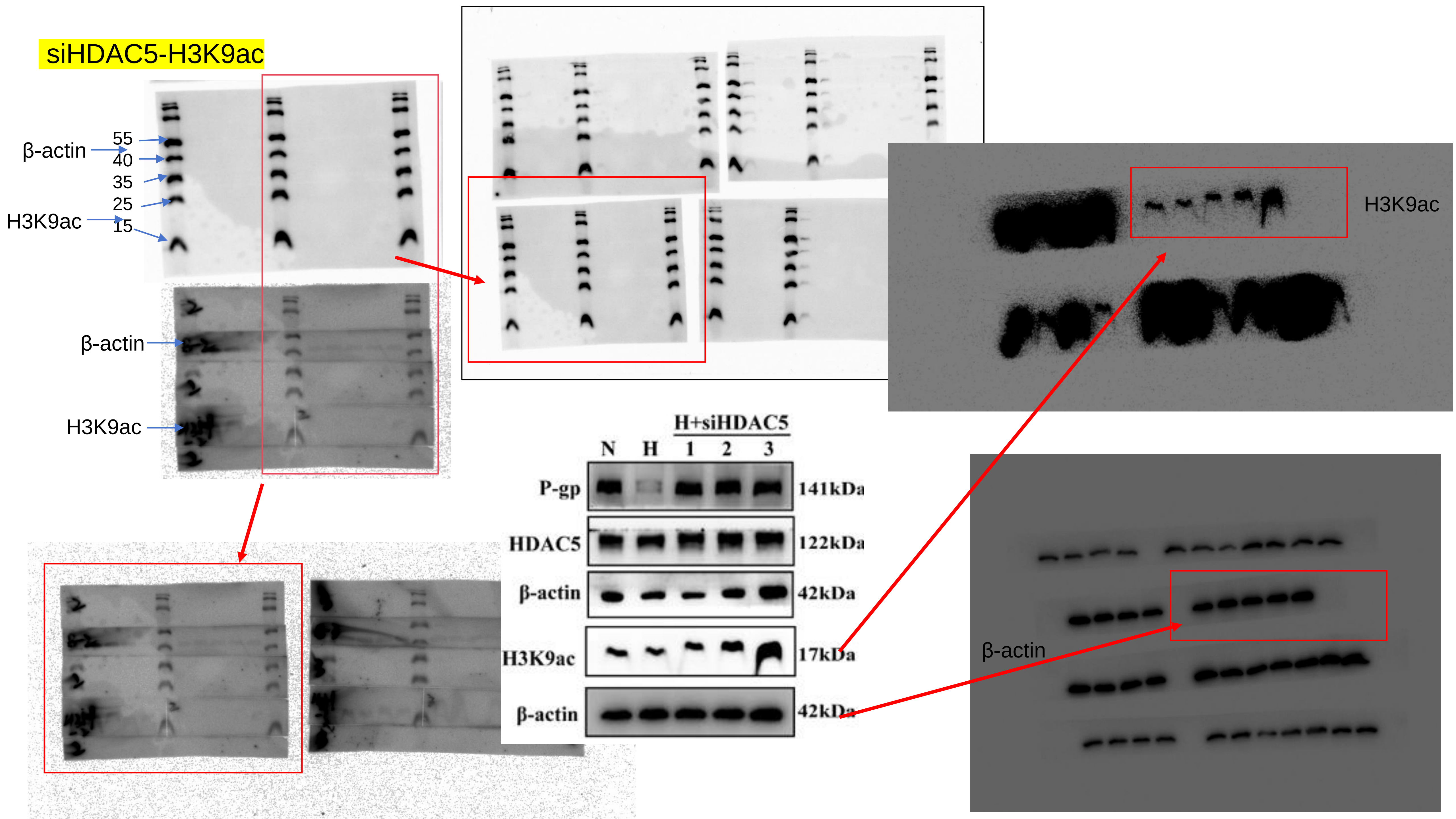

siHDAC5-H3K9ac
β-actin
55
40
35
25
15
H3K9ac
β-actin
H3K9ac
H+siHDAC5
N
H
1
2
3
P-gp
141kDa
HDAC5
122kDa
β-actin
42kDa
H3K9ac
17kDa
β-actin
42kDa
H3K9ac
β-actin

siHDAC5-SP1

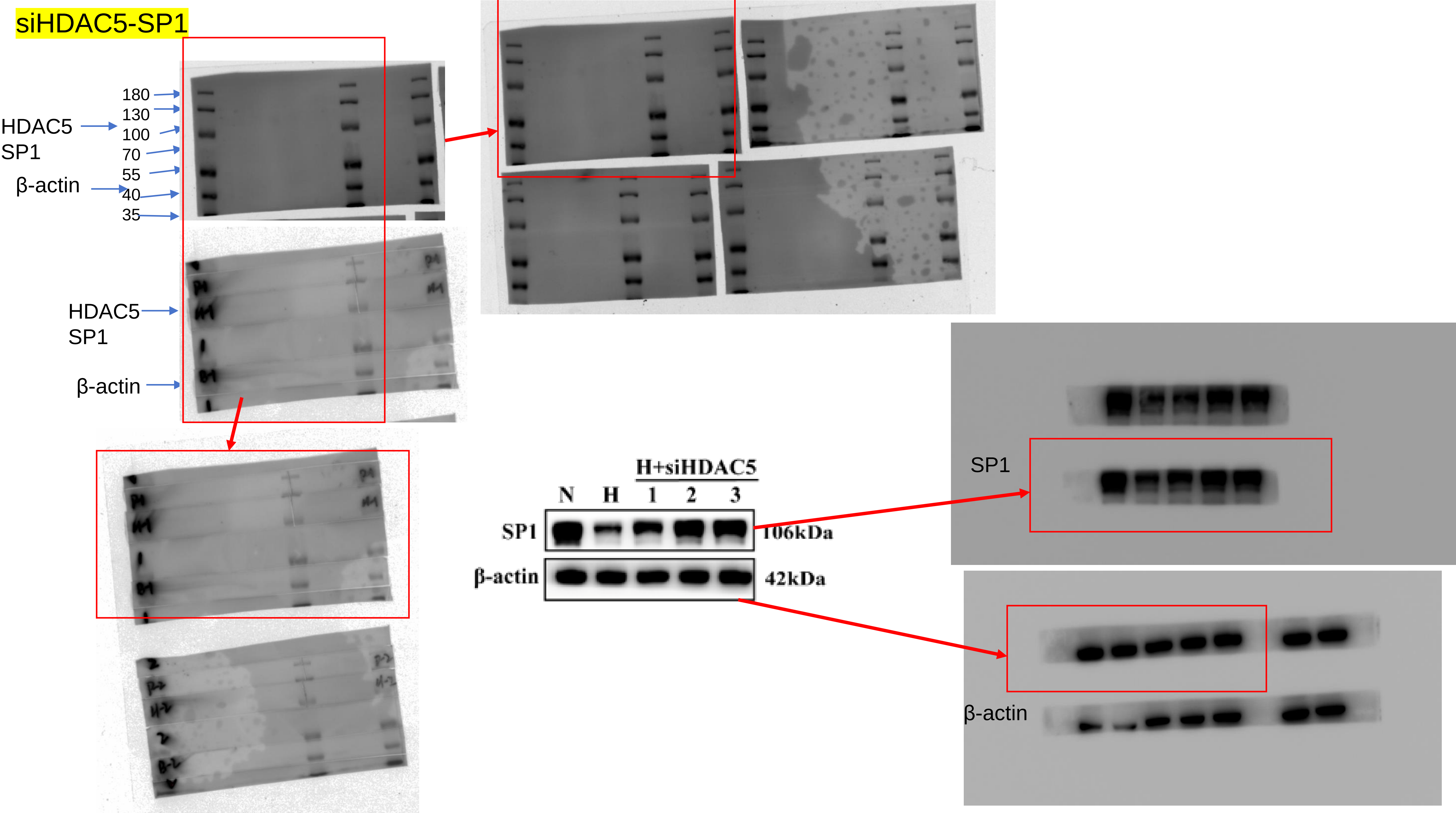
180
130
100
70
55
40
35
HDAC5
SP1
β-actin
HDAC5
SP1
β-actin
H+siHDAC5
N
H
1
2
3
SP1
106kDa
β-actin
42kDa
SP1
β-actin

SAHA (0,0.5,0.25,0.125μM)- and Bufexamac (0,1,0.5,0.25μM)-H3K9ac

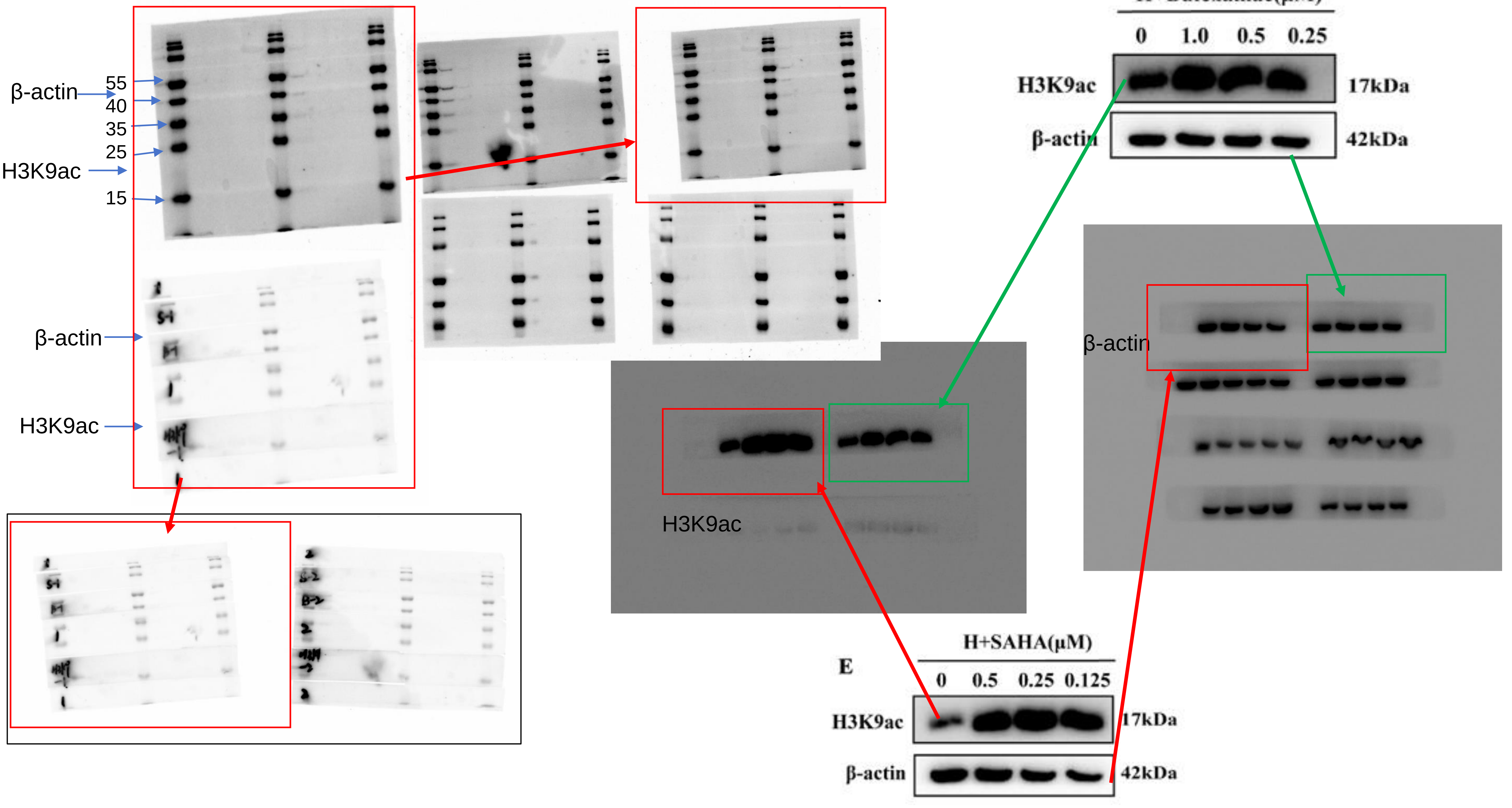

TSA (0,0.4,0.2,0.1nM)-H3K9ac

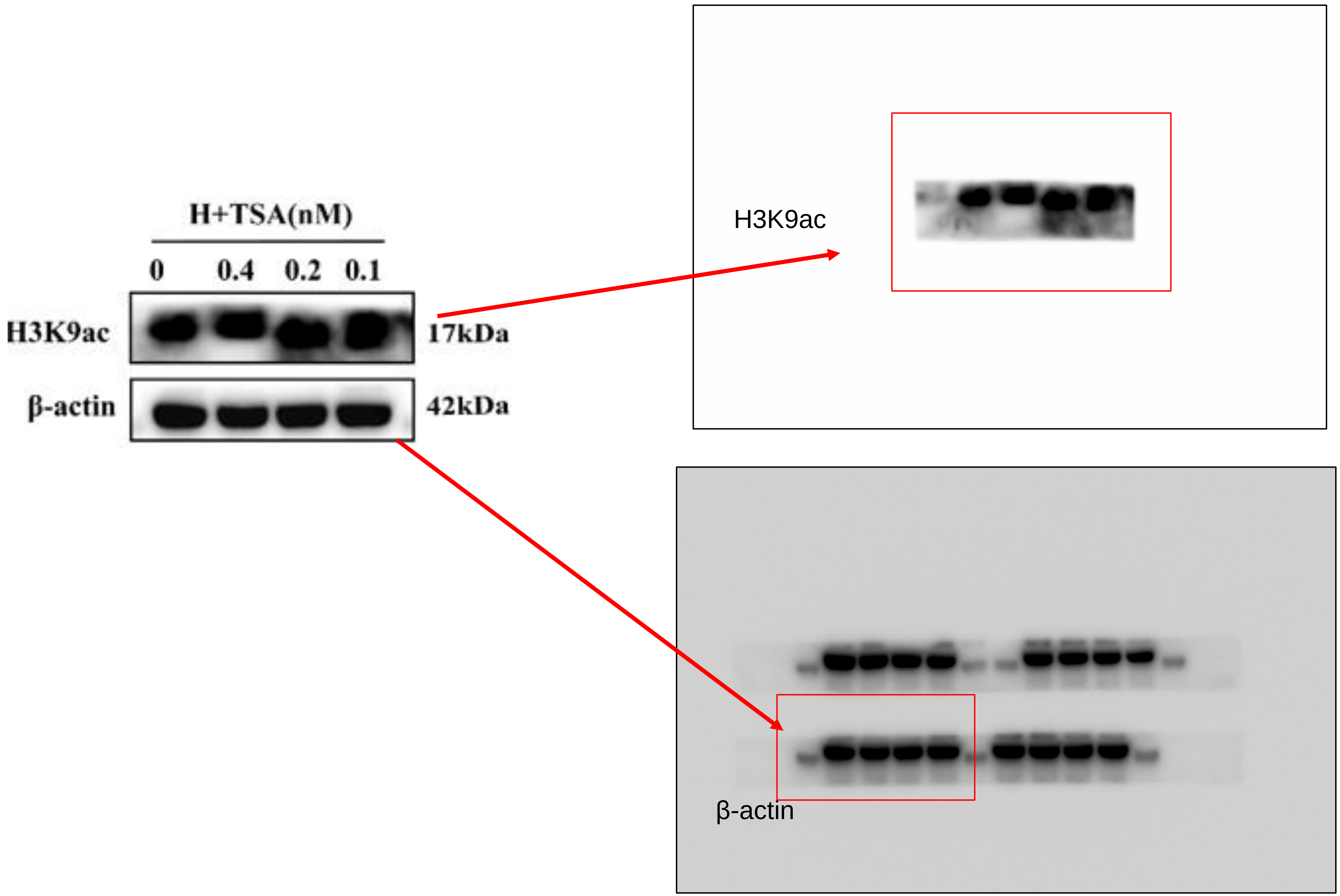

Bufexamac (0,1,0.5,0.25μM)-P-gp

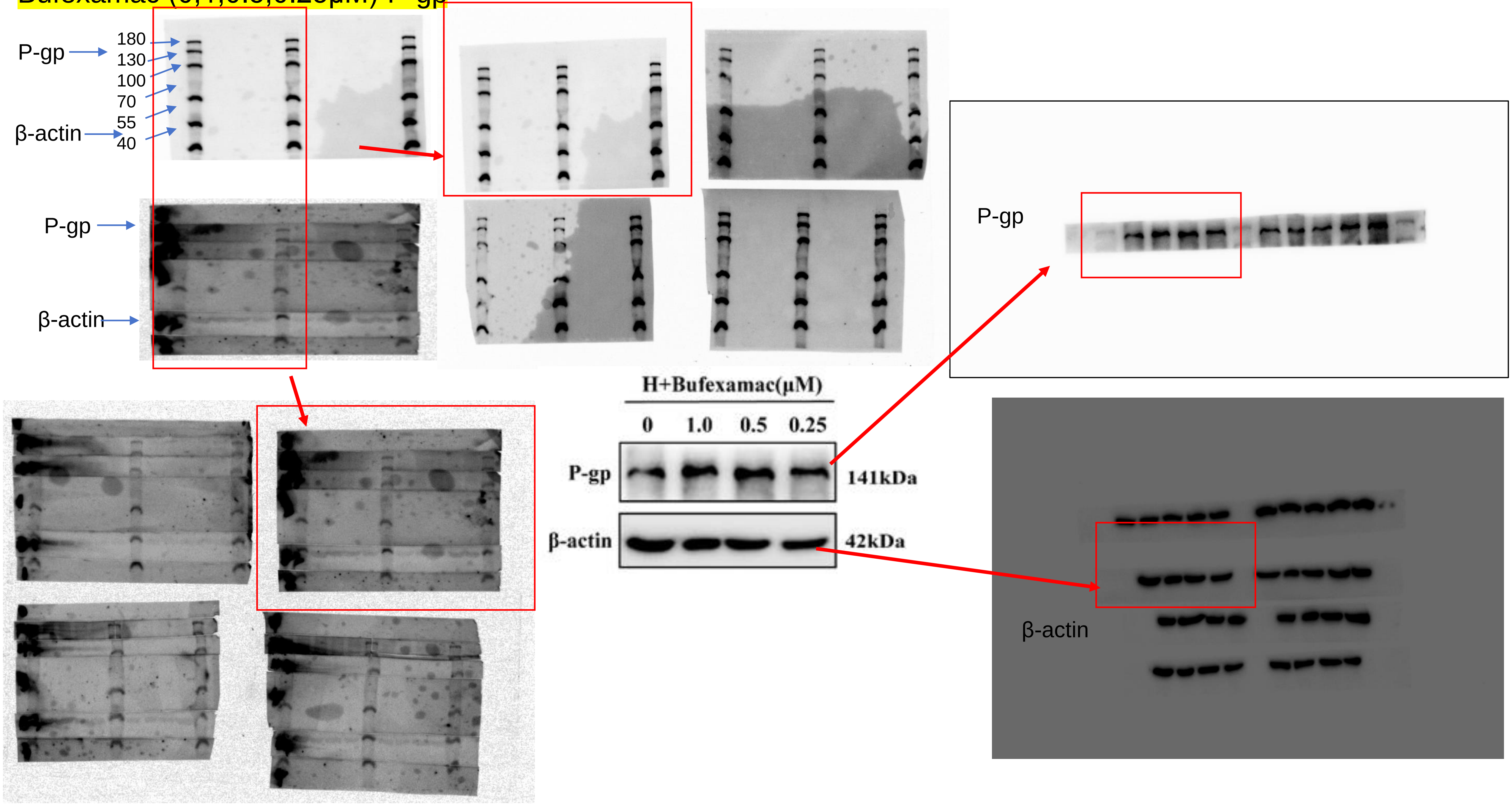

SAHA (0,0.5,0.25,0.125μM) and TSA (0,0.4,0.2,0.1nM)- P-gp

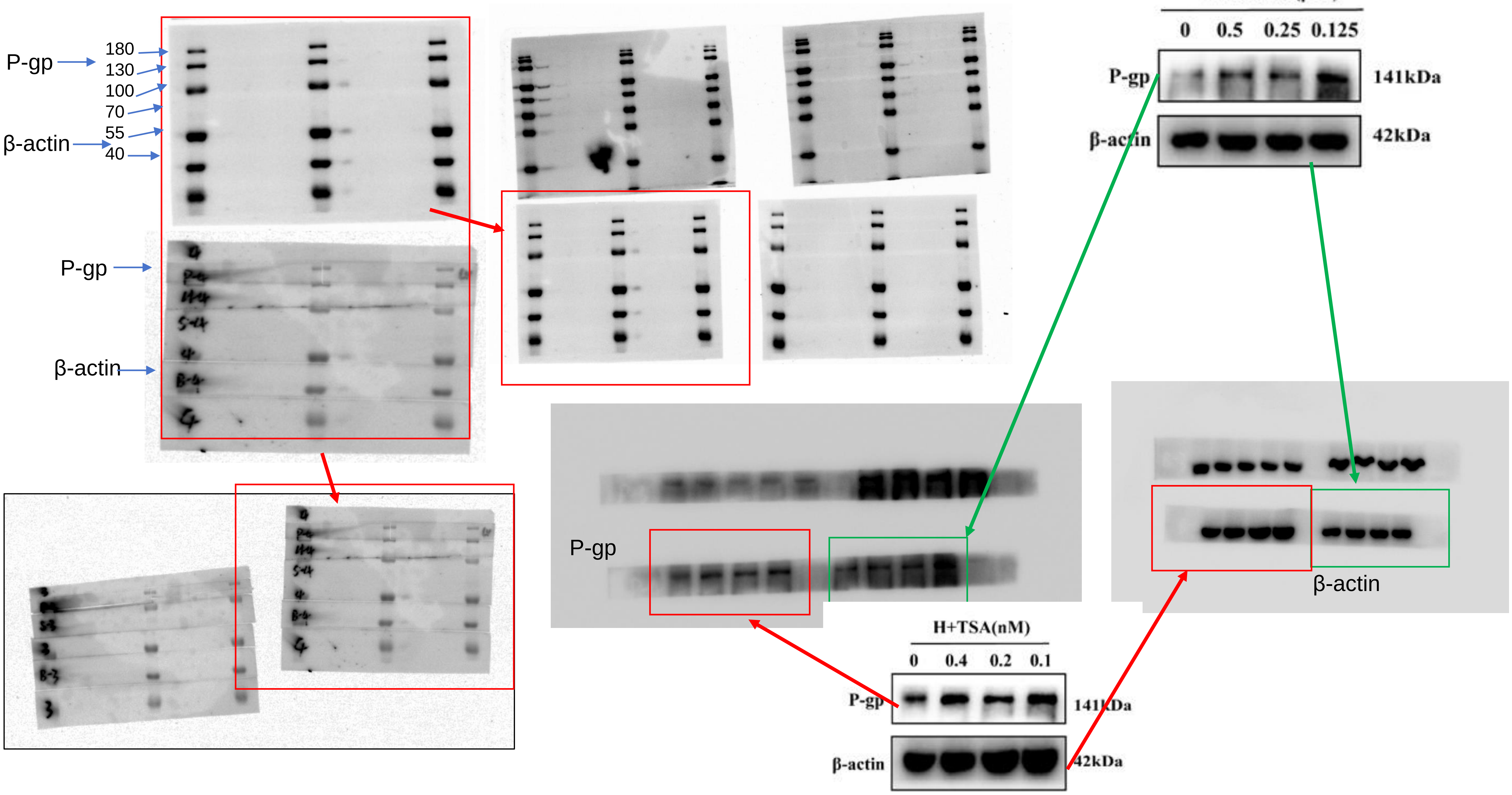

SAHA (0,0.5,0.25,0.125μM) and Bufexamac (0,1,0.5,0.25μM)-HDAC5

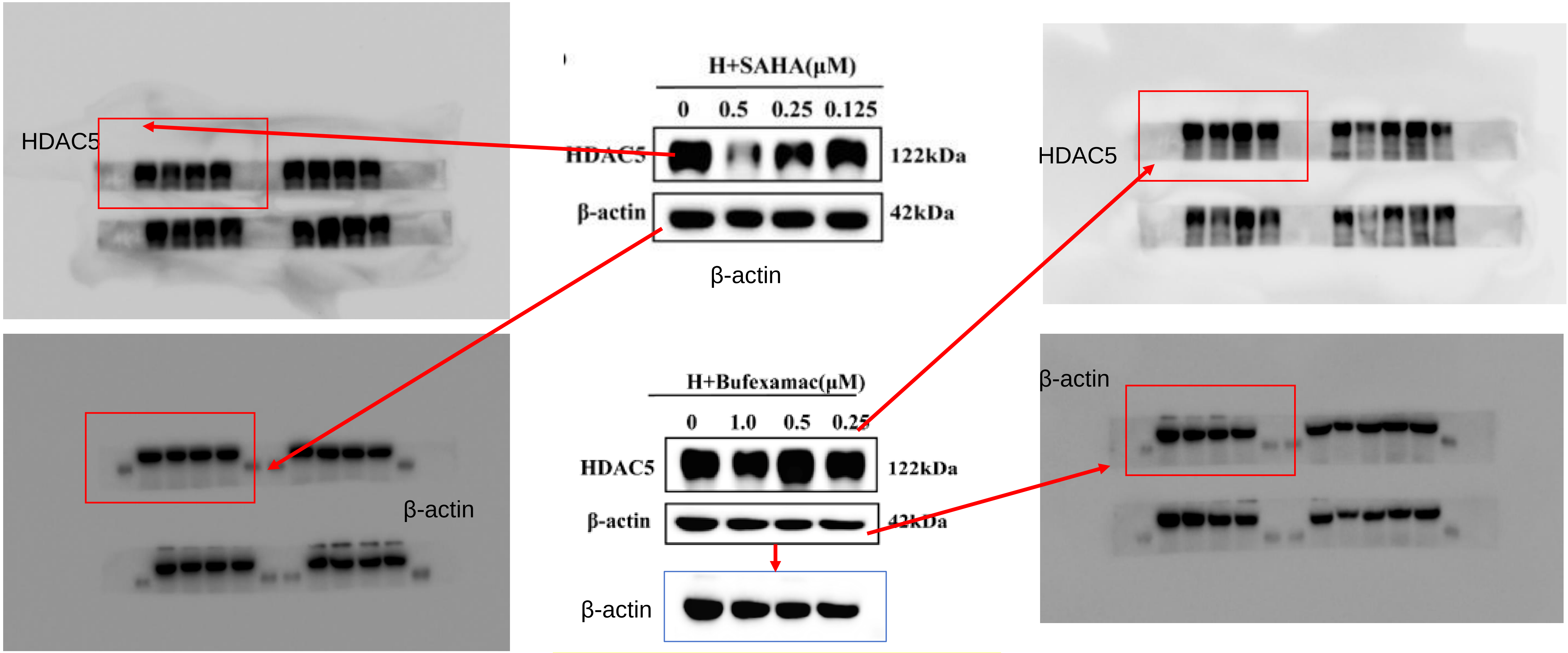

I'm very sorry, but beta-actin was distorted during cropping.

TSA (0,0.4,0.2,0.1nM)-HDAC5

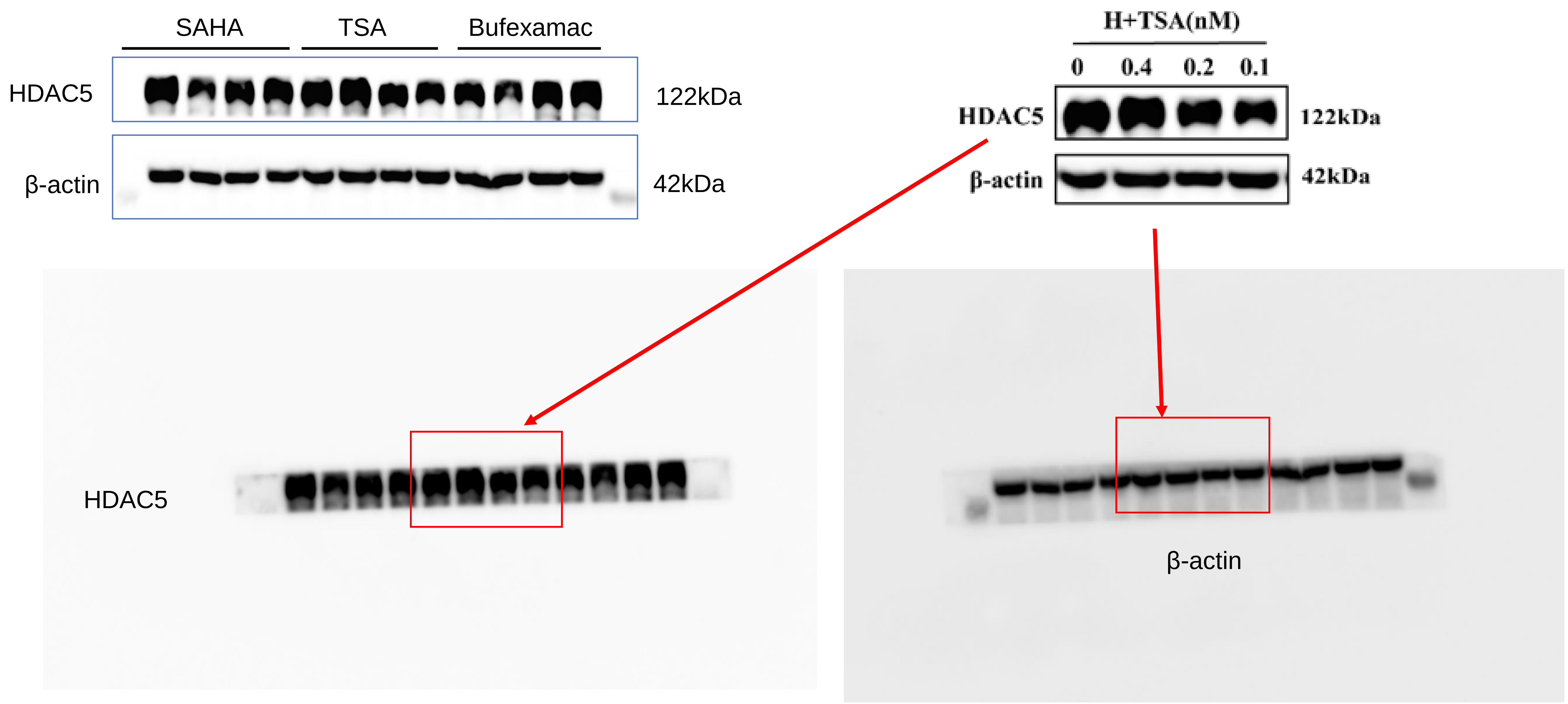

HDAC inhibitors-HDAC5

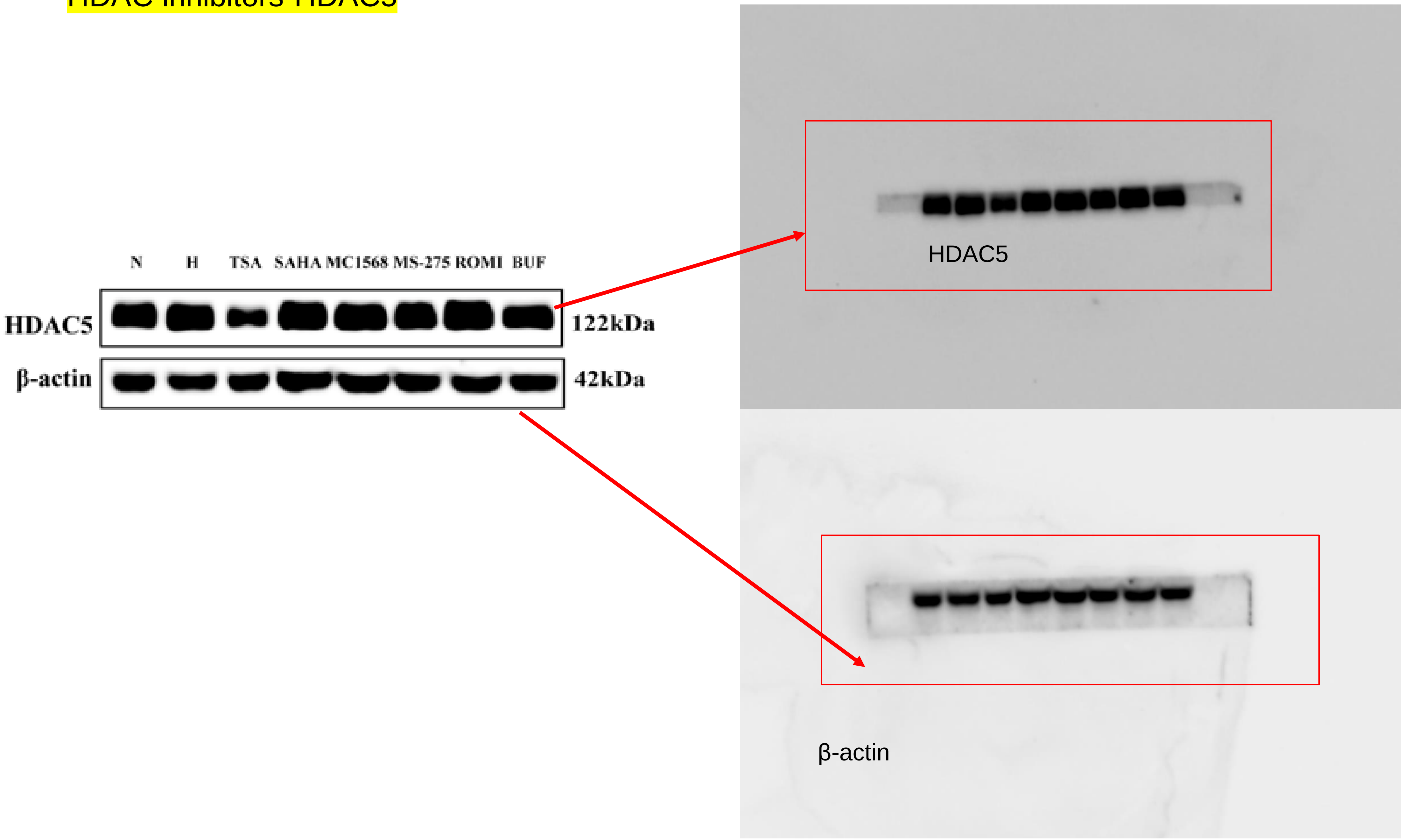

HDAC inhibitors-P-gp

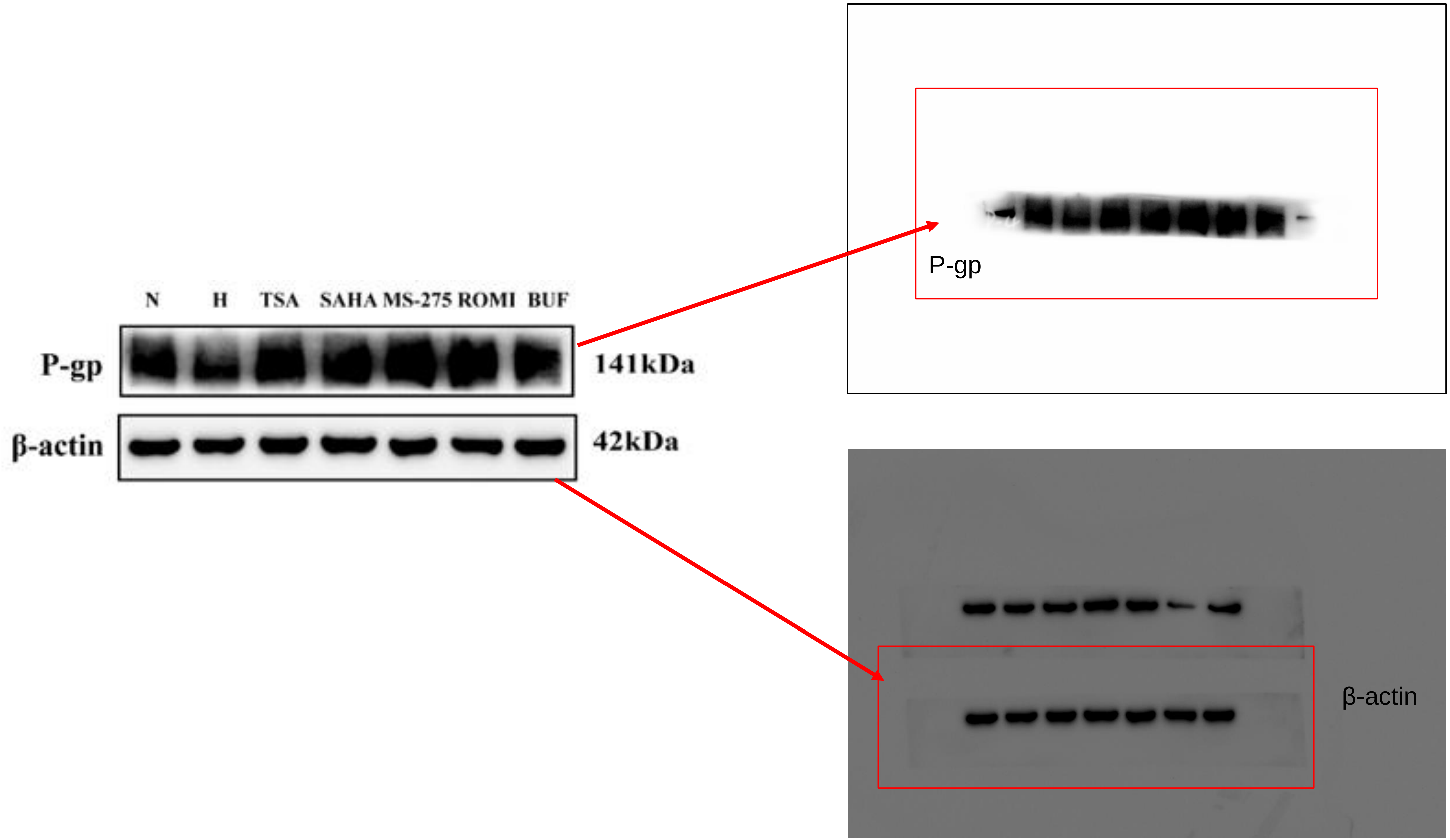

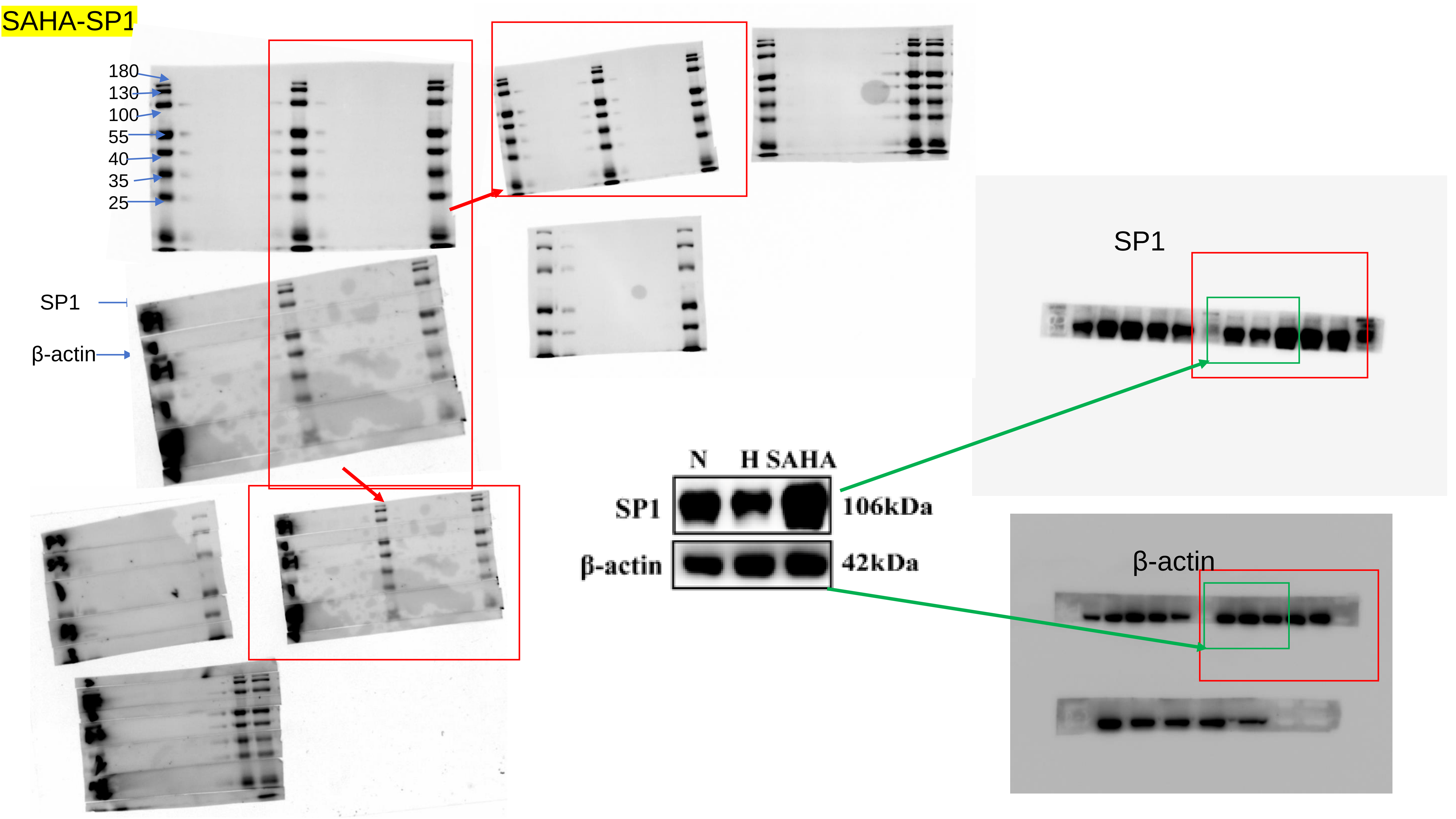
SAHA-SP1
180
130
100
55
40
35
25
SP1
β-actin
N
H
SAHA
SP1
106kDa
β-actin
42kDa
SP1
β-actin

siSP1-P-gp

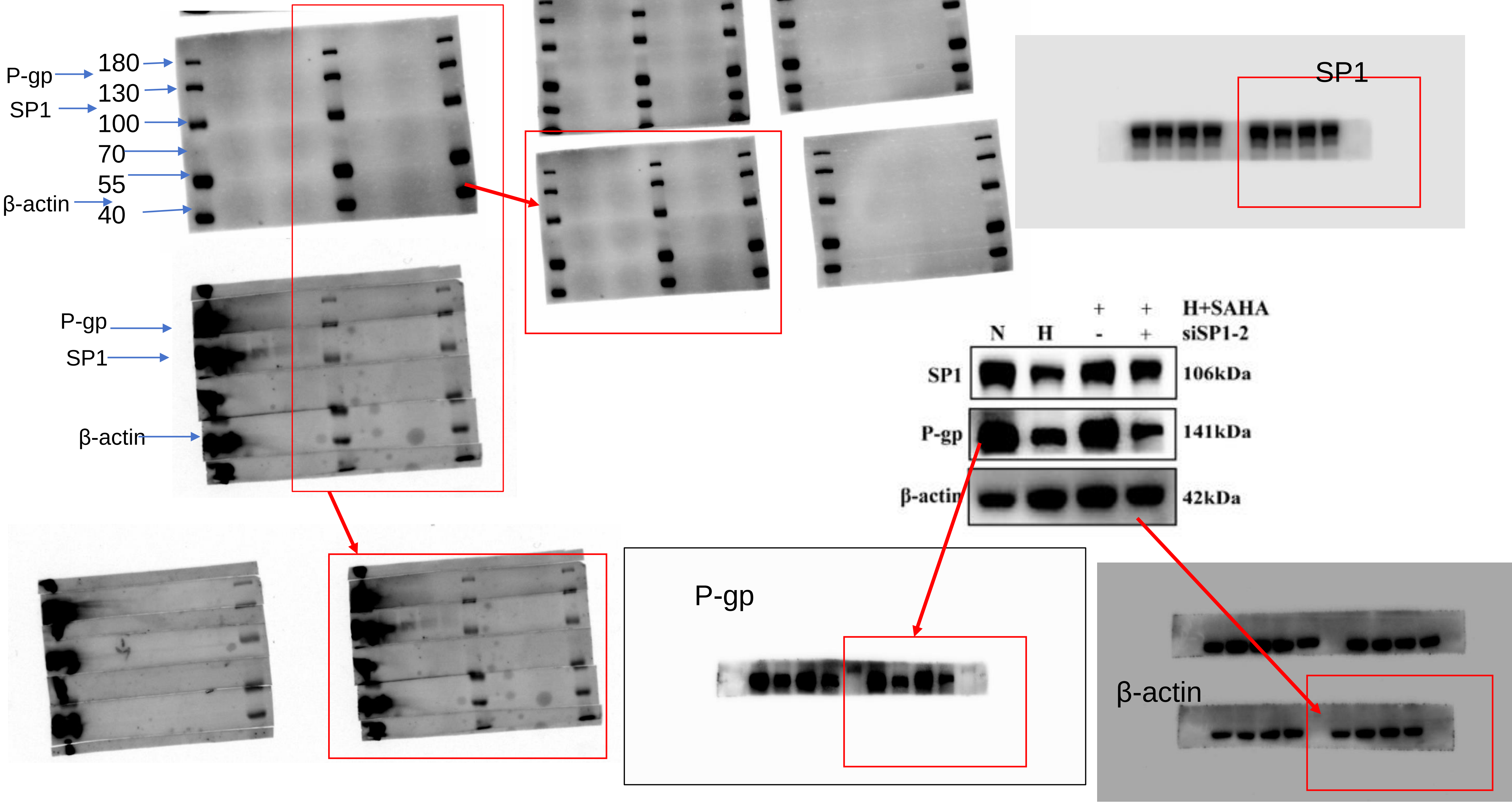
P-gp
180
130
SP1
100
70
55
β-actin
40
P-gp
SP1
β-actin
SP1
+
+
H+SAHA
N
H
-
+
siSP1-2
SP1
106kDa
P-gp
141kDa
β-actin
42kDa
P-gp
β-actin
