# Supplementary figures and images for: Regulatory mechanism of ABCB1 transcriptional repression by HDAC5 in rat hepatocytes under hypoxic environment
Source: Front Physiol. 2025 Apr 8;16:1520246. doi: 10.3389/fphys.2025.1520246 (PMC12011715; doi:10.3389/fphys.2025.1520246)

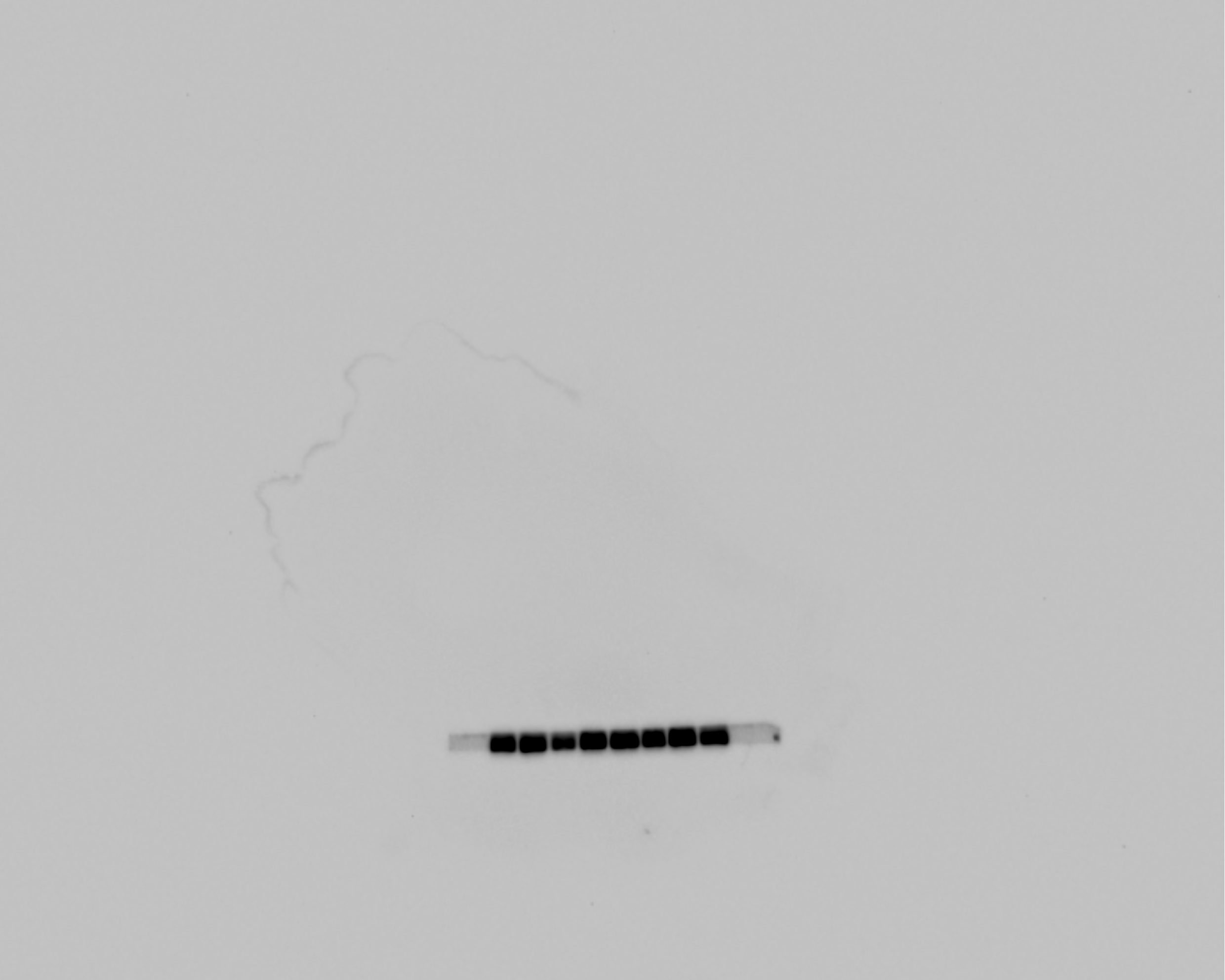

Supplement: Supplementary file 2 [file DataSheet3.zip › HDAC inhibitors-P-gp and HDAC5/HDAC inhibitiors-HDAC5/HDAC5.tif]

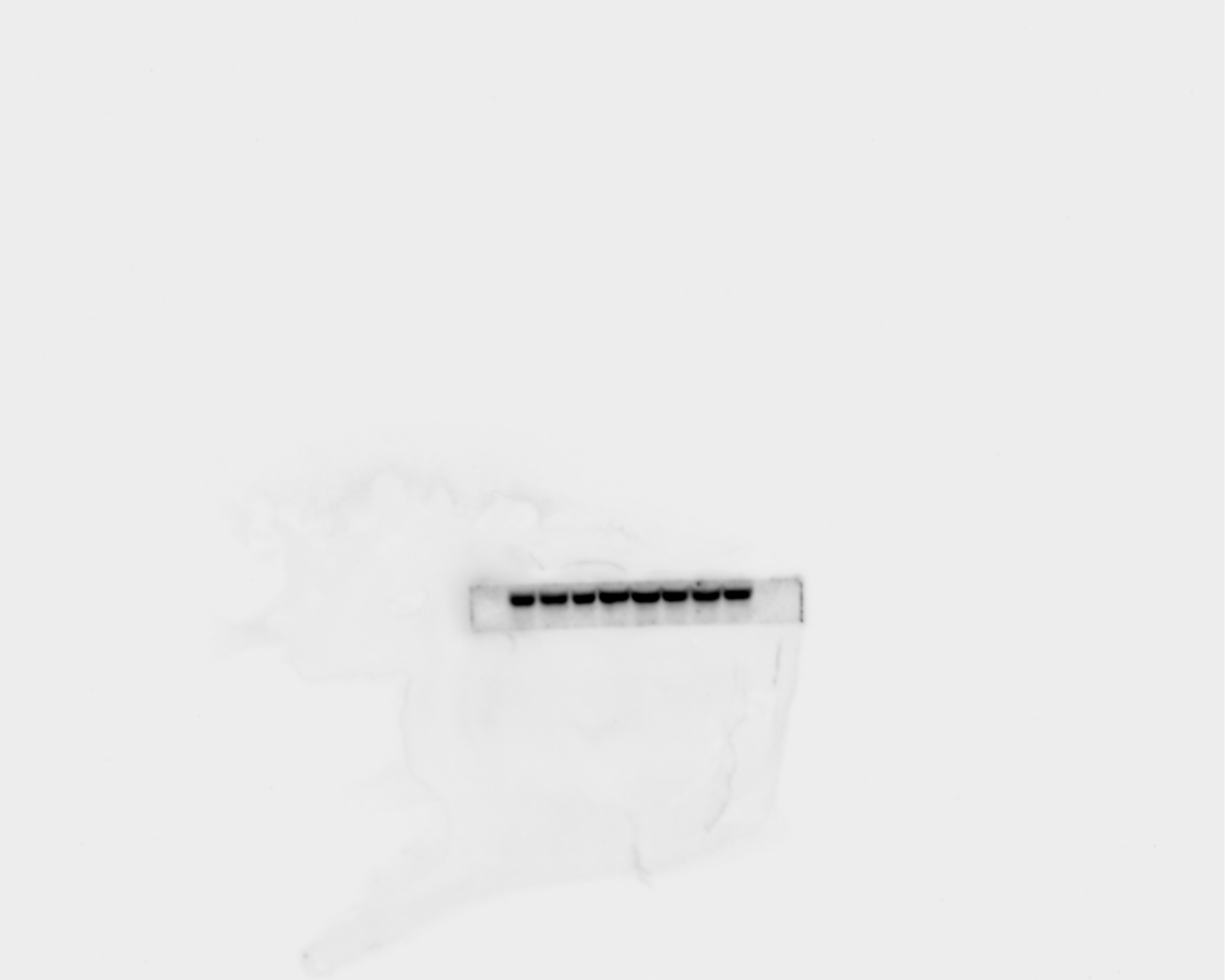

Supplement: Supplementary file 2 [file DataSheet3.zip › HDAC inhibitors-P-gp and HDAC5/HDAC inhibitiors-HDAC5/a┬-actin.tif]

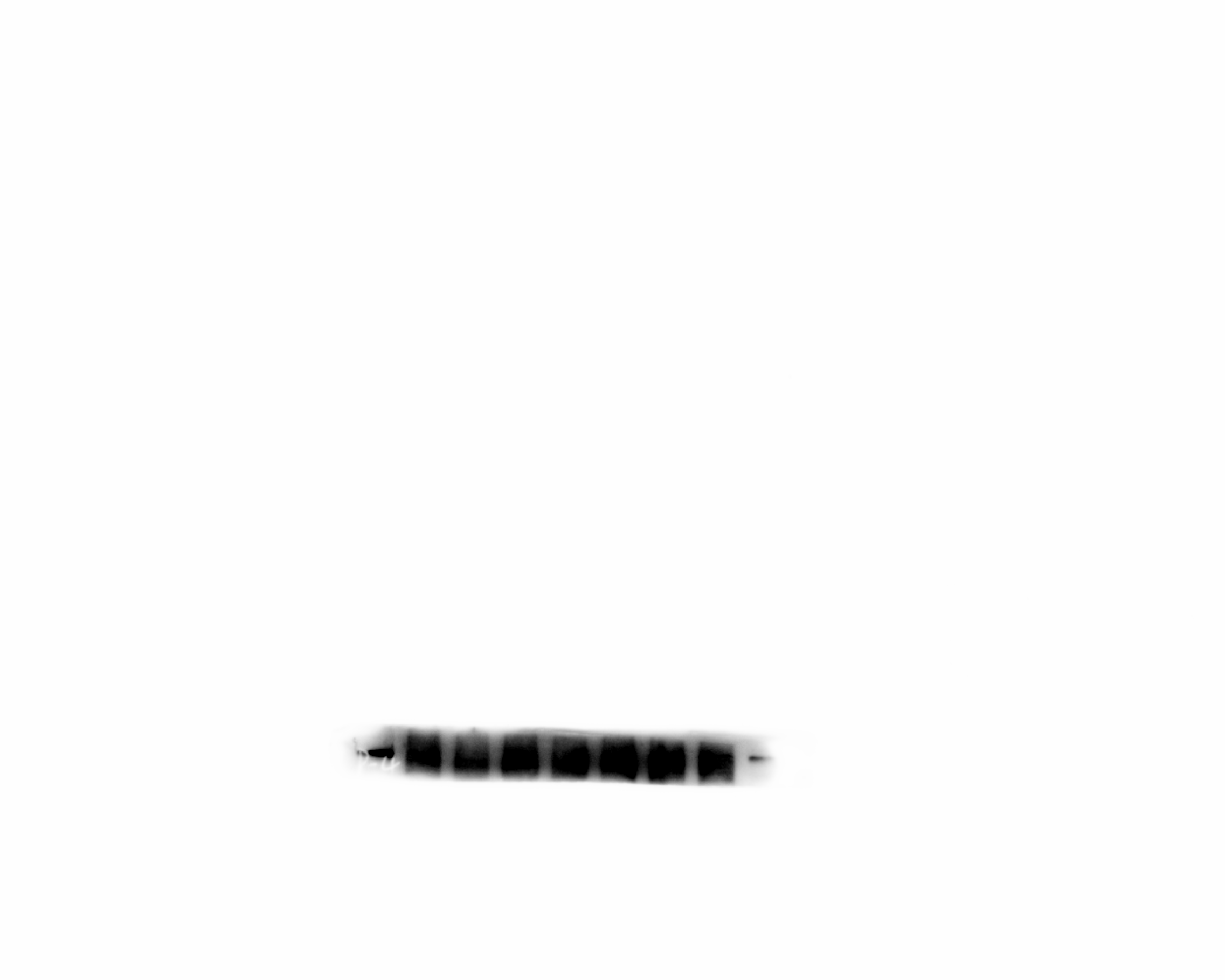

Supplement: Supplementary file 2 [file DataSheet3.zip › HDAC inhibitors-P-gp and HDAC5/HDAC inhibitors-P-gp/P-gp.tif]

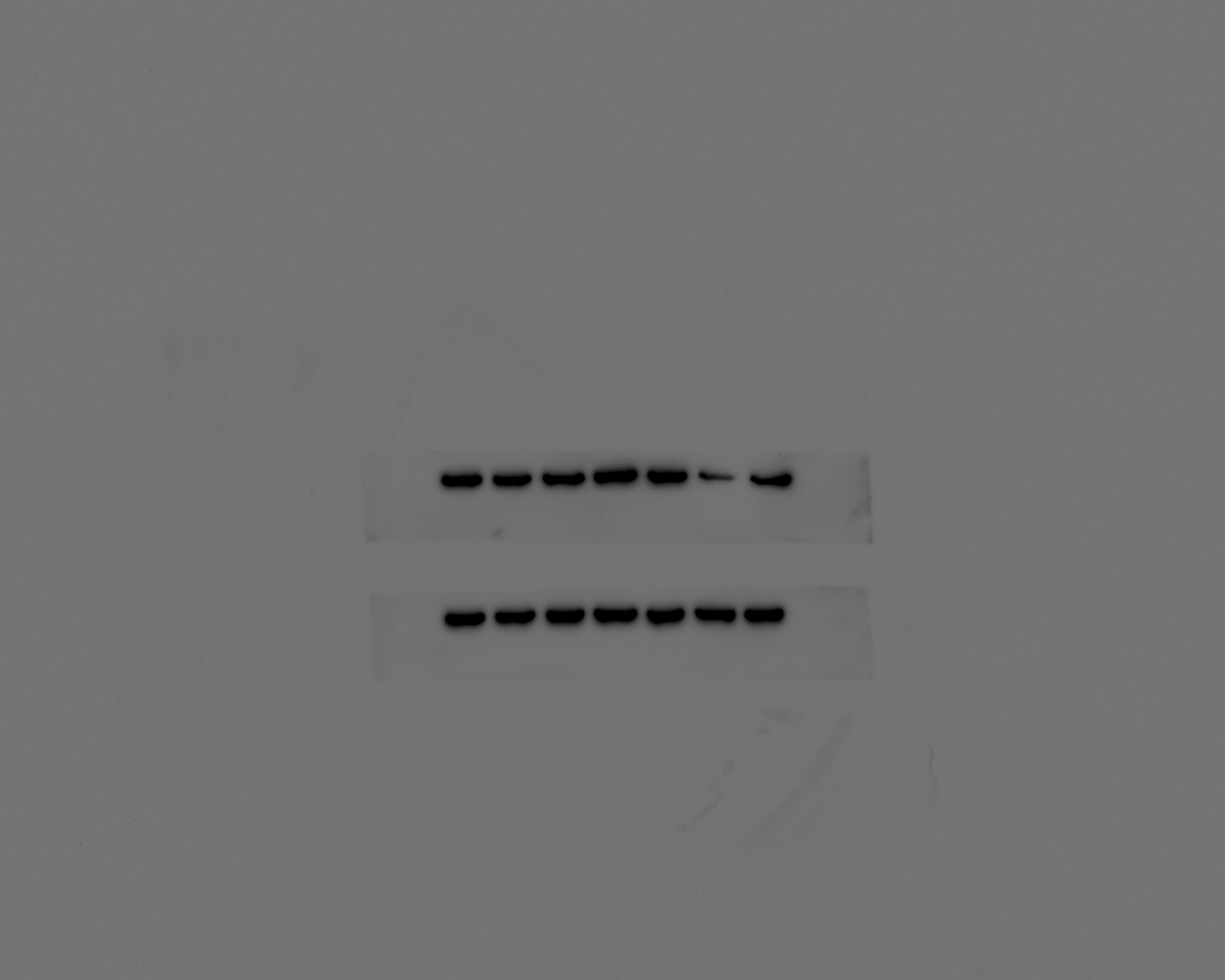

Supplement: Supplementary file 2 [file DataSheet3.zip › HDAC inhibitors-P-gp and HDAC5/HDAC inhibitors-P-gp/a┬-actin.tif]

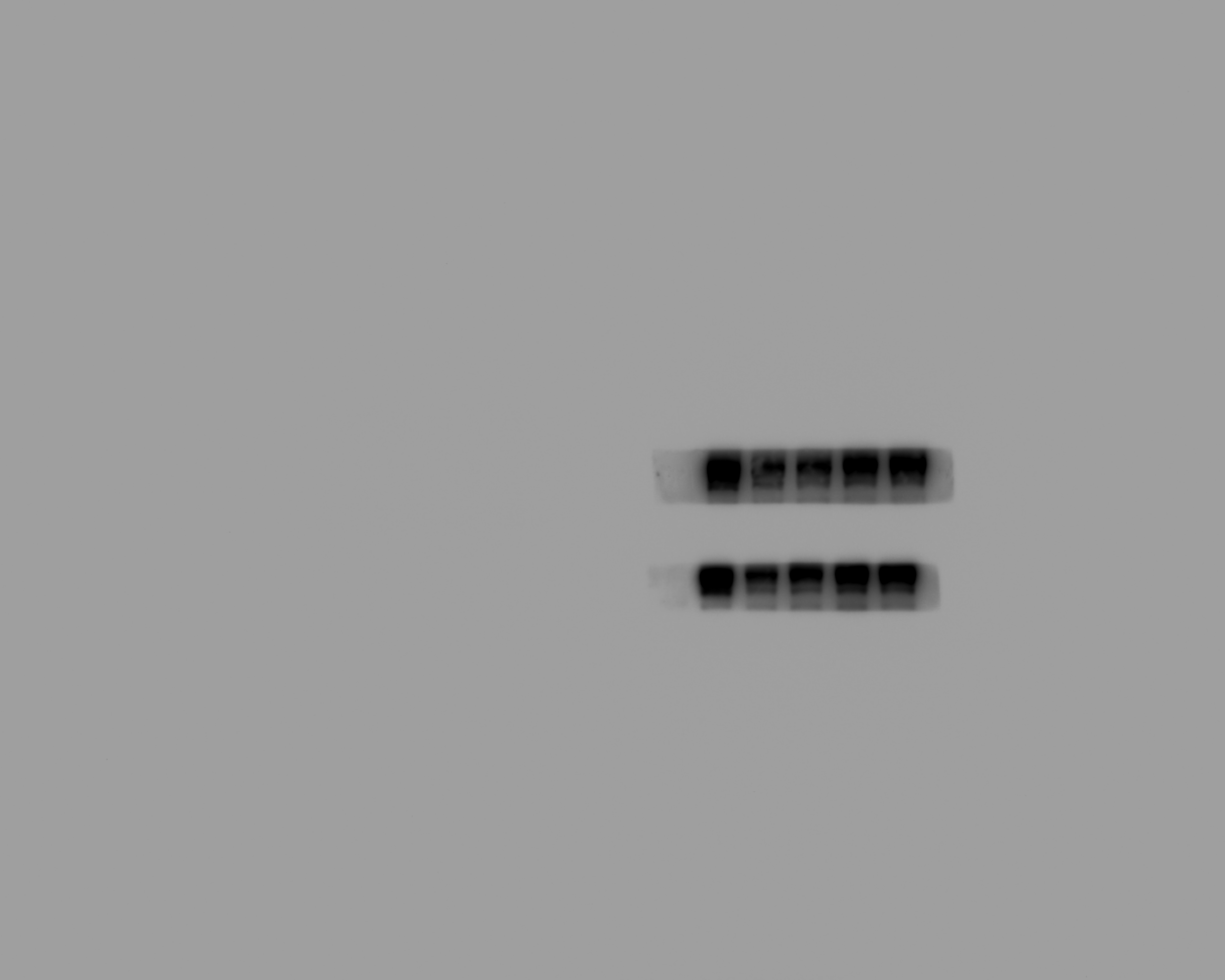

Supplement: Supplementary file 4 [file DataSheet11.zip › siHDAC5-SP1/SP1.tif]

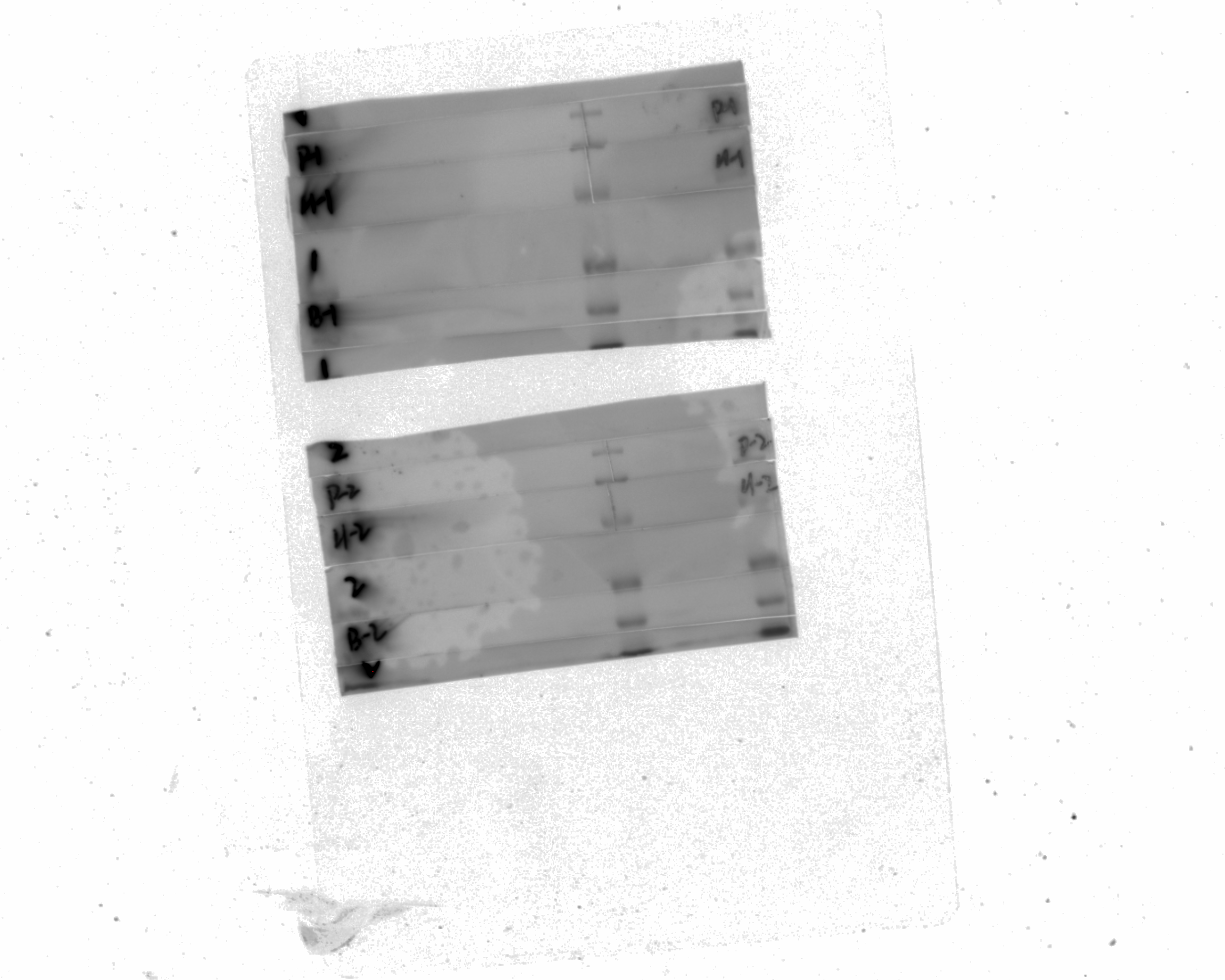

Supplement: Supplementary file 4 [file DataSheet11.zip › siHDAC5-SP1/Western blot membrane cutting..tif]

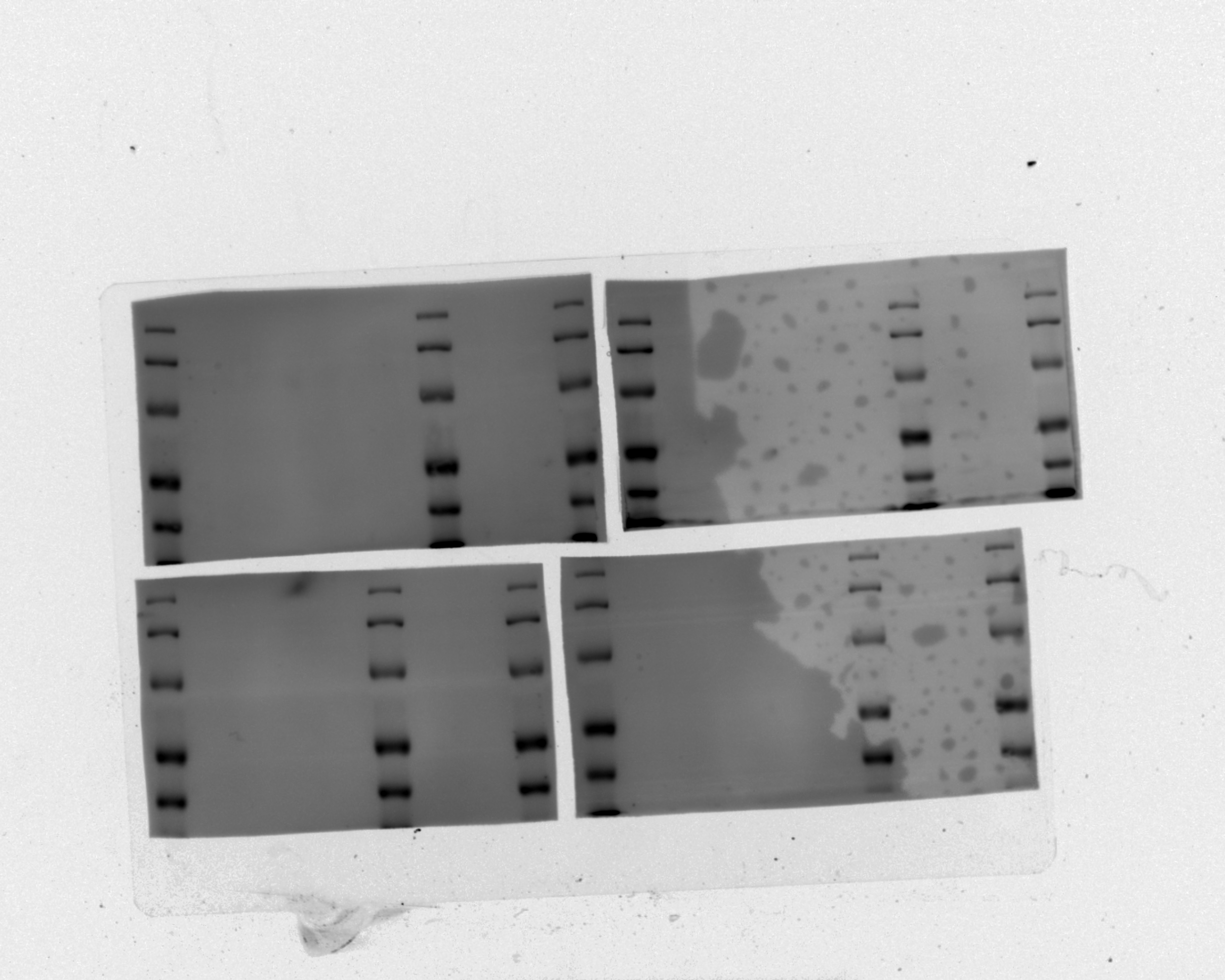

Supplement: Supplementary file 4 [file DataSheet11.zip › siHDAC5-SP1/Whole Western blot membrane..tif]

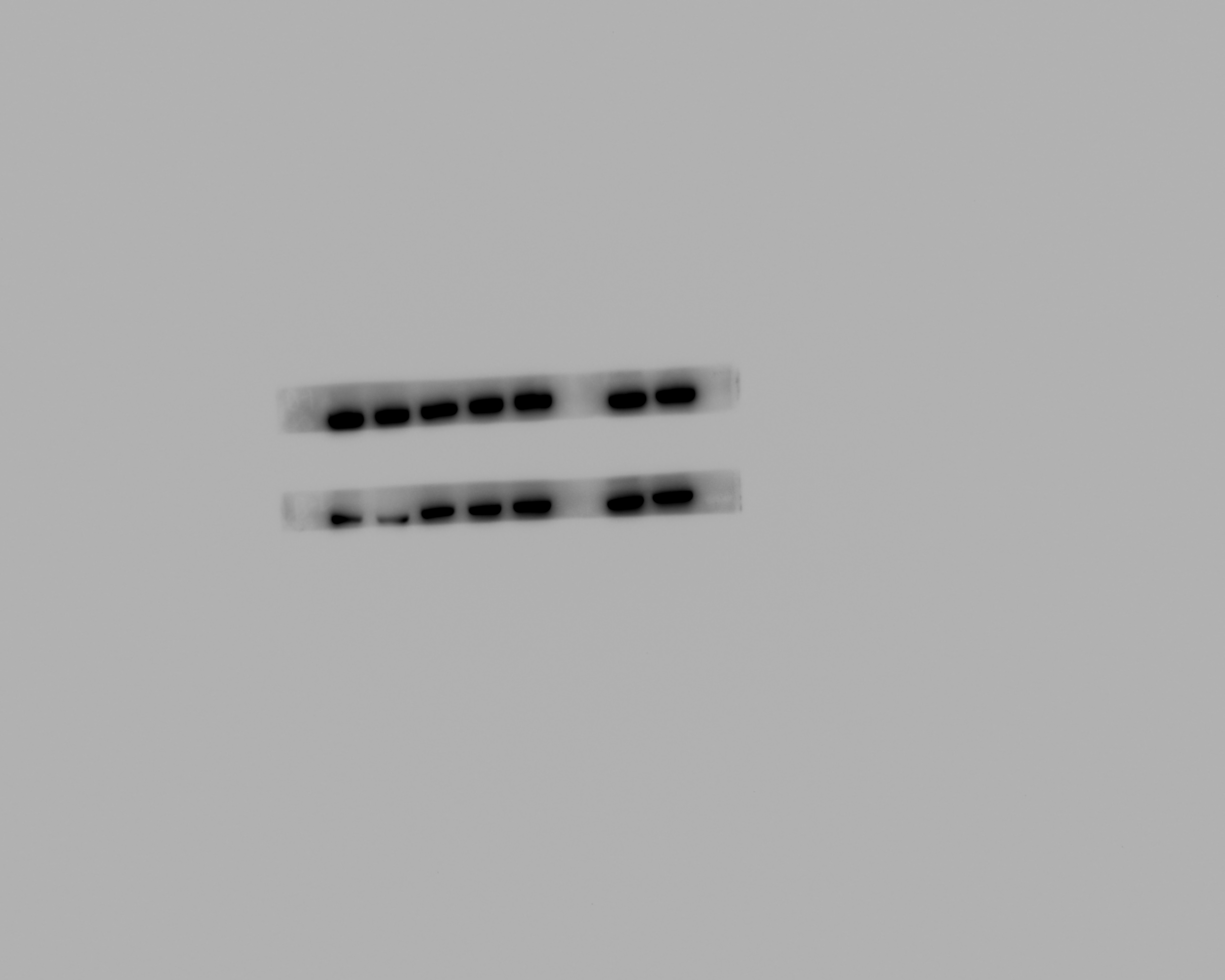

Supplement: Supplementary file 4 [file DataSheet11.zip › siHDAC5-SP1/a┬-actin.tif]

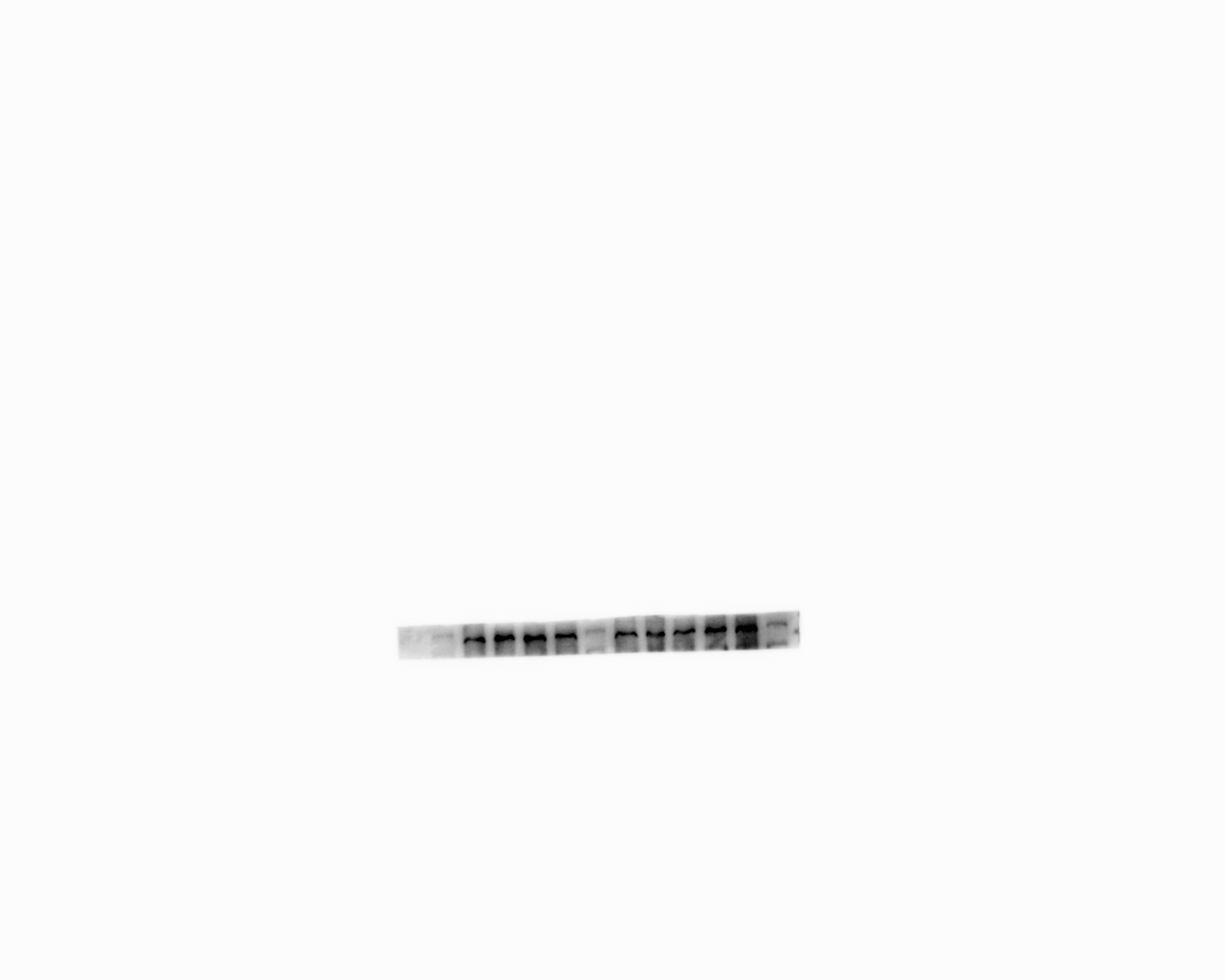

Supplement: Supplementary file 5 [file DataSheet8.zip › SAHA TSA and Bufexamac-P-gp and H3K9ac/Bufexamac-P-gp/P-gp.tif]

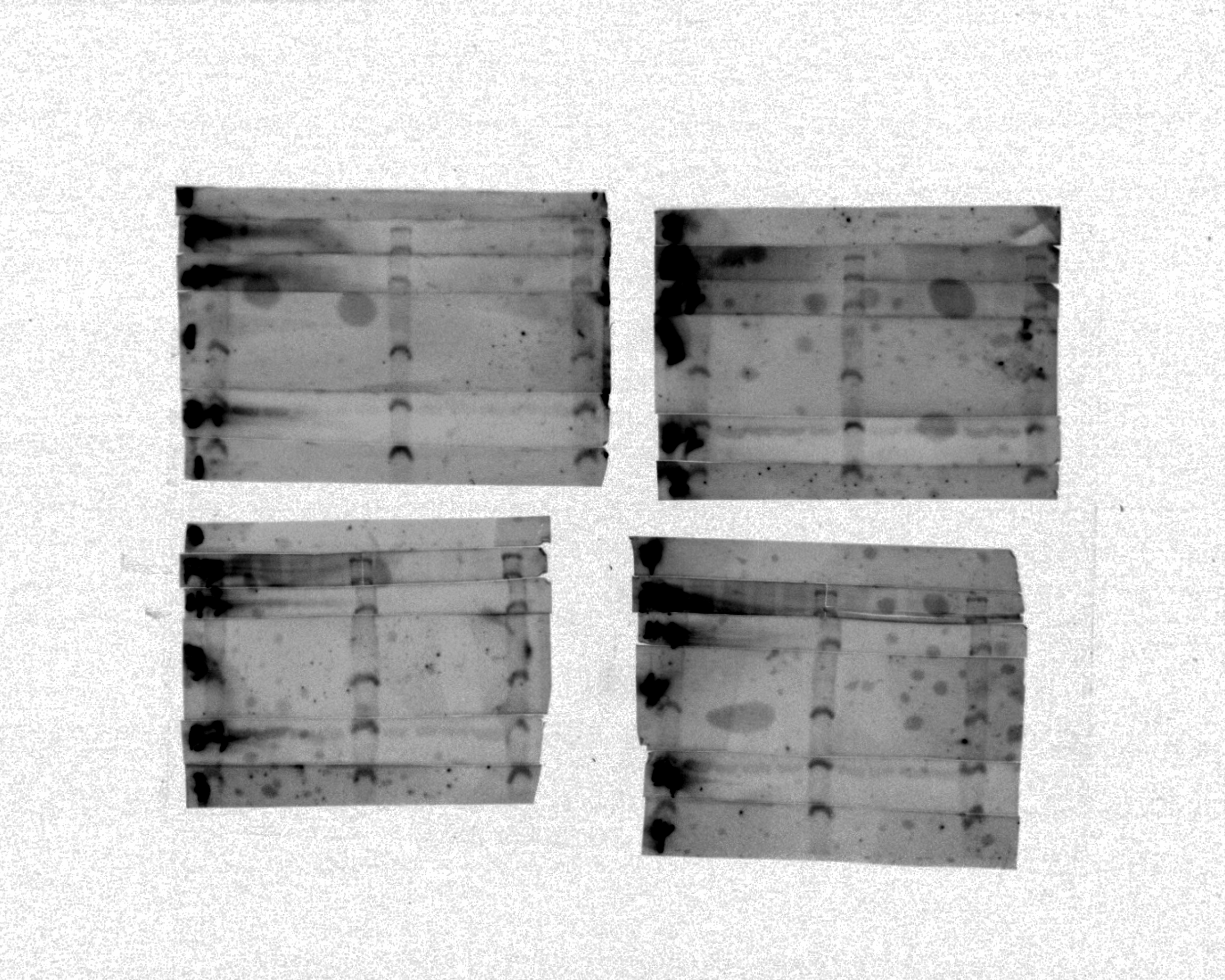

Supplement: Supplementary file 5 [file DataSheet8.zip › SAHA TSA and Bufexamac-P-gp and H3K9ac/Bufexamac-P-gp/Western blot membrane cutting..tif]

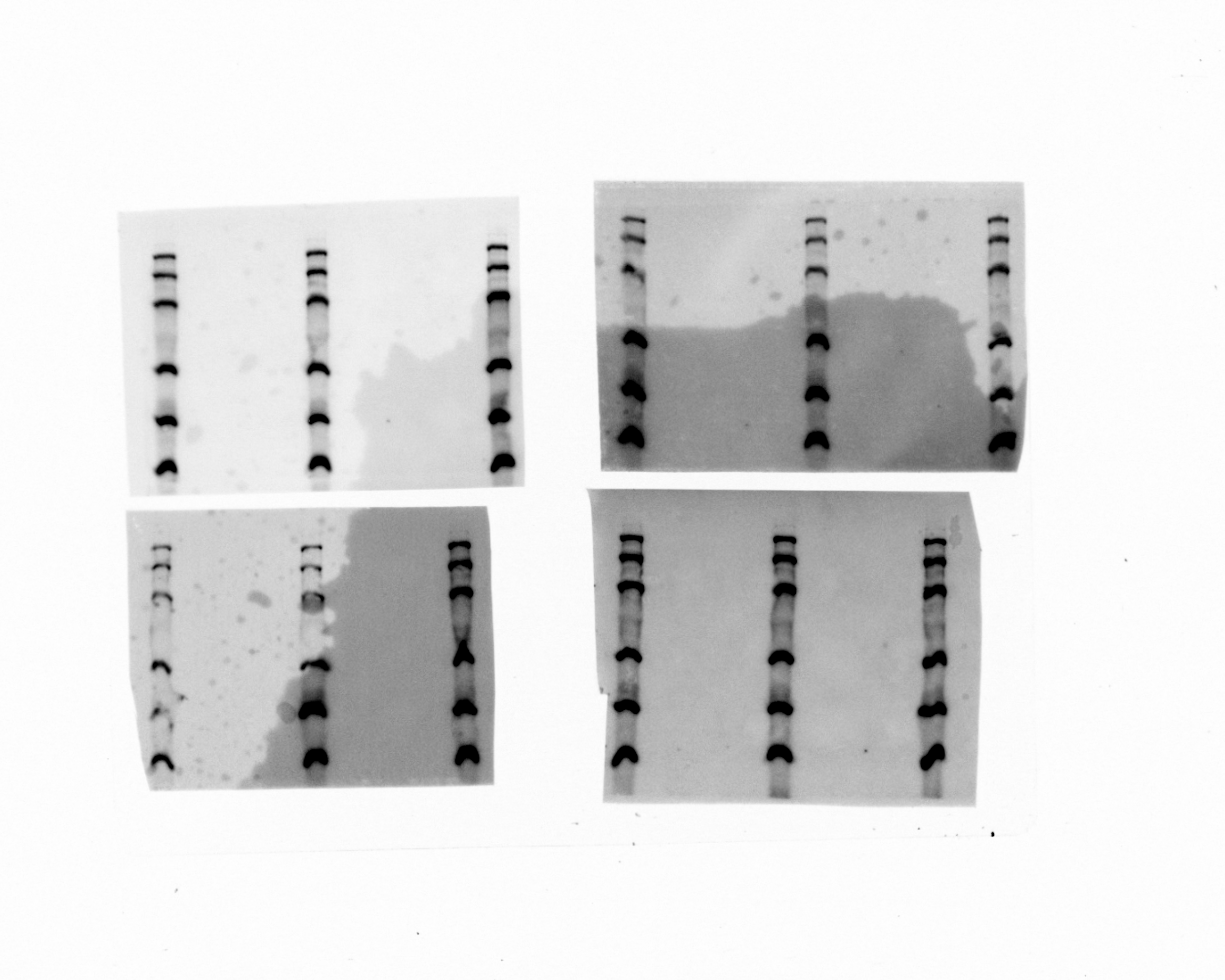

Supplement: Supplementary file 5 [file DataSheet8.zip › SAHA TSA and Bufexamac-P-gp and H3K9ac/Bufexamac-P-gp/Whole Western blot membrane..tif]

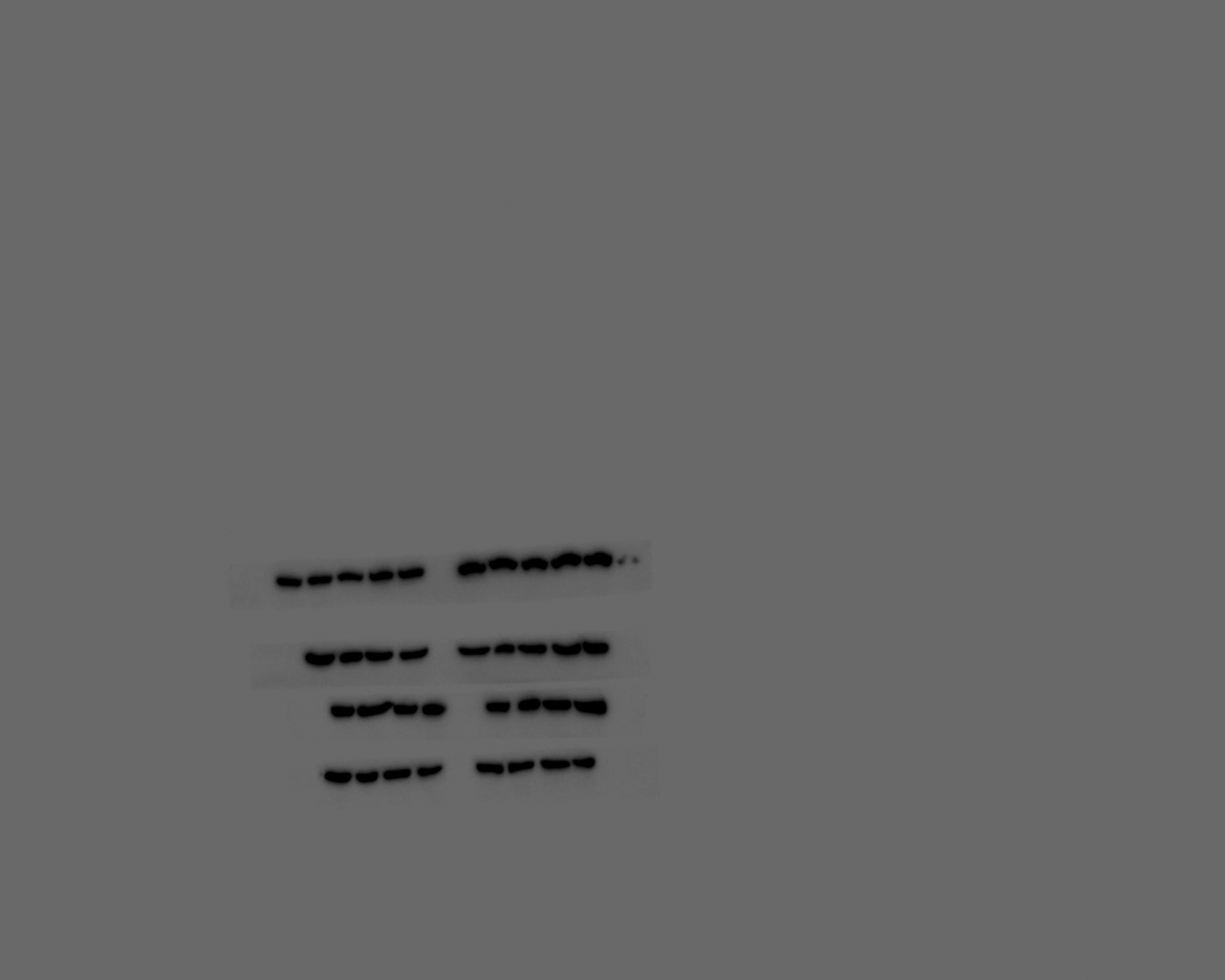

Supplement: Supplementary file 5 [file DataSheet8.zip › SAHA TSA and Bufexamac-P-gp and H3K9ac/Bufexamac-P-gp/a┬-actin.tif]

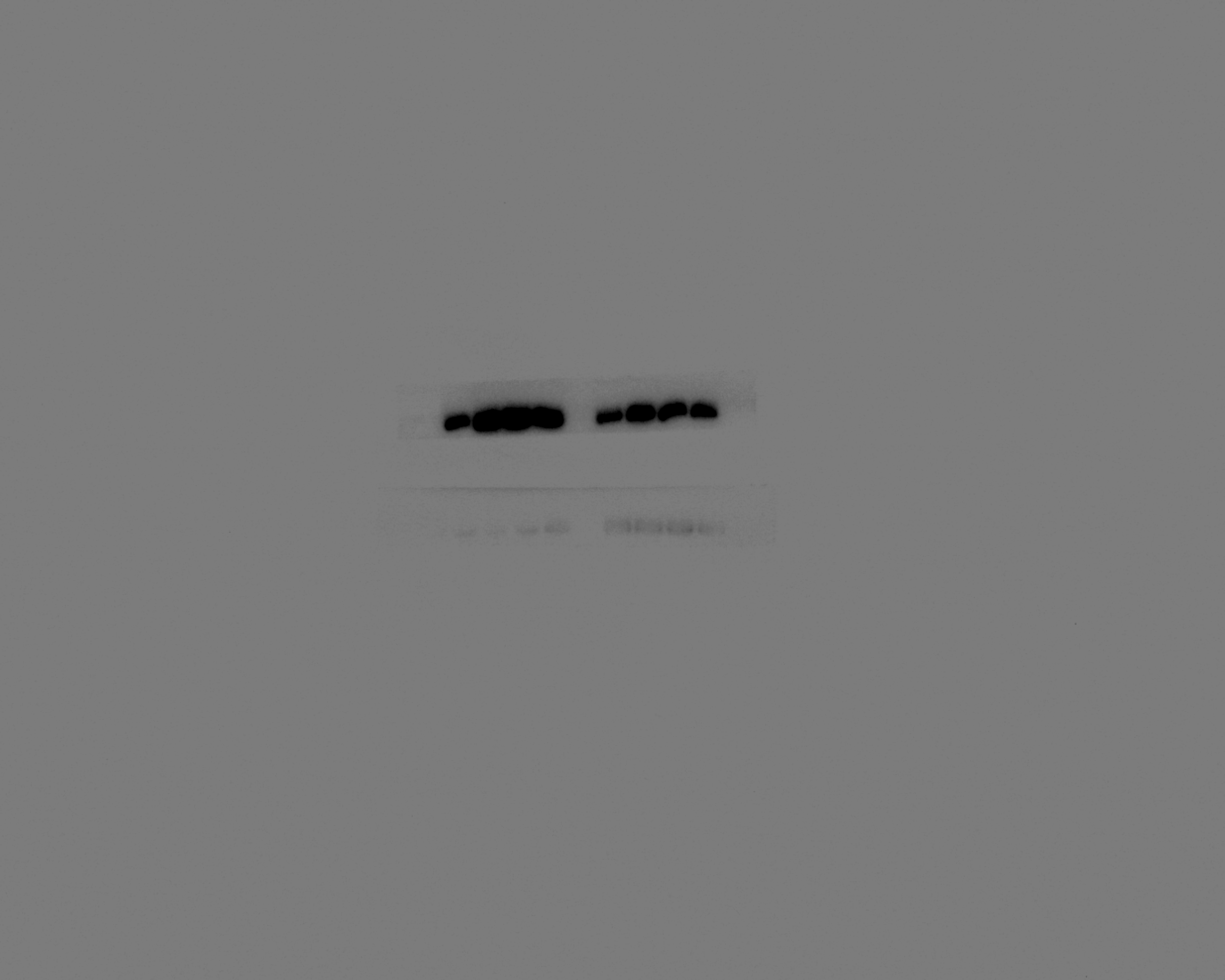

Supplement: Supplementary file 5 [file DataSheet8.zip › SAHA TSA and Bufexamac-P-gp and H3K9ac/TSA SAHA-P-gp SAHA-BU-H3K9/SAHA and Bufexamac-H3K9ac/SAHA and Bufexamac-H3K9ac.tif]

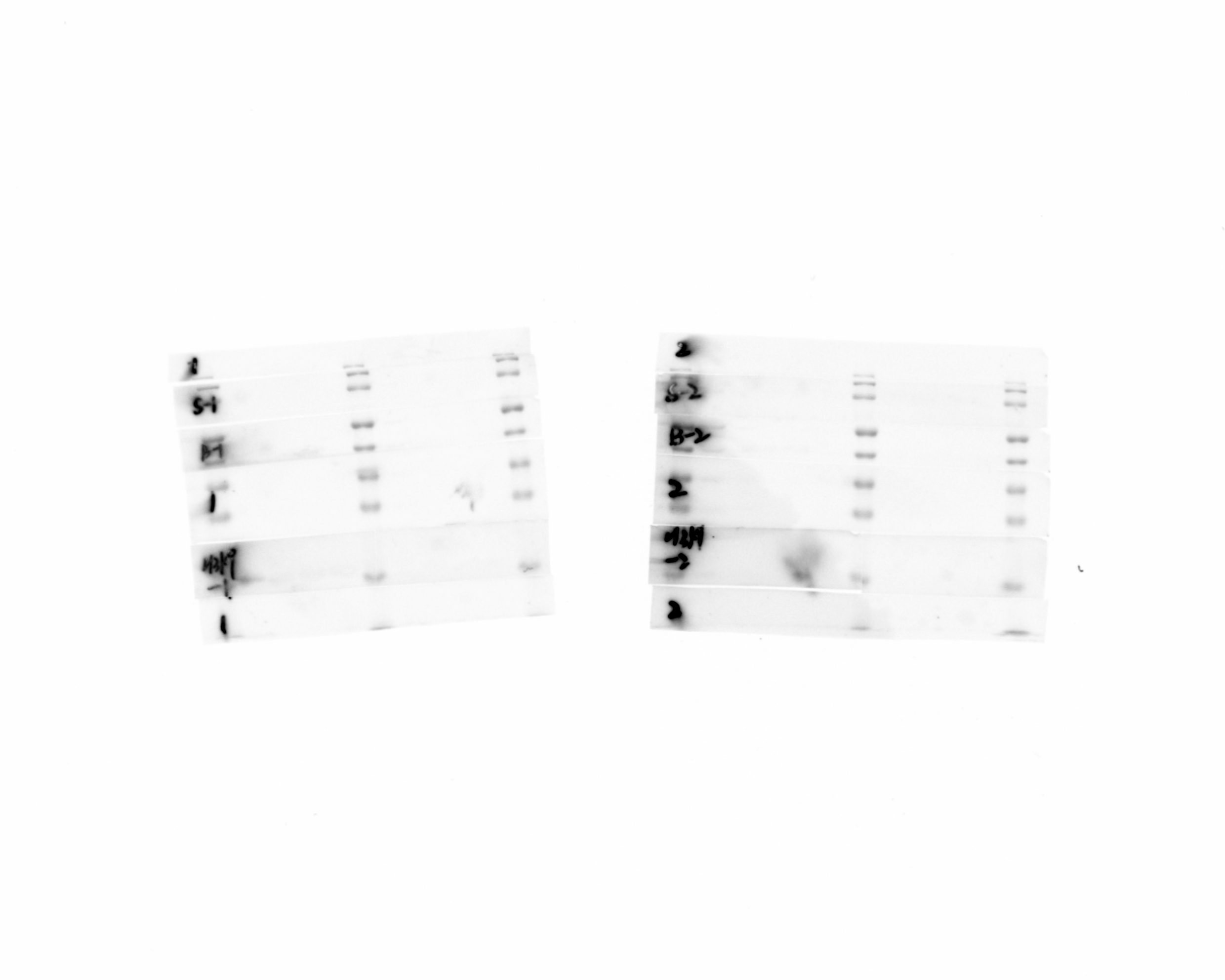

Supplement: Supplementary file 5 [file DataSheet8.zip › SAHA TSA and Bufexamac-P-gp and H3K9ac/TSA SAHA-P-gp SAHA-BU-H3K9/SAHA and Bufexamac-H3K9ac/SAHA and Bufexamac-Western blot membrane cutting..tif]

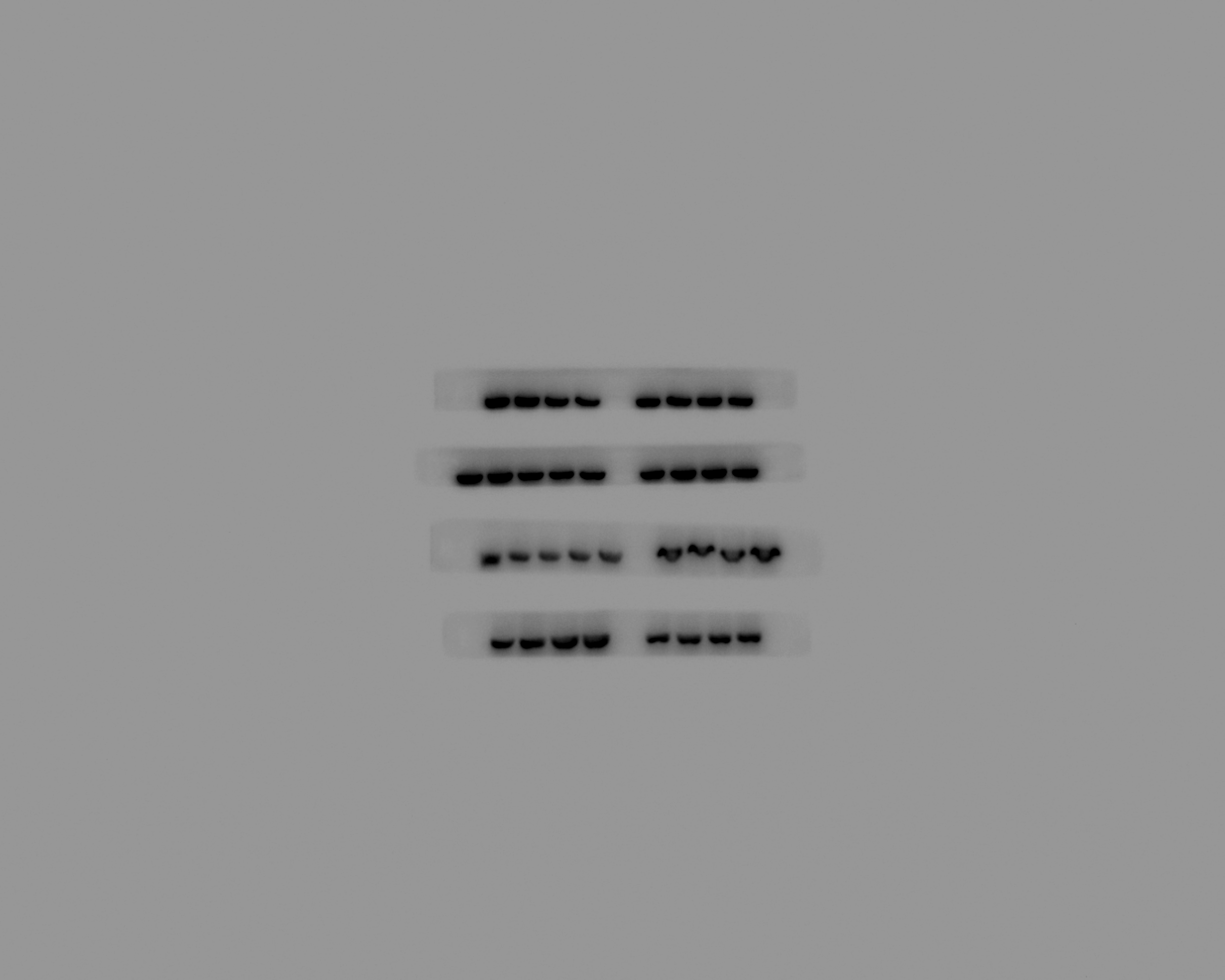

Supplement: Supplementary file 5 [file DataSheet8.zip › SAHA TSA and Bufexamac-P-gp and H3K9ac/TSA SAHA-P-gp SAHA-BU-H3K9/SAHA and Bufexamac-H3K9ac/SAHA and Bufexamac-a┬-actin.tif]

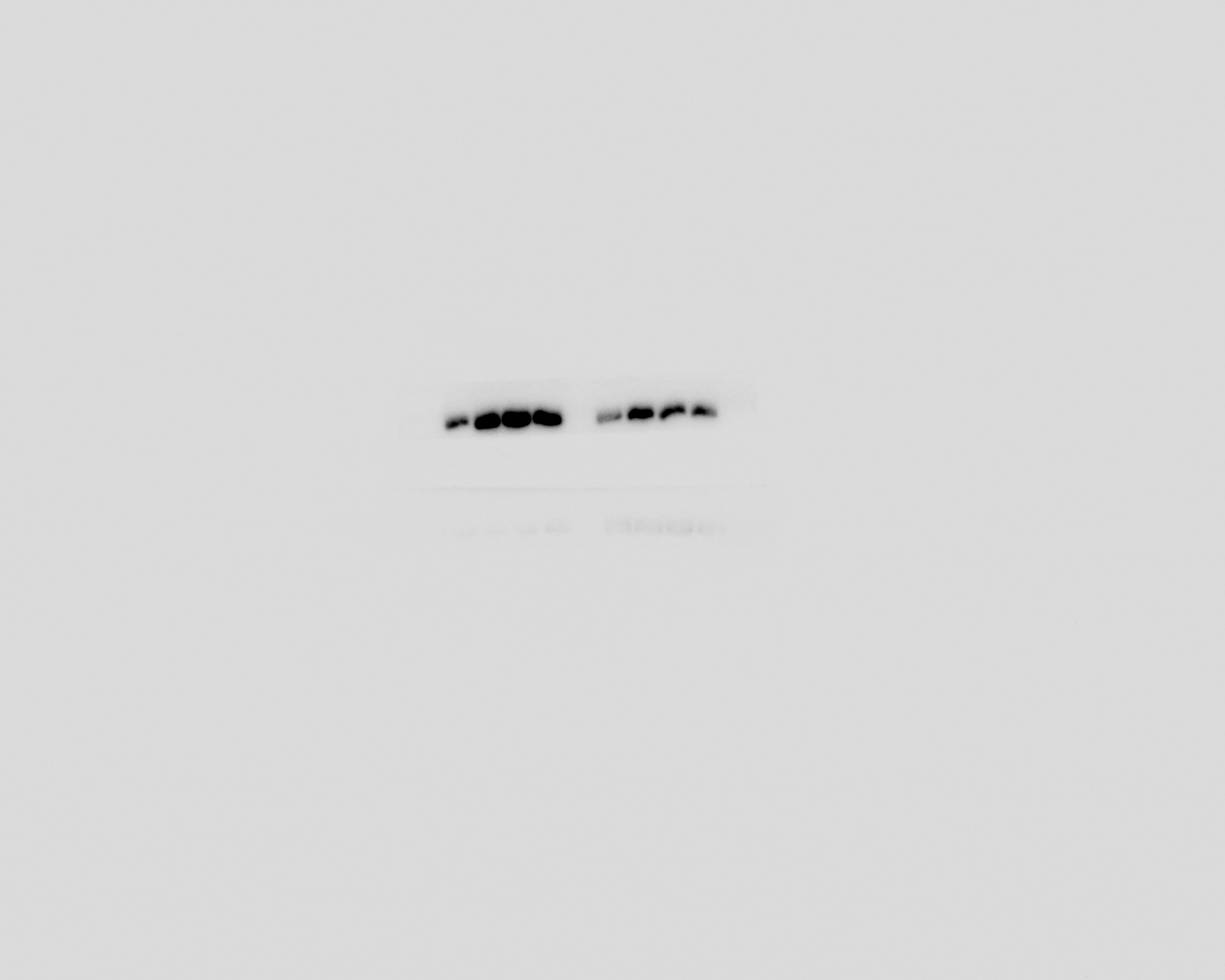

Supplement: Supplementary file 5 [file DataSheet8.zip › SAHA TSA and Bufexamac-P-gp and H3K9ac/TSA SAHA-P-gp SAHA-BU-H3K9/SAHA and Bufexamac-H3K9ac/SAHA-H3K9ac.tif]

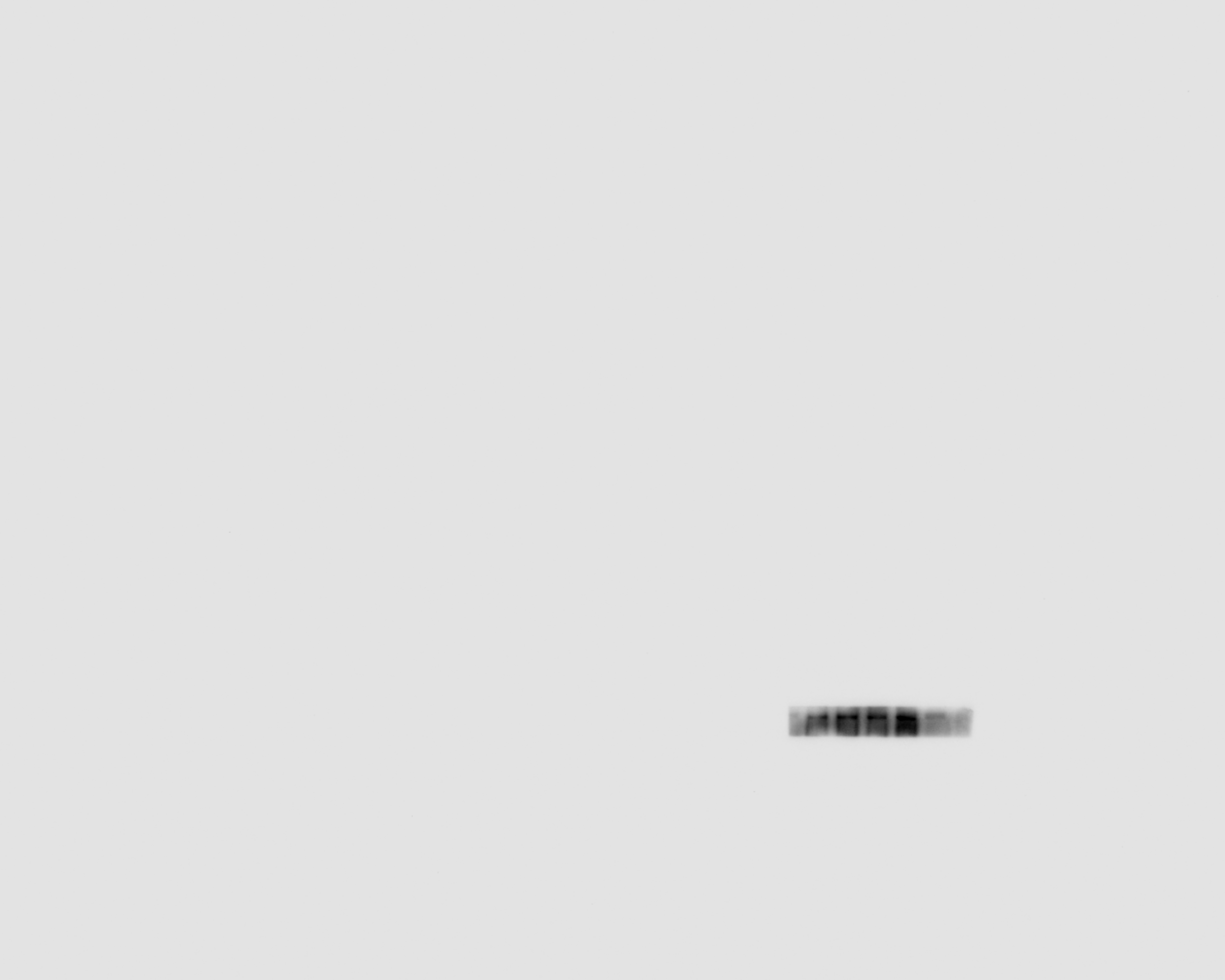

Supplement: Supplementary file 5 [file DataSheet8.zip › SAHA TSA and Bufexamac-P-gp and H3K9ac/TSA SAHA-P-gp SAHA-BU-H3K9/TSA and SAHA-P-gp/SAHA-P-gp.tif]

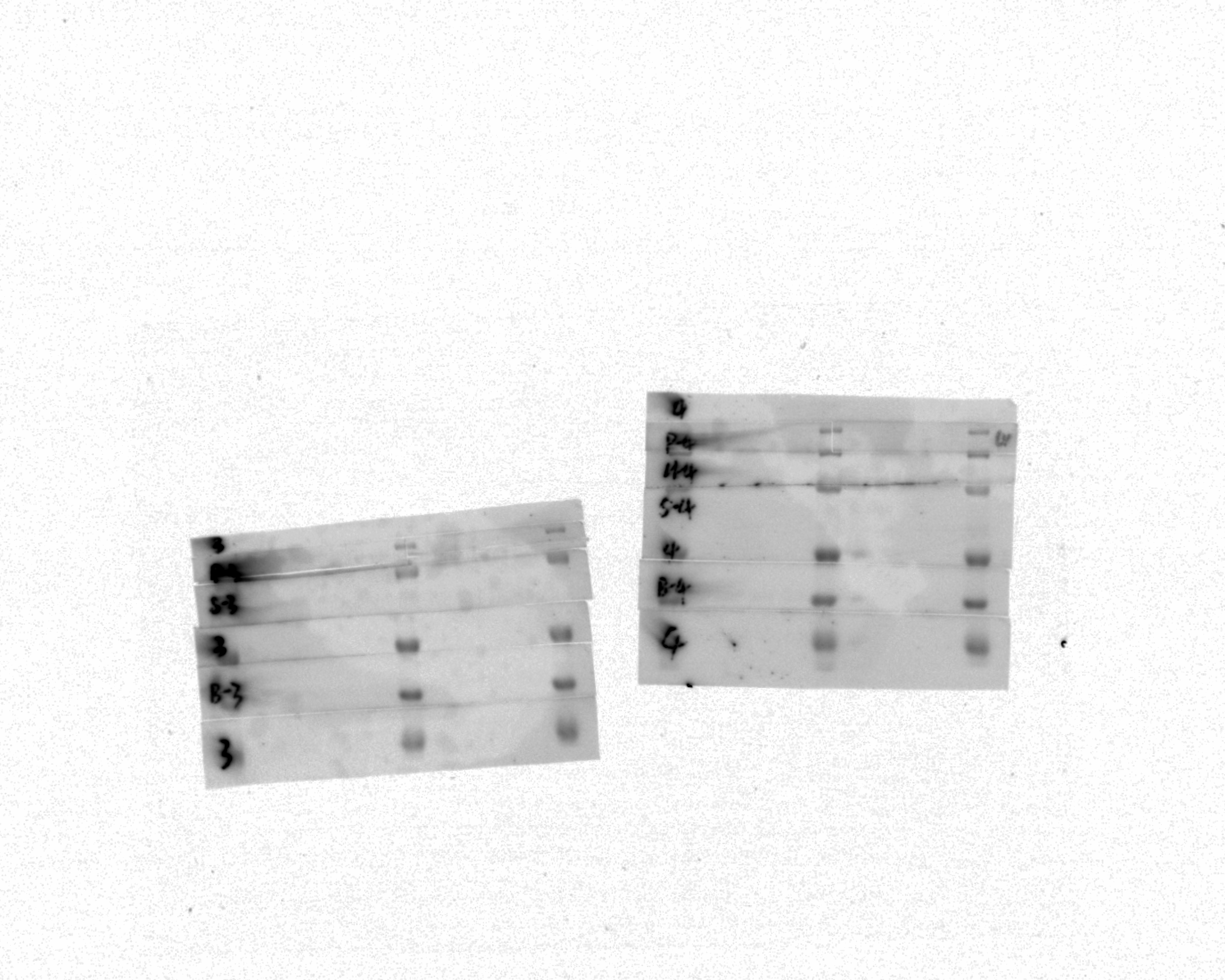

Supplement: Supplementary file 5 [file DataSheet8.zip › SAHA TSA and Bufexamac-P-gp and H3K9ac/TSA SAHA-P-gp SAHA-BU-H3K9/TSA and SAHA-P-gp/TSA and Bufexamac-Western blot membrane cutting..tif]

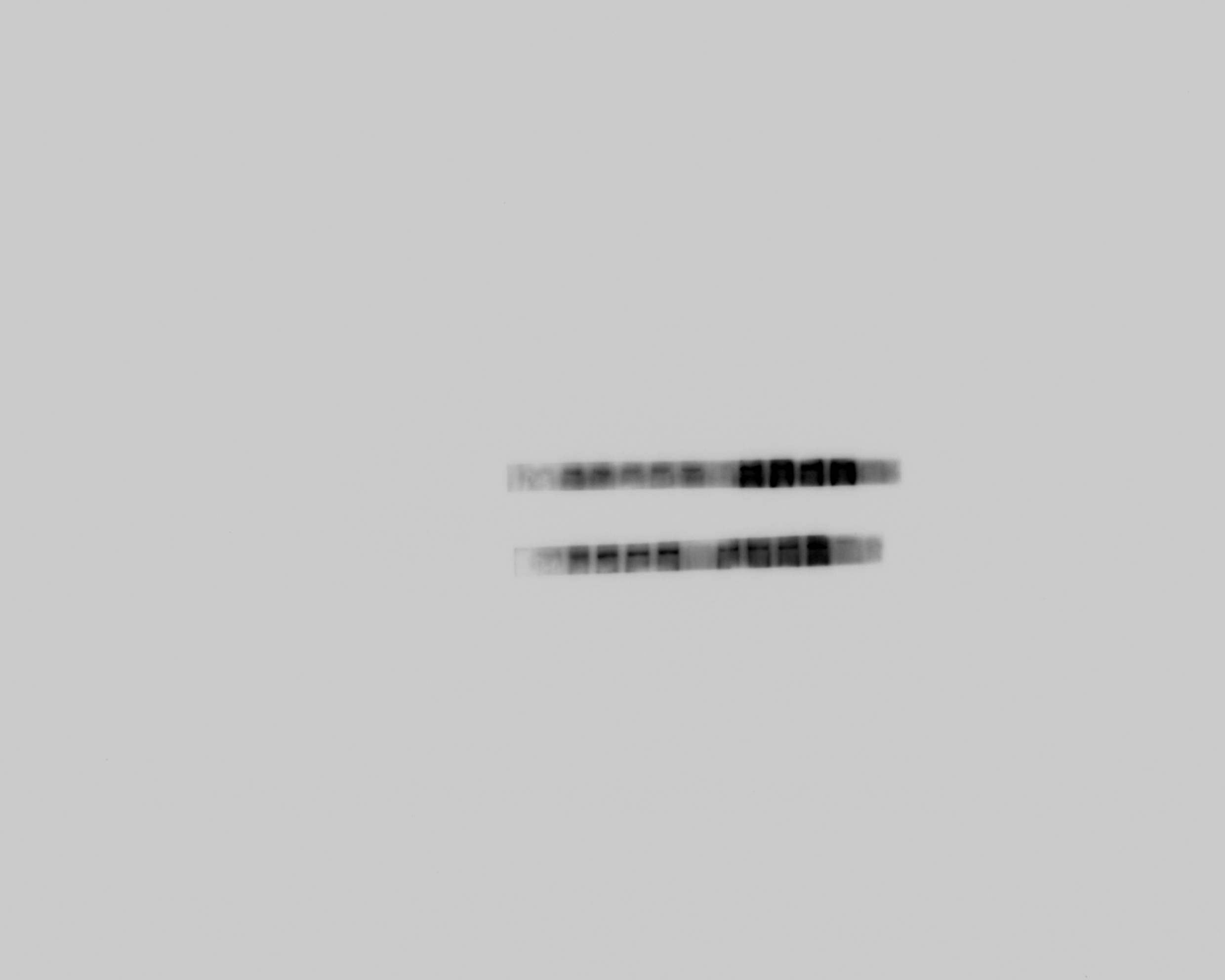

Supplement: Supplementary file 5 [file DataSheet8.zip › SAHA TSA and Bufexamac-P-gp and H3K9ac/TSA SAHA-P-gp SAHA-BU-H3K9/TSA and SAHA-P-gp/TSA and SAHA-P-gp.tif]

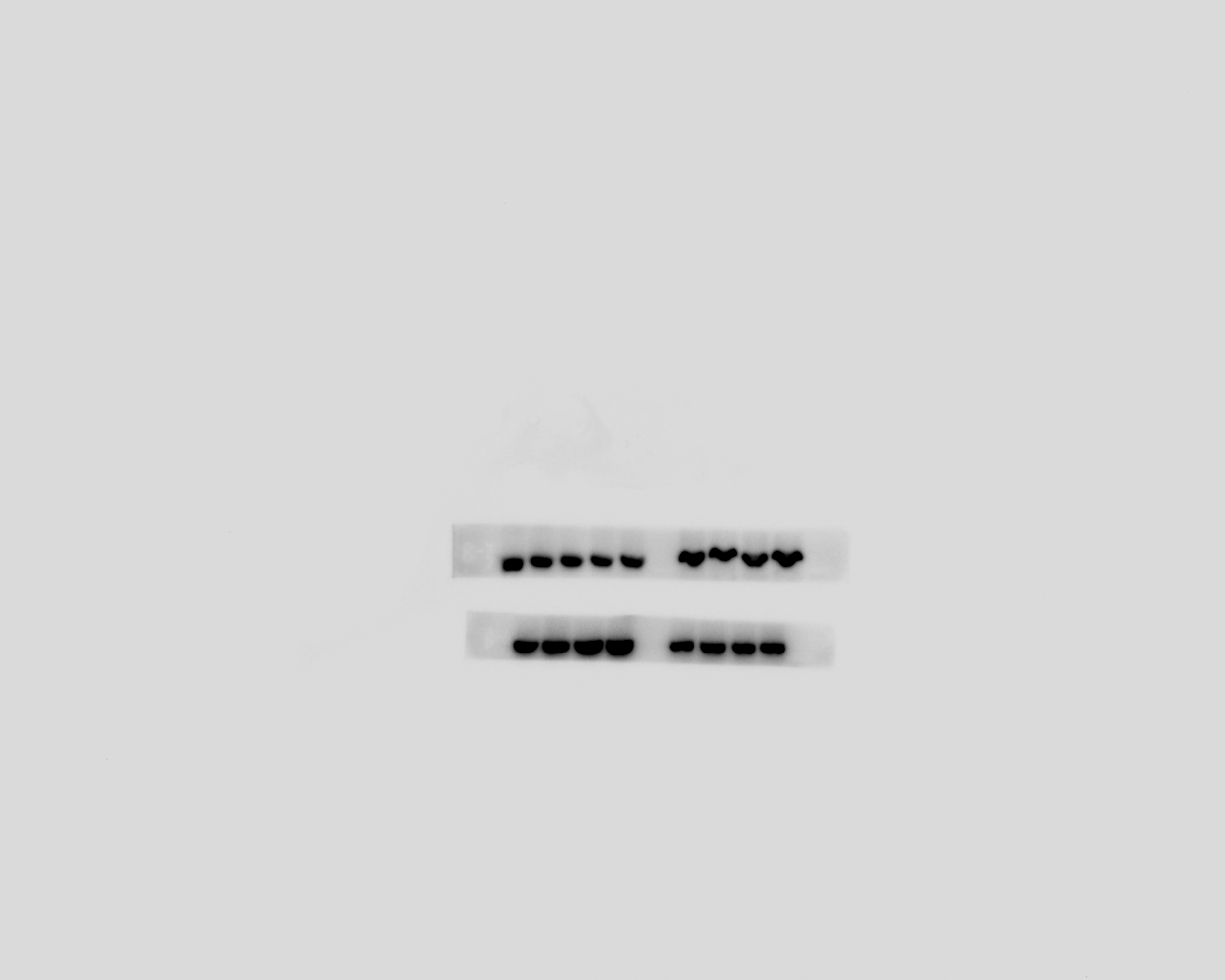

Supplement: Supplementary file 5 [file DataSheet8.zip › SAHA TSA and Bufexamac-P-gp and H3K9ac/TSA SAHA-P-gp SAHA-BU-H3K9/TSA and SAHA-P-gp/TSA and SAHA-a┬-actin.tif]

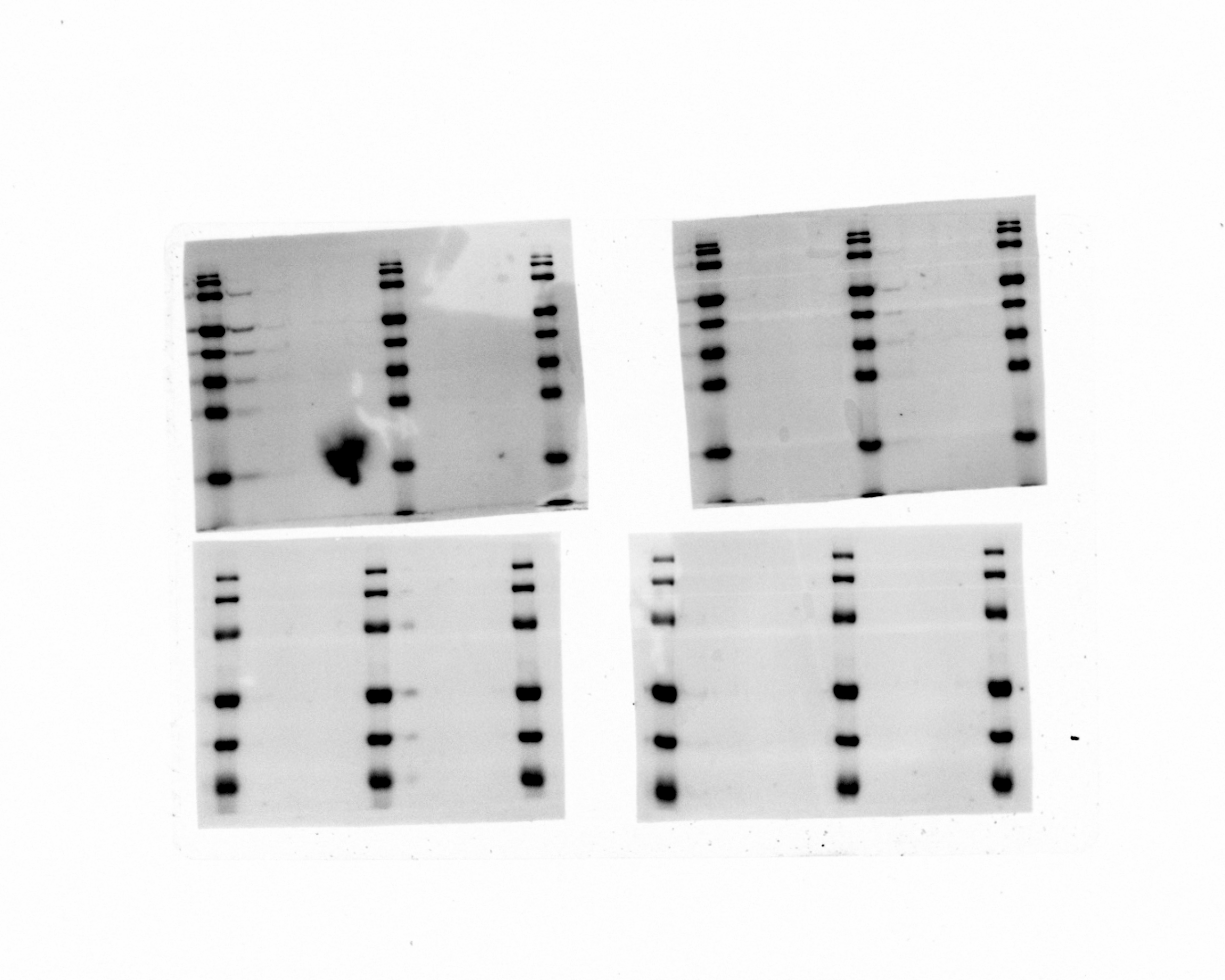

Supplement: Supplementary file 5 [file DataSheet8.zip › SAHA TSA and Bufexamac-P-gp and H3K9ac/TSA SAHA-P-gp SAHA-BU-H3K9/Whole Western blot membrane..tif]

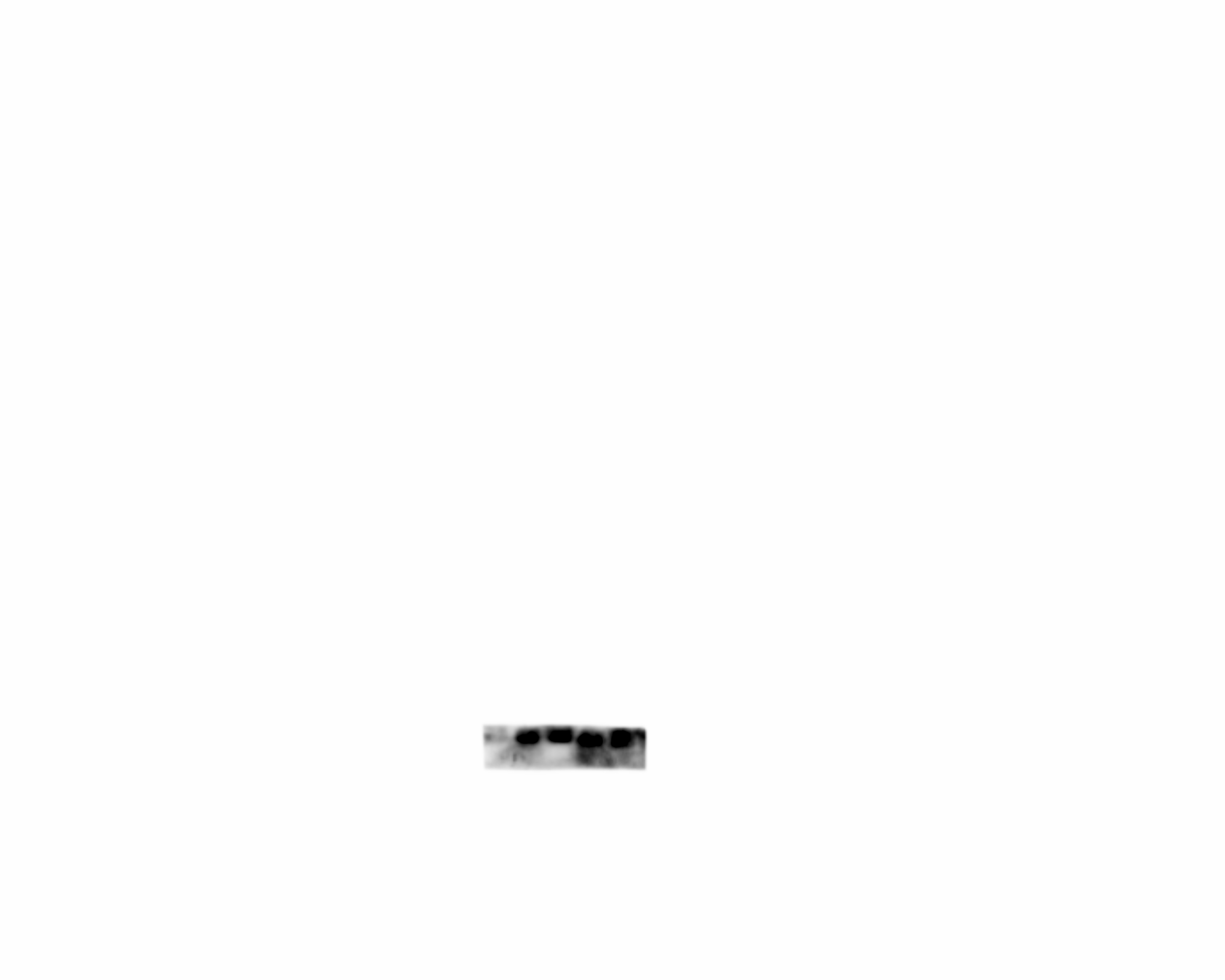

Supplement: Supplementary file 5 [file DataSheet8.zip › SAHA TSA and Bufexamac-P-gp and H3K9ac/TSA-H3K9ac/H3K9ac.tif]

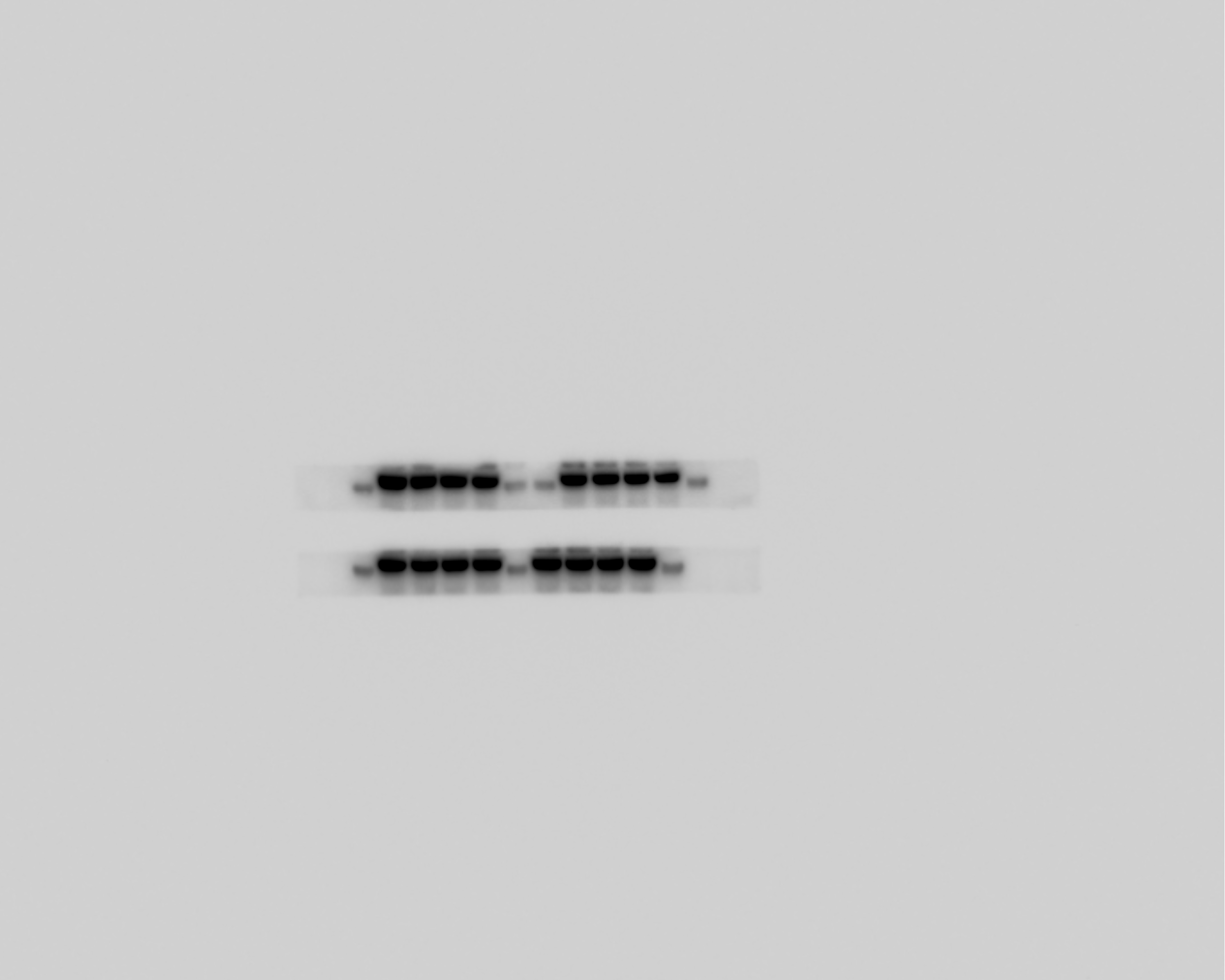

Supplement: Supplementary file 5 [file DataSheet8.zip › SAHA TSA and Bufexamac-P-gp and H3K9ac/TSA-H3K9ac/a┬-actin.tif]

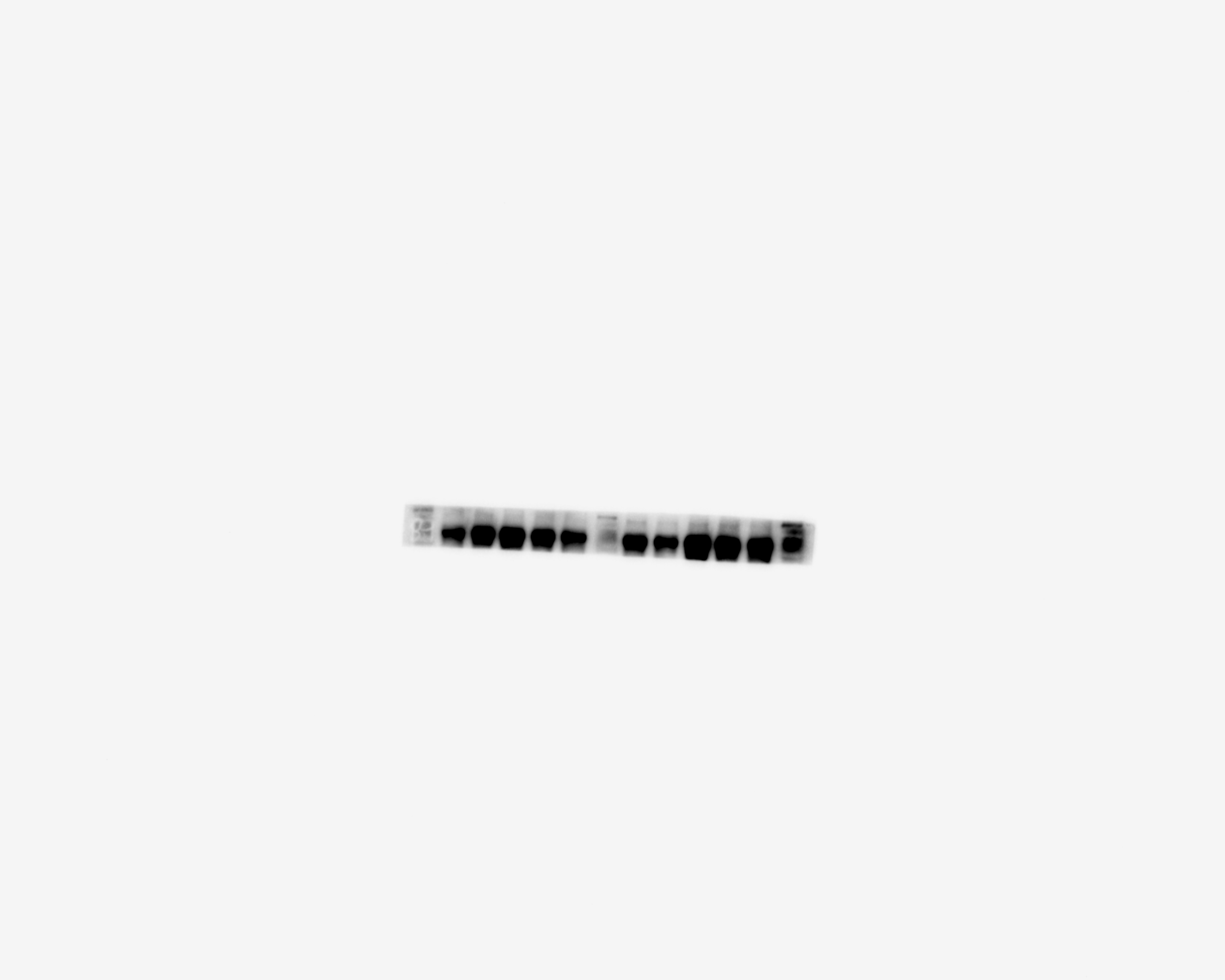

Supplement: Supplementary file 6 [file DataSheet9.zip › SAHA-SP1/SP1.tif]

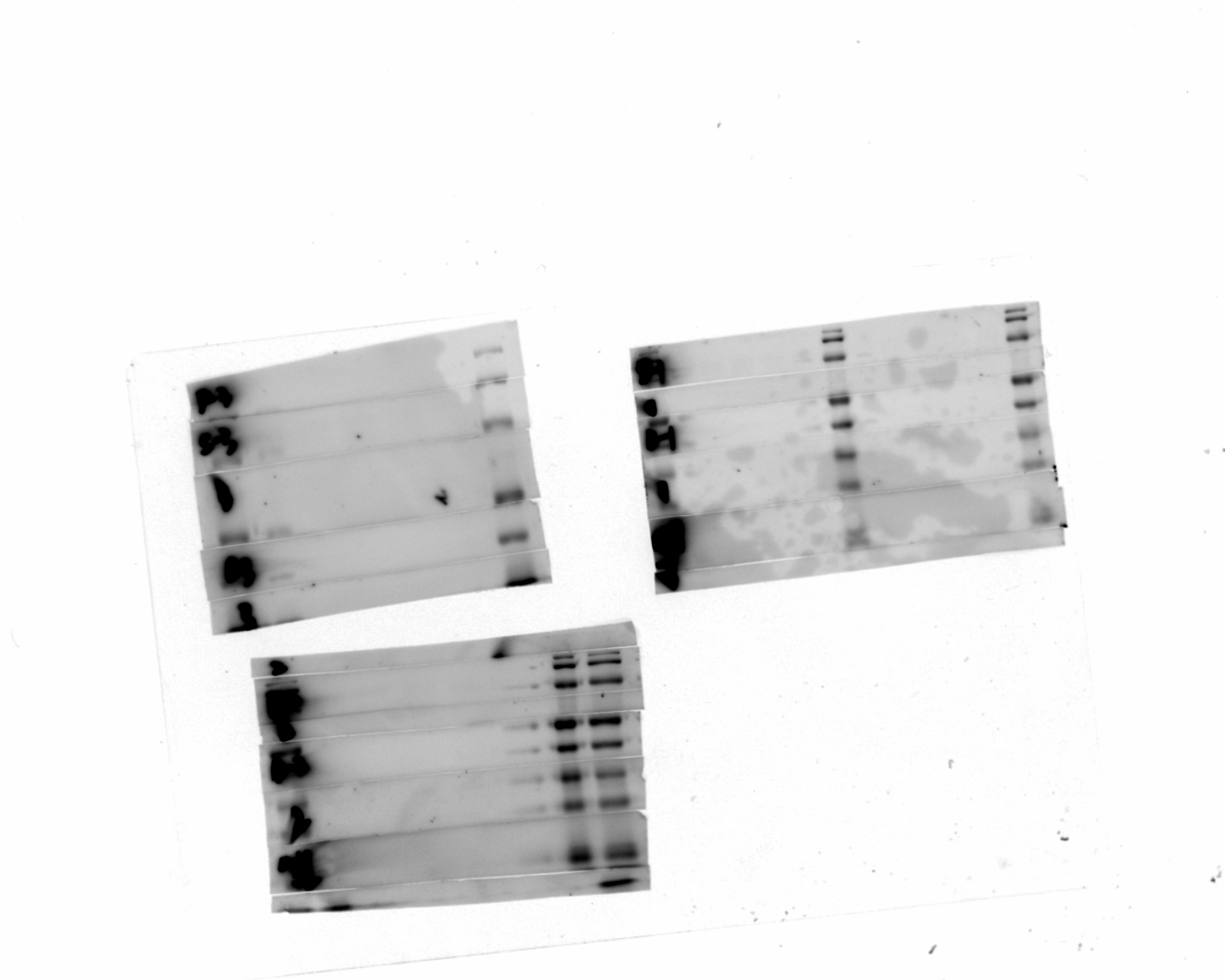

Supplement: Supplementary file 6 [file DataSheet9.zip › SAHA-SP1/Western blot membrane cutting..tif]

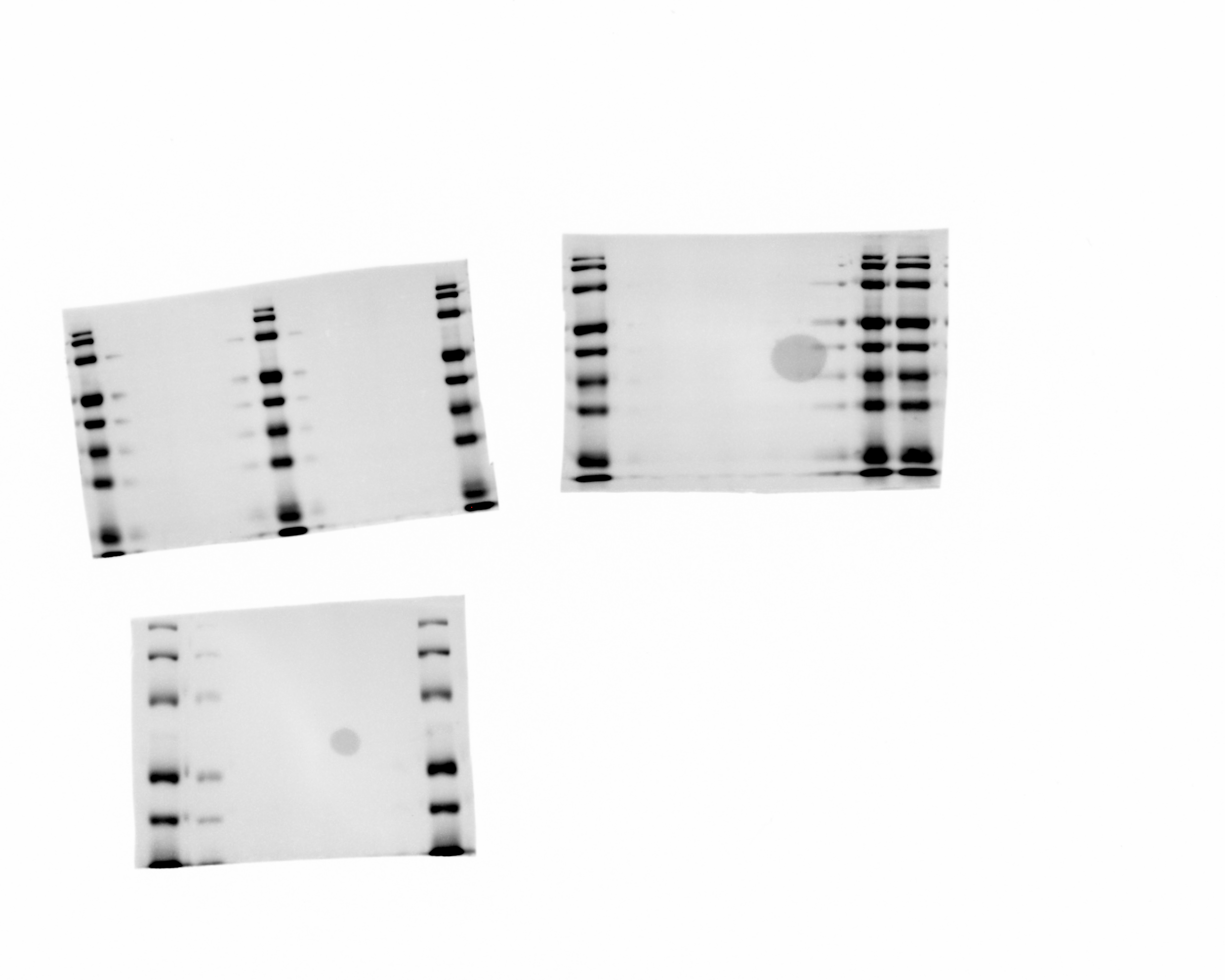

Supplement: Supplementary file 6 [file DataSheet9.zip › SAHA-SP1/Whole Western blot membrane..tif]

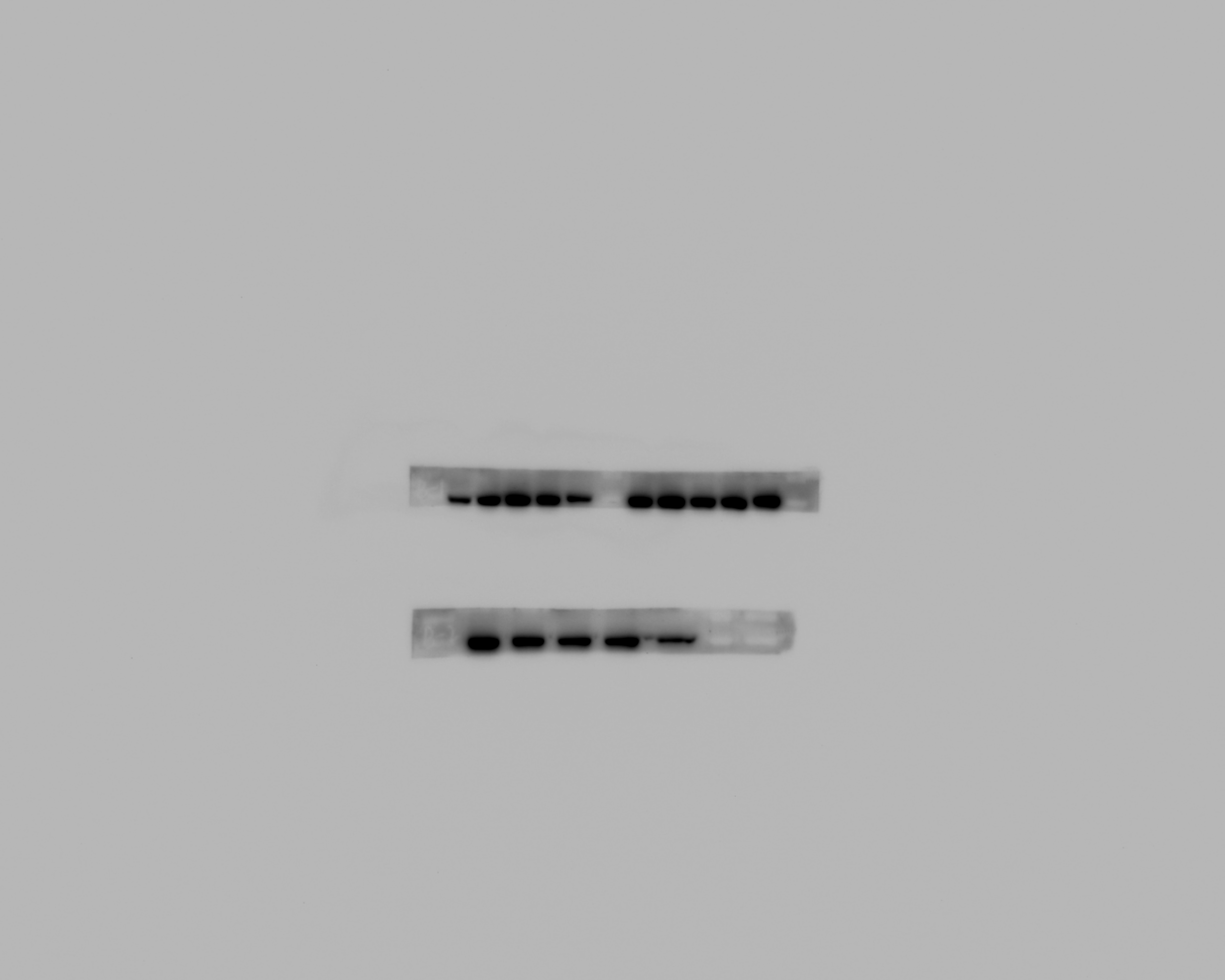

Supplement: Supplementary file 6 [file DataSheet9.zip › SAHA-SP1/a┬-actin.tif]

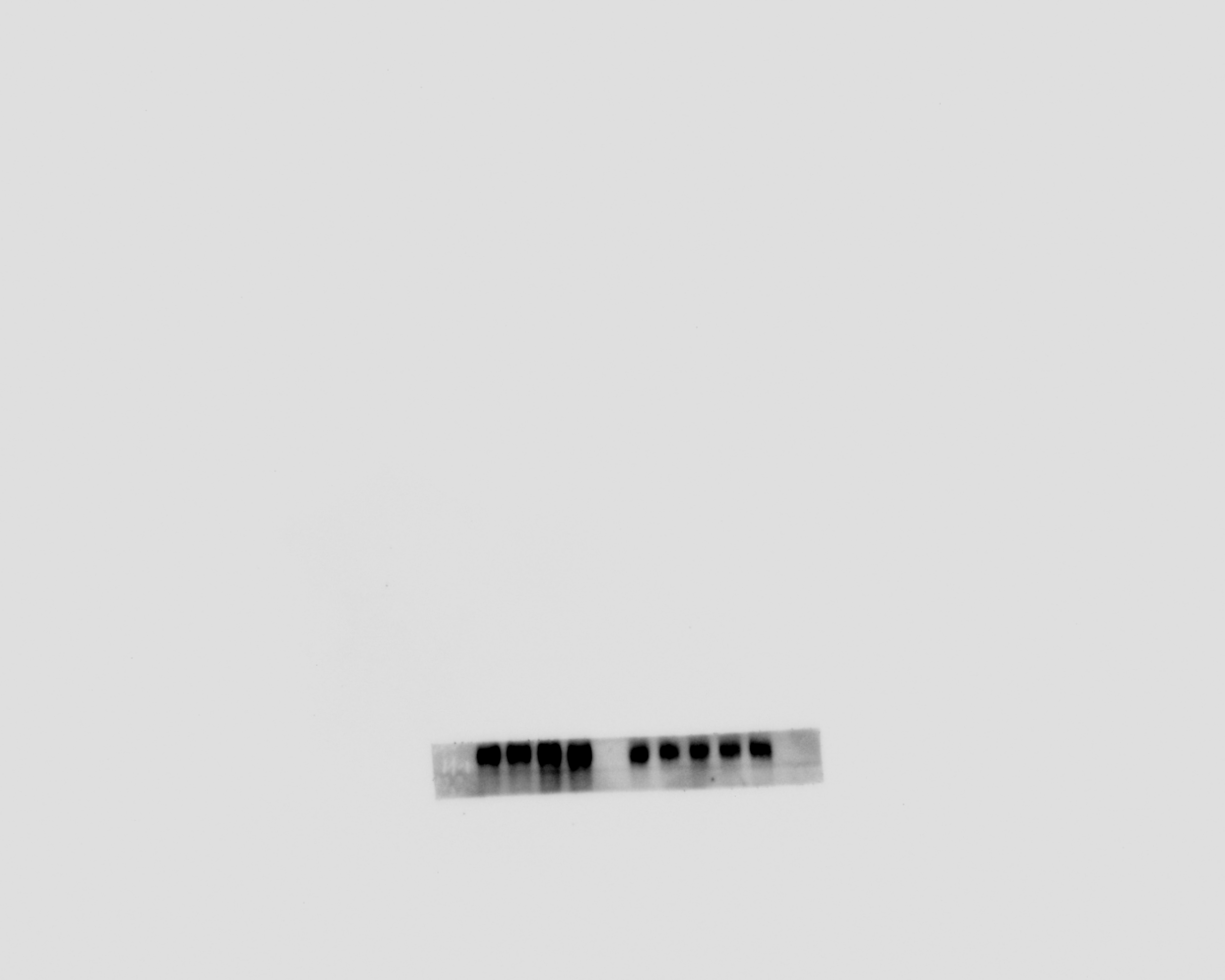

Supplement: Supplementary file 7 [file DataSheet4.zip › Normoxia vs Hypoxia-HDAC5/HDAC5.tif]

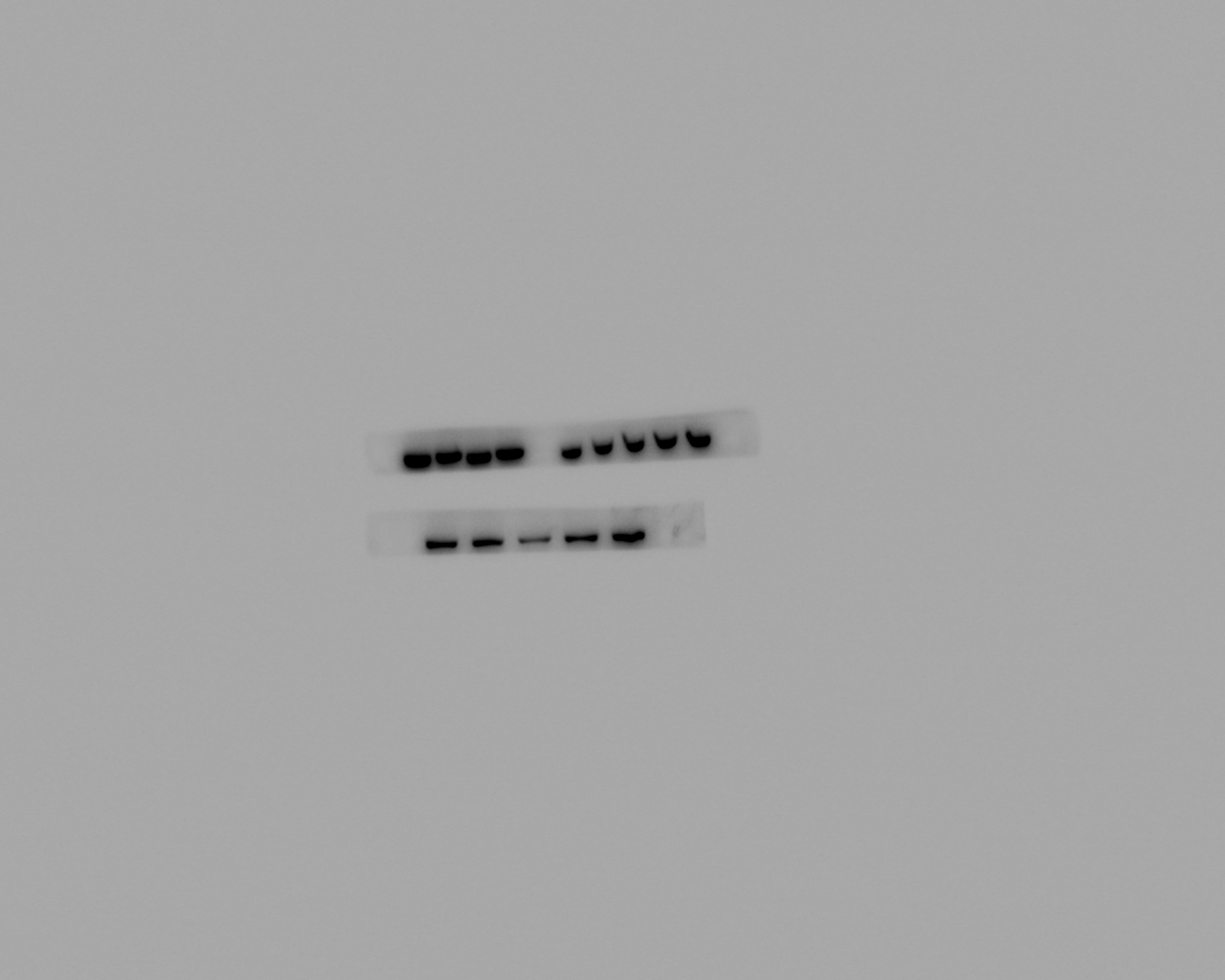

Supplement: Supplementary file 7 [file DataSheet4.zip › Normoxia vs Hypoxia-HDAC5/a┬-actin.tif]

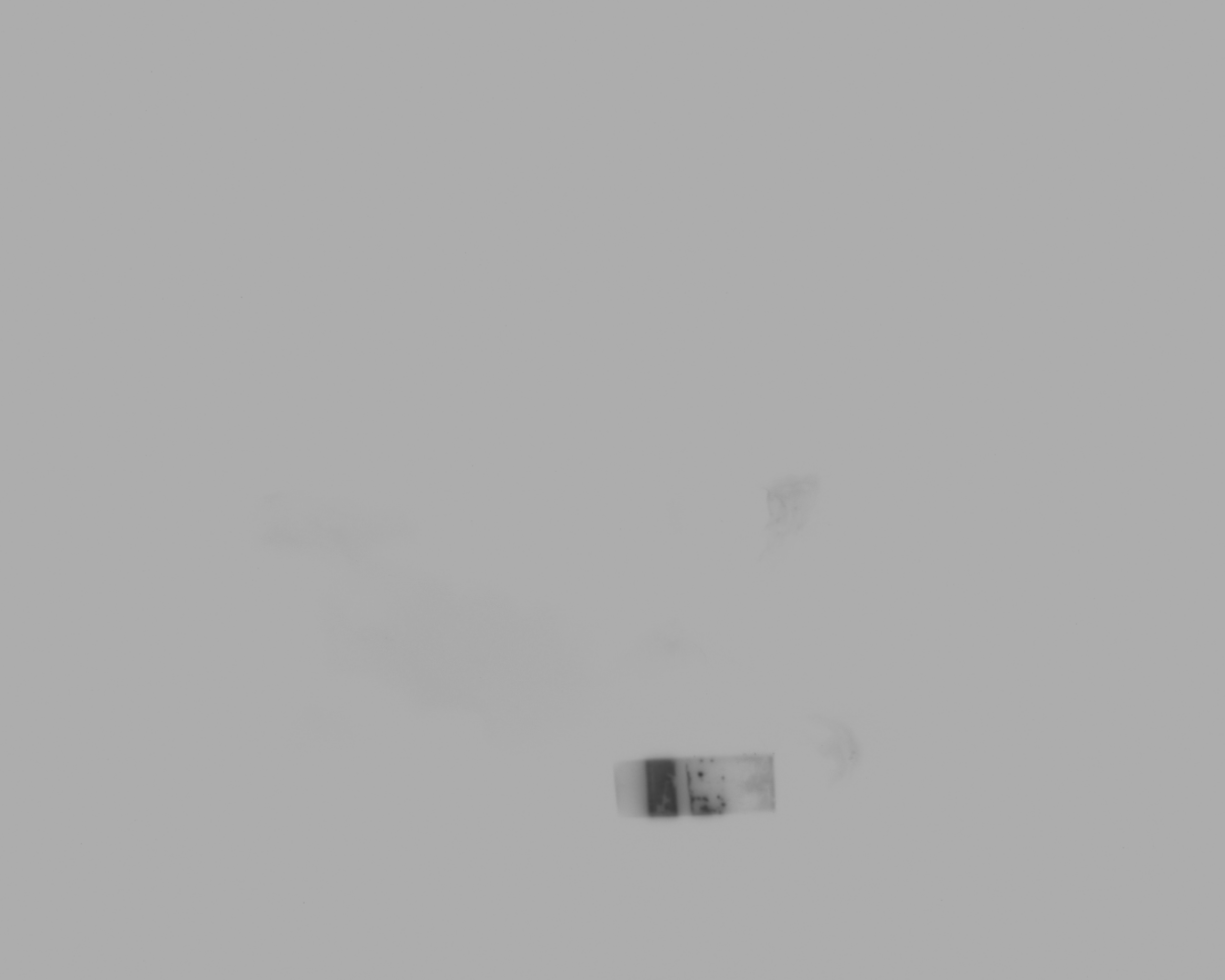

Supplement: Supplementary file 8 [file DataSheet1.zip › CO-IP/endogenous immunoprecipitation/IgG/HDAC5.tif]

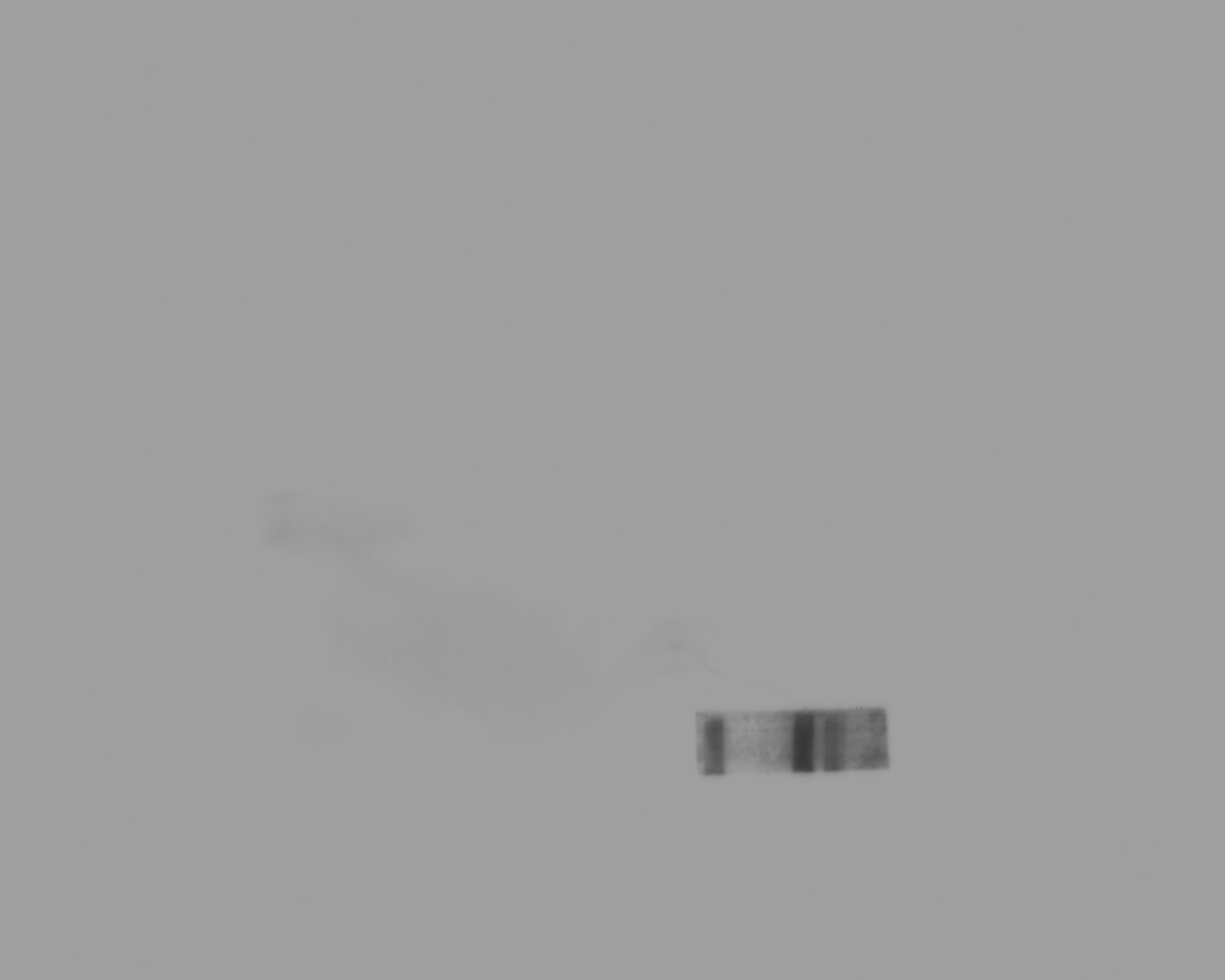

Supplement: Supplementary file 8 [file DataSheet1.zip › CO-IP/endogenous immunoprecipitation/IgG/P-gp.tif]

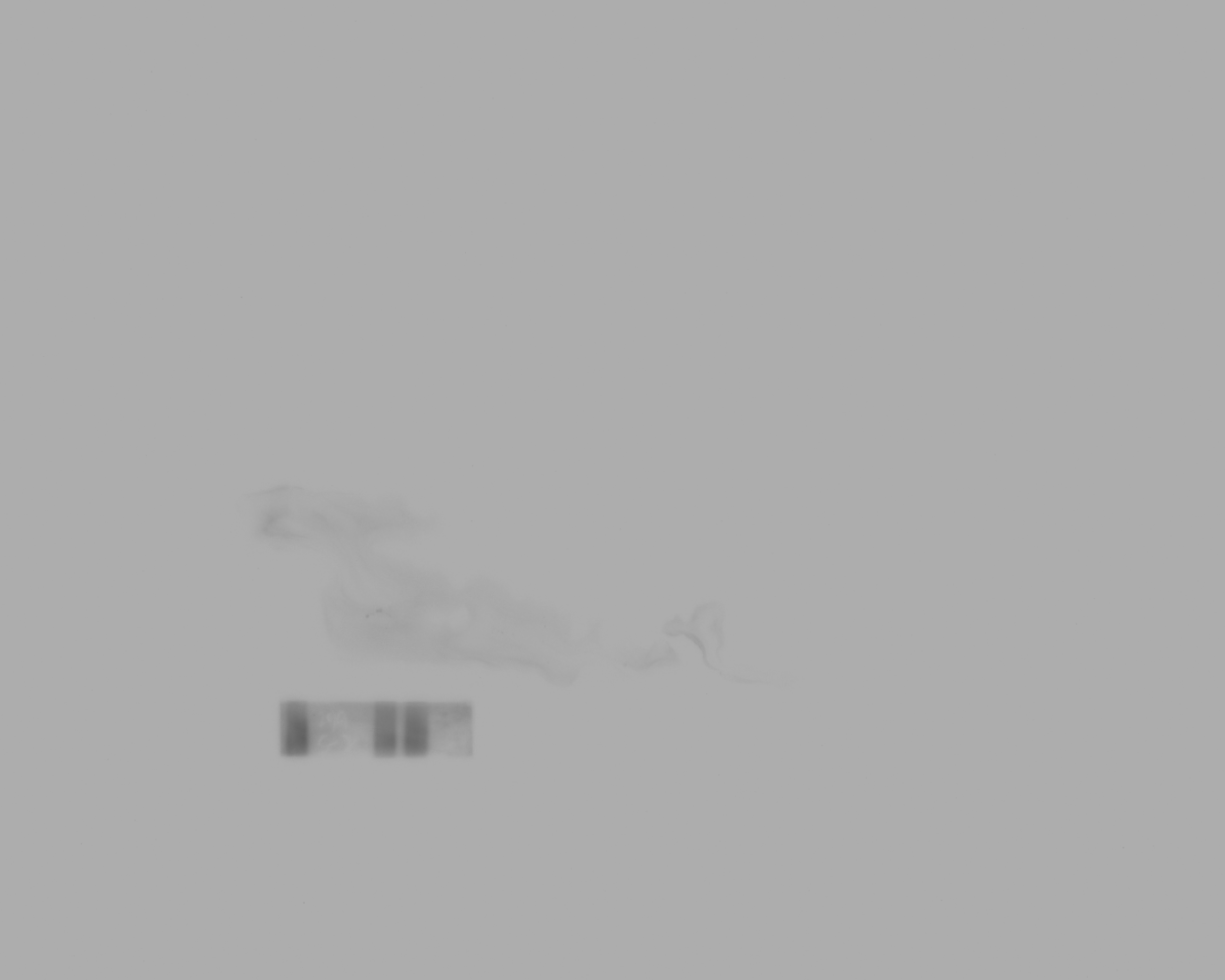

Supplement: Supplementary file 8 [file DataSheet1.zip › CO-IP/endogenous immunoprecipitation/IgG/SP1.tif]

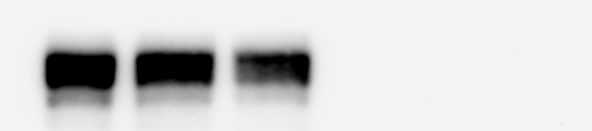

Supplement: Supplementary file 8 [file DataSheet1.zip › CO-IP/endogenous immunoprecipitation/input/HDAC5.tif]

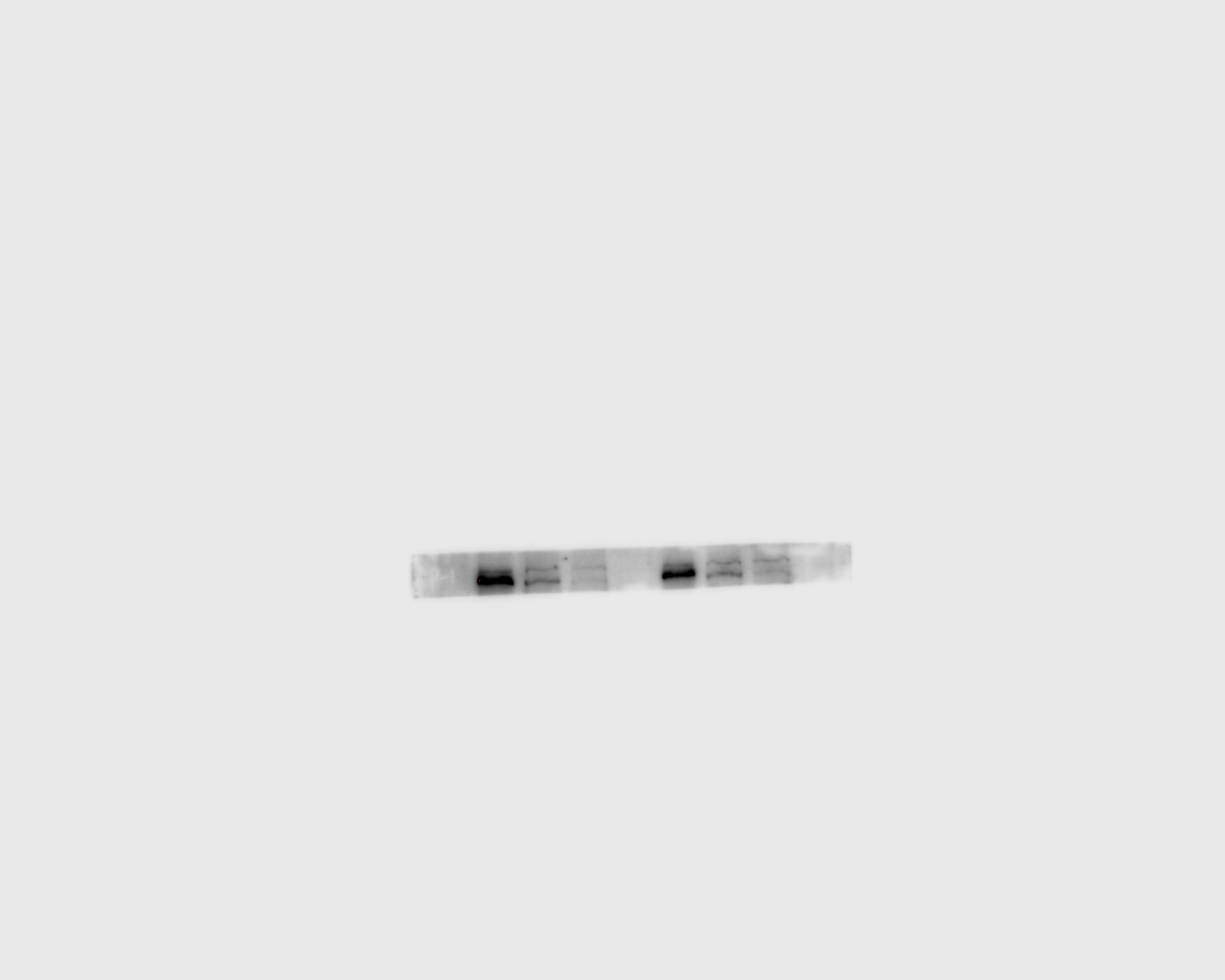

Supplement: Supplementary file 8 [file DataSheet1.zip › CO-IP/endogenous immunoprecipitation/input/P-gp.tif]

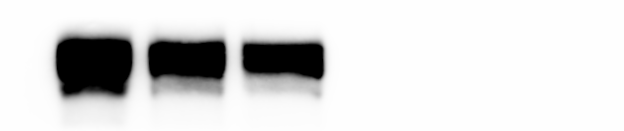

Supplement: Supplementary file 8 [file DataSheet1.zip › CO-IP/endogenous immunoprecipitation/input/SP1.tif]

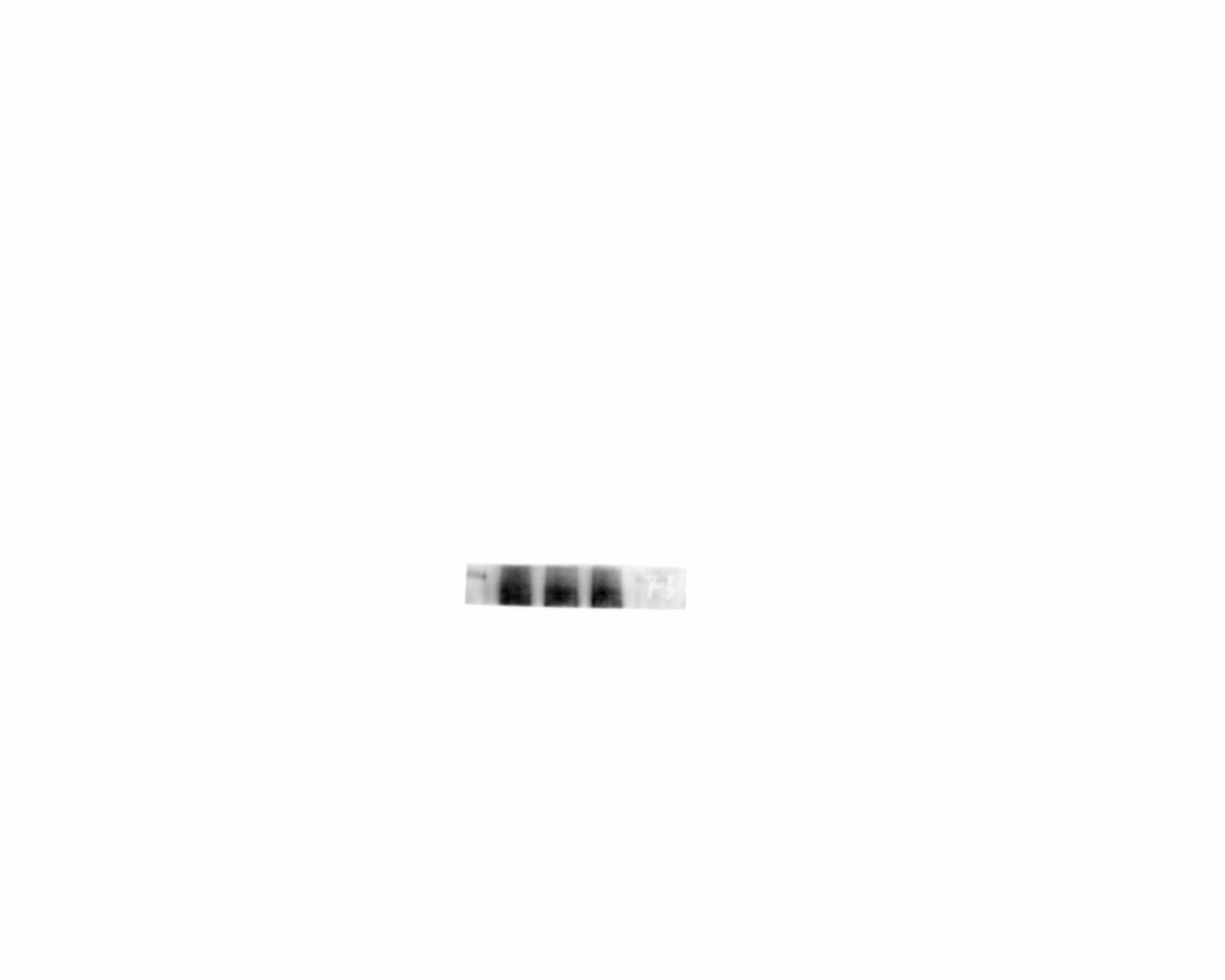

Supplement: Supplementary file 8 [file DataSheet1.zip › CO-IP/endogenous immunoprecipitation/IP HDAC5 +IB P-gp+IB SP1/P-gp.tif]

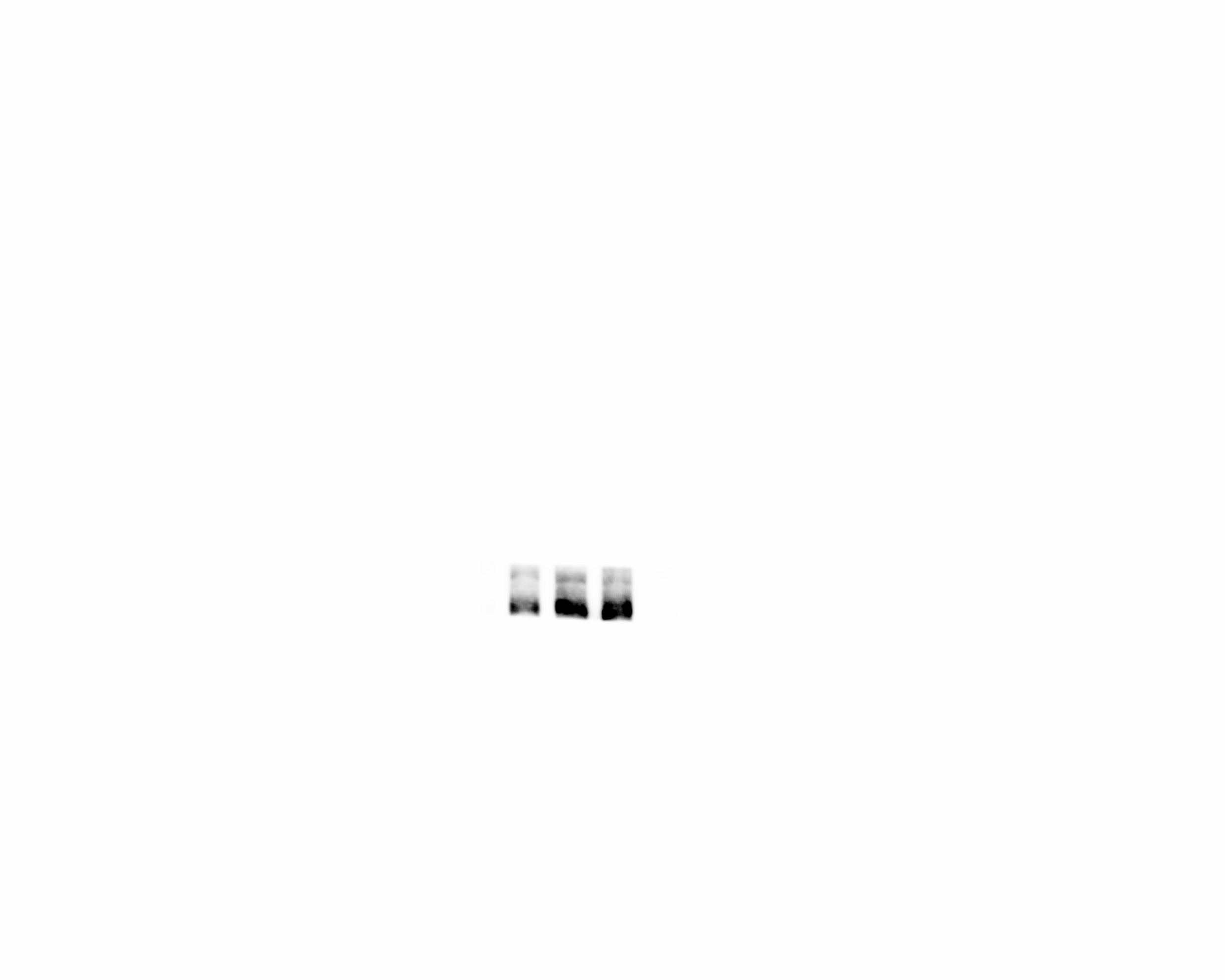

Supplement: Supplementary file 8 [file DataSheet1.zip › CO-IP/endogenous immunoprecipitation/IP HDAC5 +IB P-gp+IB SP1/SP1.tif]

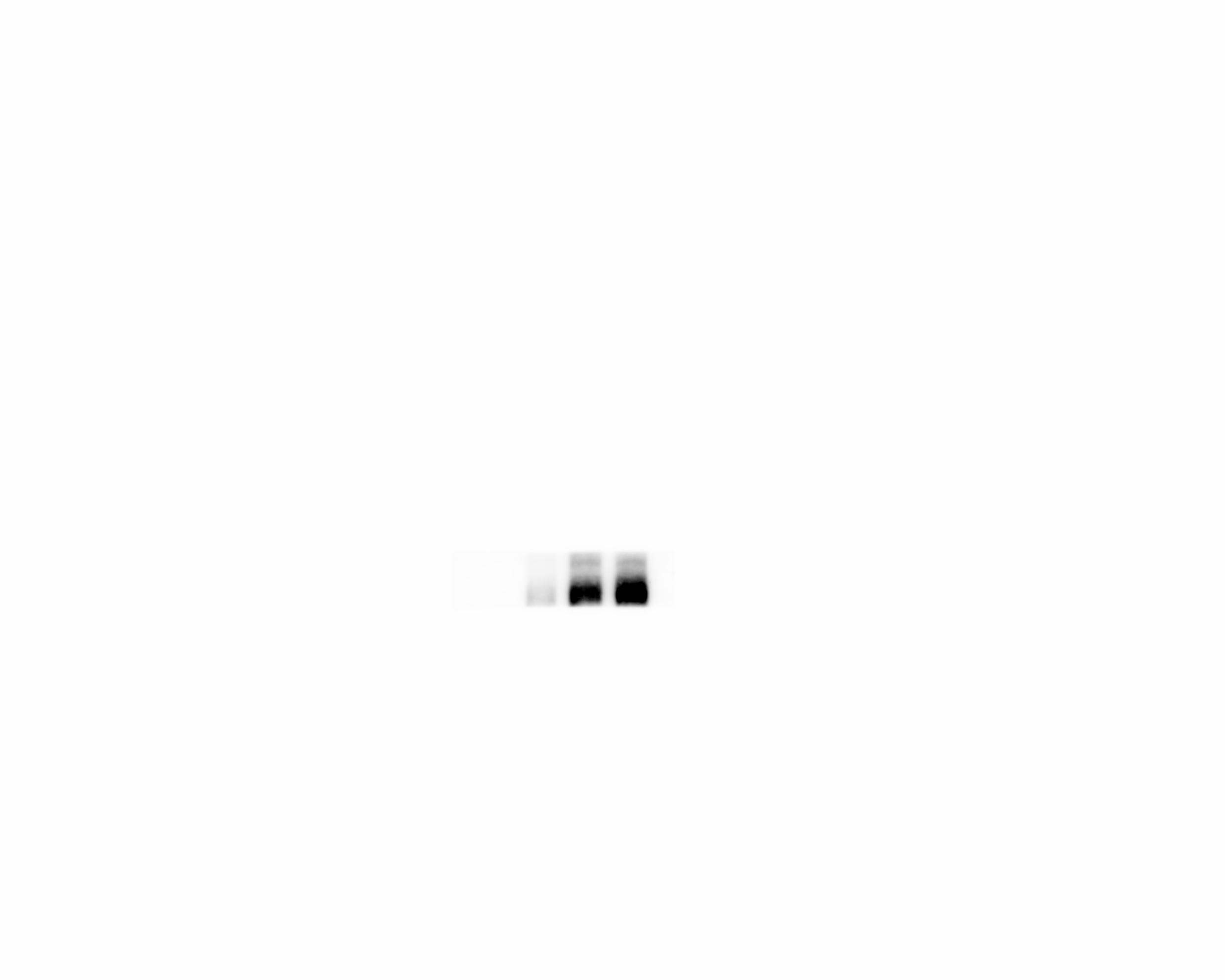

Supplement: Supplementary file 8 [file DataSheet1.zip › CO-IP/endogenous immunoprecipitation/IP P-gp +IB SP1+IB HDAC5/HDAC5.tif]

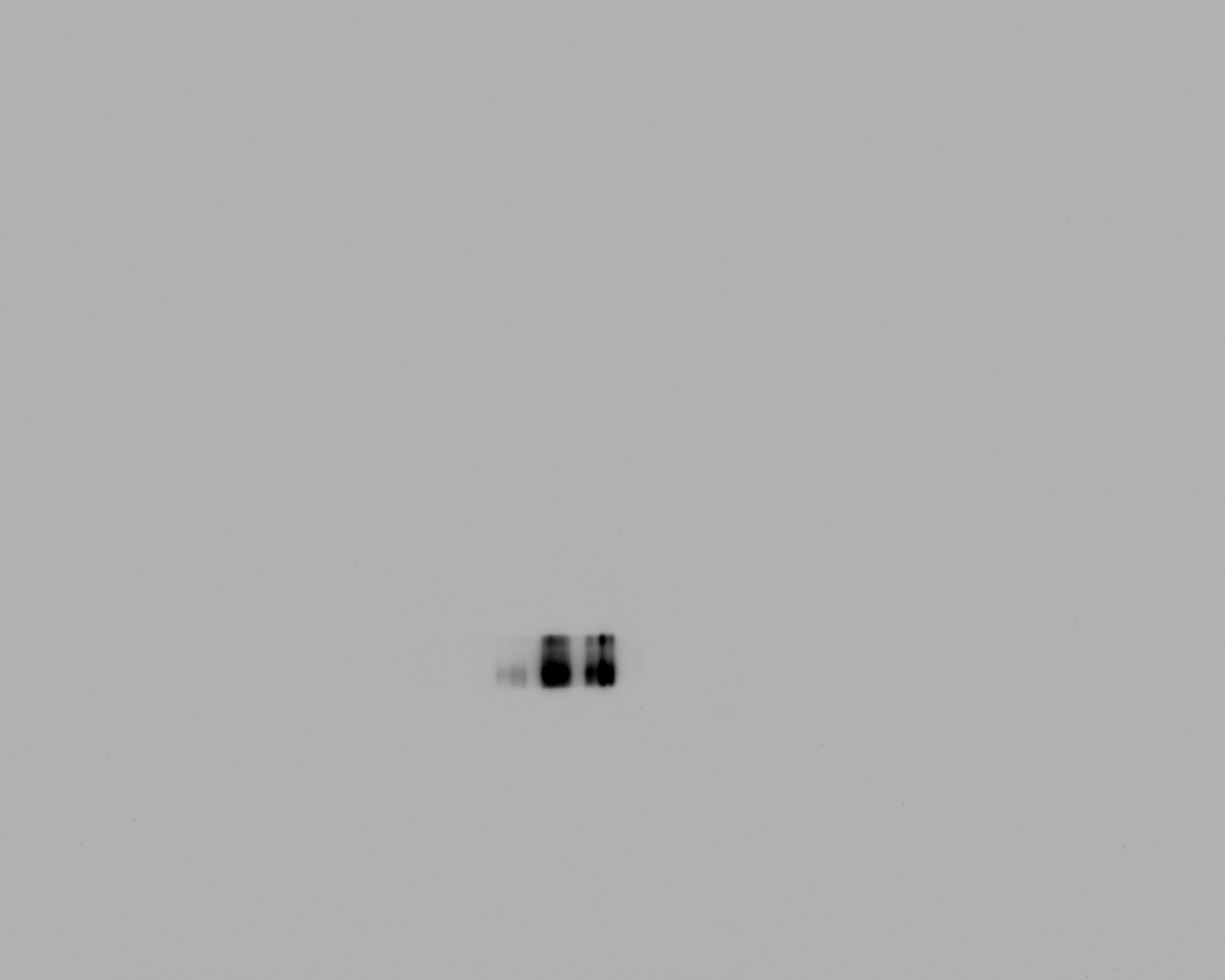

Supplement: Supplementary file 8 [file DataSheet1.zip › CO-IP/endogenous immunoprecipitation/IP P-gp +IB SP1+IB HDAC5/SP1.tif]

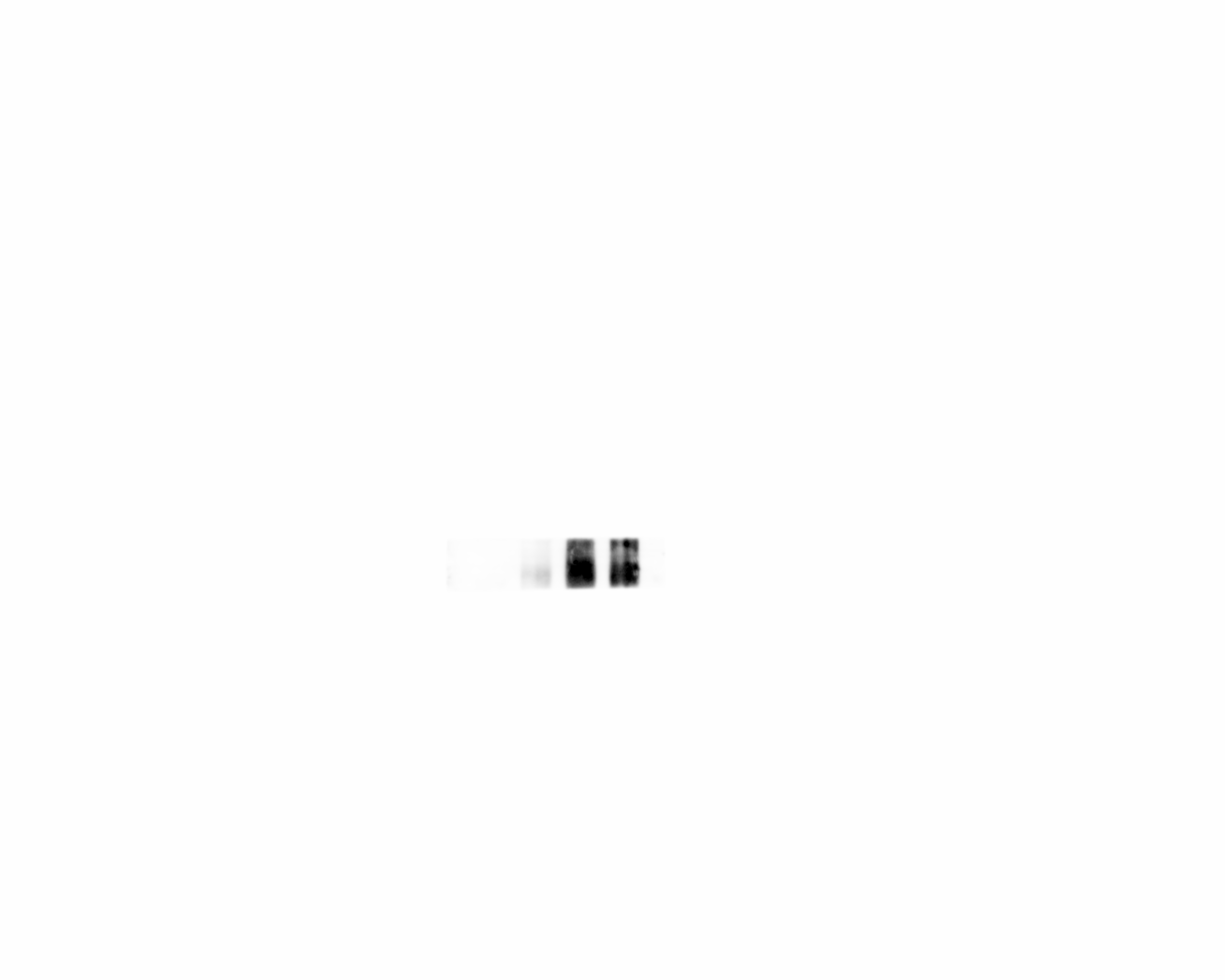

Supplement: Supplementary file 8 [file DataSheet1.zip › CO-IP/endogenous immunoprecipitation/IP SP1 +IB P-gp+IB HDAC5/HDAC5.tif]

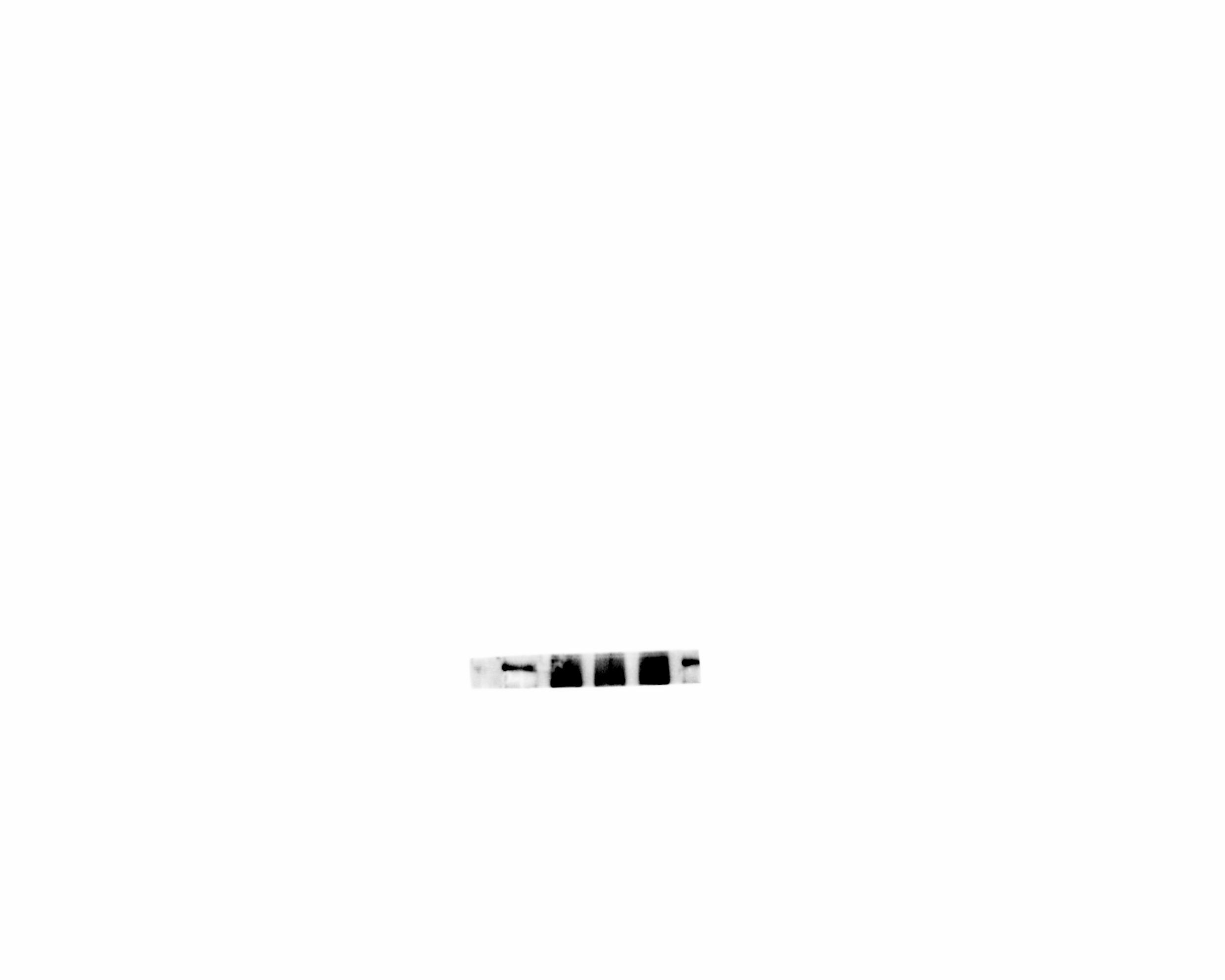

Supplement: Supplementary file 8 [file DataSheet1.zip › CO-IP/endogenous immunoprecipitation/IP SP1 +IB P-gp+IB HDAC5/P-gp.tif]

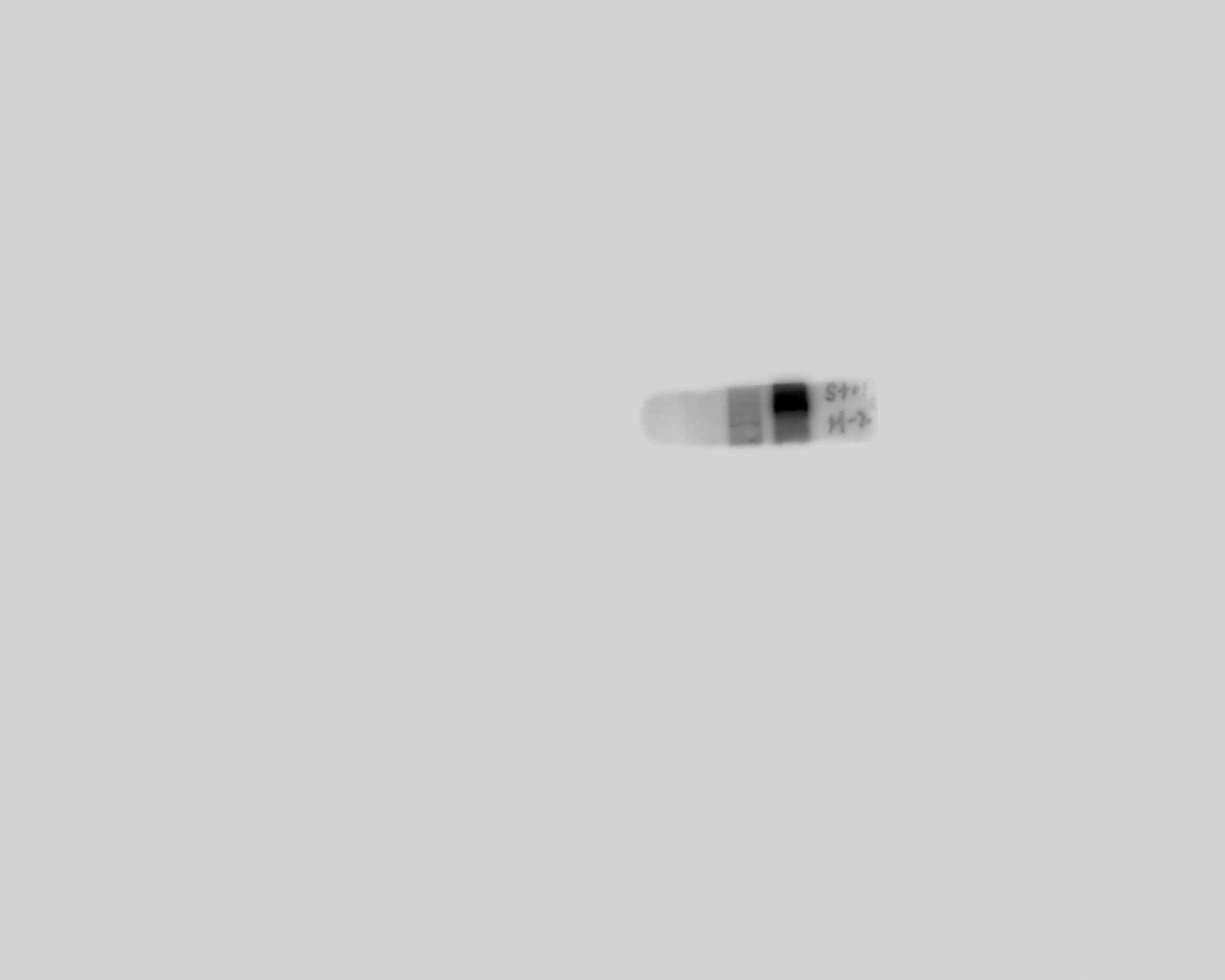

Supplement: Supplementary file 8 [file DataSheet1.zip › CO-IP/exogenous immunoprecipitation/cotransfection/IP HDAC5-Myc+ IB SP1-Flag/HDAC5-Myc.tif]

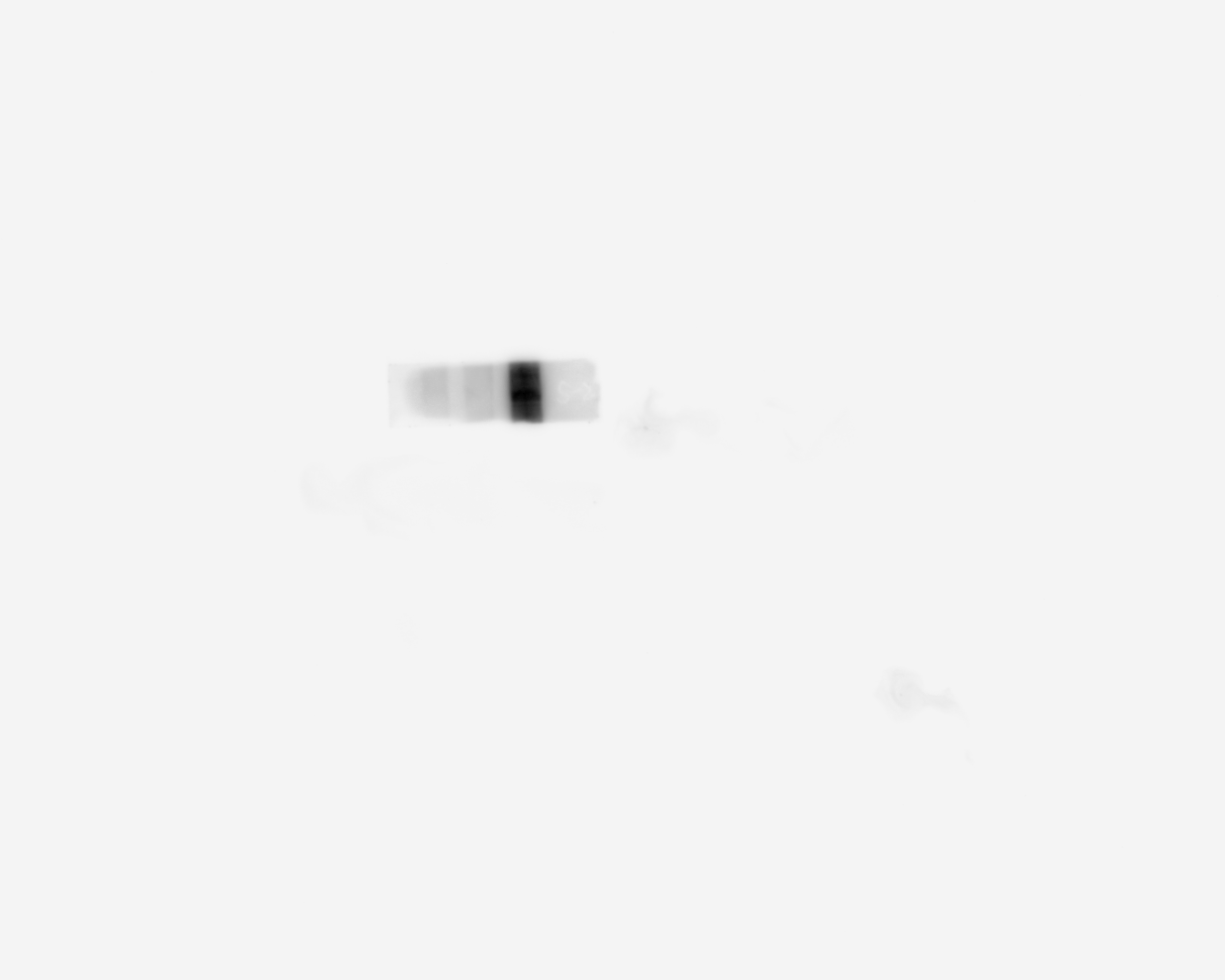

Supplement: Supplementary file 8 [file DataSheet1.zip › CO-IP/exogenous immunoprecipitation/cotransfection/IP HDAC5-Myc+ IB SP1-Flag/SP1-Flag.tif]

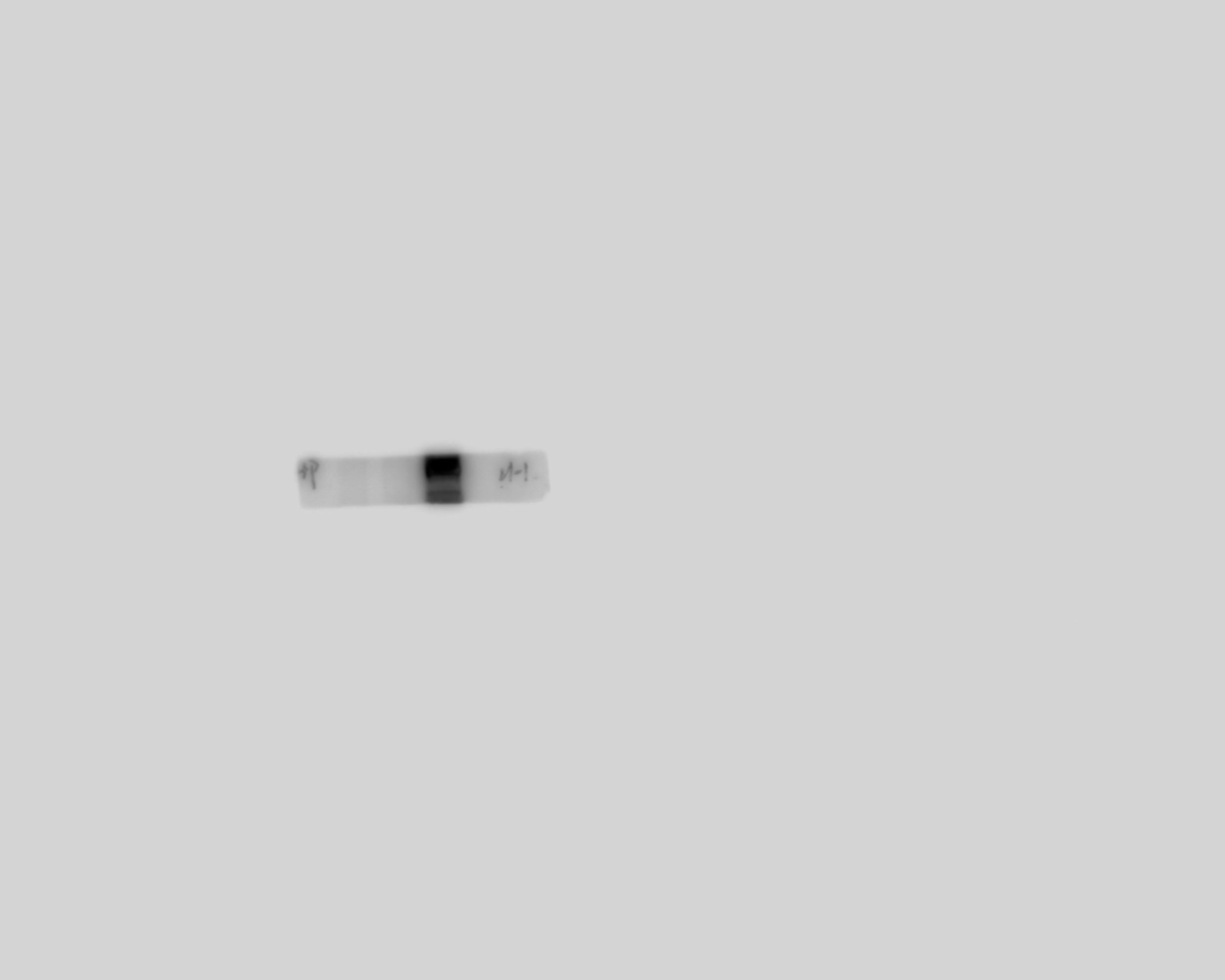

Supplement: Supplementary file 8 [file DataSheet1.zip › CO-IP/exogenous immunoprecipitation/cotransfection/IP HDAC5-Myc+IB P-gp-HA/HDAC5-Myc.tif]

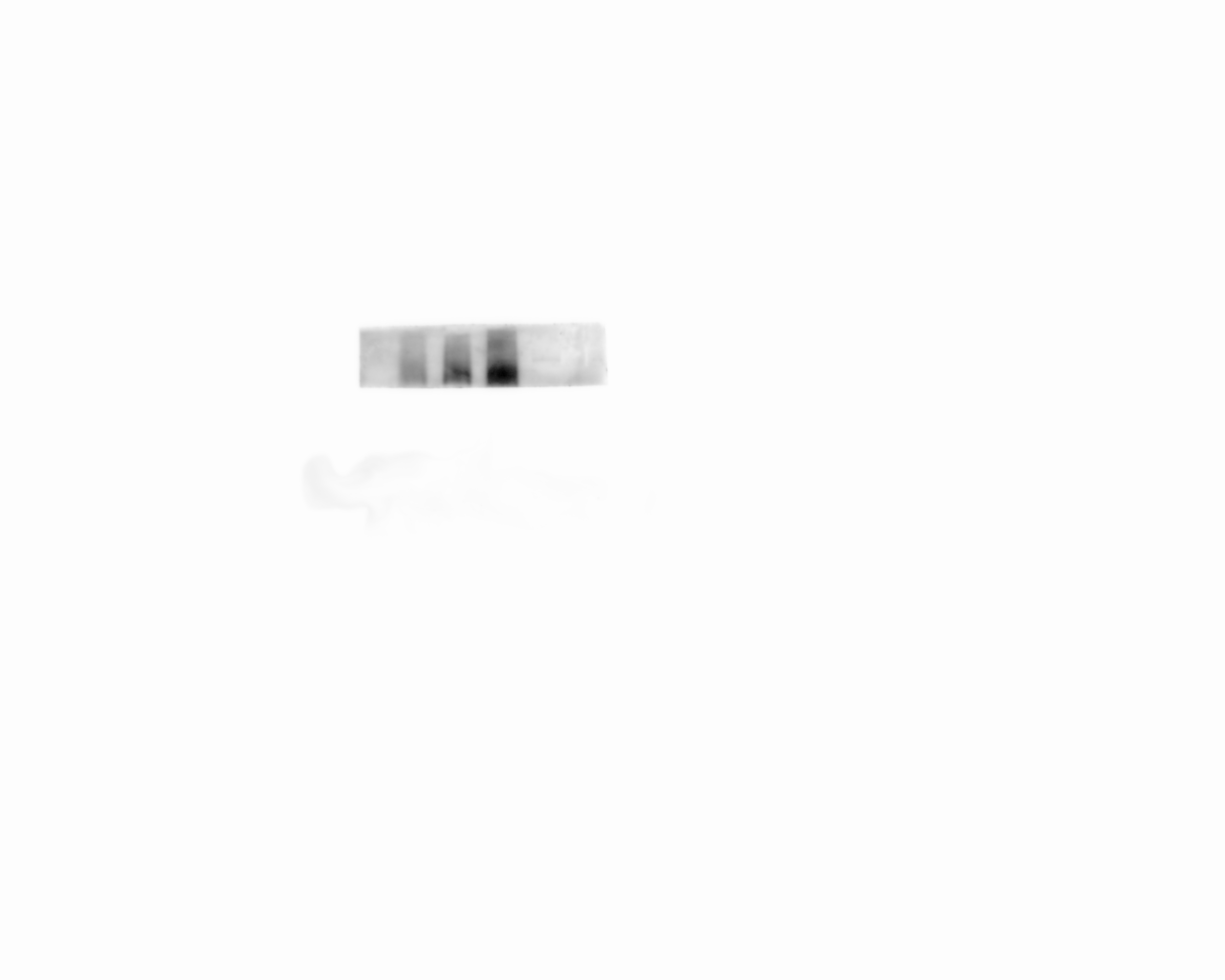

Supplement: Supplementary file 8 [file DataSheet1.zip › CO-IP/exogenous immunoprecipitation/cotransfection/IP HDAC5-Myc+IB P-gp-HA/P-gp-HA.tif]

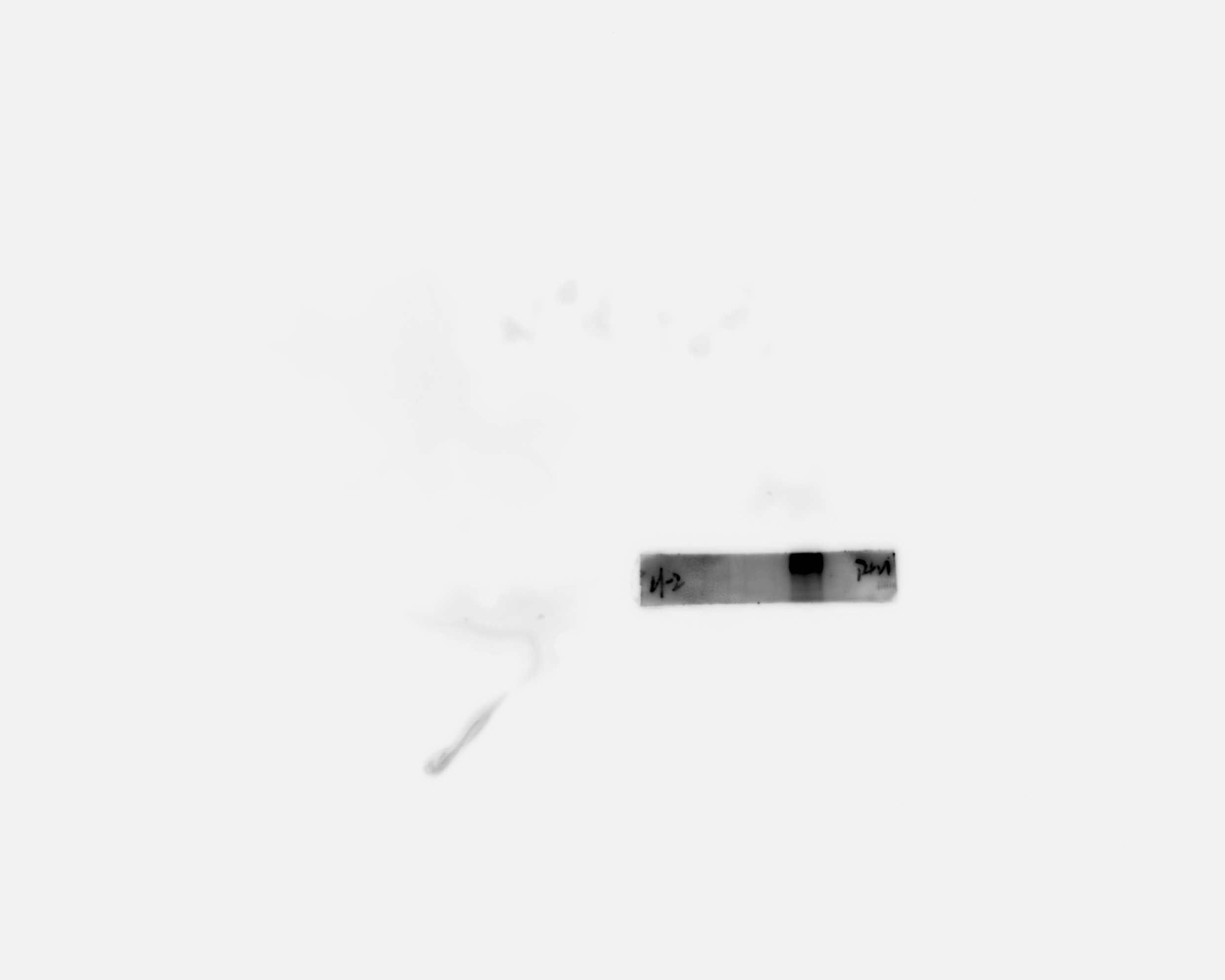

Supplement: Supplementary file 8 [file DataSheet1.zip › CO-IP/exogenous immunoprecipitation/cotransfection/IP P-gp-HA+IB HDAC5-Myc/HDAC5-Myc.tif]

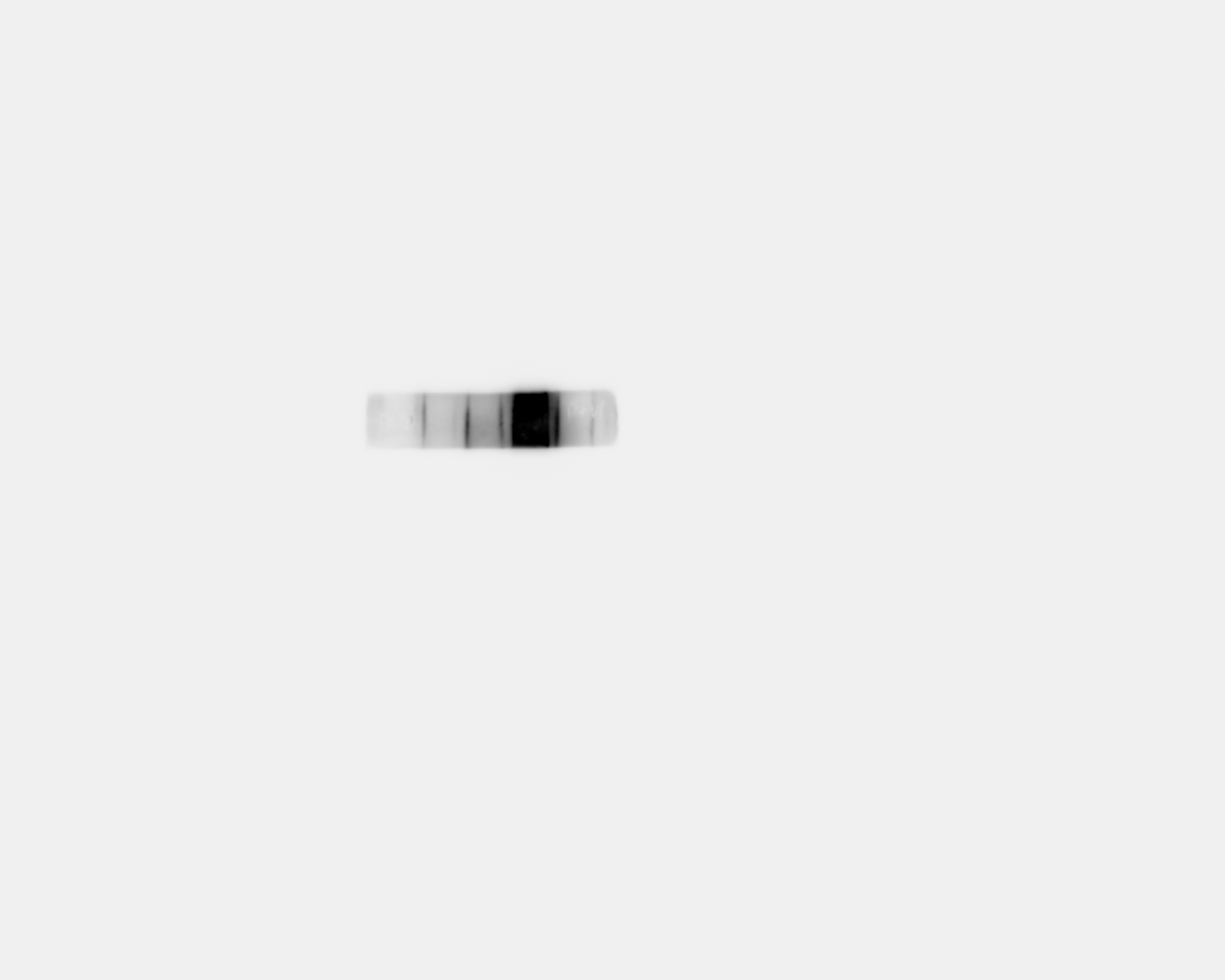

Supplement: Supplementary file 8 [file DataSheet1.zip › CO-IP/exogenous immunoprecipitation/cotransfection/IP P-gp-HA+IB HDAC5-Myc/P-gp-HA.tif]

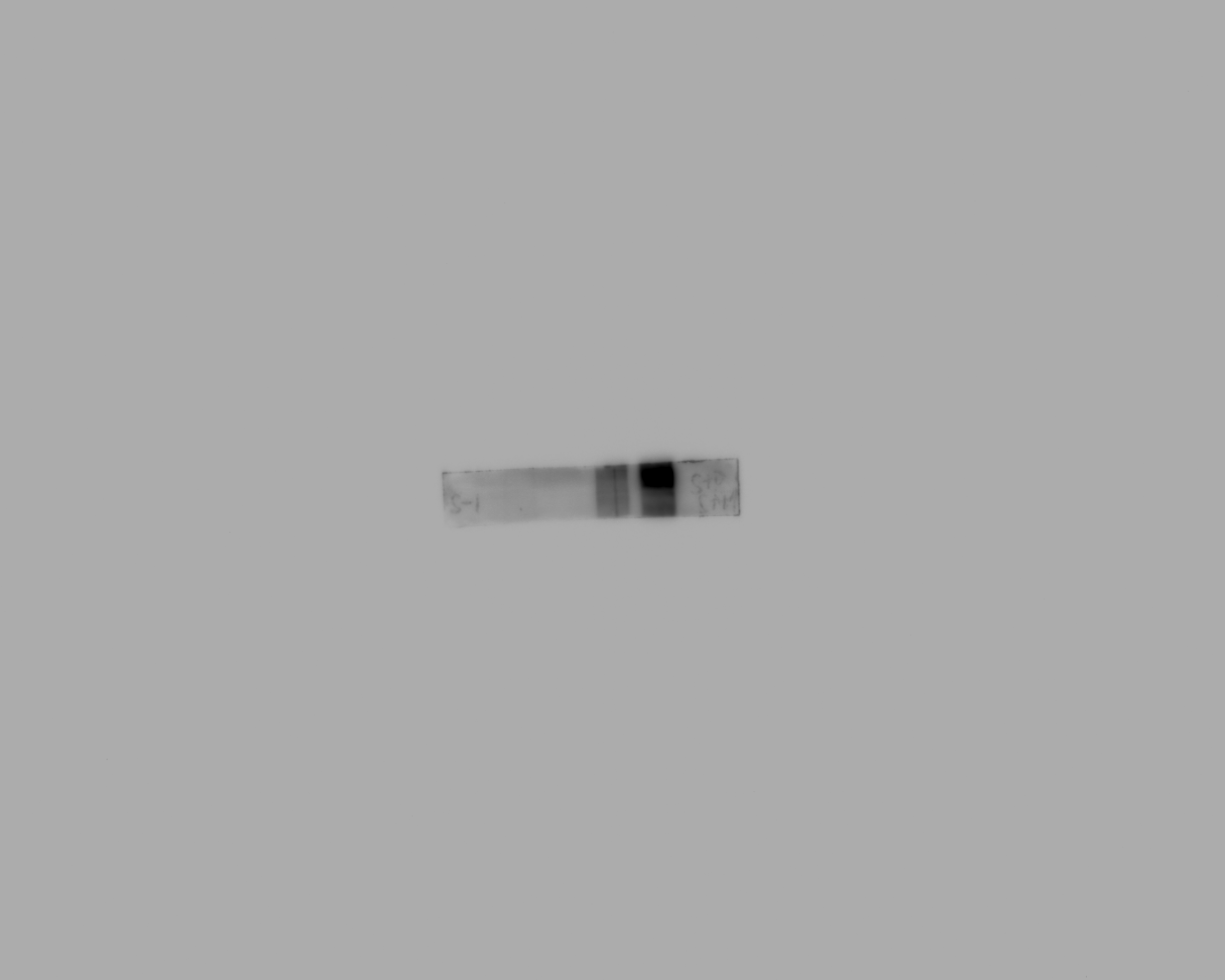

Supplement: Supplementary file 8 [file DataSheet1.zip › CO-IP/exogenous immunoprecipitation/cotransfection/IP SP1-Flag+IB HDAC5-Myc+ IP-P-gp-HA+ IB SP1-Flag/HDAC5-Myc.tif]

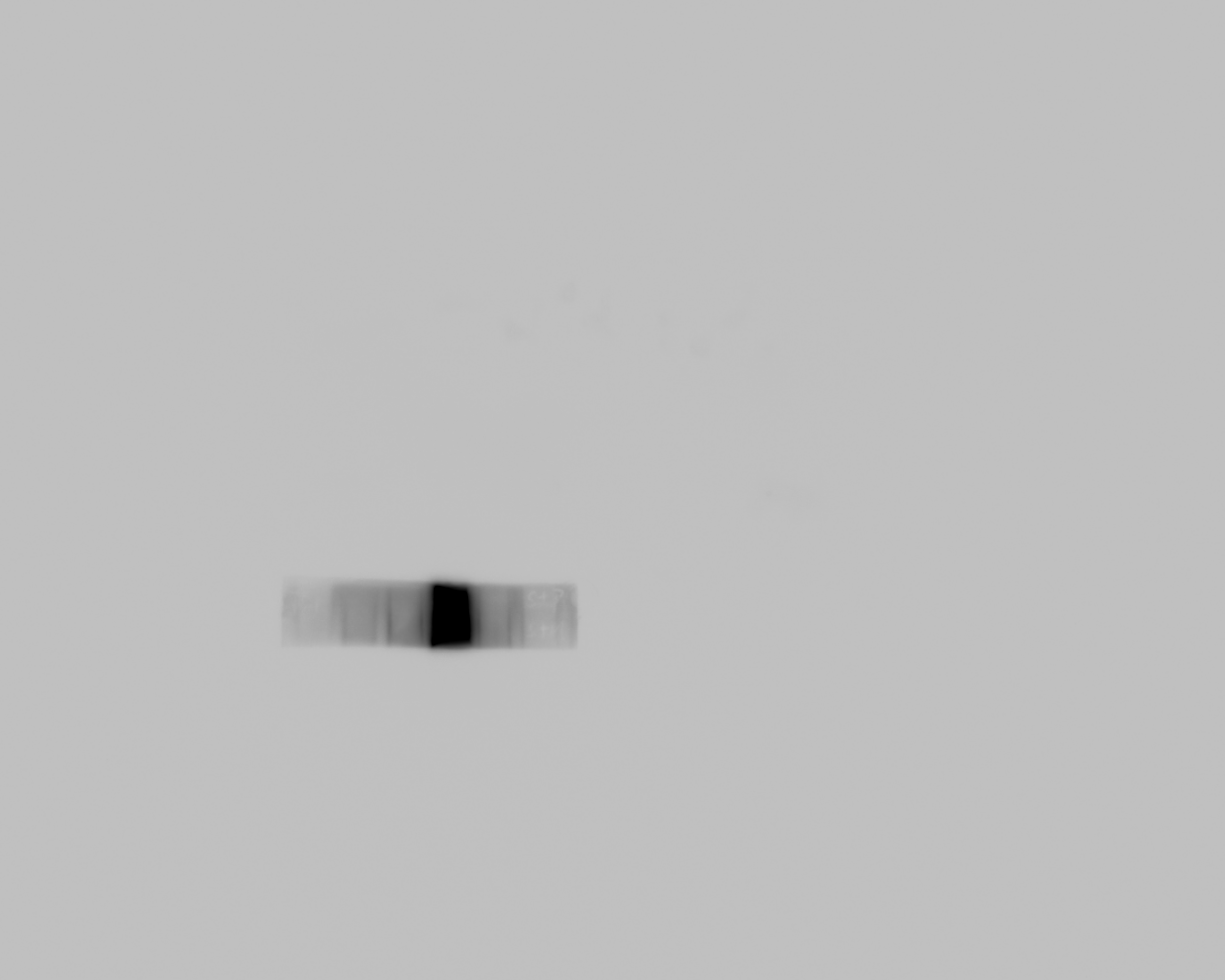

Supplement: Supplementary file 8 [file DataSheet1.zip › CO-IP/exogenous immunoprecipitation/cotransfection/IP SP1-Flag+IB HDAC5-Myc+ IP-P-gp-HA+ IB SP1-Flag/P-gp-HA.tif]

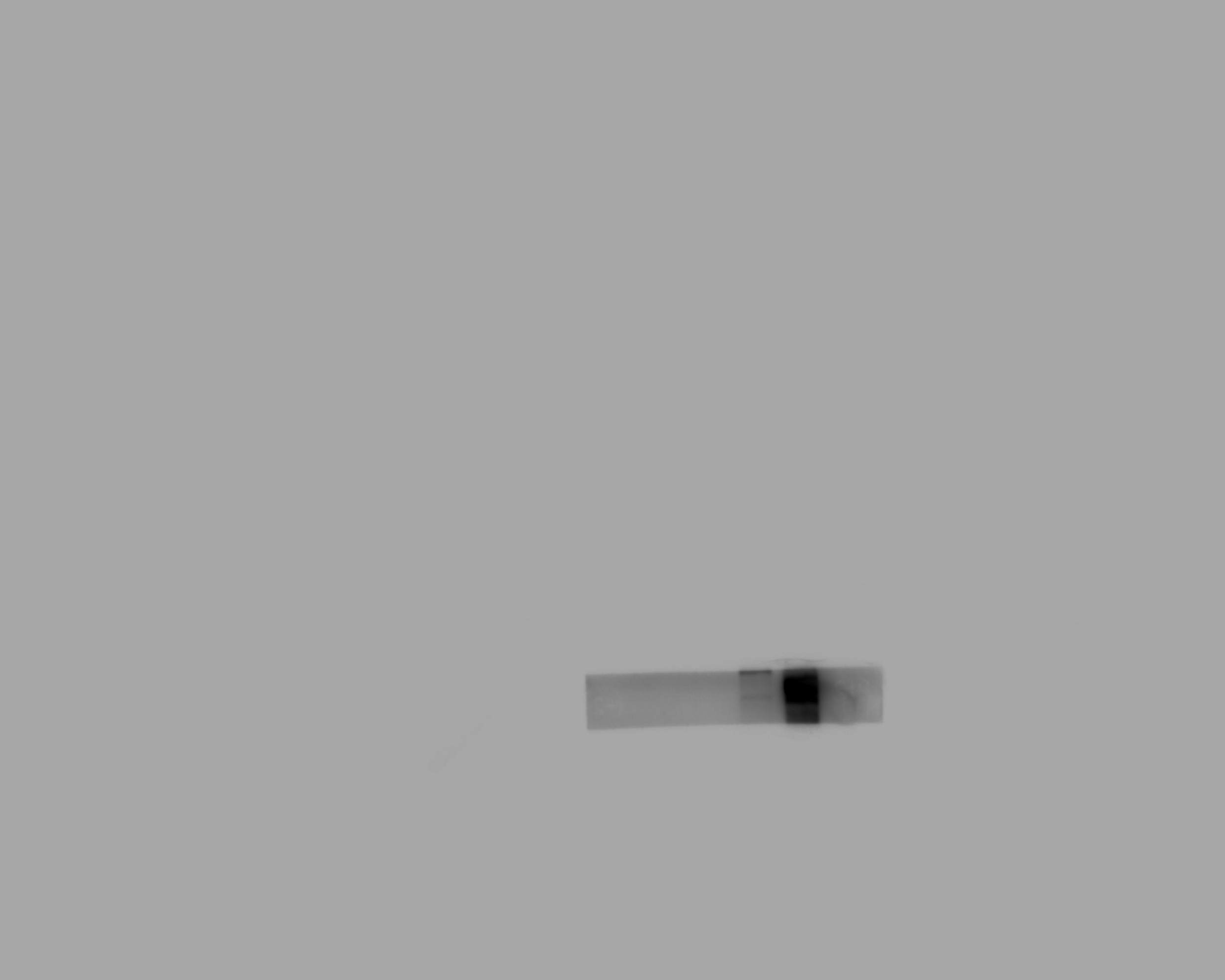

Supplement: Supplementary file 8 [file DataSheet1.zip › CO-IP/exogenous immunoprecipitation/cotransfection/IP SP1-Flag+IB HDAC5-Myc+ IP-P-gp-HA+ IB SP1-Flag/SP1-Flag.tif]

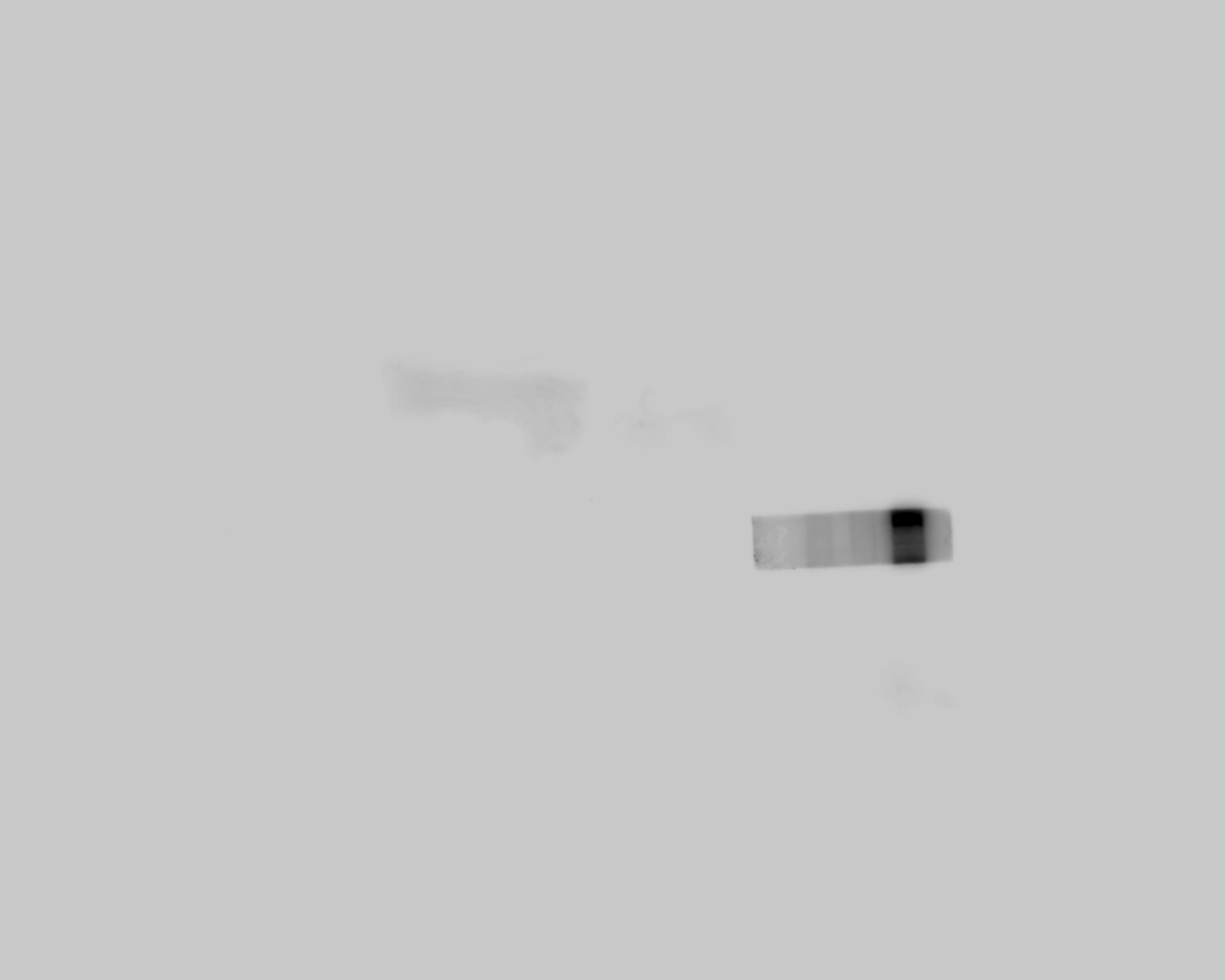

Supplement: Supplementary file 8 [file DataSheet1.zip › CO-IP/exogenous immunoprecipitation/cotransfection/IP SP1-Flag+IB P-gp-HA/SP1-Flag.tif]

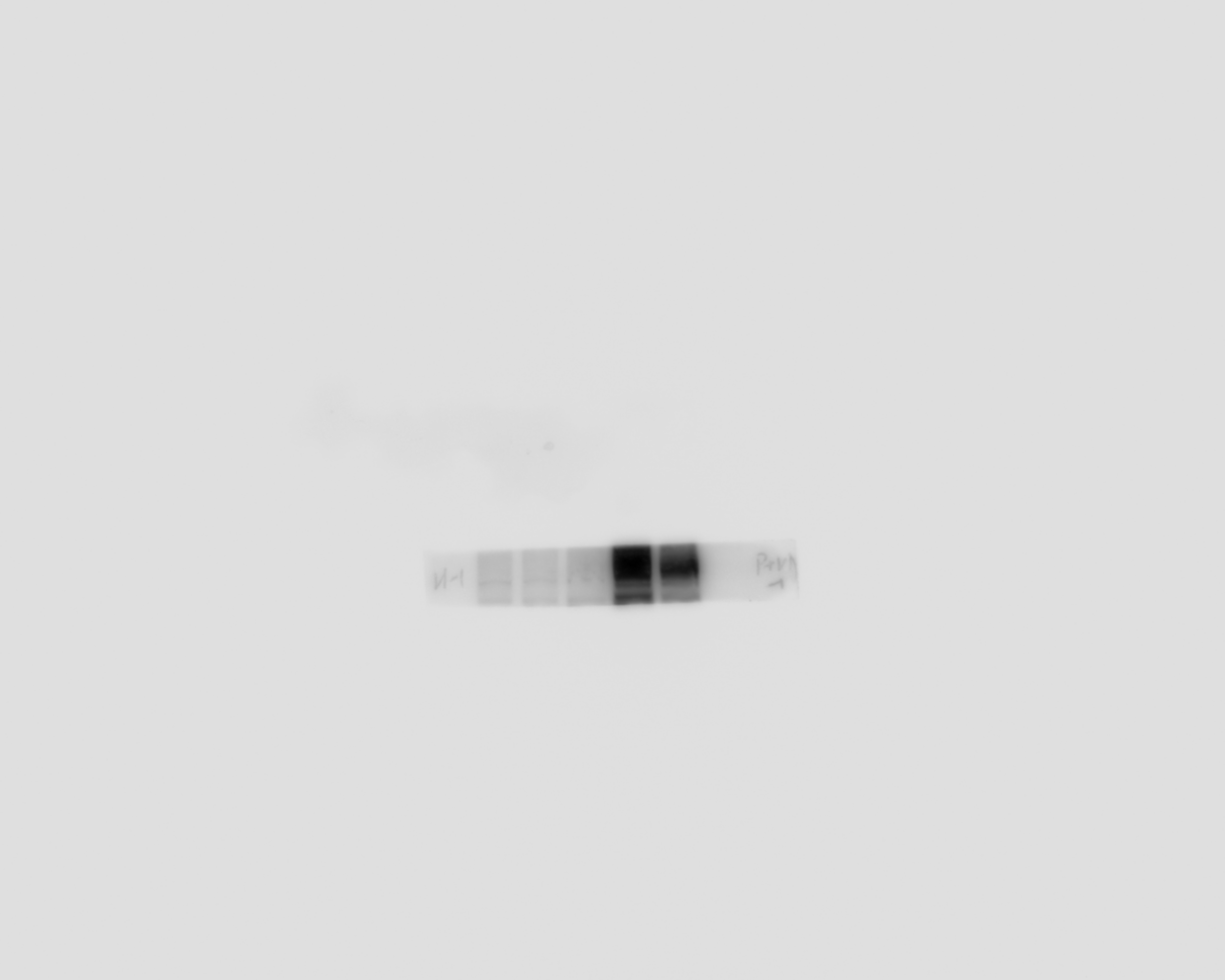

Supplement: Supplementary file 8 [file DataSheet1.zip › CO-IP/exogenous immunoprecipitation/input/P-gp-HA+HDAC5-Myc input/P-gp-HA+HDAC5-Myc-HDAC5-Myc input.tif]

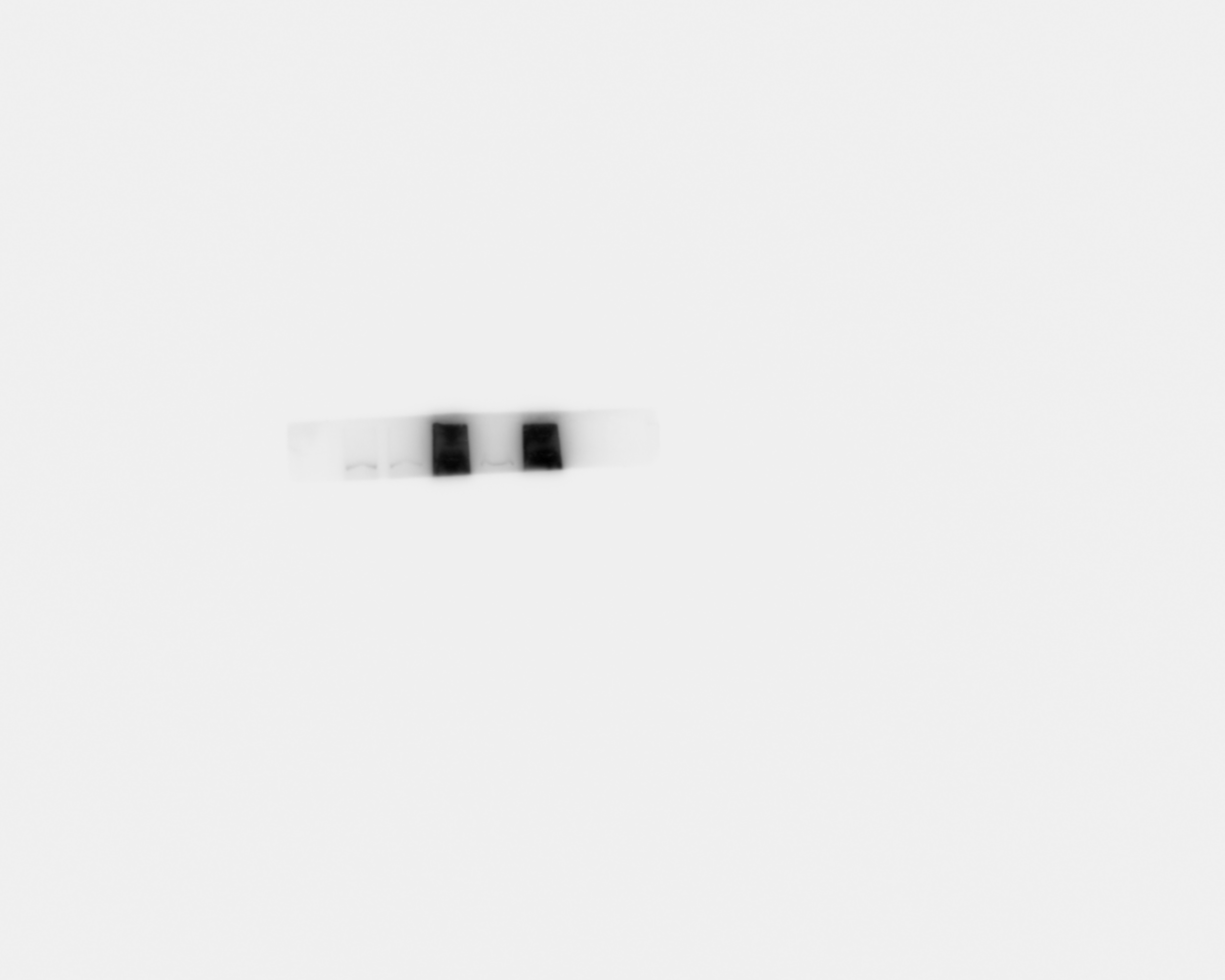

Supplement: Supplementary file 8 [file DataSheet1.zip › CO-IP/exogenous immunoprecipitation/input/P-gp-HA+HDAC5-Myc input/P-gp-HA+HDAC5-Myc-P-gp-HA input.tif]

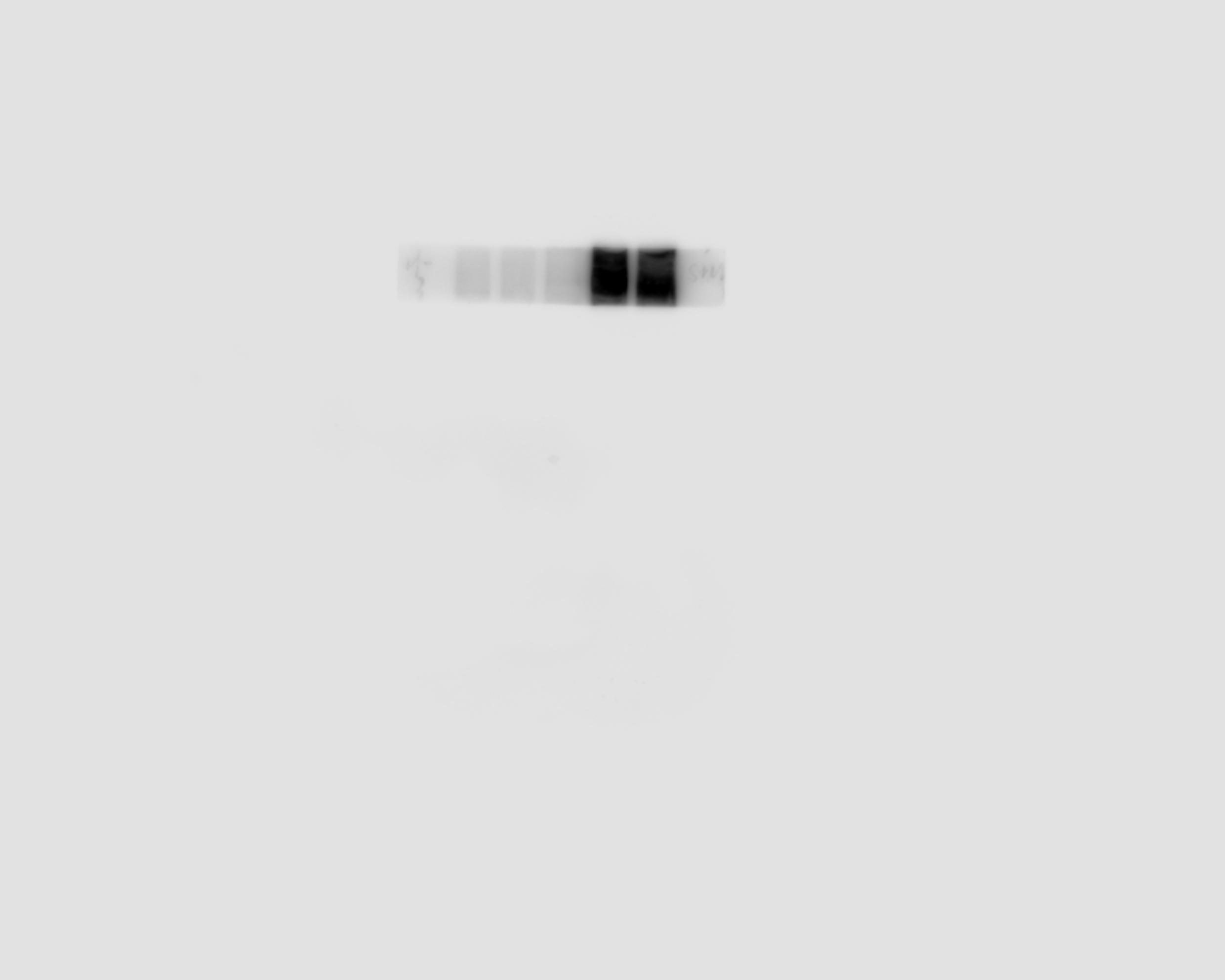

Supplement: Supplementary file 8 [file DataSheet1.zip › CO-IP/exogenous immunoprecipitation/input/SP1-Flag+HDAC5-Myc input/SP1-Flag+HDAC5-Myc-HDAC5-Myc input.tif]

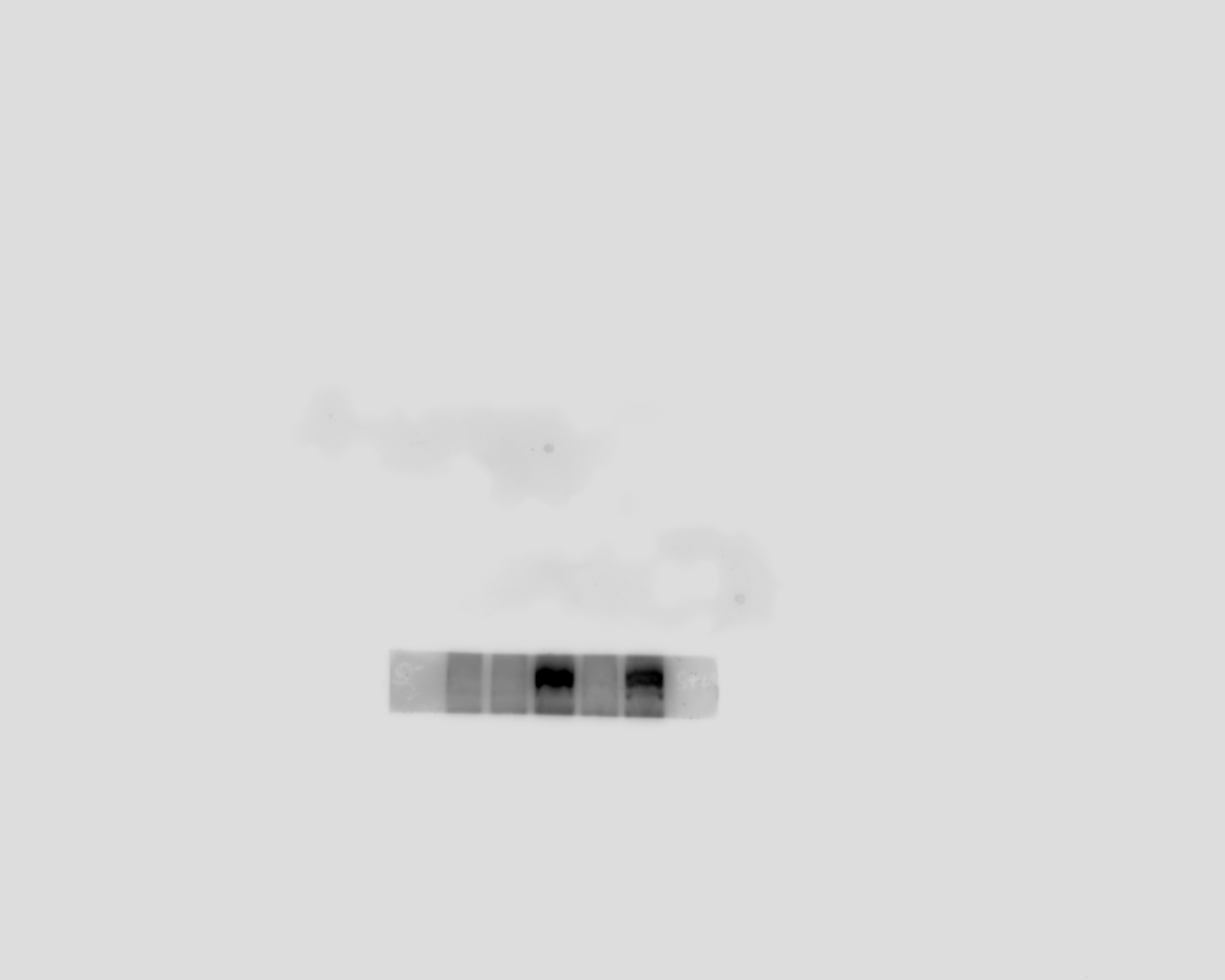

Supplement: Supplementary file 8 [file DataSheet1.zip › CO-IP/exogenous immunoprecipitation/input/SP1-Flag+HDAC5-Myc input/SP1-Flag+HDAC5-Myc-SP1-Flag input.tif]

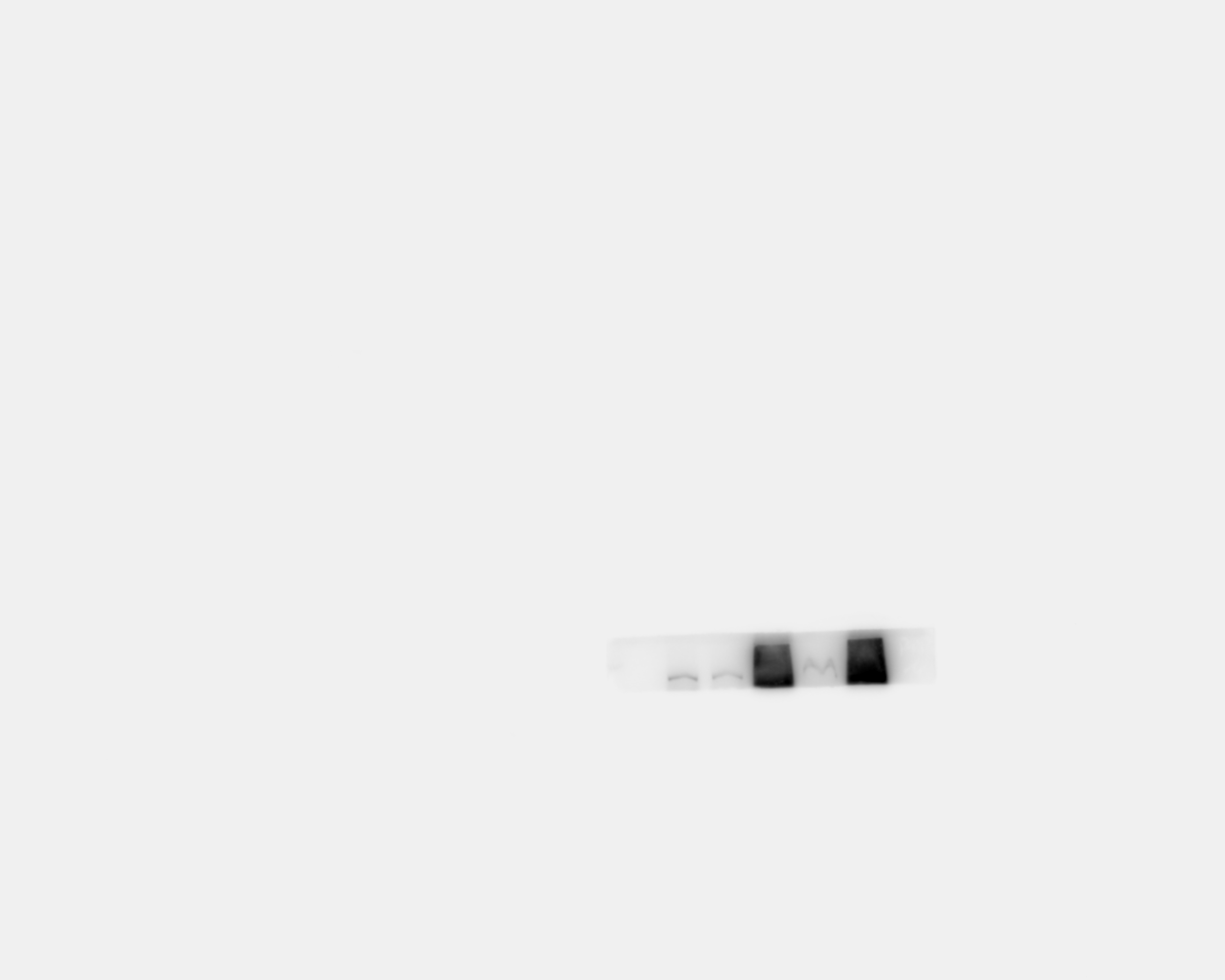

Supplement: Supplementary file 8 [file DataSheet1.zip › CO-IP/exogenous immunoprecipitation/input/SP1-Flag+P-gp-HA input/SP1-Flag+P-gp-HA-P-gp-HA input.tif]

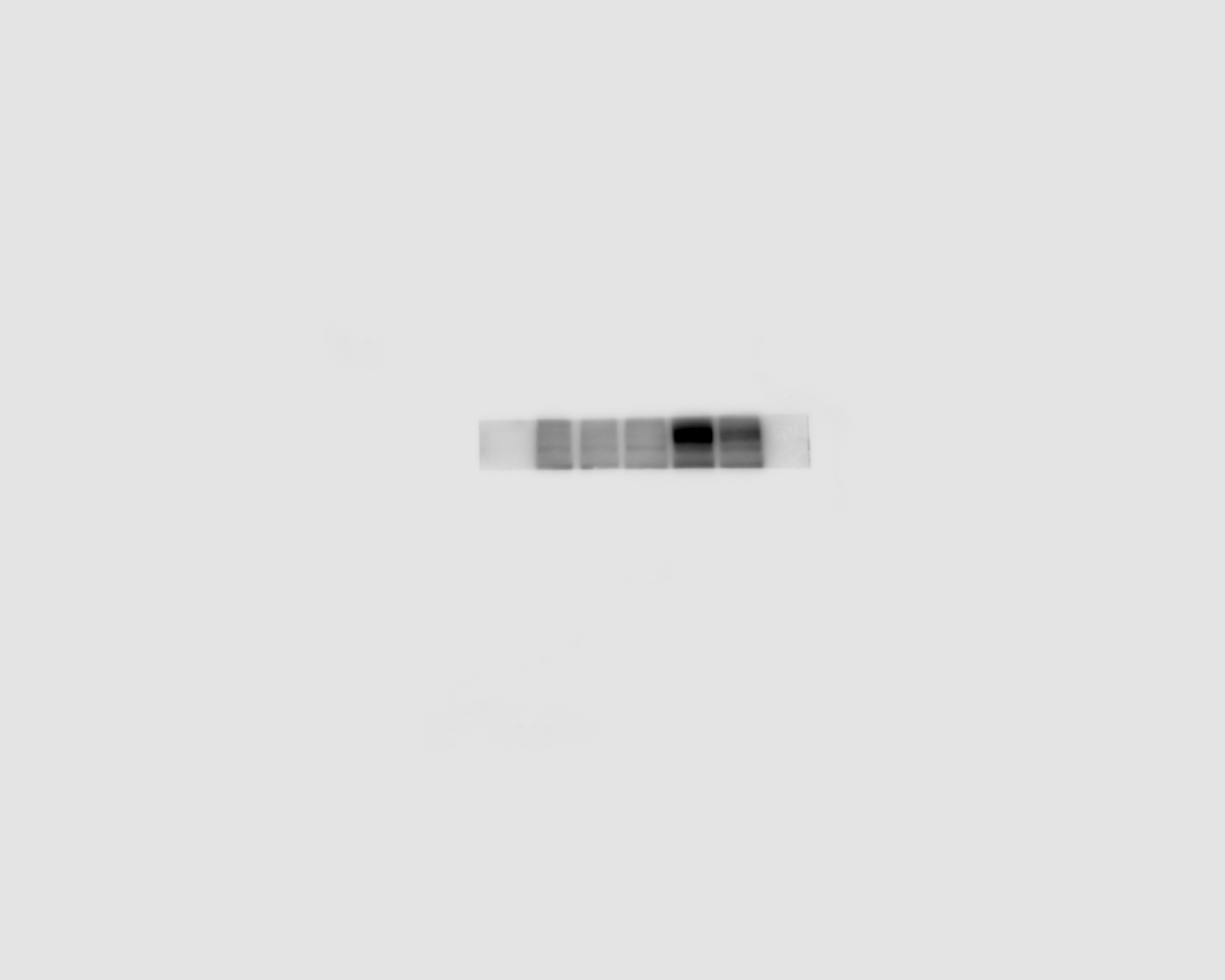

Supplement: Supplementary file 8 [file DataSheet1.zip › CO-IP/exogenous immunoprecipitation/input/SP1-Flag+P-gp-HA input/SP1-Flag+P-gp-HA-SP1-Flag-Input.tif]

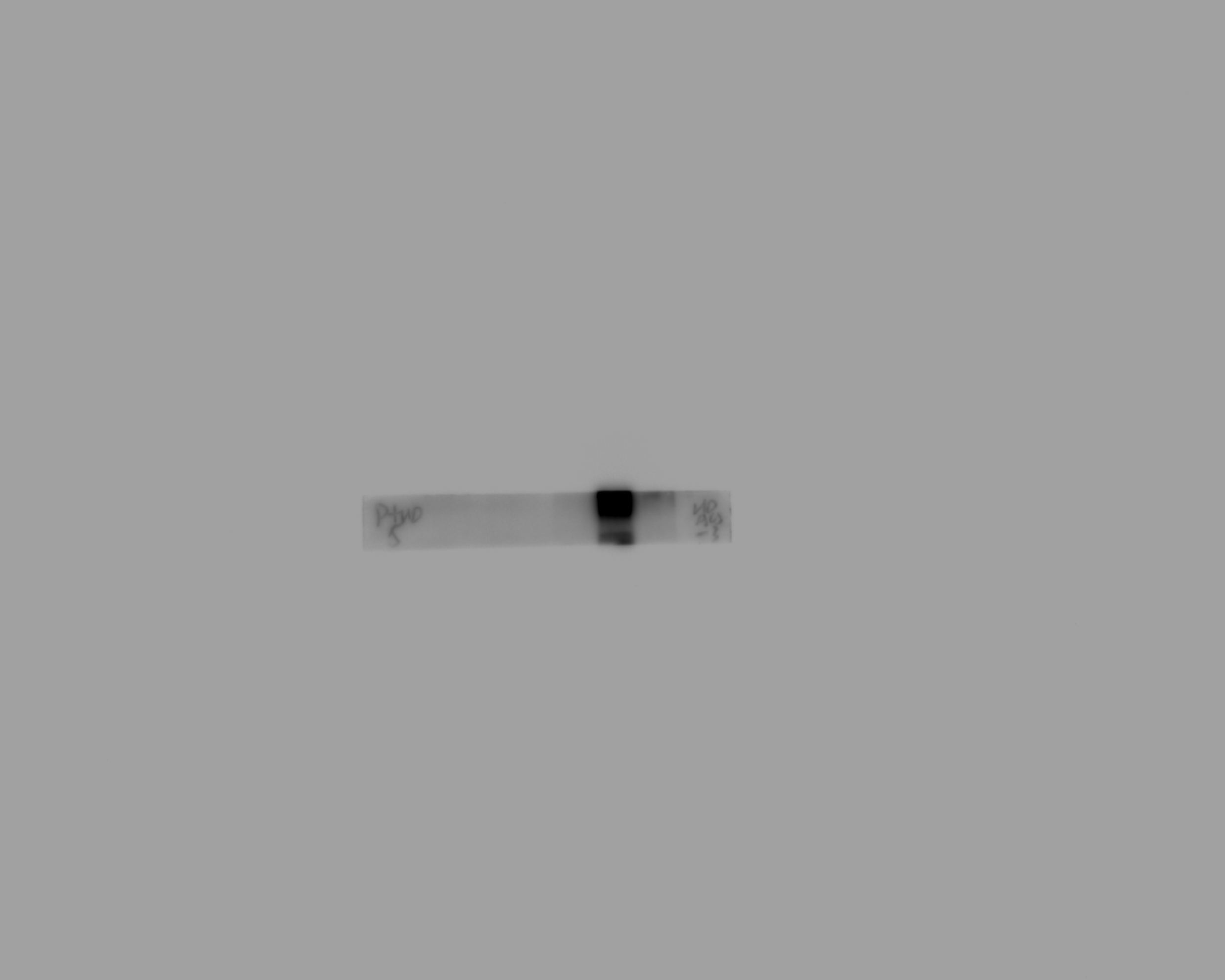

Supplement: Supplementary file 8 [file DataSheet1.zip › CO-IP/exogenous immunoprecipitation/Single gene transfection/HDAC5-Myc.tif]

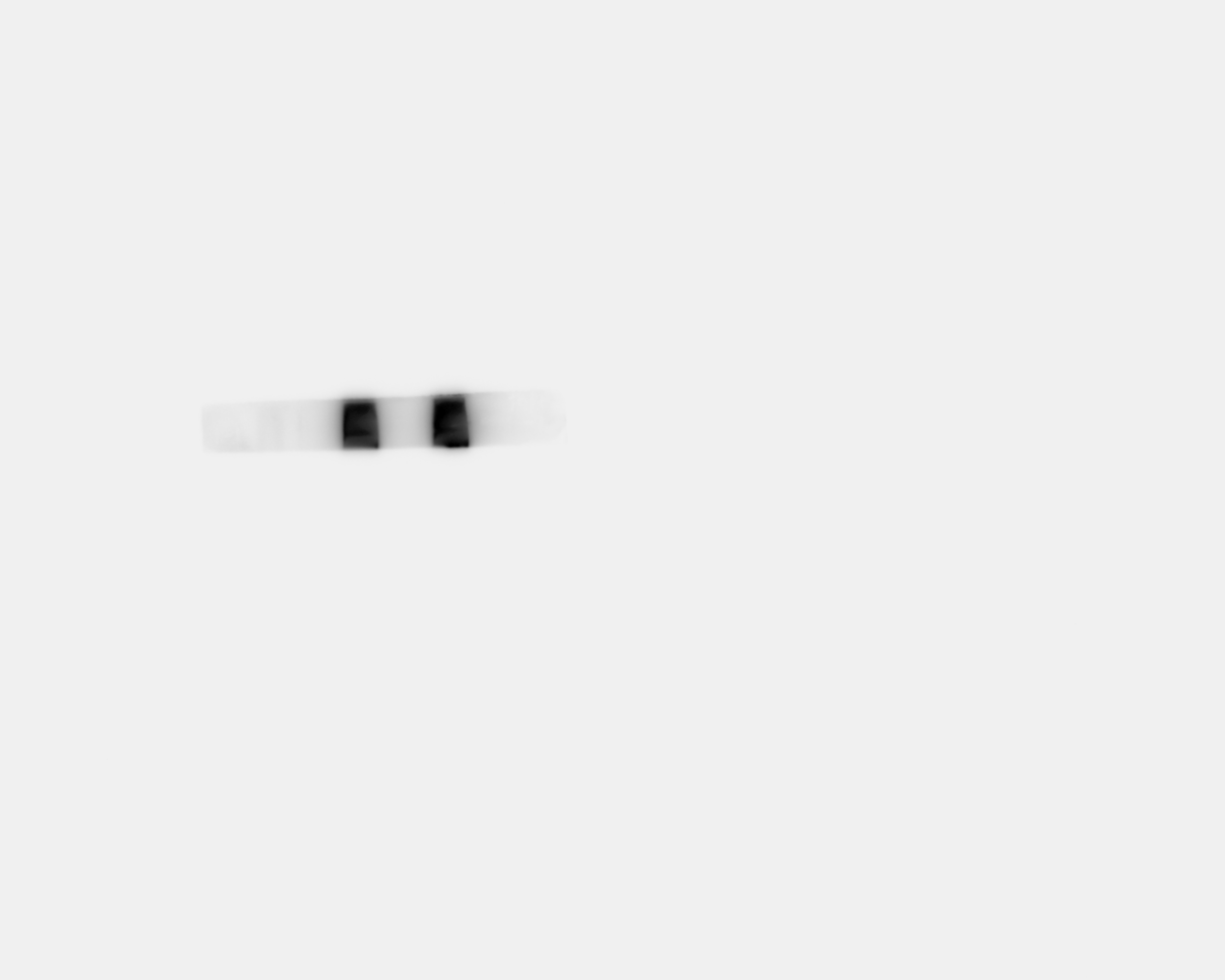

Supplement: Supplementary file 8 [file DataSheet1.zip › CO-IP/exogenous immunoprecipitation/Single gene transfection/P-gp-HA.tif]

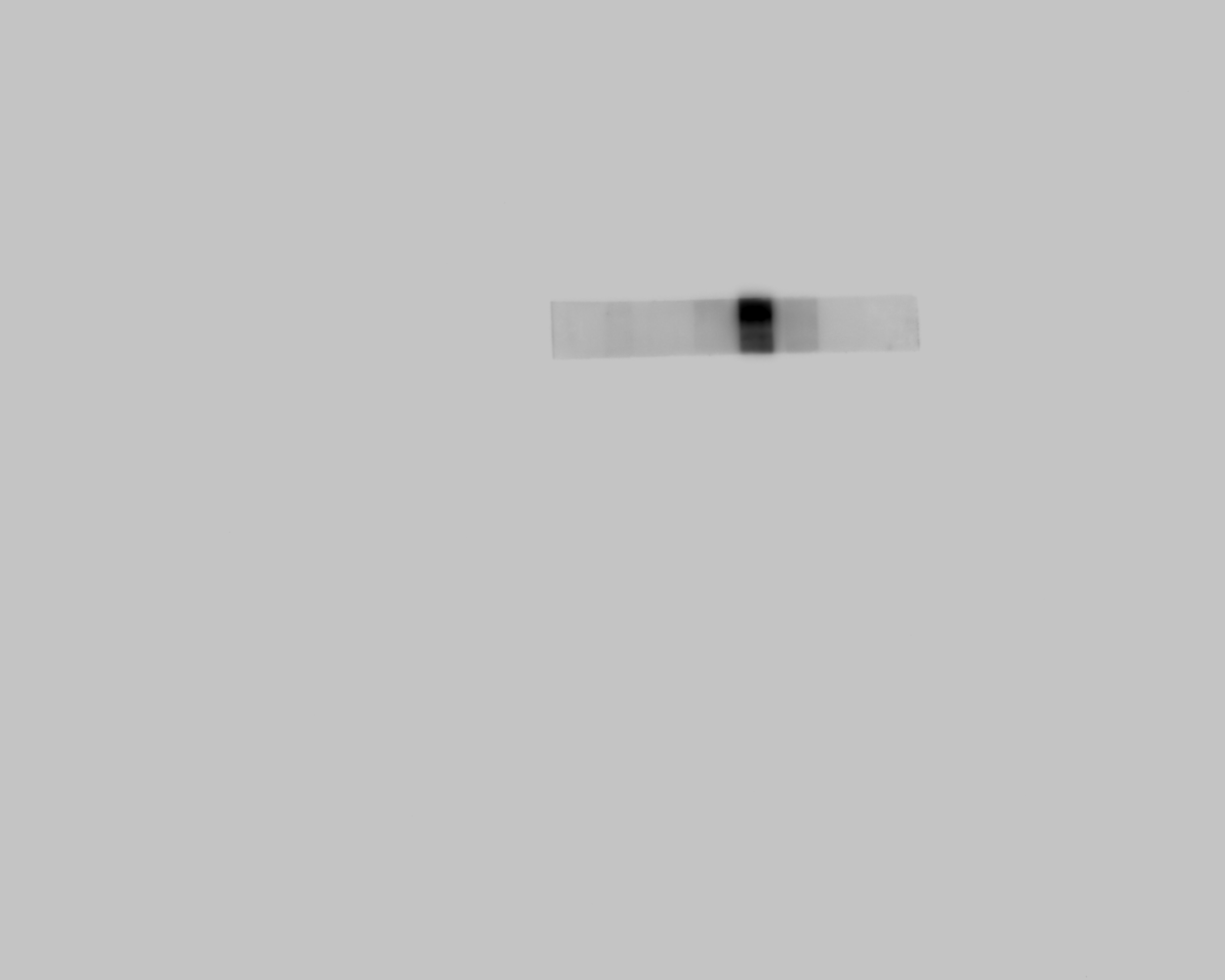

Supplement: Supplementary file 8 [file DataSheet1.zip › CO-IP/exogenous immunoprecipitation/Single gene transfection/SP1-Flag.tif]

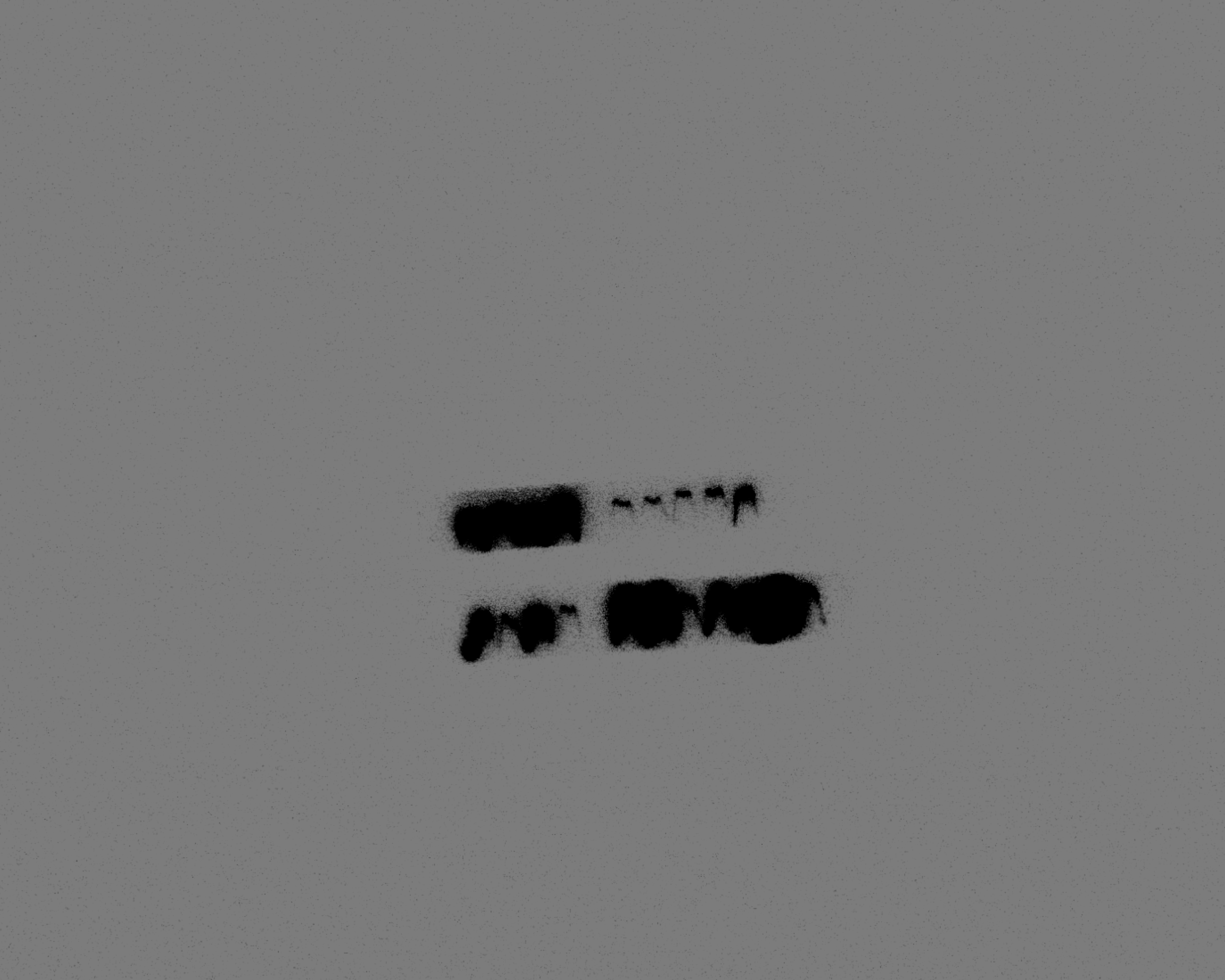

Supplement: Supplementary file 9 [file DataSheet10.zip › siHDAC5-P-gp and HDAC5 and H3K9ac/siHDAC5-H3K9ac/H3K9ac.tif]

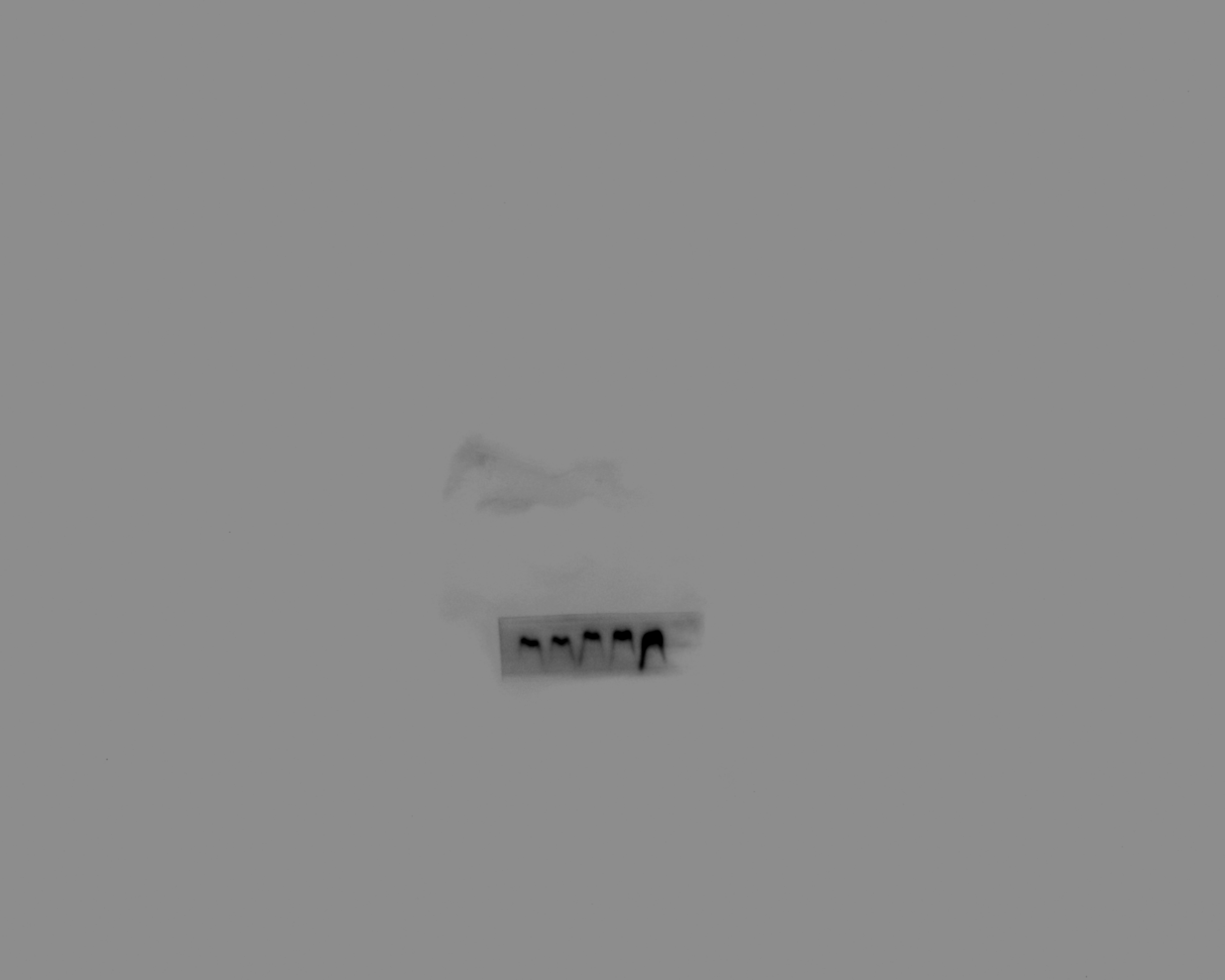

Supplement: Supplementary file 9 [file DataSheet10.zip › siHDAC5-P-gp and HDAC5 and H3K9ac/siHDAC5-H3K9ac/H3K9ac-1.tif]

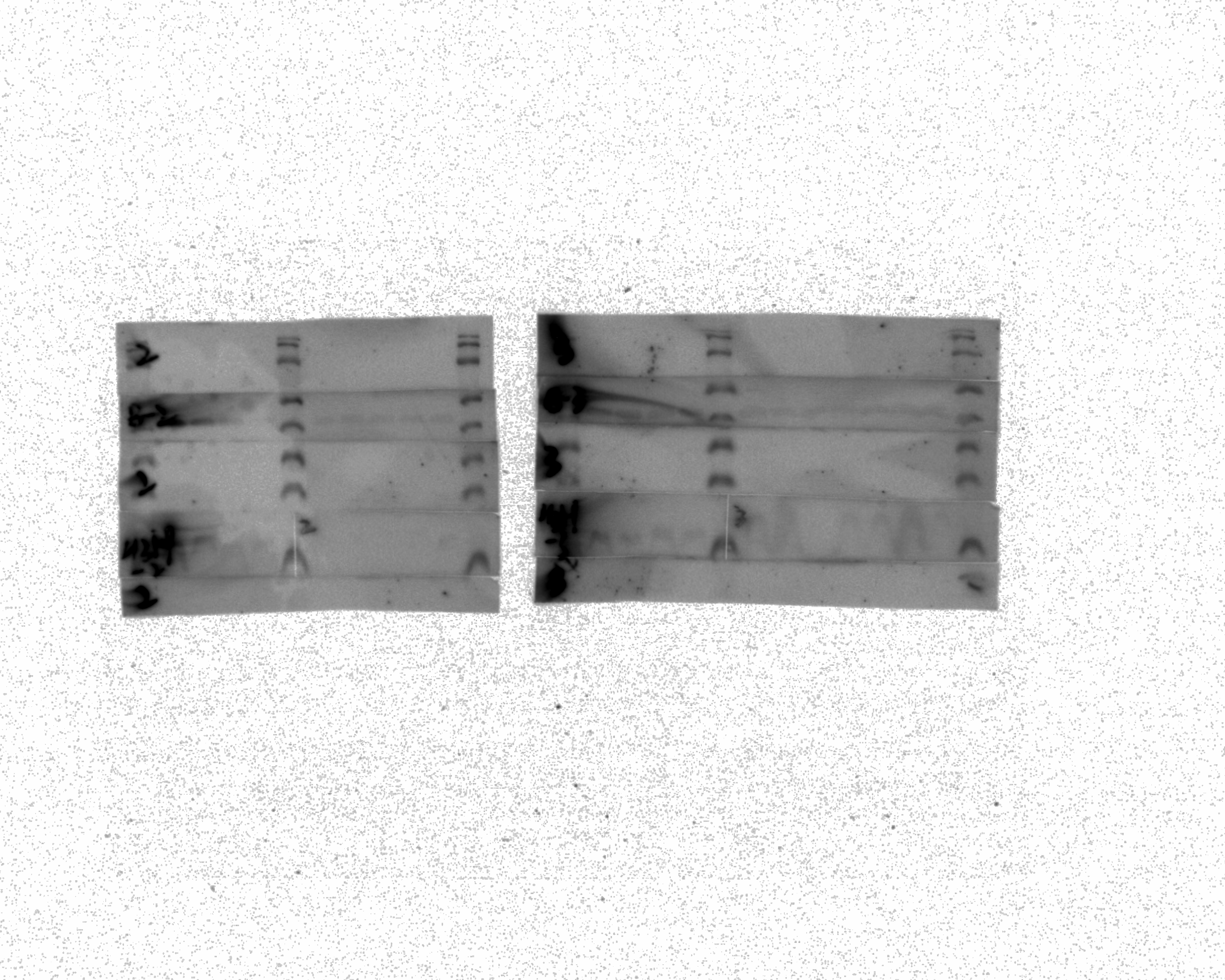

Supplement: Supplementary file 9 [file DataSheet10.zip › siHDAC5-P-gp and HDAC5 and H3K9ac/siHDAC5-H3K9ac/Western blot membrane cutting.tif]

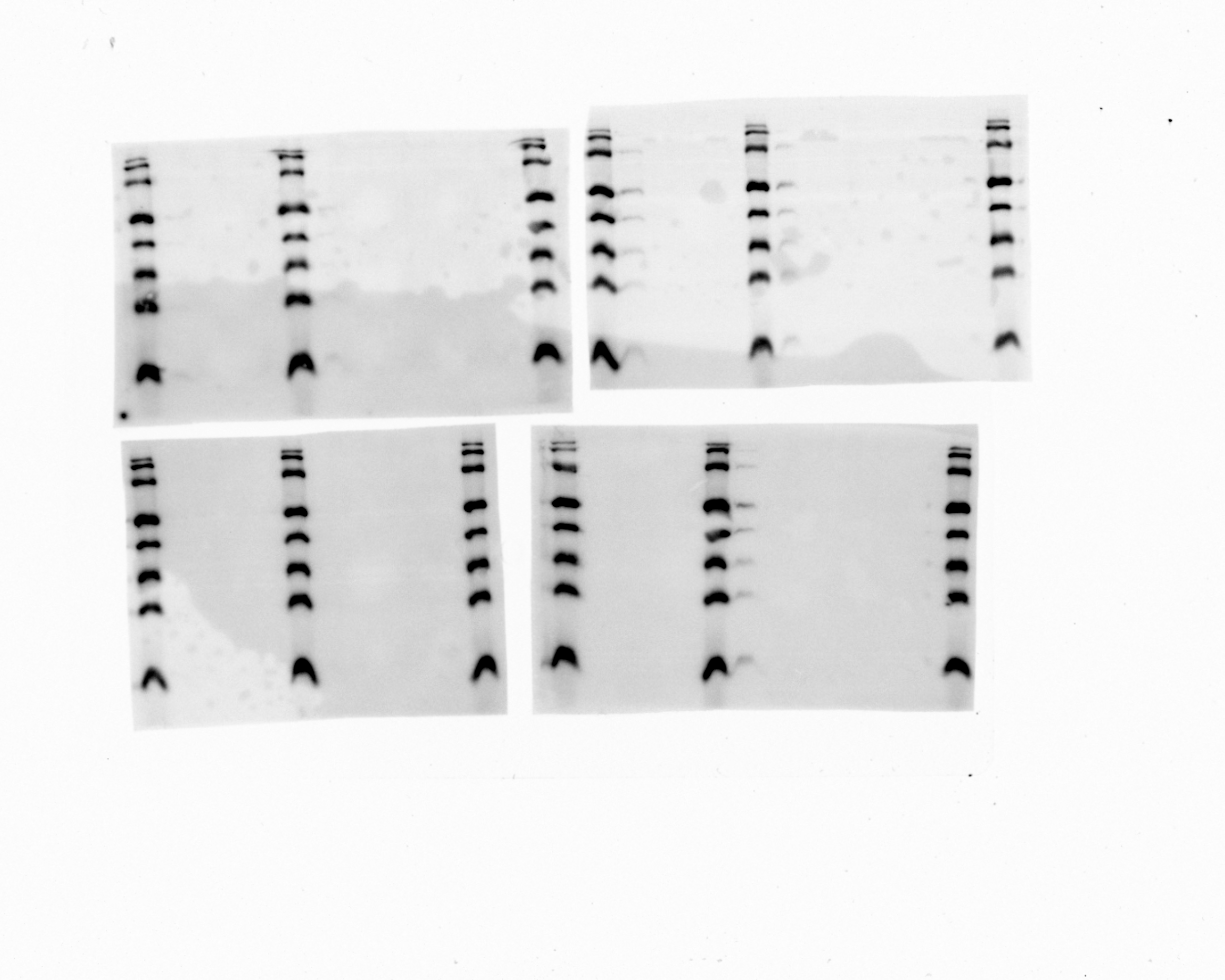

Supplement: Supplementary file 9 [file DataSheet10.zip › siHDAC5-P-gp and HDAC5 and H3K9ac/siHDAC5-H3K9ac/Whole Western blot membrane.tif]

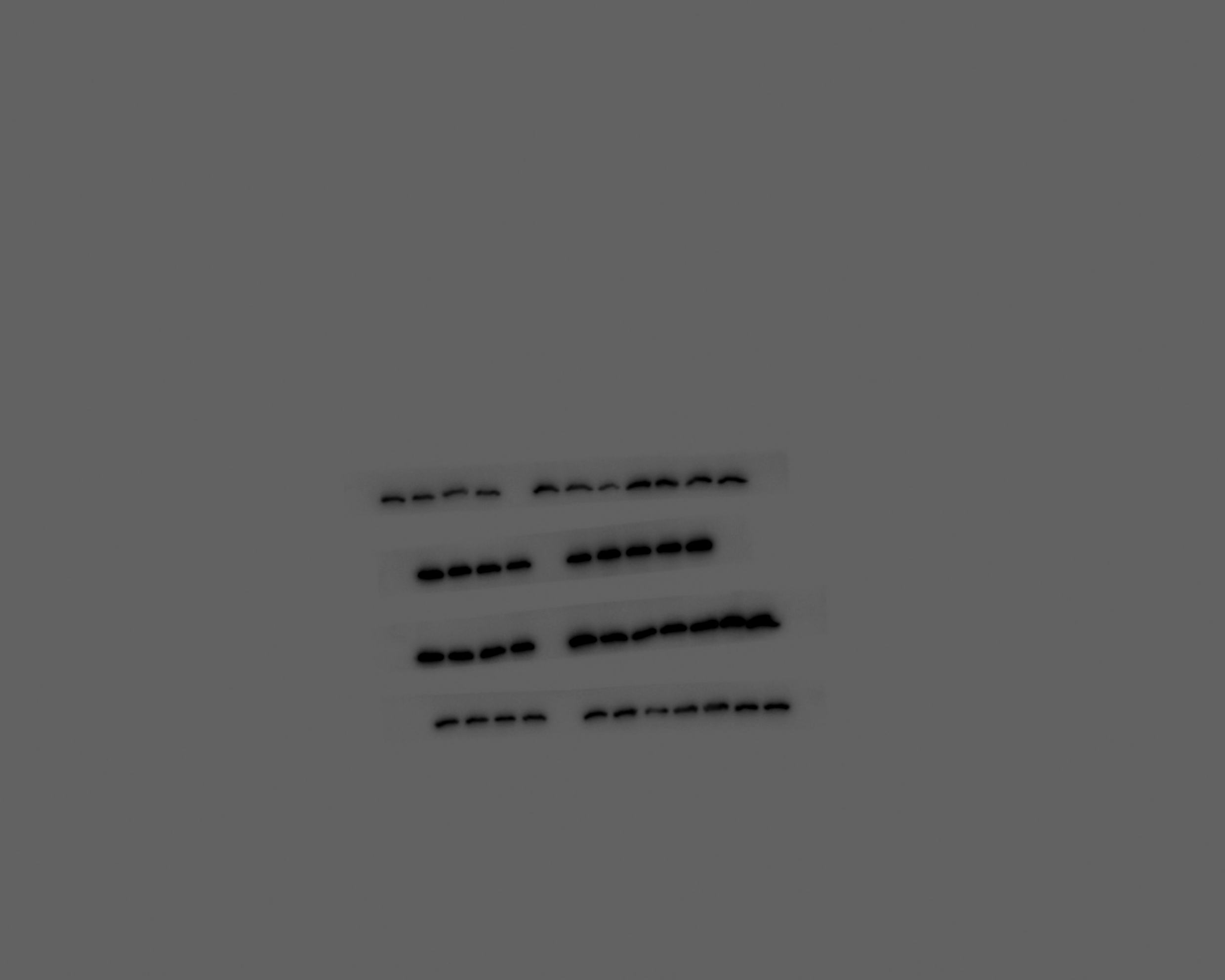

Supplement: Supplementary file 9 [file DataSheet10.zip › siHDAC5-P-gp and HDAC5 and H3K9ac/siHDAC5-H3K9ac/a┬-actin.tif]

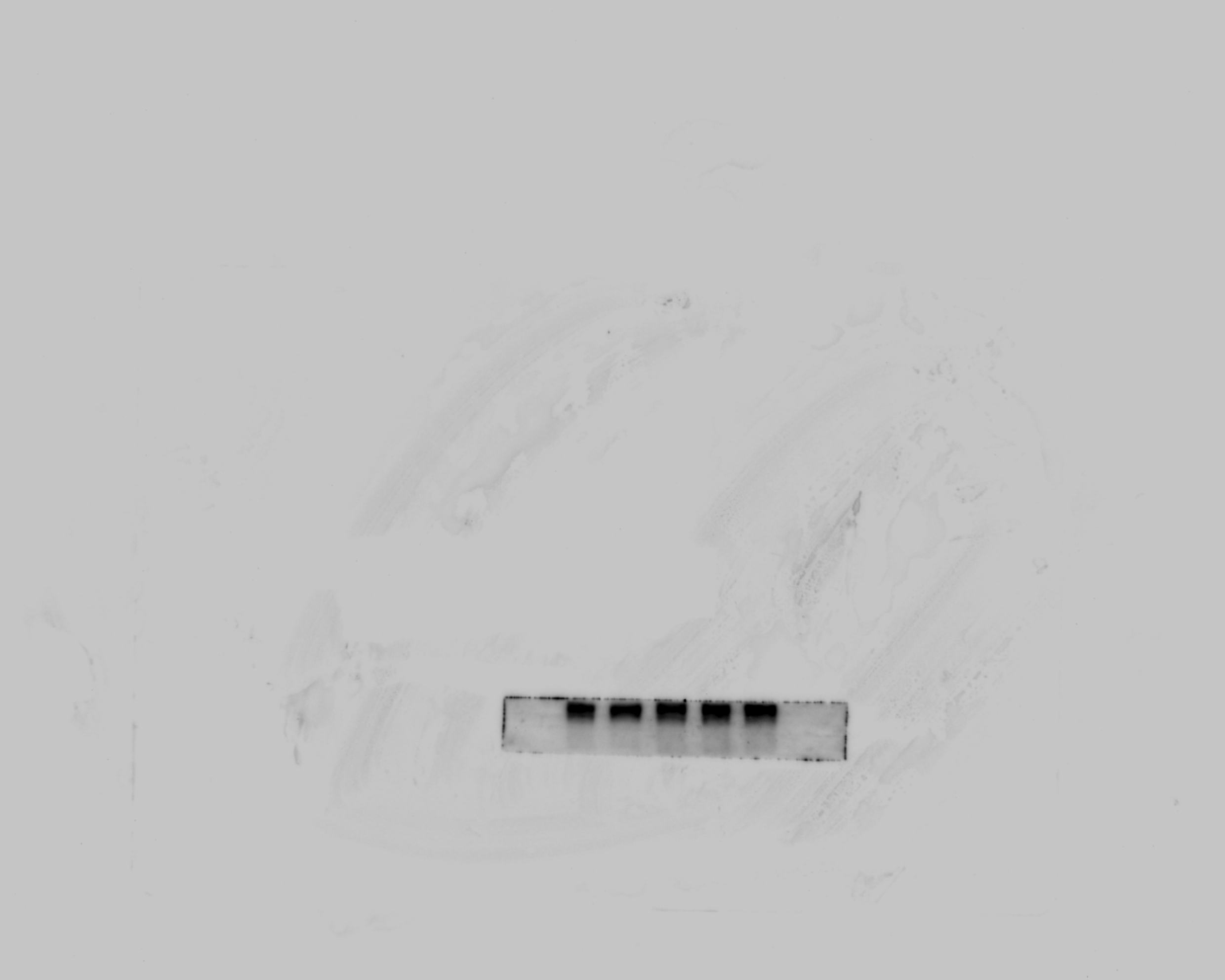

Supplement: Supplementary file 9 [file DataSheet10.zip › siHDAC5-P-gp and HDAC5 and H3K9ac/siHDAC5-P-gp and HDAC5/HDAC5.tif]

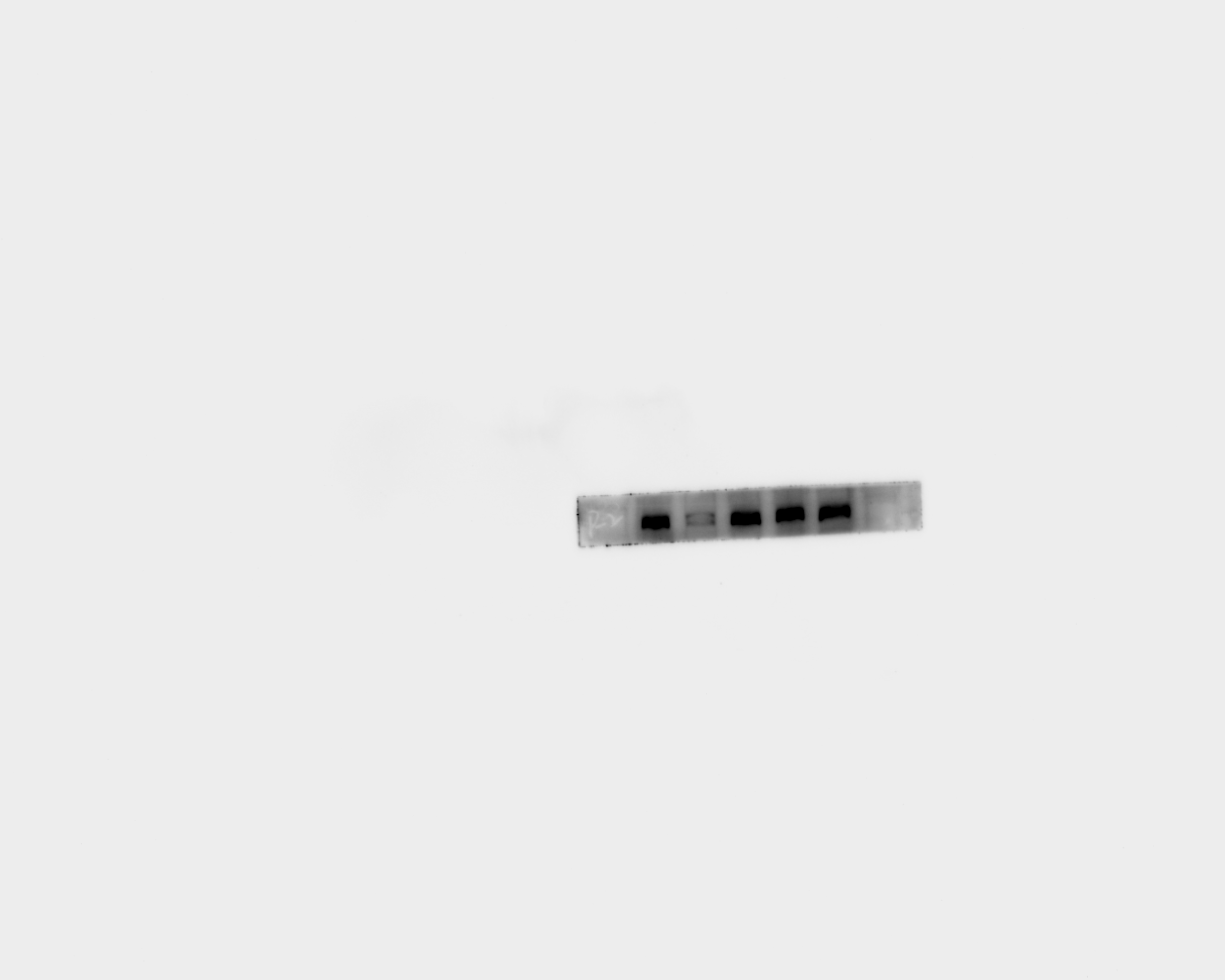

Supplement: Supplementary file 9 [file DataSheet10.zip › siHDAC5-P-gp and HDAC5 and H3K9ac/siHDAC5-P-gp and HDAC5/P-gp.tif]

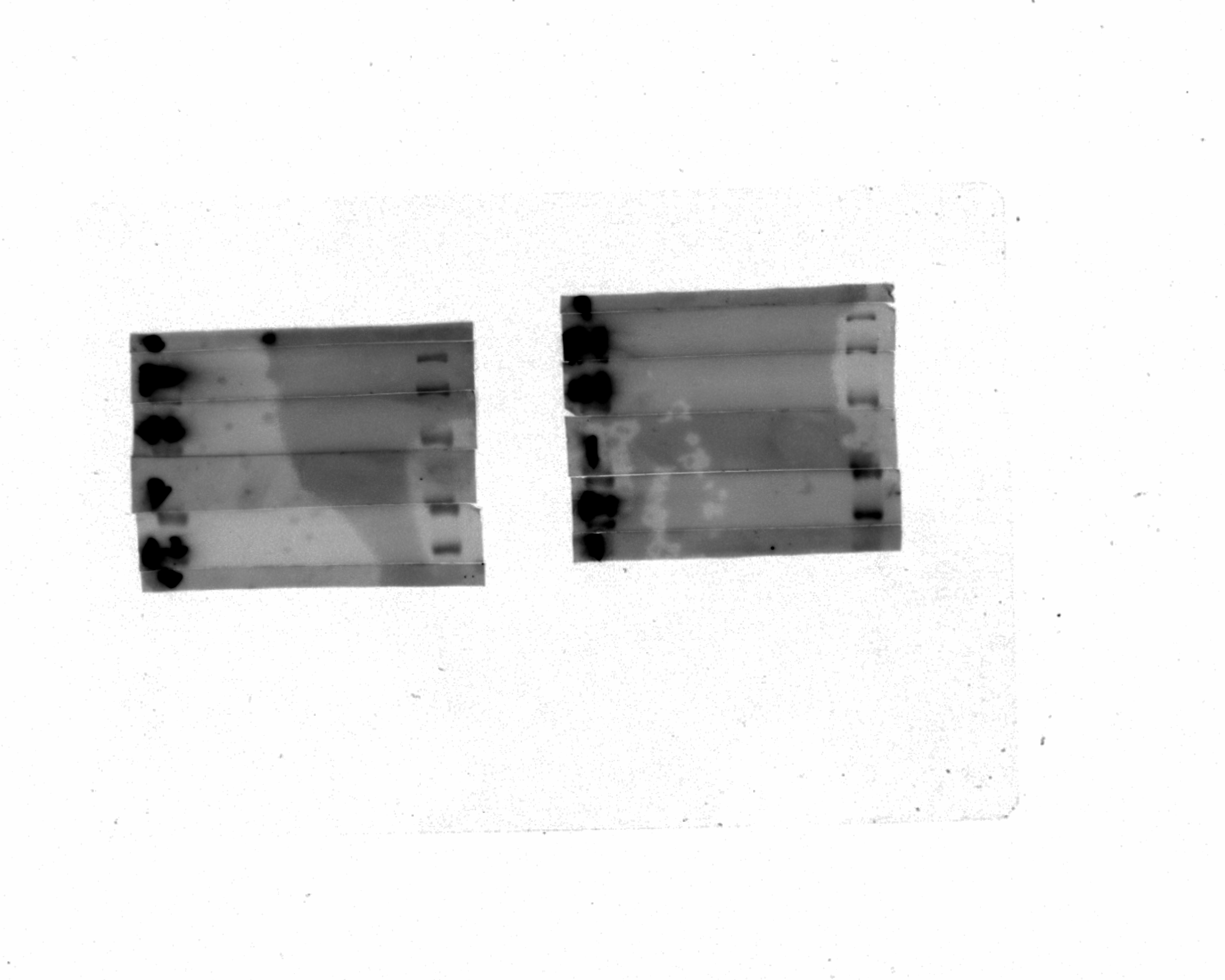

Supplement: Supplementary file 9 [file DataSheet10.zip › siHDAC5-P-gp and HDAC5 and H3K9ac/siHDAC5-P-gp and HDAC5/Western blot membrane cutting..tif]

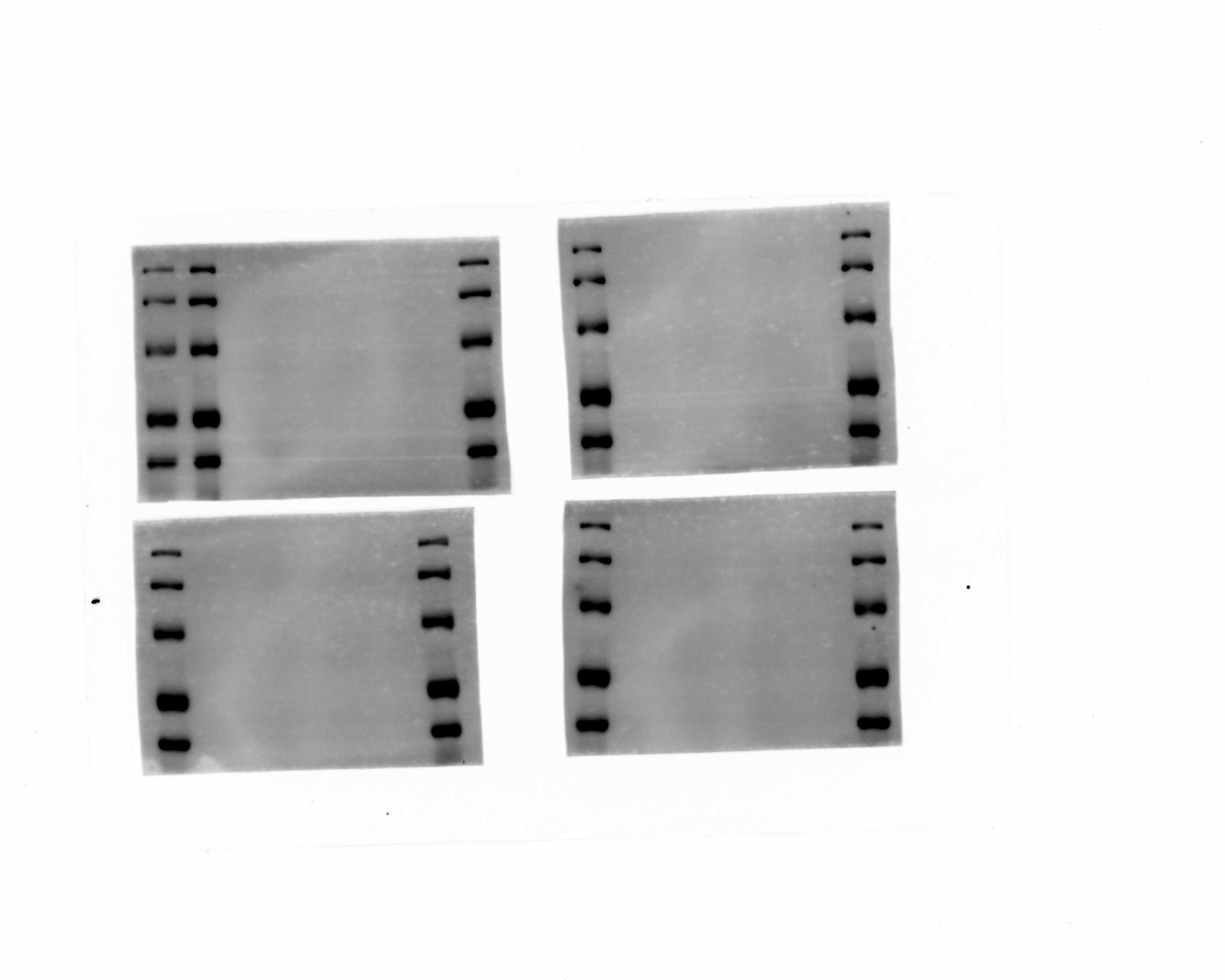

Supplement: Supplementary file 9 [file DataSheet10.zip › siHDAC5-P-gp and HDAC5 and H3K9ac/siHDAC5-P-gp and HDAC5/Whole Western blot membrane..tif]

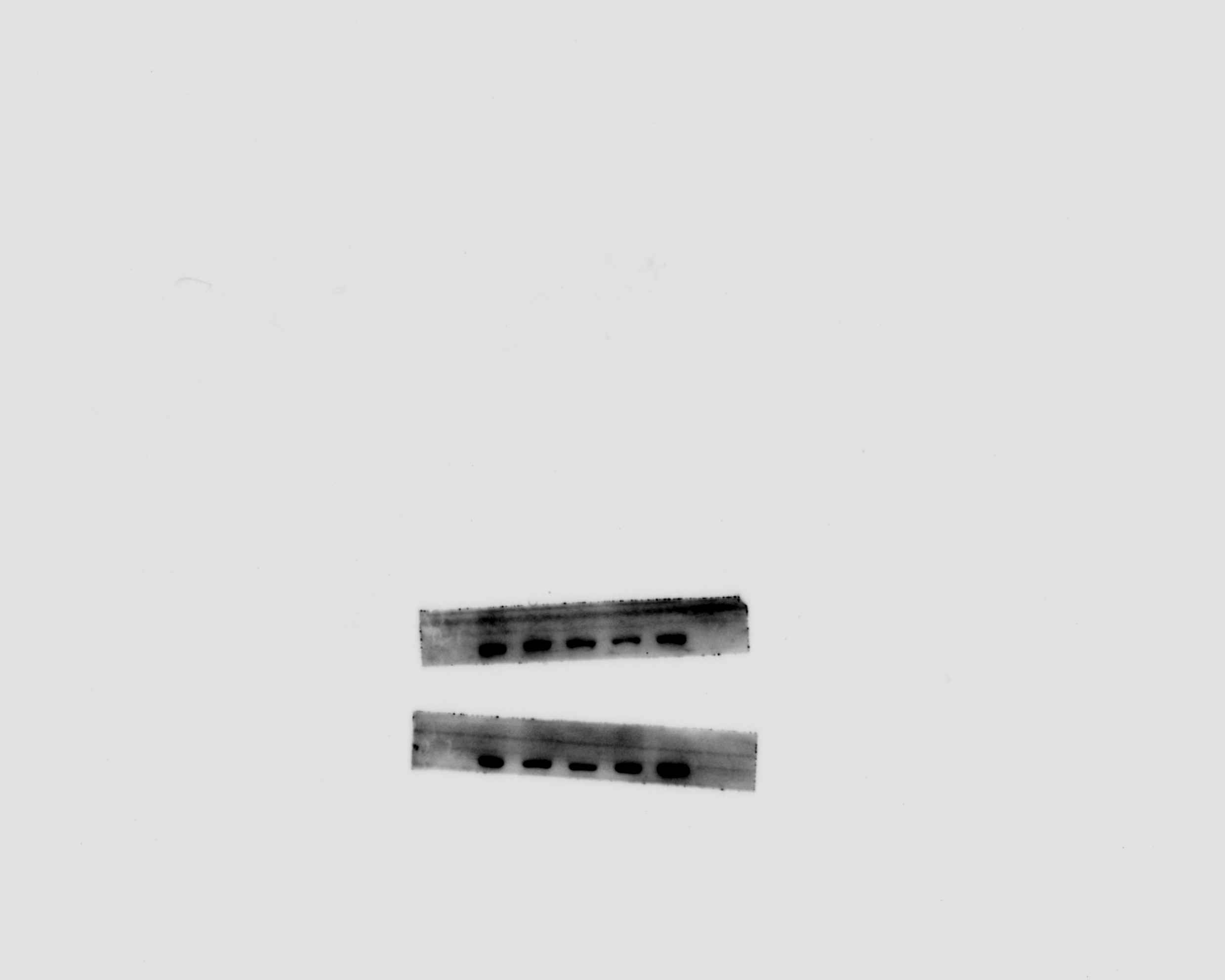

Supplement: Supplementary file 9 [file DataSheet10.zip › siHDAC5-P-gp and HDAC5 and H3K9ac/siHDAC5-P-gp and HDAC5/a┬-actin.tif]

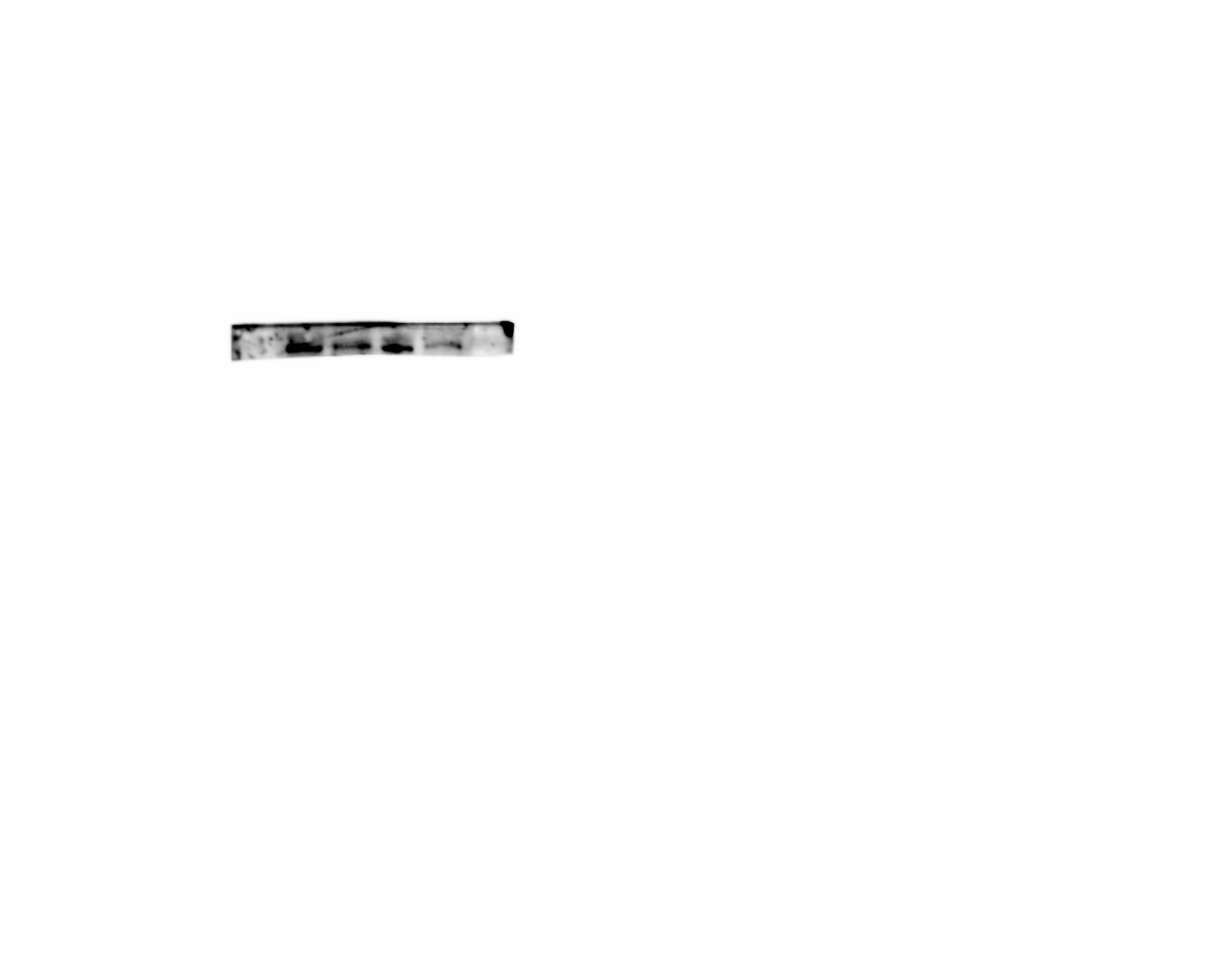

Supplement: Supplementary file 10 [file DataSheet6.zip › Normoxia vs Hypoxia-P-gp/P-gp.tif]

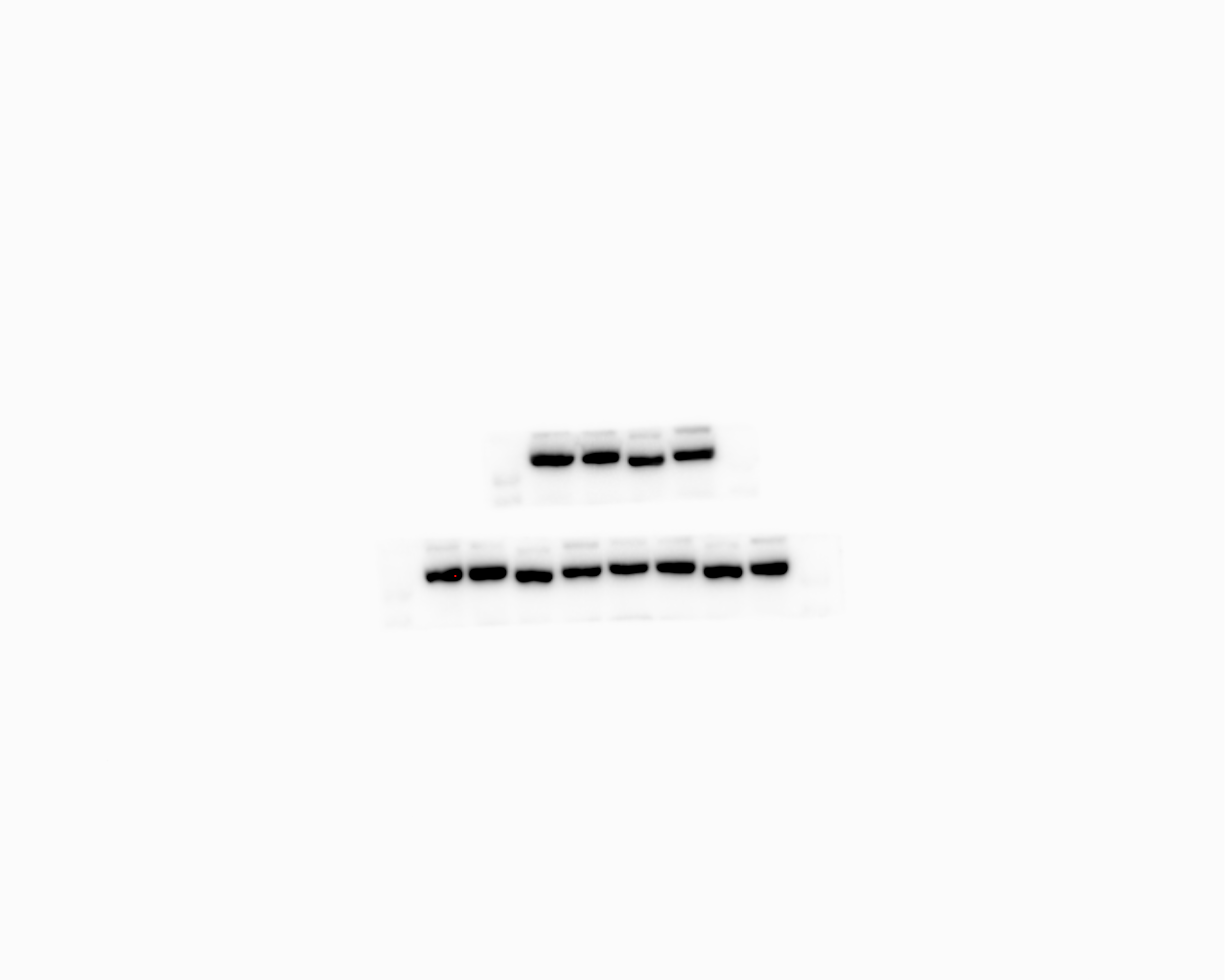

Supplement: Supplementary file 10 [file DataSheet6.zip › Normoxia vs Hypoxia-P-gp/a┬-actin.tif]

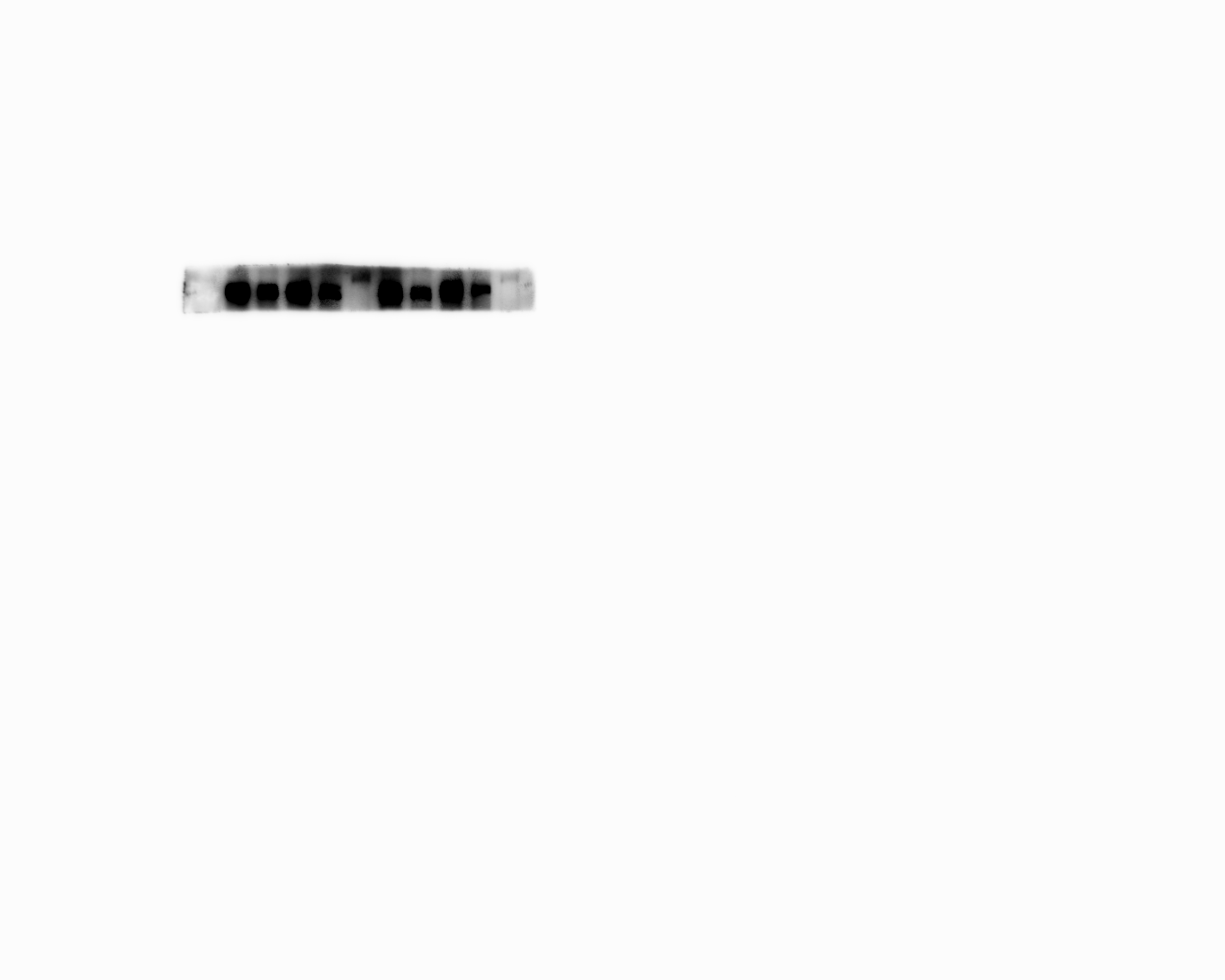

Supplement: Supplementary file 11 [file DataSheet12.zip › siSP1-P-gp/P-gp.tif]

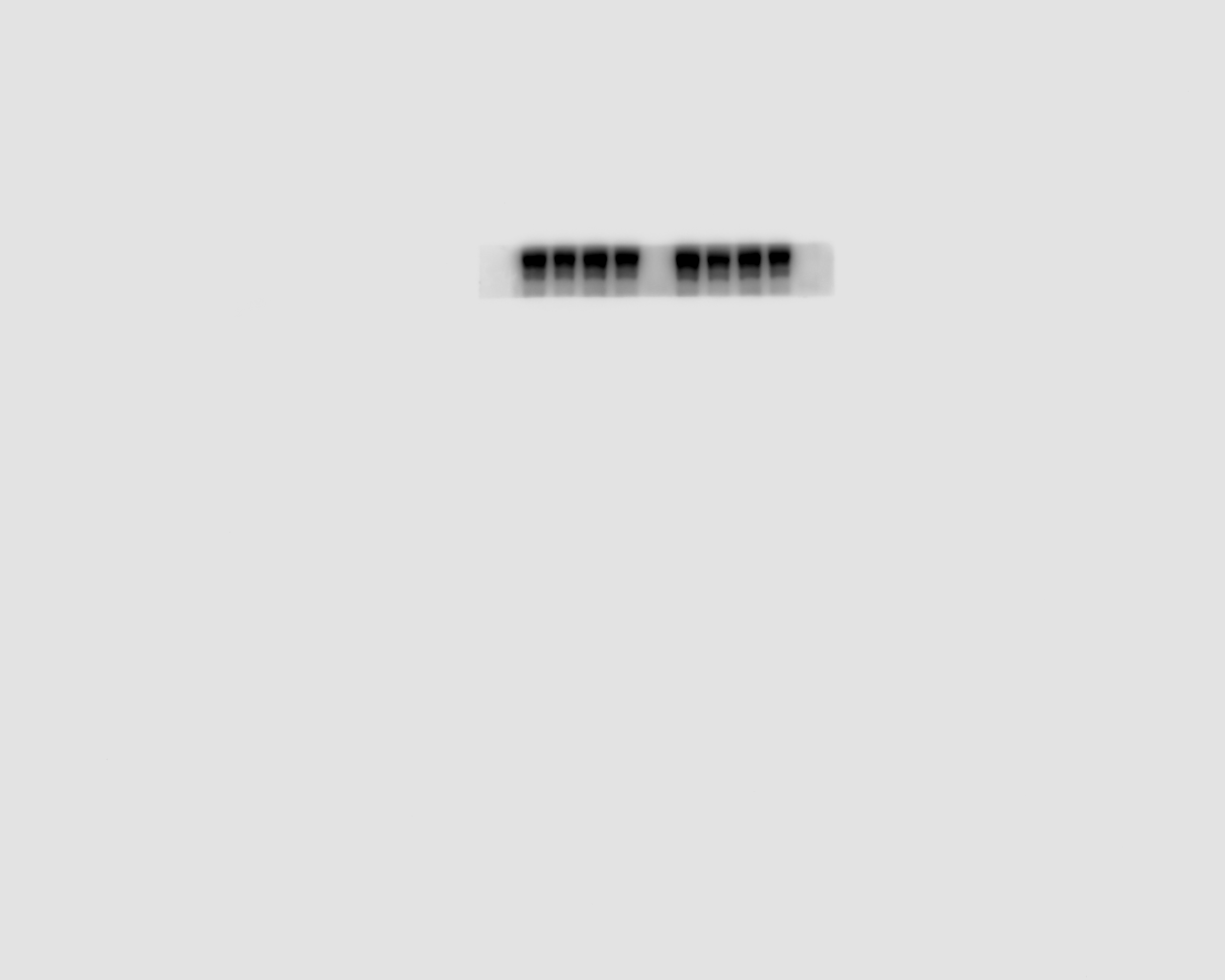

Supplement: Supplementary file 11 [file DataSheet12.zip › siSP1-P-gp/SP1.tif]

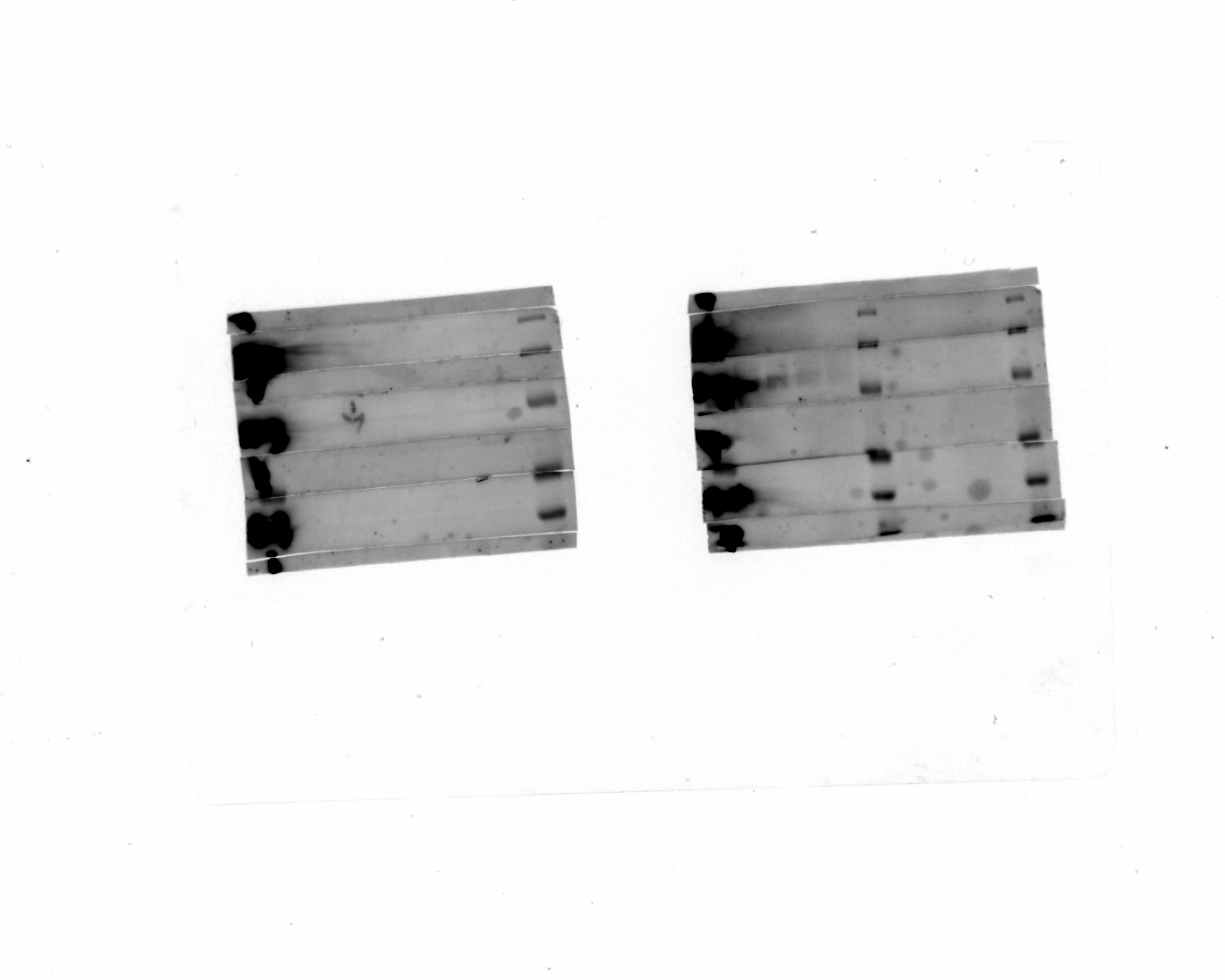

Supplement: Supplementary file 11 [file DataSheet12.zip › siSP1-P-gp/Western blot membrane cutting..tif]

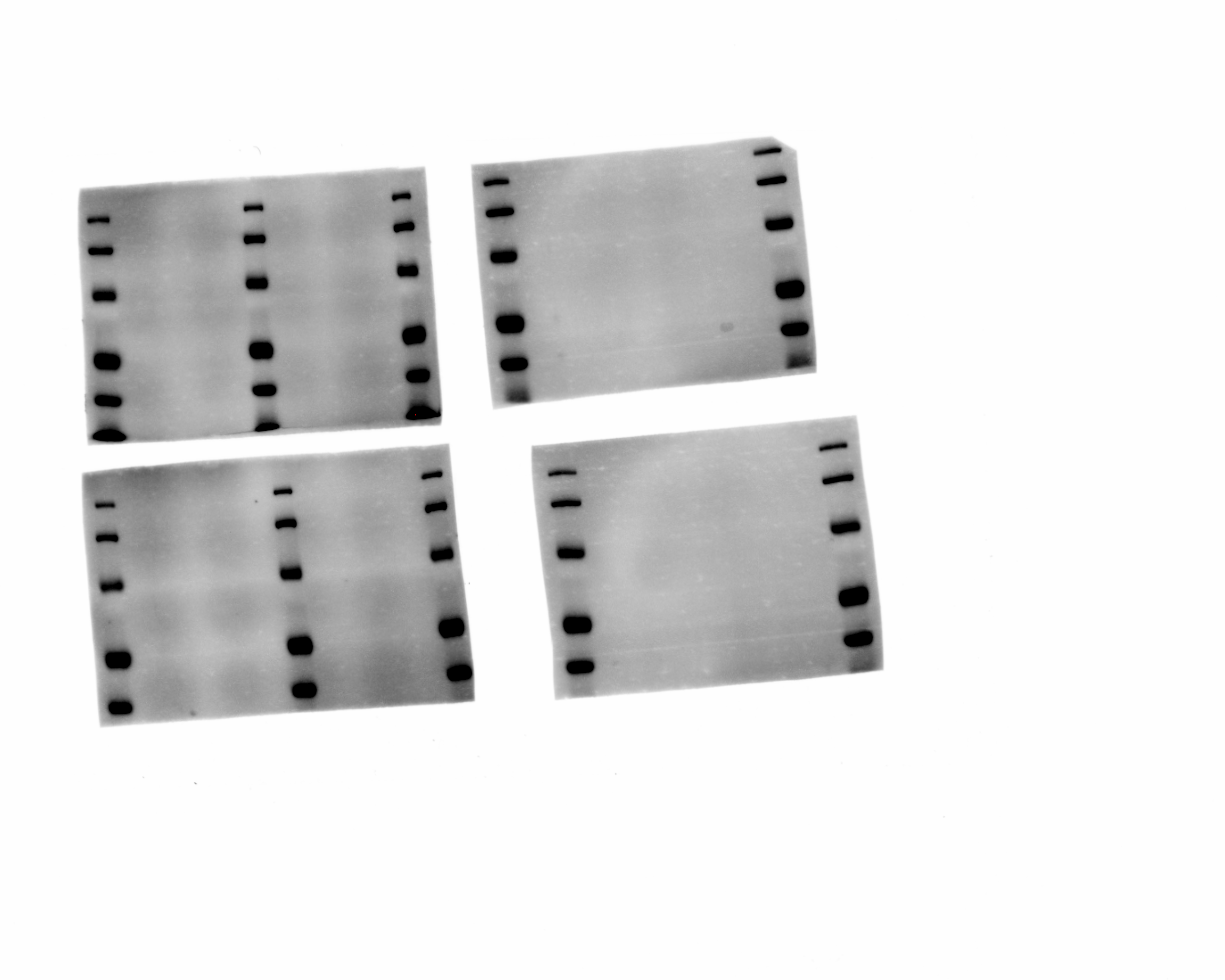

Supplement: Supplementary file 11 [file DataSheet12.zip › siSP1-P-gp/Whole Western blot membrane..tif]

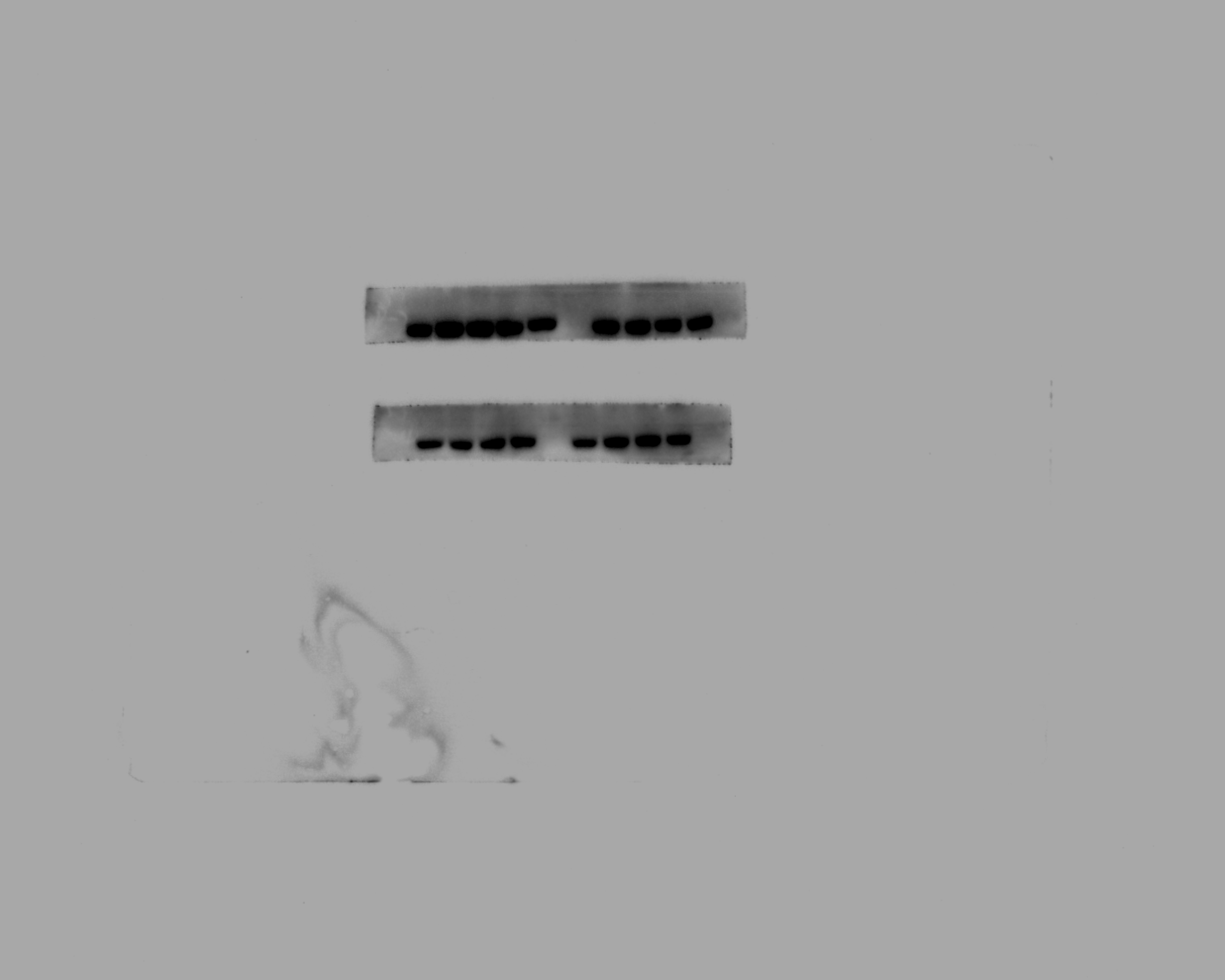

Supplement: Supplementary file 11 [file DataSheet12.zip › siSP1-P-gp/a┬-actin.tif]

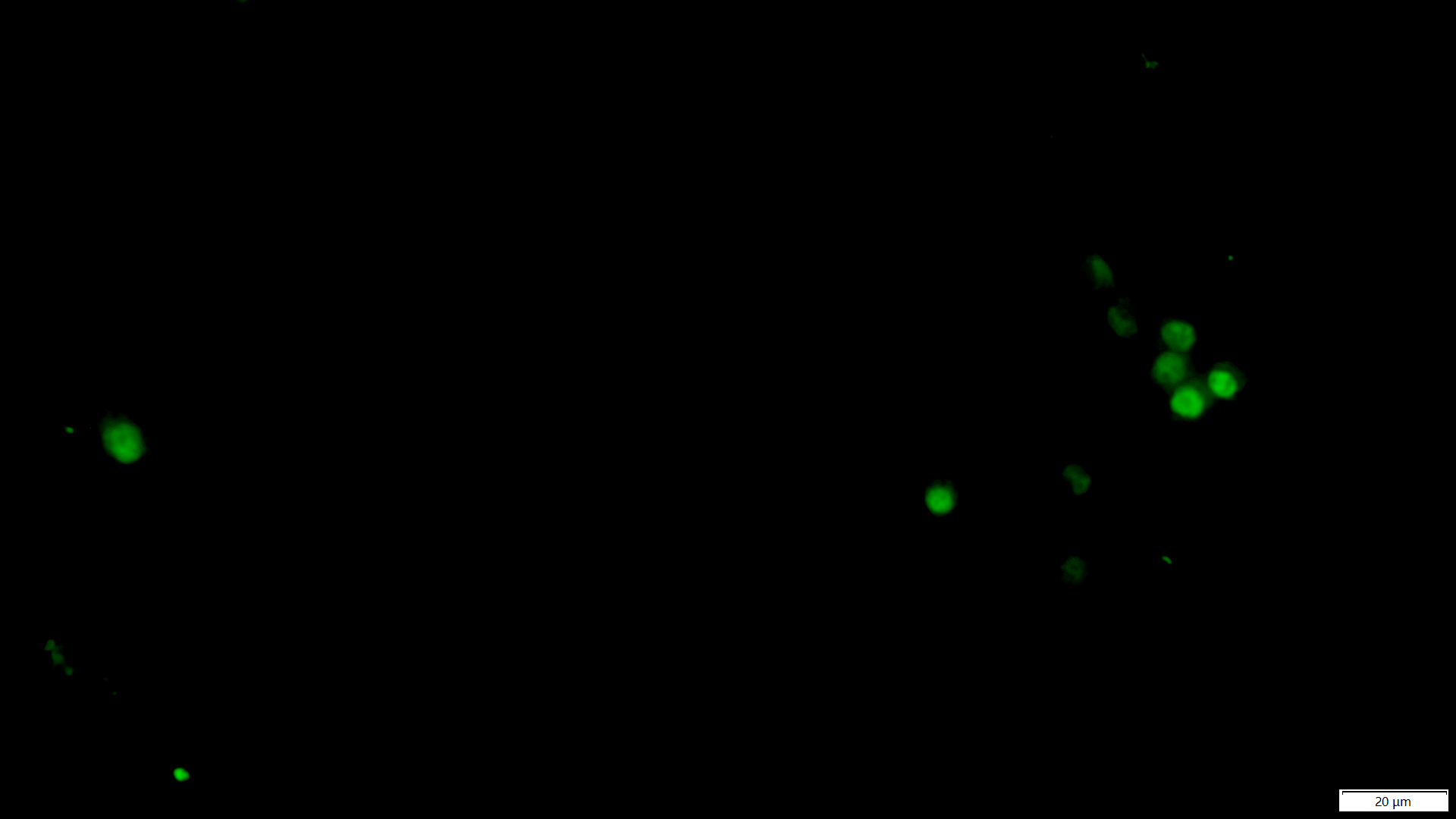

Supplement: Supplementary file 12 [file DataSheet2.zip › fluorescence co-localisation/P-gp-HA+HDAC5-Myc/GREEN HDAC5-Myc 40X.tif]

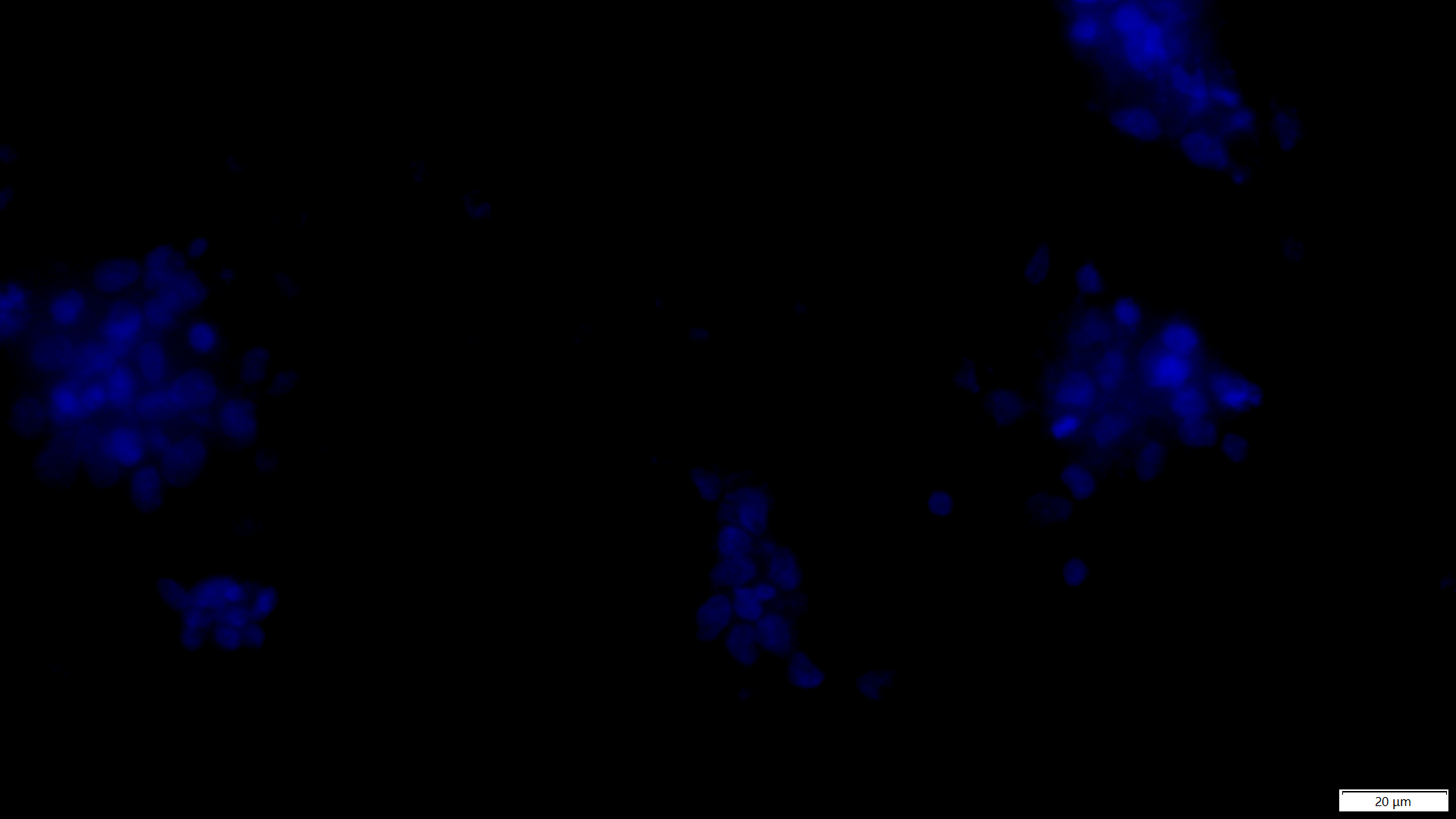

Supplement: Supplementary file 12 [file DataSheet2.zip › fluorescence co-localisation/P-gp-HA+HDAC5-Myc/P+H DAPI 40X.tif]

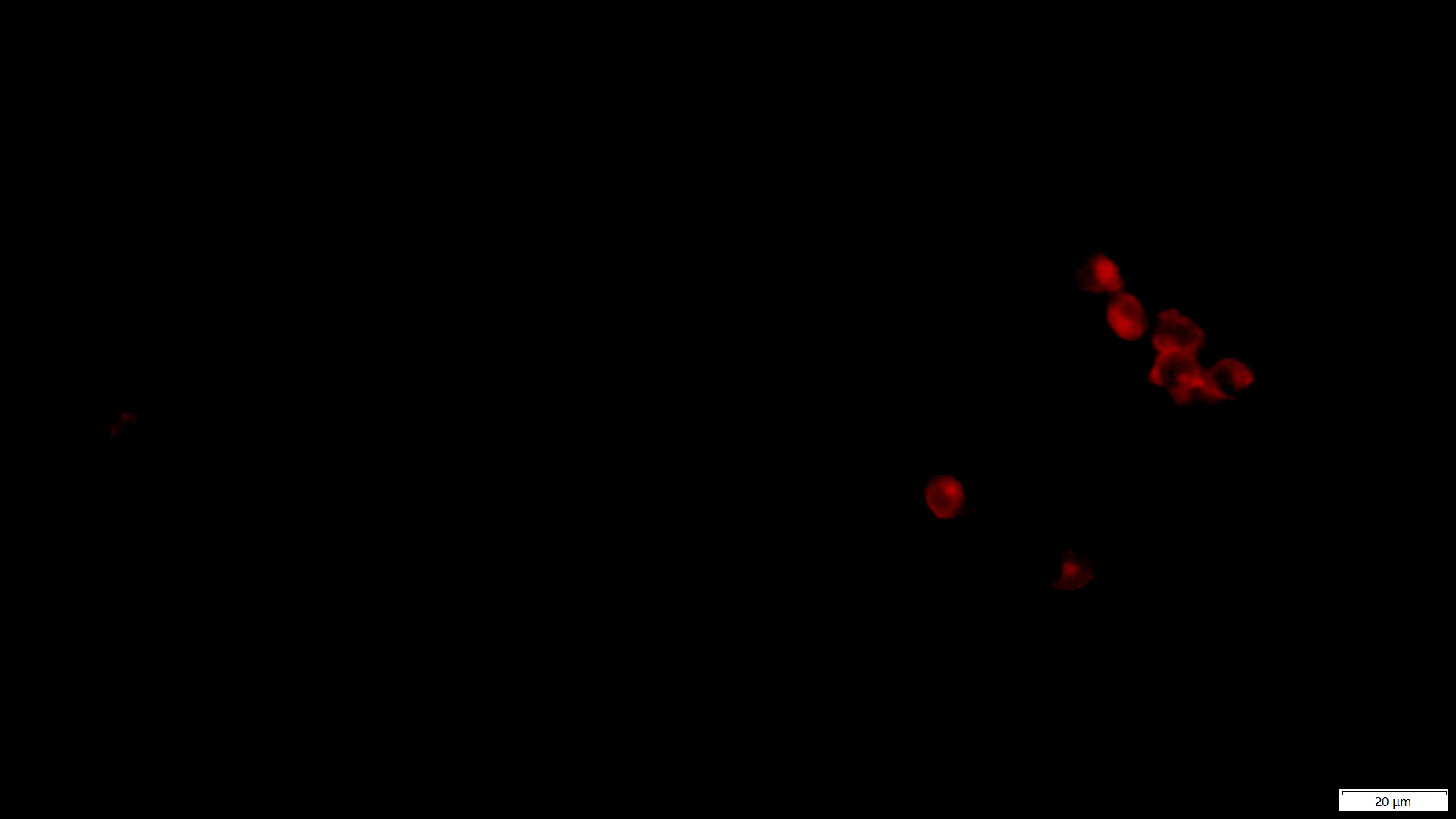

Supplement: Supplementary file 12 [file DataSheet2.zip › fluorescence co-localisation/P-gp-HA+HDAC5-Myc/P+H RED P-gp-HA 40X.tif]

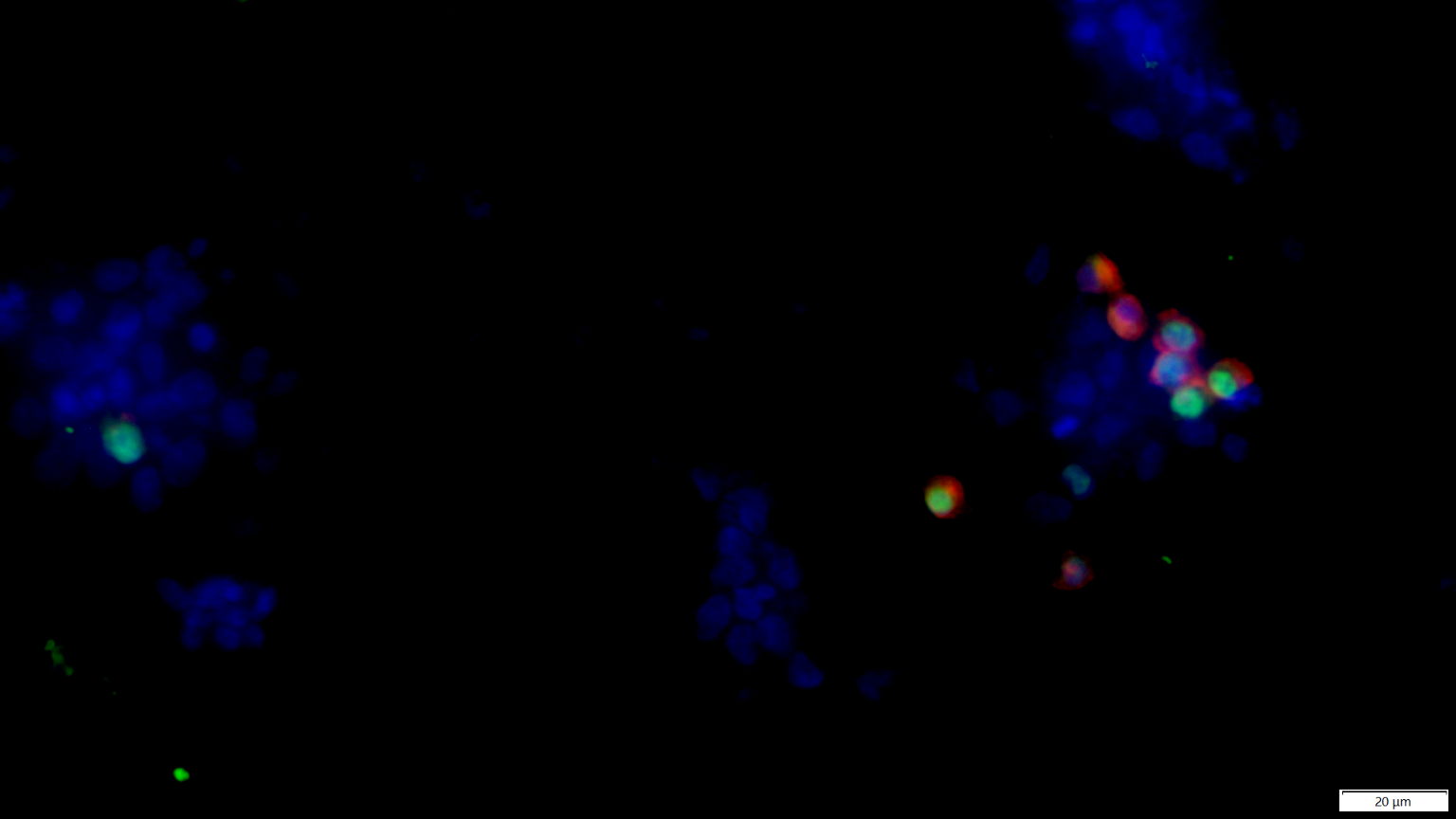

Supplement: Supplementary file 12 [file DataSheet2.zip › fluorescence co-localisation/P-gp-HA+HDAC5-Myc/P-gp-HA+HDAC5-Myc 40X-M.tif]

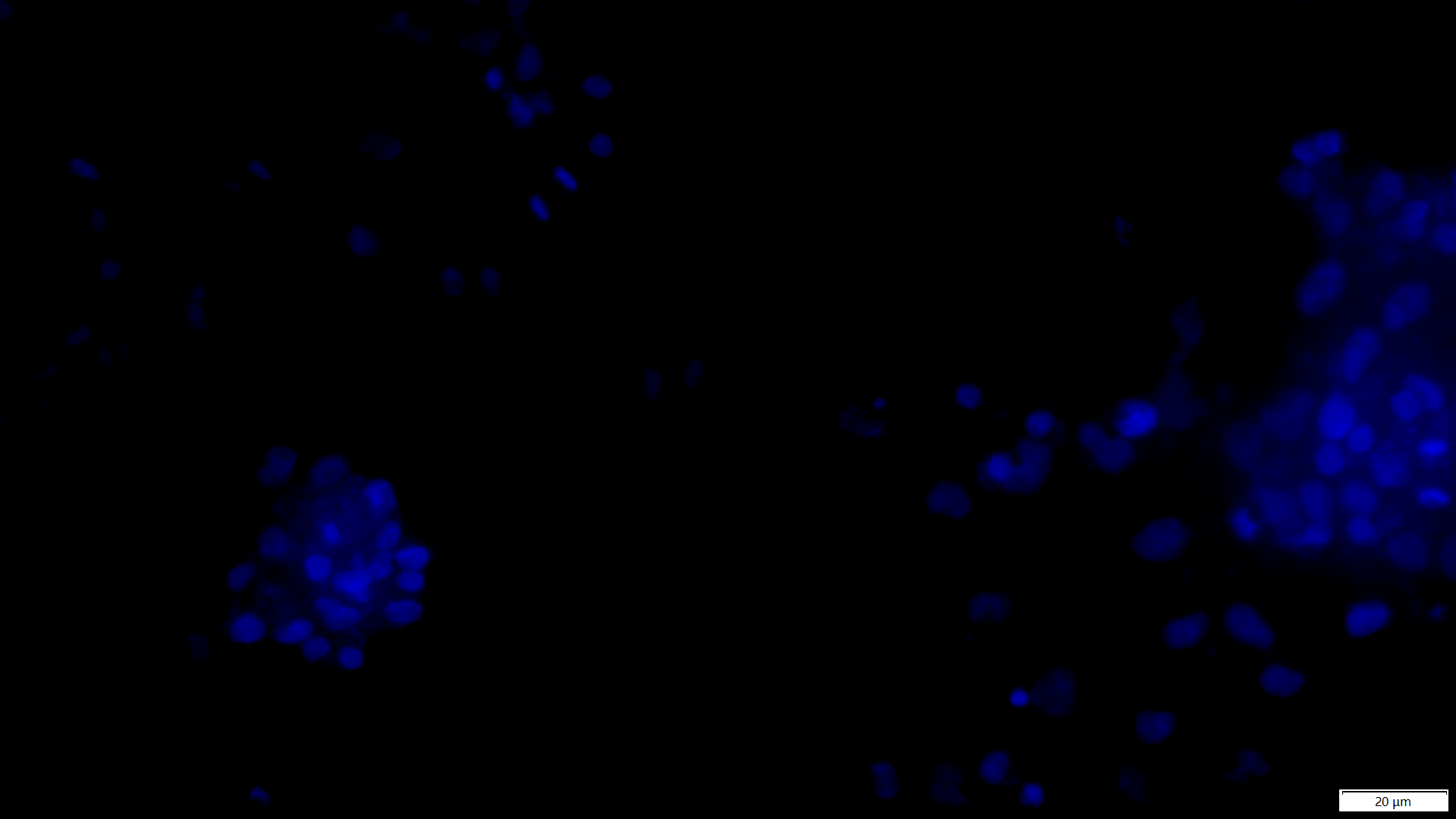

Supplement: Supplementary file 12 [file DataSheet2.zip › fluorescence co-localisation/P-gp-HA+SP1-Flag/P+S DAPI-1 40x.tif]

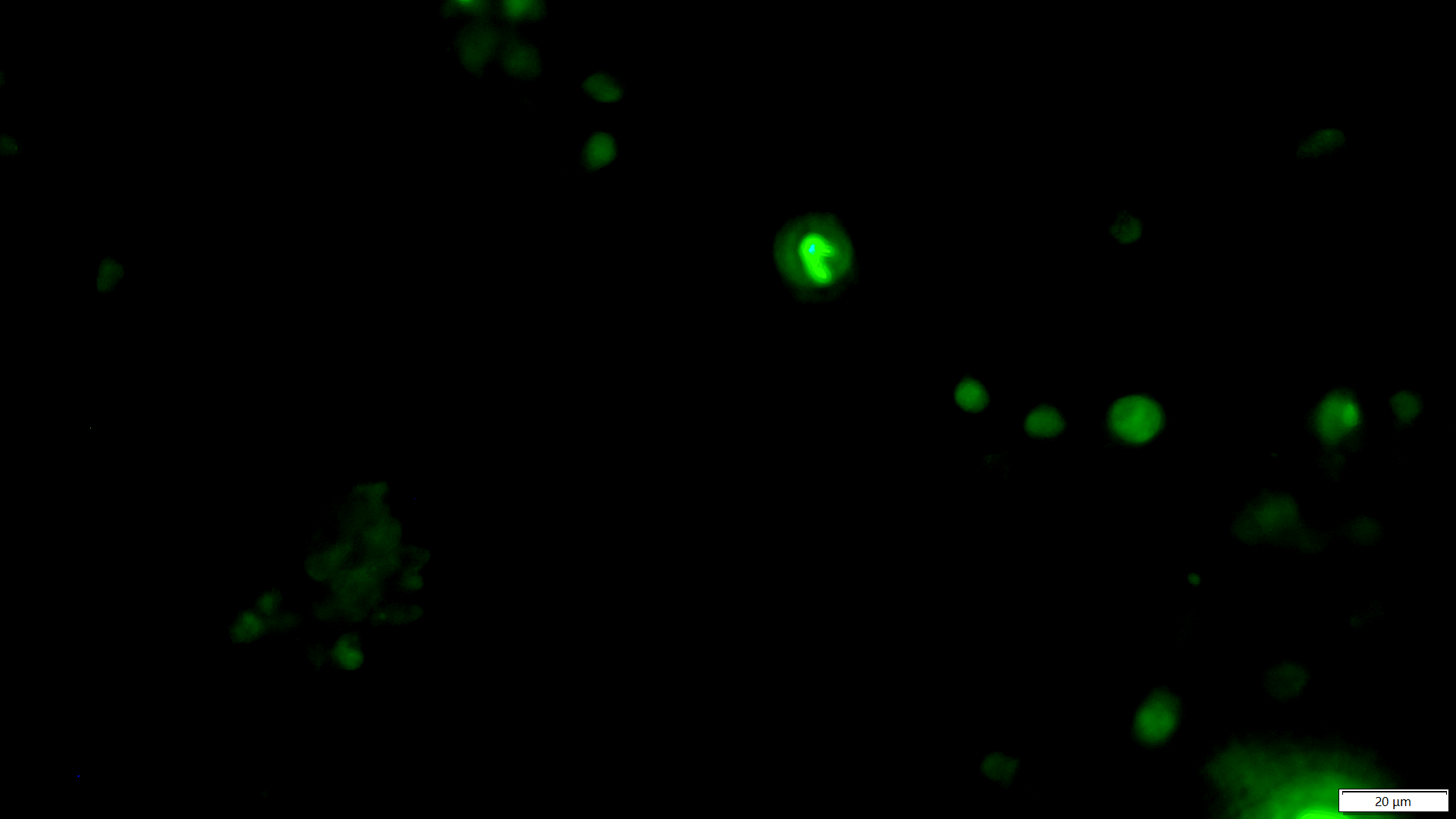

Supplement: Supplementary file 12 [file DataSheet2.zip › fluorescence co-localisation/P-gp-HA+SP1-Flag/P+S GREEN-SP1-Flag 40x.tif]

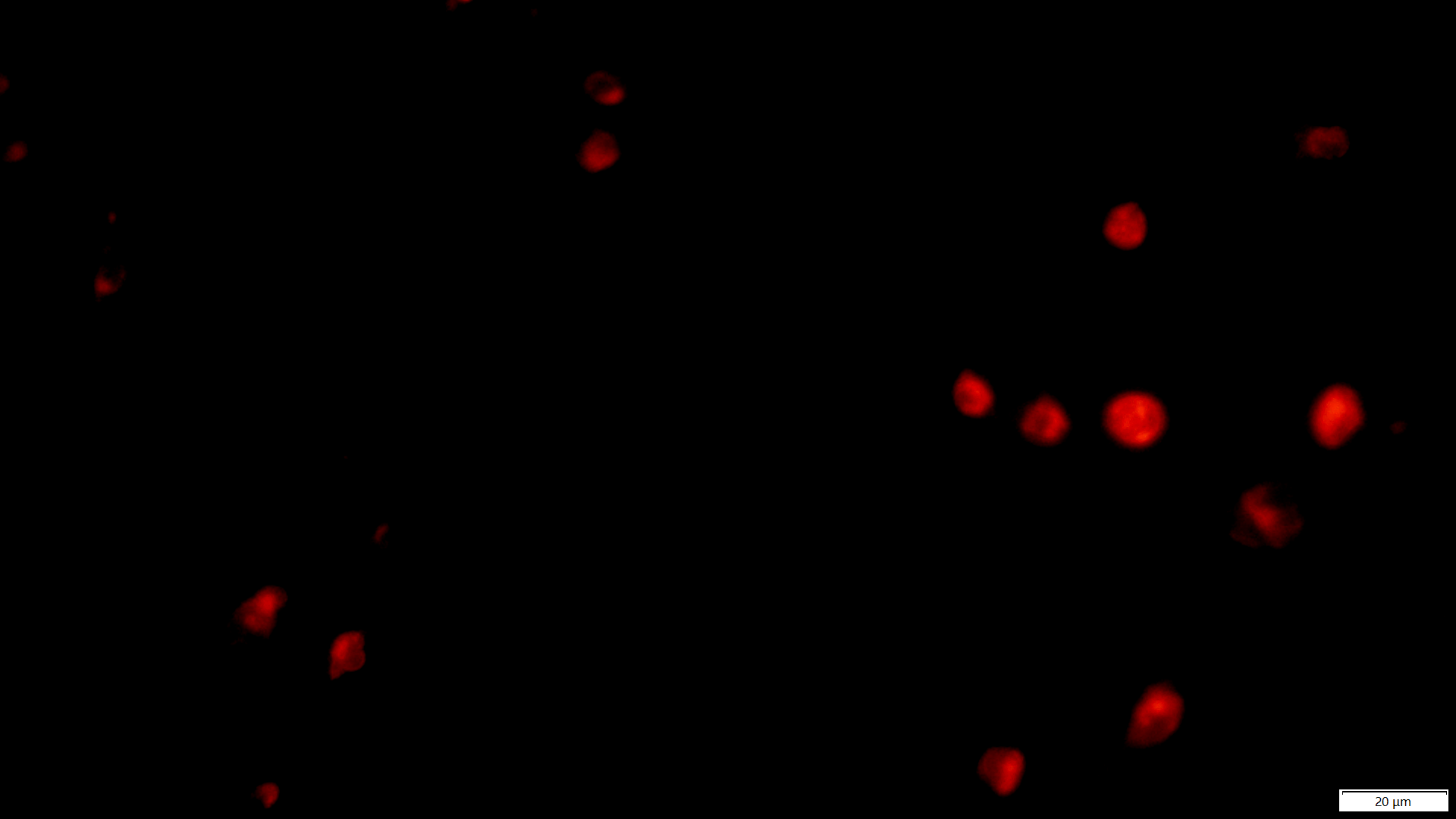

Supplement: Supplementary file 12 [file DataSheet2.zip › fluorescence co-localisation/P-gp-HA+SP1-Flag/P+S red P-gp-HA 40x.tif]

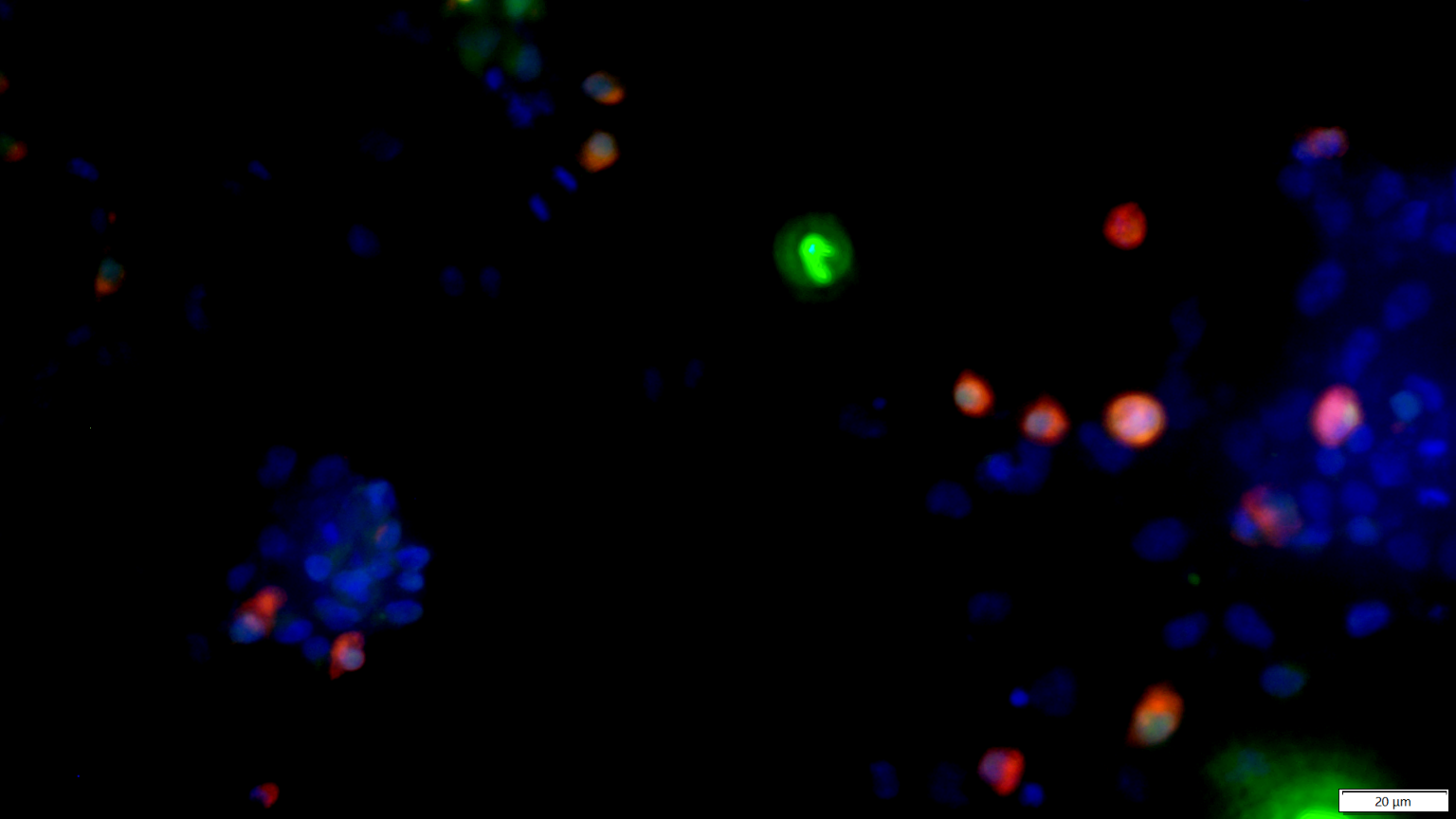

Supplement: Supplementary file 12 [file DataSheet2.zip › fluorescence co-localisation/P-gp-HA+SP1-Flag/P-gp-HA+SP1-Flag 40x-M.tif]

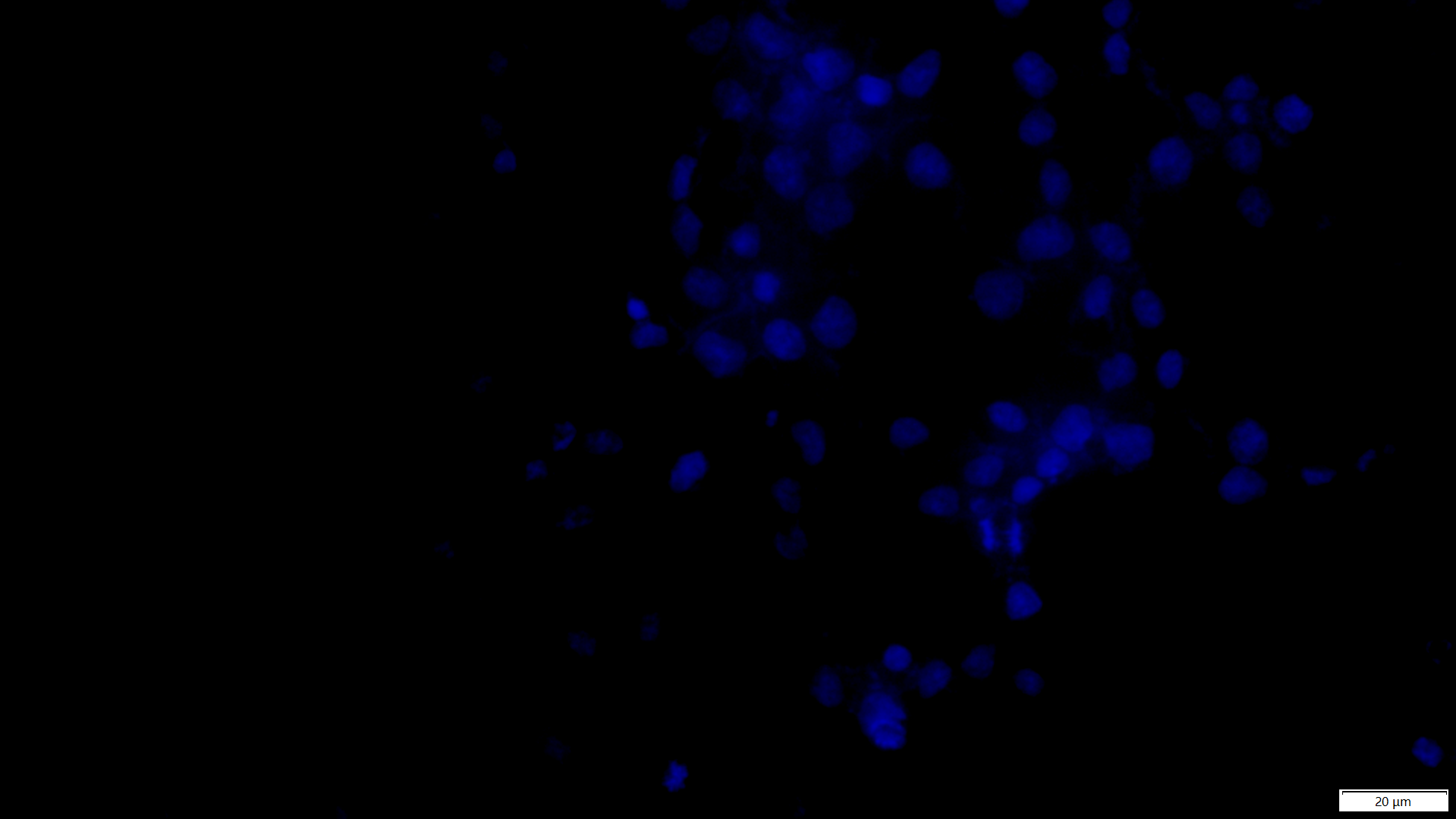

Supplement: Supplementary file 12 [file DataSheet2.zip › fluorescence co-localisation/SP1-Flag+HDAC5-Myc/S+H DAPI 40x.tif]

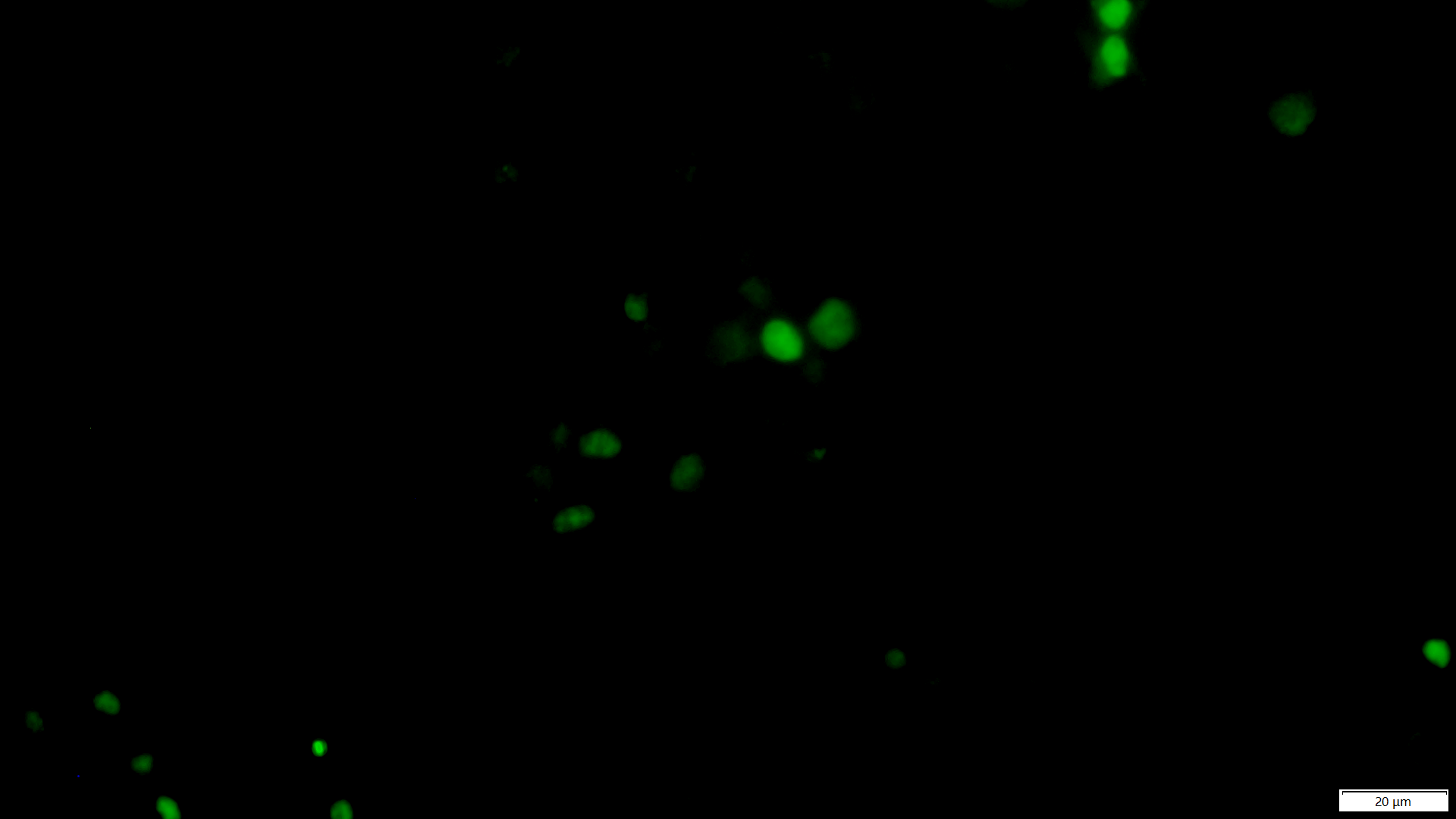

Supplement: Supplementary file 12 [file DataSheet2.zip › fluorescence co-localisation/SP1-Flag+HDAC5-Myc/S+H GERRN-SP1-Flag 40x.tif]

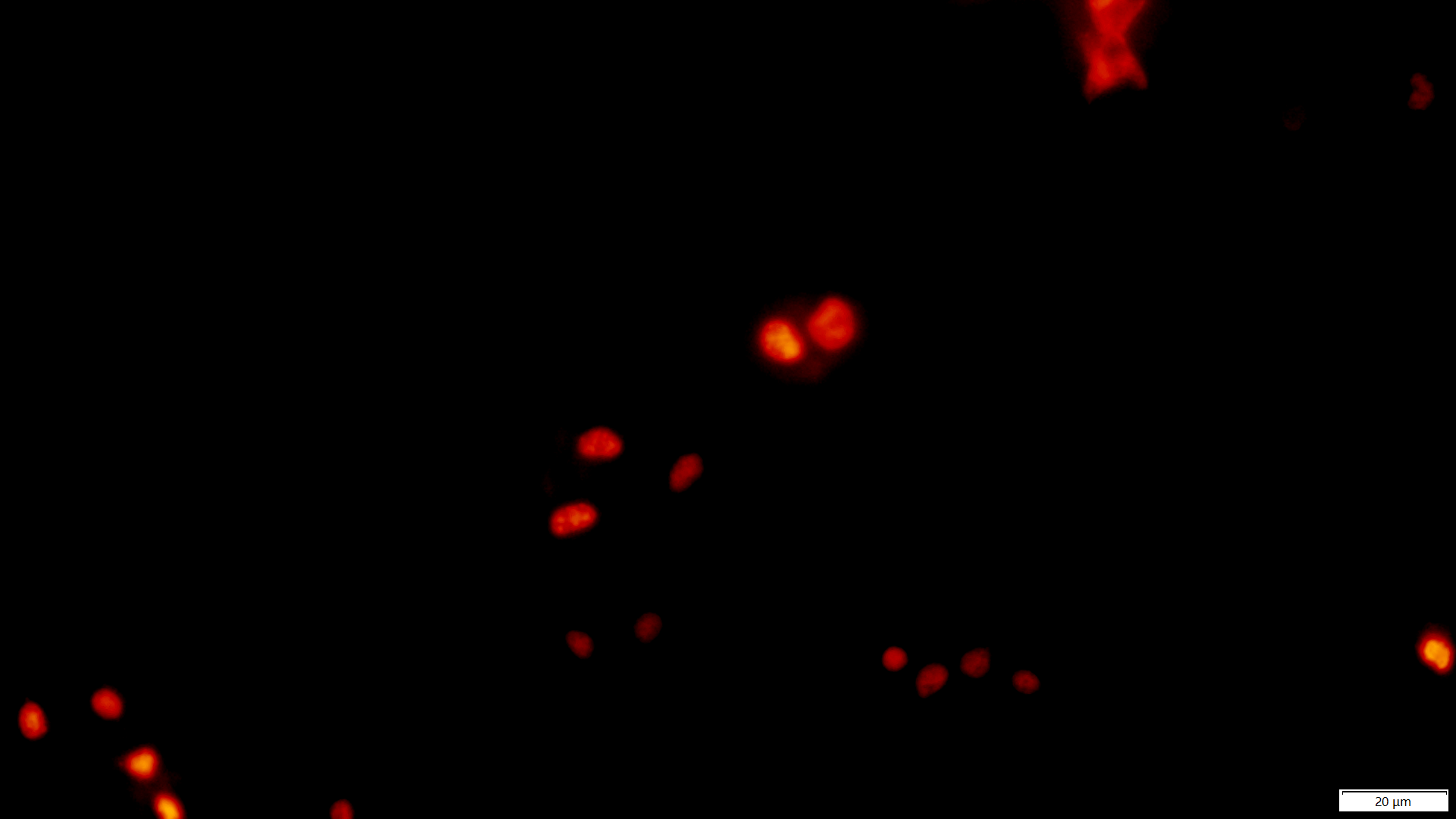

Supplement: Supplementary file 12 [file DataSheet2.zip › fluorescence co-localisation/SP1-Flag+HDAC5-Myc/S+H red-HDAC5-Myc 40x.tif]

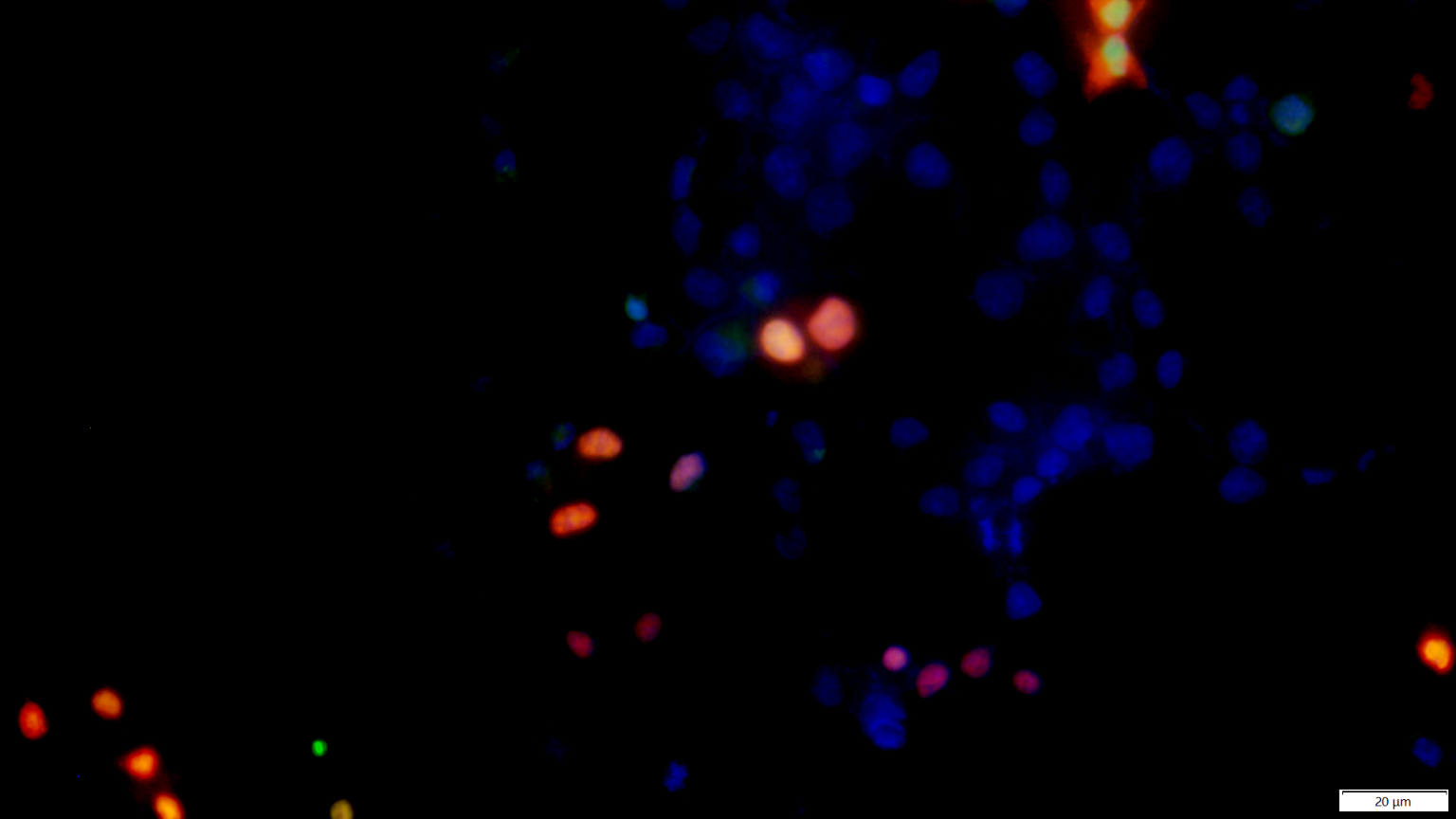

Supplement: Supplementary file 12 [file DataSheet2.zip › fluorescence co-localisation/SP1-Flag+HDAC5-Myc/SP1-Flag+HDAC5-Myc 40X-m.tif]

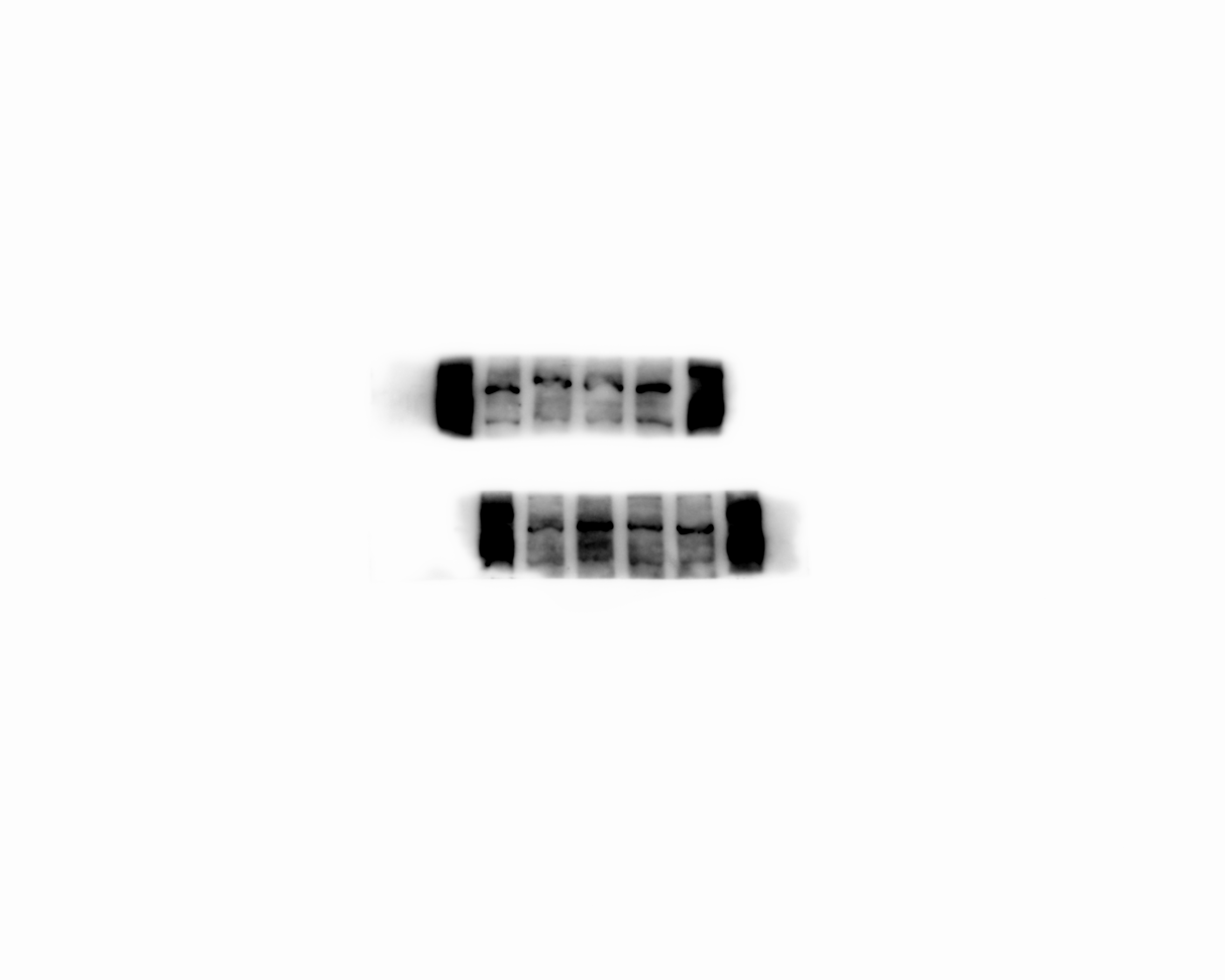

Supplement: Supplementary file 13 [file DataSheet5.zip › Normoxia vs Hypoxia¿CHIF-1a┴/HIF-1a┴.tif]

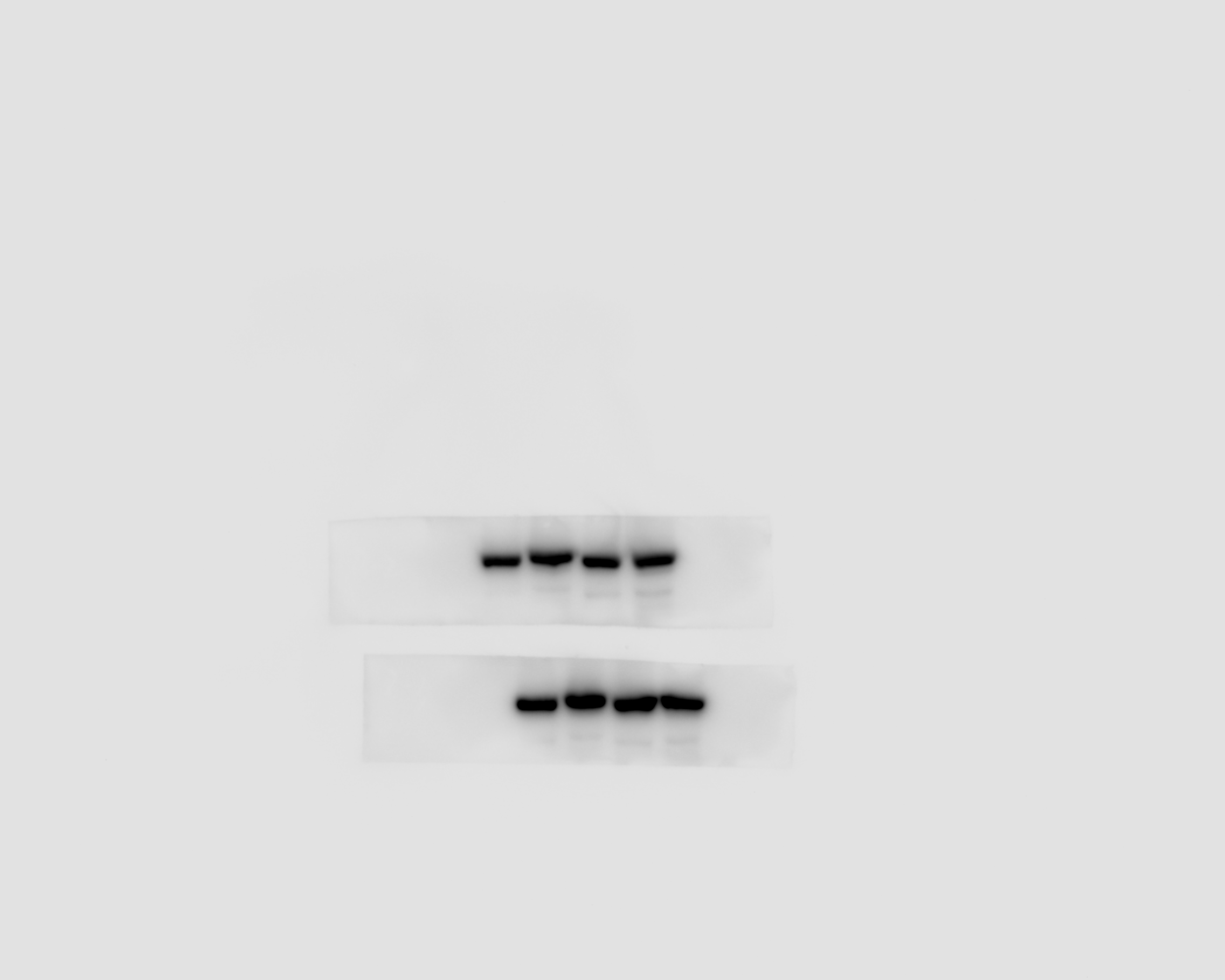

Supplement: Supplementary file 13 [file DataSheet5.zip › Normoxia vs Hypoxia¿CHIF-1a┴/a┬-actin.tif]

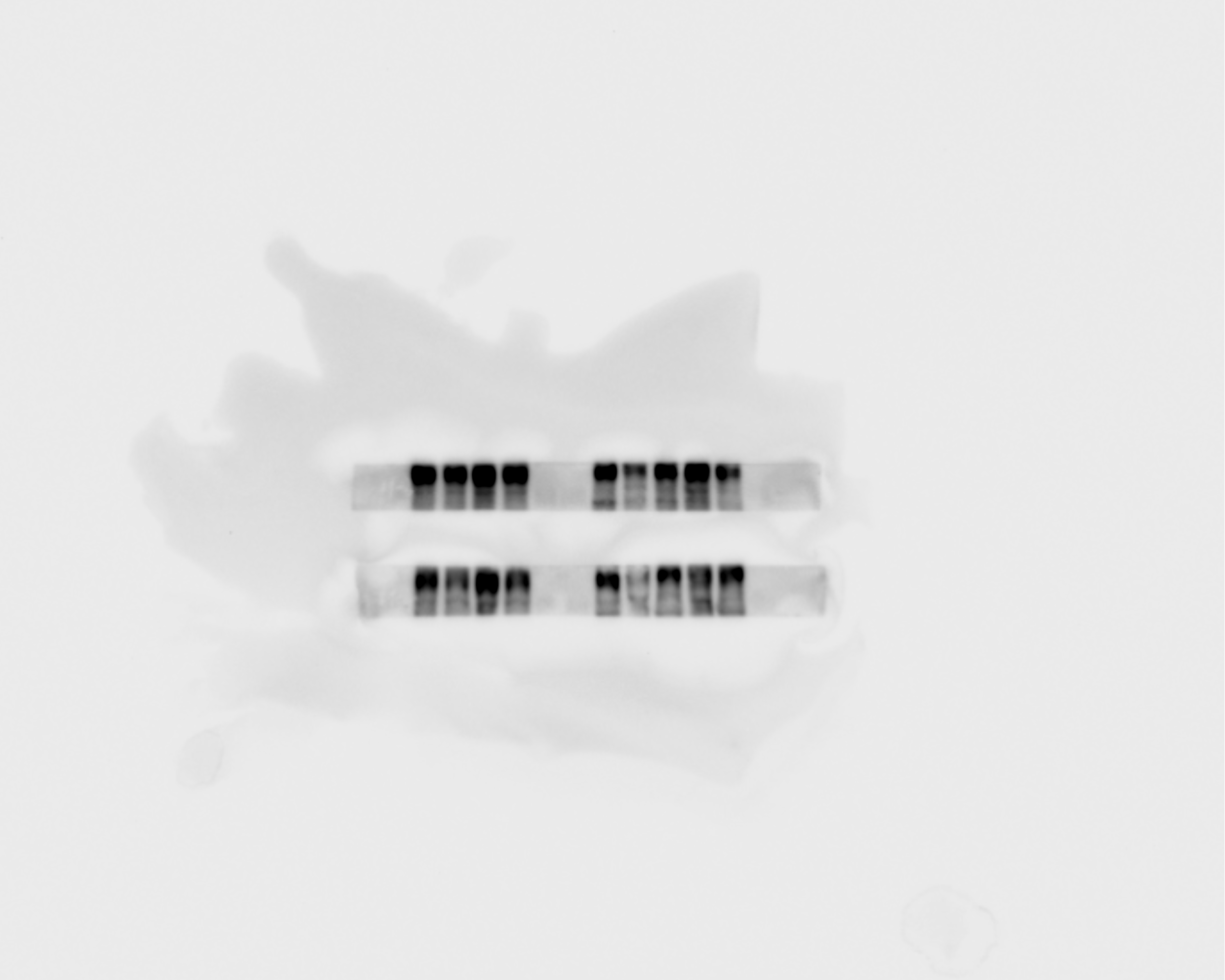

Supplement: Supplementary file 14 [file DataSheet7.zip › SAHA TSA and Bufexamac-HDAC5/Bufexamac-HDAC5.tif]

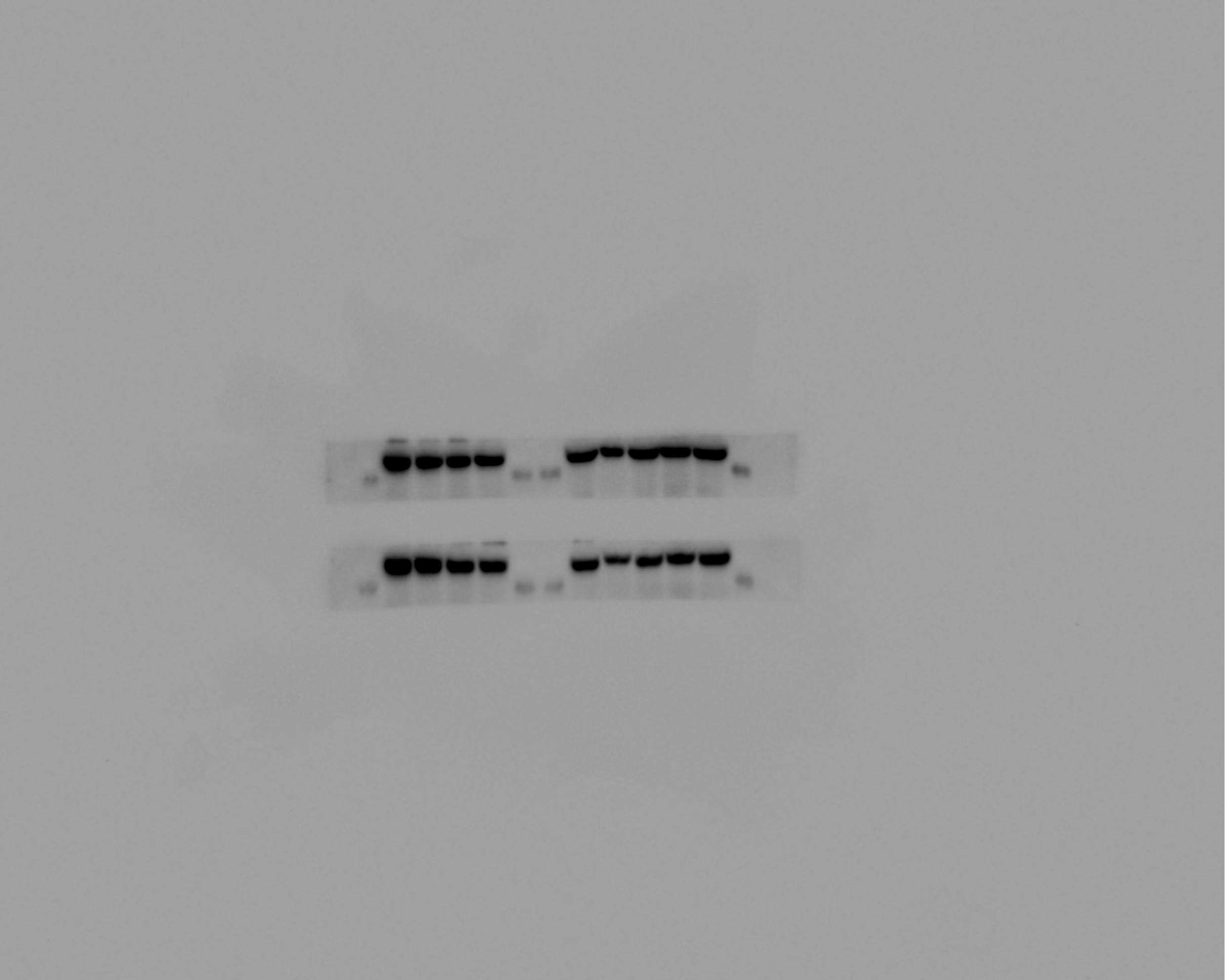

Supplement: Supplementary file 14 [file DataSheet7.zip › SAHA TSA and Bufexamac-HDAC5/Bufexamac-a┬-actin.tif]

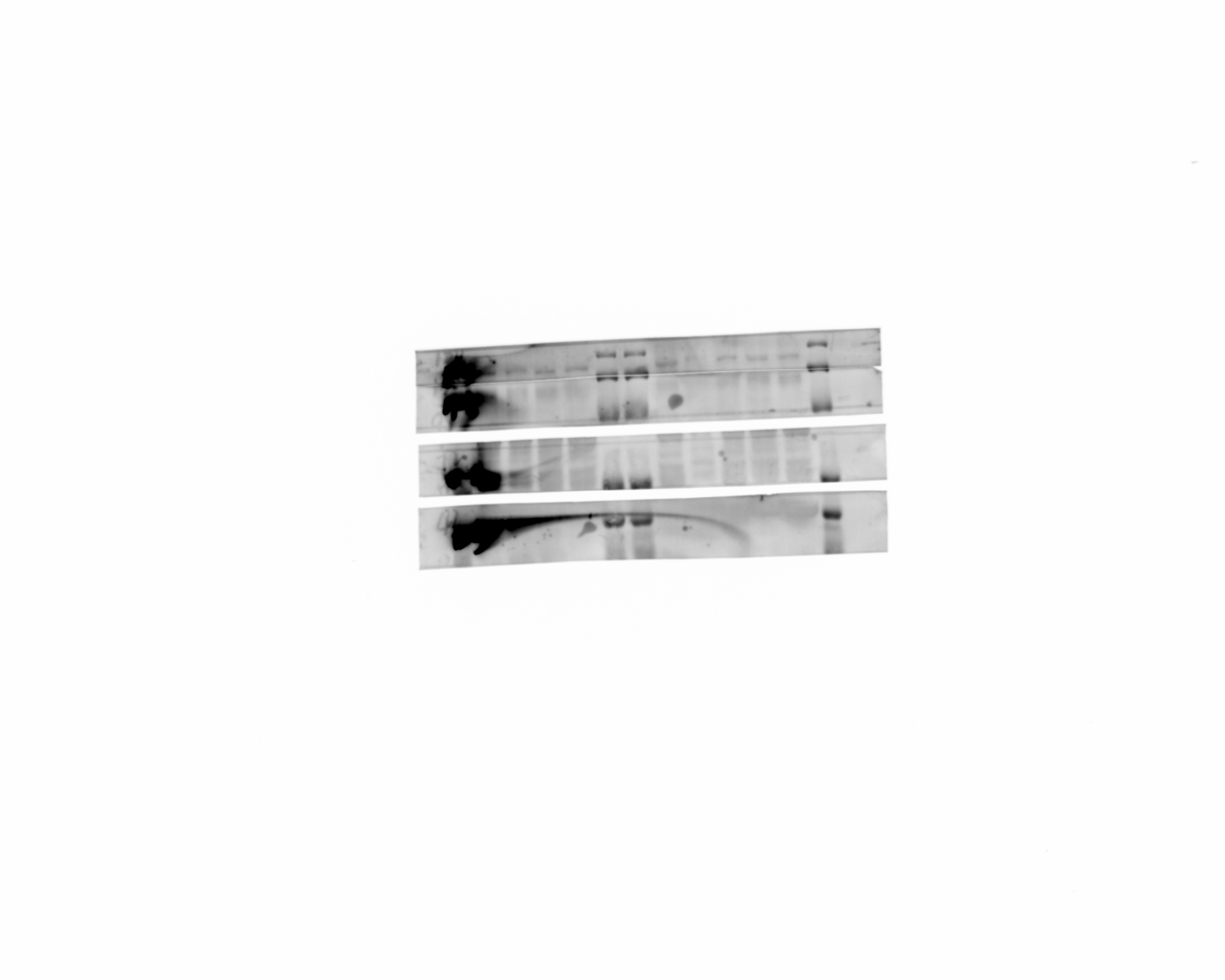

Supplement: Supplementary file 14 [file DataSheet7.zip › SAHA TSA and Bufexamac-HDAC5/SAHA andBufexamac-Western blot membrane cutting/Western blot membrane cutting..tif]

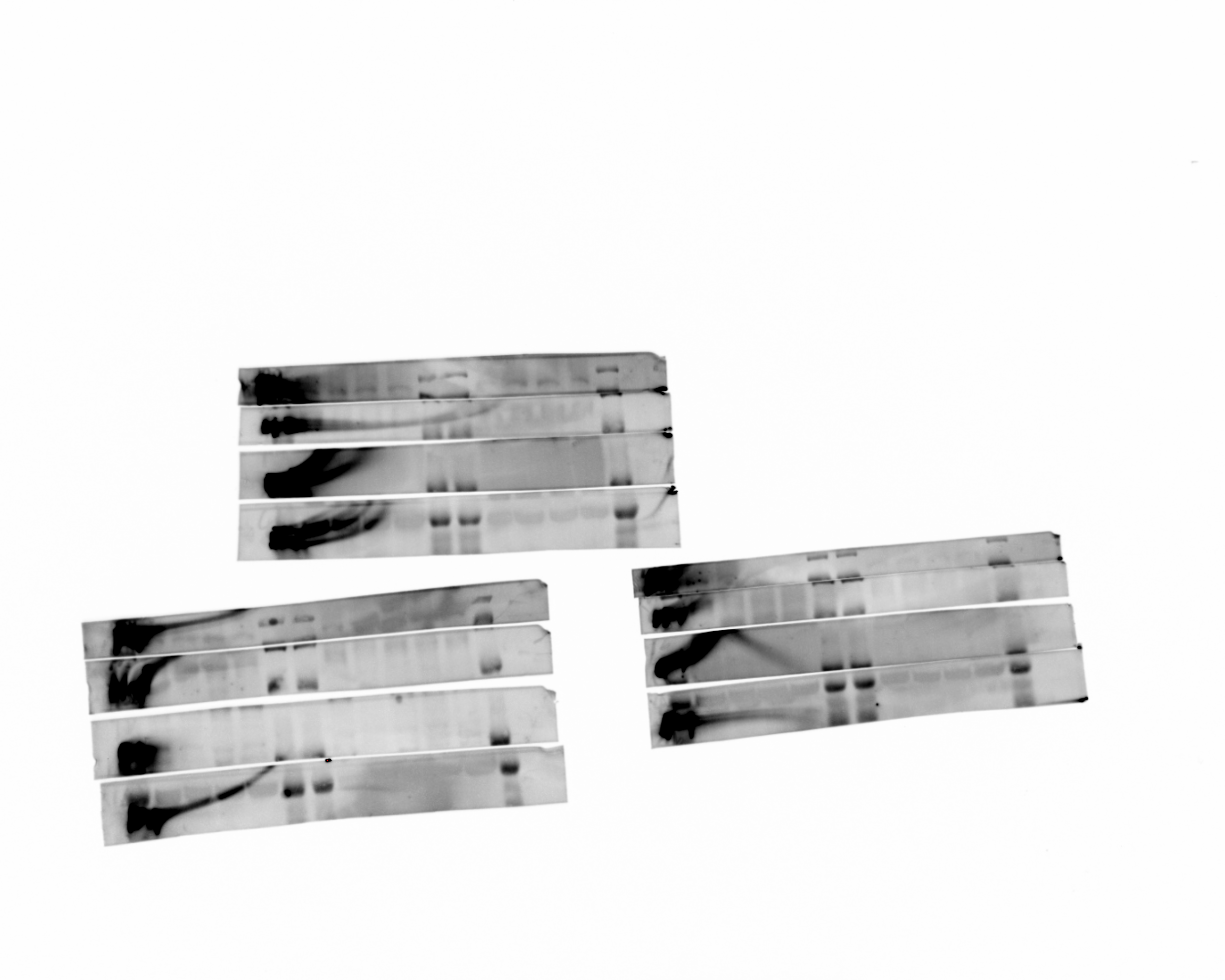

Supplement: Supplementary file 14 [file DataSheet7.zip › SAHA TSA and Bufexamac-HDAC5/SAHA andBufexamac-Western blot membrane cutting/Western blot membrane cutting1.tif]

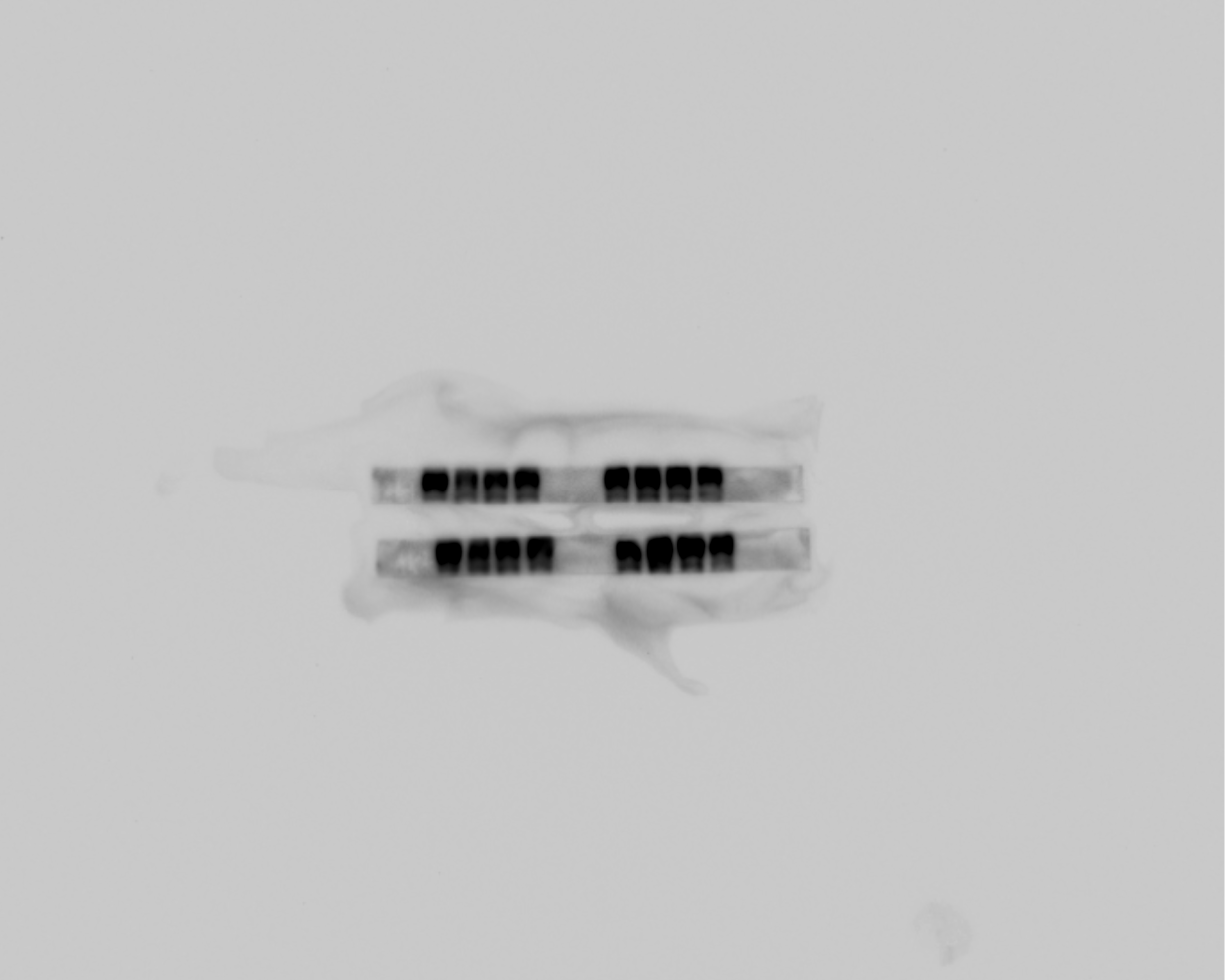

Supplement: Supplementary file 14 [file DataSheet7.zip › SAHA TSA and Bufexamac-HDAC5/SAHA-HDAC5.tif]

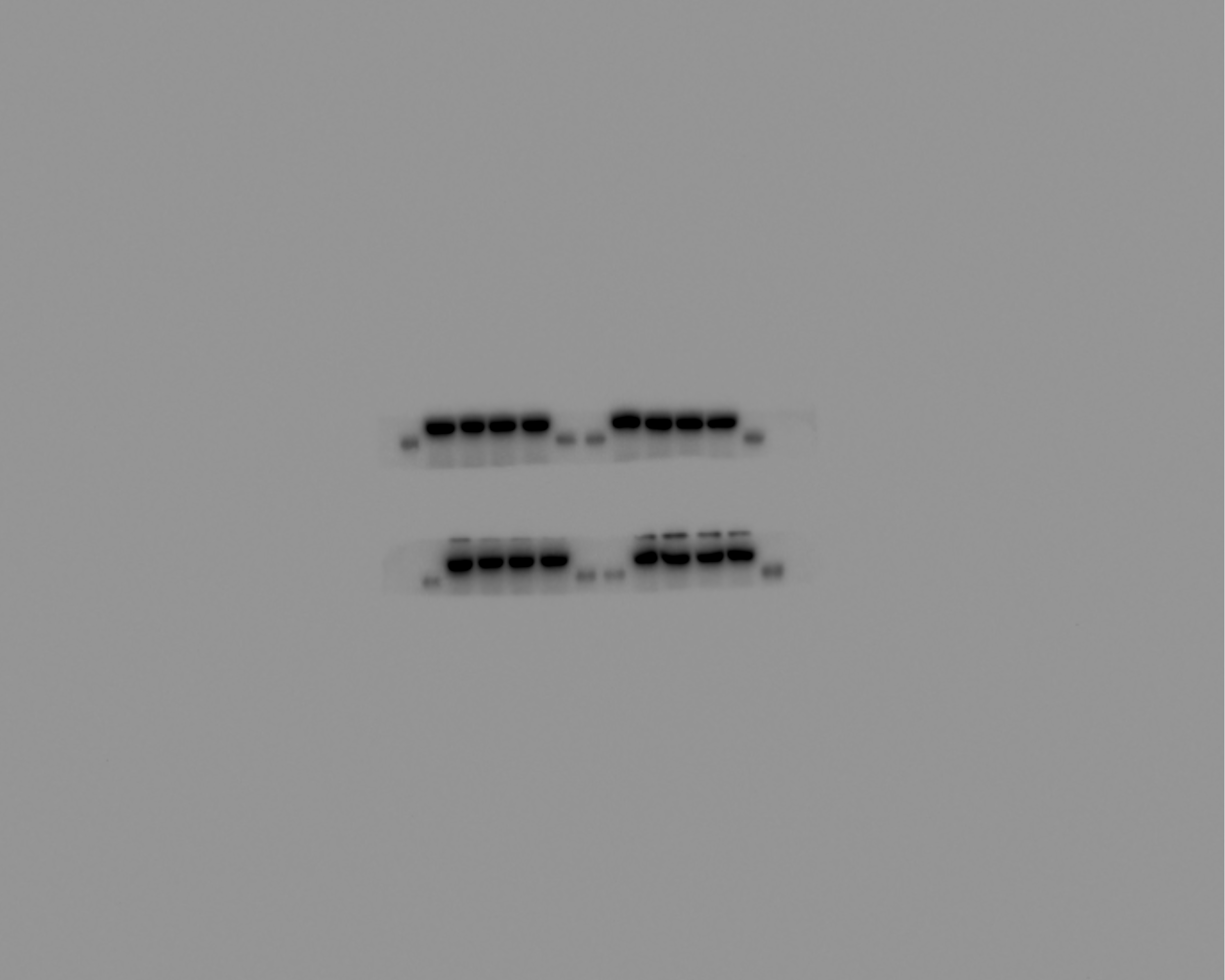

Supplement: Supplementary file 14 [file DataSheet7.zip › SAHA TSA and Bufexamac-HDAC5/SAHA-a┬-actin.tif]

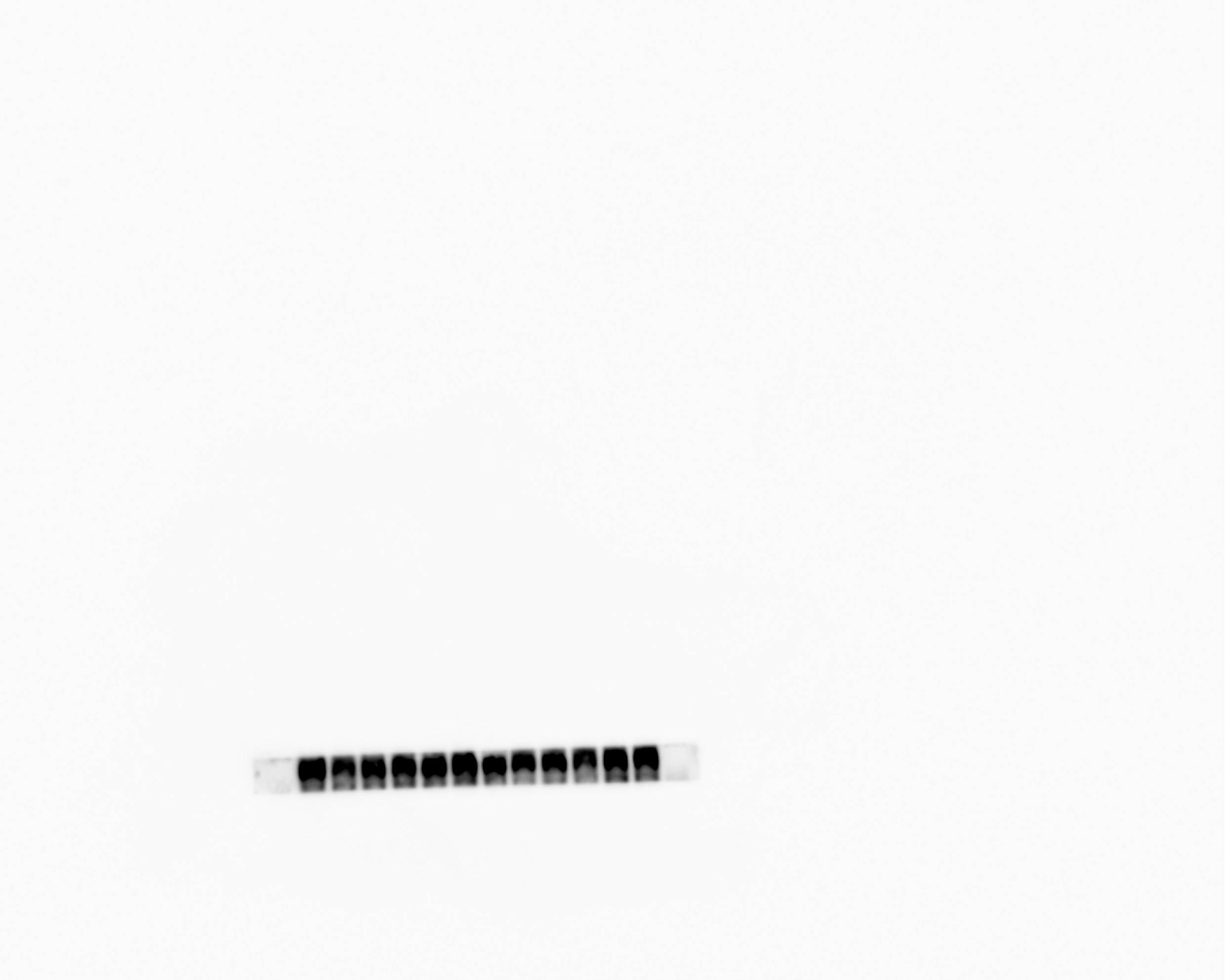

Supplement: Supplementary file 14 [file DataSheet7.zip › SAHA TSA and Bufexamac-HDAC5/TSA-HDAC5.tif]

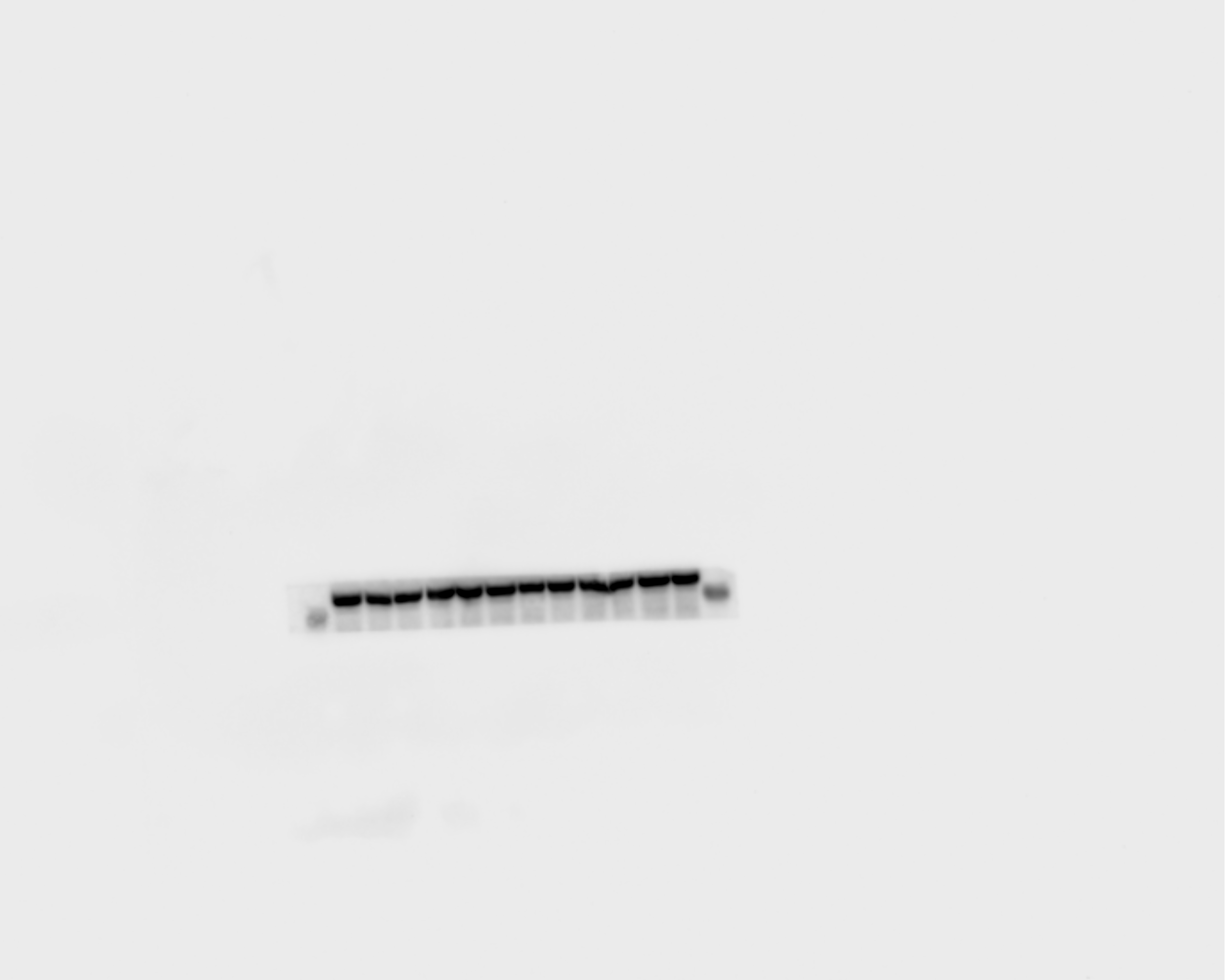

Supplement: Supplementary file 14 [file DataSheet7.zip › SAHA TSA and Bufexamac-HDAC5/TSA-a┬-actin.tif]
